# Safety and immunogenicity of 2-dose heterologous Ad26.ZEBOV, MVA-BN-Filo Ebola vaccination in children and adolescents in Africa: A randomised, placebo-controlled, multicentre Phase II clinical trial

**DOI:** 10.1371/journal.pmed.1003865

**Published:** 2022-01-11

**Authors:** Zacchaeus Anywaine, Houreratou Barry, Omu Anzala, Gaudensia Mutua, Sodiomon B. Sirima, Serge Eholie, Hannah Kibuuka, Christine Bétard, Laura Richert, Christine Lacabaratz, M. Juliana McElrath, Stephen C. De Rosa, Kristen W. Cohen, Georgi Shukarev, Michael Katwere, Cynthia Robinson, Auguste Gaddah, Dirk Heerwegh, Viki Bockstal, Kerstin Luhn, Maarten Leyssen, Rodolphe Thiébaut, Macaya Douoguih, on behalf of the EBL2002 Study group

**Affiliations:** 1 Medical Research Council/Uganda Virus Research Institute and London School of Hygiene and Tropical Medicine Uganda Research Unit, Entebbe, Uganda; 2 Centre MURAZ, Bobo-Dioulasso, Burkina Faso; 3 KAVI - Institute of Clinical Research University of Nairobi, Nairobi, Kenya; 4 Centre National de Recherche et de Formation sur le Paludisme (CNRFP), Unité de Recherche Clinique de Banfora, Banfora, Burkina Faso; 5 Unit of Infectious and Tropical Diseases, BPV3, Treichville University Teaching Hospital, Abidjan, Côte d’Ivoire; 6 Makerere University - Walter Reed Project, Kampala, Uganda; 7 Univ. Bordeaux, Inserm, Bordeaux Population Health Research Center, UMR 1219; Inria SISTM team; CHU Bordeaux; CIC 1401, EUCLID/F-CRIN Clinical Trials Platform, Bordeaux, France; 8 Vaccine Research Institute (VRI), Créteil, France; 9 Université Paris-Est Créteil, Faculté de Médecine, INSERM U955, Team 16, Créteil, France; 10 Vaccine and Infectious Disease Division, Fred Hutchinson Cancer Research Center, Seattle, Washington, United States of America; 11 Janssen Vaccines and Prevention, Leiden, the Netherlands; 12 Janssen Research & Development, Beerse, Belgium; Burnet Institute, AUSTRALIA

## Abstract

**Background:**

Reoccurring Ebola outbreaks in West and Central Africa have led to serious illness and death in thousands of adults and children. The objective of this study was to assess safety, tolerability, and immunogenicity of the heterologous 2-dose Ad26.ZEBOV, MVA-BN-Filo vaccination regimen in adolescents and children in Africa.

**Methods and findings:**

In this multicentre, randomised, observer-blind, placebo-controlled Phase II study, 131 adolescents (12 to 17 years old) and 132 children (4 to 11 years old) were enrolled from Eastern and Western Africa and randomised 5:1 to receive study vaccines or placebo. Vaccine groups received intramuscular injections of Ad26.ZEBOV (5 × 10^10^ viral particles) and MVA-BN-Filo (1 × 10^8^ infectious units) 28 or 56 days apart; placebo recipients received saline. Primary outcomes were safety and tolerability. Solicited adverse events (AEs) were recorded until 7 days after each vaccination and serious AEs (SAEs) throughout the study. Secondary and exploratory outcomes were humoral immune responses (binding and neutralising Ebola virus [EBOV] glycoprotein [GP]-specific antibodies), up to 1 year after the first dose. Enrolment began on February 26, 2016, and the date of last participant last visit was November 28, 2018. Of the 263 participants enrolled, 217 (109 adolescents, 108 children) received the 2-dose regimen, and 43 (20 adolescents, 23 children) received 2 placebo doses. Median age was 14.0 (range 11 to 17) and 7.0 (range 4 to 11) years for adolescents and children, respectively. Fifty-four percent of the adolescents and 51% of the children were male. All participants were Africans, and, although there was a slight male preponderance overall, the groups were well balanced. No vaccine-related SAEs were reported; solicited AEs were mostly mild/moderate. Twenty-one days post-MVA-BN-Filo vaccination, binding antibody responses against EBOV GP were observed in 100% of vaccinees (106 adolescents, 104 children). Geometric mean concentrations tended to be higher after the 56-day interval (adolescents 13,532 ELISA units [EU]/mL, children 17,388 EU/mL) than the 28-day interval (adolescents 6,993 EU/mL, children 8,007 EU/mL). Humoral responses persisted at least up to Day 365.

A limitation of the study is that the follow-up period was limited to 365 days for the majority of the participants, and so it was not possible to determine whether immune responses persisted beyond this time period. Additionally, formal statistical comparisons were not preplanned but were only performed post hoc.

**Conclusions:**

The heterologous 2-dose vaccination was well tolerated in African adolescents and children with no vaccine-related SAEs. All vaccinees displayed anti-EBOV GP antibodies after the 2-dose regimen, with higher responses in the 56-day interval groups. The frequency of pyrexia after vaccine or placebo was higher in children than in adolescents. These data supported the prophylactic indication against EBOV disease in a paediatric population, as licenced in the EU.

**Trial registration:**

ClinicalTrials.gov NCT02564523.

## Abstract 2

Zacchaeus Anywaine and co-workers study safety and immunogenicity of an Ebola vaccine among children and adolescents across four African countries.

## Author summary

**Why was the study done?:**

- There have been larger and more extensive Ebola virus disease (EVD) outbreaks in Africa in the past decade with no licenced treatments available. As such, there is an unmet medical need for prophylactic Ebola vaccines.
- This study was performed to evaluate whether a 2-dose heterologous Ad26.ZEBOV, MVA-BN-Filo Ebola vaccination was safe and immunogenic in healthy African children.

**What did the researchers do and find?:**

- In this randomised, placebo-controlled, Phase II clinical trial, the Ad26.ZEBOV, MVA-BN-Filo Ebola vaccination regimen was administered to African participants in 2 age cohorts (12 to 17 and 4 to 11 years).
- No vaccine-related serious adverse events were reported, and robust immune responses were induced in both adolescents and children after receiving the active 2-dose regimen.

**What do these findings mean?:**

- These data support the use of the Ad26.ZEBOV, MVA-BN-Filo vaccination regimen in African adolescents and children at risk of Ebola infection.
- Although vaccination according to a 28-day regimen may lead to protection against EVD in a shorter time frame, vaccination according to a 56-day regimen results in higher EBOV GP binding and neutralising antibody responses.
- The observation that Ad26 preexisting immunity in the majority of participants does not affect the EBOV GP-specific antibody responses postvaccination augurs well for the use of this vaccine regimen even in regions with a high prevalence of preexisting Ad26 seropositivity.

## Introduction

Ebola disease due to the Ebola virus (EBOV) has been responsible for several major outbreaks in Africa since first being identified in 1976 [1]. The 2 largest outbreaks have been in Guinea, Liberia, and Sierra Leone (2014 to 2016) [2], and the Democratic Republic of the Congo (2018 to 2020) [3]. The lack of effective therapy and the lethality of Ebola virus disease (EVD) makes effective vaccination a major medical need, which has driven several vaccine development programmes, typically based on the presentation of the viral surface glycoprotein (GP).

A 1-dose, recombinant, replication-competent vesicular stomatitis viral vectored vaccine expressing the Kikwit GP (rVSV-ZEBOV-GP, Ervebo, Merck) demonstrated 97.5% to 100% efficacy when used in a ring-vaccination strategy, and it was approved by the FDA and EMA for use in persons ≥18 years [4–7]. Janssen Vaccines & Prevention B.V. has developed a 2-dose heterologous regimen, which recently received approval under exceptional circumstances by the EMA for prophylactic use in persons aged 1 year and older [8–10]. This regimen comprises Ad26.ZEBOV (Zabdeno) and MVA-BN-Filo (Mvabea) administered approximately 8 weeks apart. Phase I/II studies in European and African adults established acceptable safety, tolerability, and robust immunogenicity of the 2-dose regimen with intervals of 28 or 56 days between vaccinations [11–16]. Both vaccines are recommended by SAGE in outbreak settings for infants and children from birth to 17 years of age [17].

We previously reported a Phase II study in healthy and HIV-infected adult African participants to assess the safety and immunogenicity of different timing intervals between vaccinations in a heterologous regimen [16]. This report presents the safety and immunogenicity results in 12- to 17-year-old adolescents and 4- to 11-year-old children from the same study.

## Methods

### Study overview

This randomised, observer-blind, placebo-controlled Phase II study was performed in 7 sites in 4 African countries: Burkina Faso (Bobo-Dioulasso and Banfora), Côte d’Ivoire (Abidjan and Toupah/Ousrou), Kenya (Nairobi), and Uganda (Masaka and Kampala). The protocol was approved by the local Independent Ethics Committees and/or Institutional Review Boards at each site, with central ethics approval in Burkina Faso provided by Burkina Faso Central Ethics Committee (approval number: 2017-02-023) and performed in accordance with Declaration of Helsinki and Good Clinical Practice guidelines and local regulations. The trial was registered with ClinicalTrials.gov NCT02564523. The study protocol, including the CONSORT checklist, can be found in S1 Study Protocol and S1 CONSORT Checklist.

The primary objective was to assess the safety and tolerability in adolescents (12 to 17 years old) and children (4 to 11 years old) of the 2-dose heterologous vaccine regimen with Ad26.ZEBOV administered on day 1 and MVA-BN-Filo on day 29 or 57. Secondary and exploratory objectives included assessments of humoral and cellular immune responses to EBOV GP at different time points up to 1 year after Ad26.ZEBOV, and impact of baseline neutralising antibodies to Ad26 on the response.

### Study participants, randomisation, and blinding

Study participants were recruited from the general population. Information was shared through community meetings, posters, and school-based conferences where volunteers and their legal guardian were invited to study sites. Signed informed consent was obtained from legal guardians, and signed informed assent was obtained from children over 6 or 12 years old (depending on country) before inclusion. On the day of randomisation, eligible participants aged 12 to 17 years or 4 to 11 years (inclusive) had to be healthy based on investigator’s judgement, medical history, physical examination, vital signs, and clinical laboratory testing. Major exclusion criteria comprised any history of Ebola infection or prior exposure to EBOV (including travel to an epidemic Ebola area within 1 month of screening), previous receipt of a candidate Ebola vaccine or any experimental candidate Ad26- or MVA-based vaccine, and a known allergy or history of anaphylaxis or other serious adverse reactions to vaccines or to vaccine products.

Participants enrolled in both age cohorts were randomised 1:1 to the 28-day or 56-day dosing interval group using an interactive web response system (IWRS) provided by the sponsor, which was balanced using randomly permuted blocks and stratified by sites’ peripheral blood mononuclear cell (PBMC) sampling capability. The IWRS assigned each participant a unique code, which was maintained within the IWRS and was not provided to the investigators. Participants in each group were further randomly assigned 5:1 to receive either Ad26.ZEBOV or placebo on day 1 and MVA-BN-Filo or placebo on day 29 or 57. Participants, investigators, and study staff remained blinded to the allocation of investigational products throughout the study. Vaccines and placebo were prepared by a site pharmacist who was the only unblinded member of staff. The pharmacist received the randomisation number and allocated the right study vaccine to the participant. Masking tape was used to cover the dispensing syringes containing the study vaccine/placebo allocated to each study participant.

### Sample size determination

The planned sample size for the adolescent and children cohorts included 264 participants who were to receive either the 2-dose regimen or placebo, to substantially contribute to the overall safety database of the regimen. In each cohort, a total of 110 participants were to receive Ad26.ZEBOV and MVA-BN-Filo (55 participants in each 28-day or 56-day interval group); 22 participants were to receive placebo. Sample size determination was not based on formal statistical hypothesis testing. However, in case a specific adverse event (AE) was not observed, the one-sided 97.5% upper confidence limit of the true incidence rate of this AE was less than 6.5% and 3.3% for a sample size of 55 and 110 participants, respectively.

### Vaccines

The heterologous 2-dose vaccine regimen comprises Ad26.ZEBOV (Zabdeno, Janssen-Cilag International N.V., Leiden, the Netherlands), a recombinant, replication-incompetent Ad26-based vector that encodes the full-length EBOV Mayinga GP, and MVA-BN-Filo (Mvabea, Bavarian Nordic, Kvistgård, Denmark), a recombinant, nonreplicating, modified vaccinia Ankara-vectored vaccine encoding EBOV Mayinga, Sudan virus Gulu, and Marburg virus Musoke variant GPs, as well as Tai Forest virus nucleoprotein. Ad26.ZEBOV containing 5 × 10^10^ viral particles on day 1 was followed by MVA-BN-Filo 1 × 10^8^ infectious units (Inf.U) on day 29 or day 57. Both vaccines were supplied as frozen liquid suspensions and thawed before use. Both vaccines and placebo (0.9% saline) were administered by intramuscular injection (0.5 mL) in the deltoid.

### Safety and tolerability assessments

After each vaccination, participants were assessed at 30 and 60 minutes for any immediate AEs. Solicited local and systemic AEs and daily body temperature were recorded for up to 7 days in diary cards, and unsolicited AEs were evaluated until 42 days after the second vaccination. Serious AEs (SAEs) were to be reported to investigators at any time. AEs were graded as 1 (mild), 2 (moderate), or 3 (severe) according to the adapted US Division of Microbiology and Infectious Diseases Toxicity Tables (2007) [18]. An independent data monitoring committee was established to regularly assess safety data.

### Immunogenicity assessments

Blood samples were taken on day 1, day 29 or 57, day 50 or 78, day 209 or 237, and day 365. EBOV GP-specific total IgG binding antibody concentrations were measured in sera by the Filovirus Animal Nonclinical Group (FANG) ELISA at Q^2^ Solutions (San Juan Capistrano, CA, US) [14,16,19] and were expressed as group geometric mean concentrations (GMCs) of ELISA units (EU)/mL with 95% confidence intervals (CIs). EBOV GP-specific neutralising antibody titres were measured using a pseudovirion neutralisation assay (psVNA) at Monogram (San Francisco, CA, US) [14,16] and expressed as group geometric mean titres (GMTs) of the half maximal inhibitory titre (IC_50_) with 95% CIs.

Ad26-specific neutralising antibodies were measured using an Ad26-specific virus neutralisation assay (Ad26 VNA) at baseline (Janssen Vaccines & Prevention B.V., Leiden, the Netherlands) and were expressed as group GMTs of 90% inhibitory concentration (IC_90_) with 95% CIs.

PBMCs, from a subset of additionally consented participants at sites capable of processing blood samples for PBMCs, were collected at the same time points specified above and were frozen for later determination of CD4+ and CD8+ T cells producing interferon (IFN)-γ, interleukin (IL)-2, and tumour necrosis factor (TNF)-α by intracellular cytokine staining (ICS). ICS data were expressed as the median percentage of each T cell subset (CD4+ or CD8+) producing at least one of the 3 investigated cytokines (IFN-γ, IL-2, TNF-α). IFN-γ responses to EBOV GP were evaluated using enzyme-linked immunospot (ELISpot) and were expressed as spot-forming units (SFU) per million PBMCs (reported as median reportable value). Both ICS and ELISpot were performed at the HIV Vaccine Trials Network (HVTN, Seattle, WA, USA) [11–13,20].

For sample positivity and responder definitions used in the FANG ELISA, psVNA, ELISpot, and ICS analyses, see **Text A in** S1 Supporting information.

### Statistical analyses

The study was originally designed as a prospective study with no formal hypothesis testing. Safety is presented descriptively for the full analysis set entailing all participants who were randomised and received at least one vaccine or placebo dose irrespective of protocol deviations. Immunogenicity is presented for the per protocol set, which includes all randomised and vaccinated participants who received both vaccinations, had at least one evaluable immunogenicity sample postvaccination, and had no major protocol deviations influencing the immune response. Binding antibodies were expressed as group GMCs of EU with 95% CIs at each time point, and responder rates (i.e., the percentage of each group with postvaccination concentrations >2.5-fold the lower limit of quantification [LLOQ; 36.11 EU/mL] in baseline seronegative individuals, or >2.5-fold the baseline value in initially seropositive participants). All values below the LLOQ were imputed with half the LLOQ value.

Spearman correlation coefficients were calculated for EBOV GP-specific binding antibody concentrations (FANG ELISA) versus psVNA titres 21 days post-MVA-BN-Filo, and for FANG ELISA and psVNA (21 days post-MVA-BN-Filo) versus Ad26 VNA (baseline) data. All statistical analyses were performed by the sponsor using SAS (version 9.2, SAS Institute, Cary, NC).

While no formal statistical testing was originally planned, statistical comparisons were performed post hoc for primary and secondary outcomes. The statistical significance was set at *p*-value < 0.05. No test multiplicity adjustments were performed.

## Results

### Participant enrolment and baseline demographics

**Fig 1:**
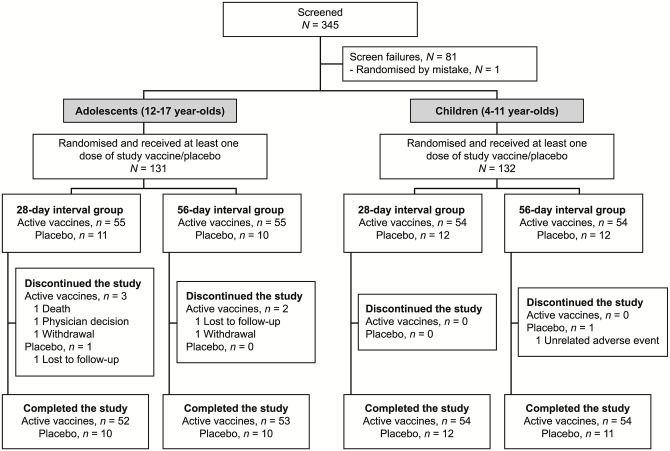
Study disposition of adolescents and children.

Enrolment of participants began on February 26, 2016, and the date of last participant last visit was November 28, 2018. Following screening, 263 eligible participants were randomised and received a study vaccine: 131 adolescents and 132 children. There was good study compliance, with 125/131 (95%) adolescents and 131/132 (99%) children completing the study (Fig 1) and no vaccination-related withdrawals. Study adolescents and children had a median age of 14.0 (range 11 to 17) and 7.0 (range 4 to 11) years, respectively. Other demographics across the dosing interval groups within the 2 age cohorts were similar (Table 1).

**Table 1:** Baseline characteristics of the participants; full analysis set*.

	Adolescents (12–17 years)				Children (4–11 years)			
	28-day interval group		56-day interval group		28-day interval group		56-day interval group	
Characteristic	Active vaccines	Placebo	Active vaccines	Placebo	Active vaccines	Placebo	Active vaccines	Placebo
*N* =	55	11	55	10	54	12	54	12
Sex—No. (%)								
Male	30 (55)	6 (55)	29 (53)	6 (60)	27 (50)	7 (58)	26 (48)	7 (58)
Female	25 (45)	5 (45)	26 (47)	4 (40)	27 (50)	5 (42)	28 (52)	5 (42)
Black—No. (%)	55 (100)	11 (100)	55 (100)	10 (100)	54 (100)	12 (100)	54 (100)	12 (100)
Age (years)								
Mean (SD)	14.4 (1.76)	14.5 (1.86)	14.1 (1.56)	14.2 (1.81)	7.6 (2.06)	7.1 (2.07)	7.8 (2.23)	7.3 (2.09)
Median (range)	14.0 (11–17)	14.0 (12–17)	14.0 (12–17)	14.0 (12–17)	7.0 (4–11)	6.5 (4–10)	8.0 (4–11)	7.0 (4–11)
Body mass index (kg/m^2^)								
Mean (SD)	19.36 (2.78)	18.17 (3.82)	18.95 (3.26)	18.03 (1.84)	-	-	-	-
Weight for age percentile								
Mean (SD)	-	-	-	-	29.32 (21.58)	38.69 (25.88)	28.51 (23.39)	37.91 (16.26)

*The numbers of participants who received at least 1 dose of the study vaccines or placebo.

*N*, all participants who received at least 1 dose of the study vaccines or placebo; SD, standard deviation.

### Safety

**Fig 2:**
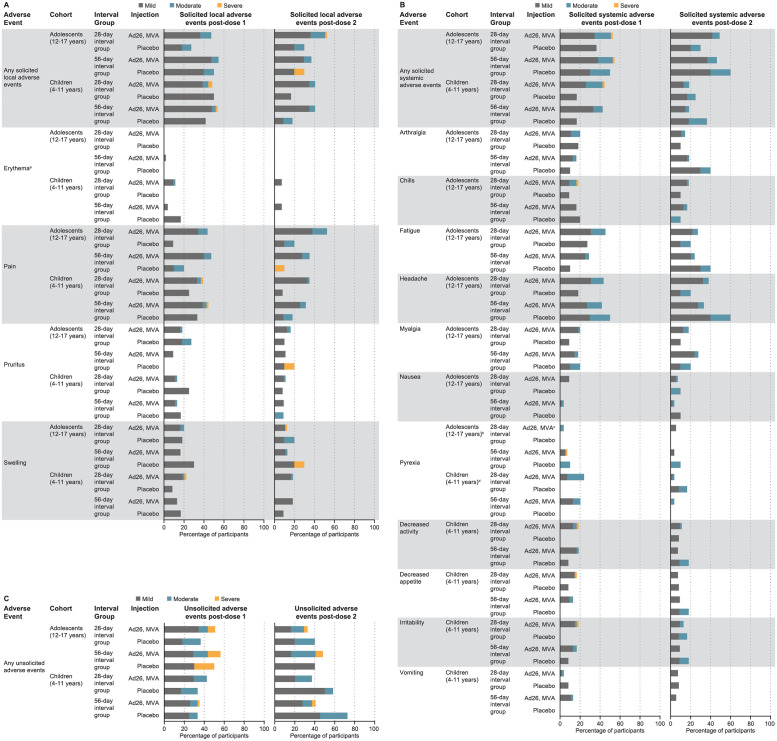
Solicited local AEs. (**A**) Solicited systemic AEs (**B**) and unsolicited AEs (**C**); full analysis set. Percentages reflect *n*/*N* where *n* is the number of participants with one or more AEs and *N* is the number of participants with available reactogenicity data after the given dose (solicited AEs) or the number of participants who received the given dose (unsolicited AEs). Only unsolicited AEs reported between the dose 1 vaccination and 28 days post-dose 1, and between dose 2 vaccination and 28 days post-dose 2 are included in this table. Different diaries were used in adolescents (12–17 years) and children (4–11 years) to collect solicited systemic AEs. Solicited systemic AEs collected in adolescents: arthralgia, chills, fatigue, headache, myalgia, nausea, and pyrexia; in children: pyrexia, decreased activity, decreased appetite, irritability, and vomiting. ^a^Per the DMID Toxicity Tables used in this study, erythema was graded based on the diameter data only. ^b^Pyrexia mild: 38.0–38.4 °C, moderate: 38.5–38.9 °C, severe: >38.9 °C. ^c^*N =* 54. ^d^Pyrexia mild: 38.0–38.4 °C, moderate: 38.5–40.0 °C, severe: >40.0 °C. Ad26: Ad26.ZEBOV at a dose of 5 × 10^10^ viral particles; MVA: MVA-BN-Filo at a dose of 1 × 10^8^ Inf.U. Ad26, Ad26.ZEBOV; AE, adverse event; DMID, Division of Microbiology and Infectious Diseases; Inf.U, infectious units; MVA, MVA-BN-Filo.

In general, both vaccines were well tolerated in adolescents and children, with solicited AEs that were mostly mild to moderate in severity and of short duration (Fig 2; **Tables A and E in** S1 Supporting information). The frequency of AEs appeared to be unaffected by the interval between doses.

One adolescent died from an unrelated typhoid fever and malaria infection with onset 52 days after MVA-BN-Filo vaccination. Two children had SAEs, a child burned in a domestic accident, and another child diagnosed with malaria, who recovered within 5 days; these were not considered by the investigator to be related to the study vaccine (**Table F in** S1 Supporting information).

#### Solicited AEs—Adolescents (12–17 years)

The frequency of any solicited local AE was numerically higher in vaccinees; reported in 56/110 (51%), 49/109 (45%), 8/21 (38%), and 6/20 (30%) adolescents following Ad26.ZEBOV, MVA-BN-Filo, first and second placebo injections, respectively: *p*-value = 0.34 for the events following Ad26.ZEBOV compared to first placebo injection, and *p*-value = 0.23 for the events following MVA-BN-Filo compared to second placebo injection (**Table C in** S1 Supporting information). Pain at the injection site was the most frequently reported solicited local AE in adolescents. Two severe solicited local AEs were reported in adolescents (both cases of swelling): one reported in a vaccinee following MVA-BN-Filo injection and one in a placebo recipient following second placebo injection.

Solicited systemic AEs were reported in 59/110 (54%), 52/109 (48%), 9/21 (43%), and 9/20 (45%) adolescents following Ad26.ZEBOV, MVA-BN-Filo, first and second placebo injections, respectively (Fig 2A and 2B; **Table A in** S1 Supporting information): *p*-value = 0.48 for the events following Ad26.ZEBOV compared to first placebo injection, and *p*-value = 1 for the events following MVA-BN-Filo compared to second placebo injection (**Table C in** S1 Supporting information). Headache was the most frequently reported solicited systemic AE in adolescents. Pyrexia was reported in identical frequencies (5%) in vaccinees (6/110 participants following Ad26.ZEBOV injection and 5/108 following MVA-BN-Filo injection) and placebo recipients (1/21 participants following first placebo injection and 1/20 following second placebo injection). Severe solicited systemic AEs were only reported by 2 adolescents (*n =* 1 fever; *n* = 1 chills), both following Ad26.ZEBOV injection.

#### Unsolicited AEs—Adolescents (12–17 years)

The number of adolescents reporting unsolicited AE was numerically higher following injection with Ad26.ZEBOV (59/110, 54%) than MVA-BN-Filo (44/109, 40%), first placebo (9/21, 43%), or second placebo (8/20, 40%) (Fig 2C; **Table A in** S1 Supporting information): *p*-value = 0.48 for the events following Ad26.ZEBOV compared to first placebo injection, and *p*-value = 1 for the events following MVA-BN-Filo compared to second placebo injection (**Table C in** S1 Supporting information). Severe unsolicited AEs were reported in 11/110 (10%), 6/109 (6%), and 2/21 (10%) adolescents following Ad26.ZEBOV, MVA-BN-Filo and first placebo injections, respectively, and in no adolescent following second placebo injection.

#### Solicited AEs—Children (4–11 years)

Solicited local AEs were reported in 55/108 (51%), 44/108 (41%), 11/24 (46%), and 4/23 (17%) children following Ad26.ZEBOV, MVA-BN-Filo, first placebo injection, and second placebo injection, respectively: *p*-value = 0.82 for the events following Ad26.ZEBOV compared to first placebo injection, and *p*-value = 0.05 for the events following MVA-BN-Filo compared to second placebo injection (**Table C in** S1 Supporting information). Compared to the adolescents, the rates were not significantly different following either Ebola vaccine (*p*-value = 1 following Ad26.ZEBOV; *p*-value = 0.6 following MVA-BN-Filo; **Table B in** S1 Supporting information). Pain at the injection site was the most frequently reported solicited local AE in children. Severe solicited local AEs were reported in 3 children; all of these occurred following Ad26.ZEBOV vaccination (severe pain [*n =* 2]; swelling [*n* = 1]).

The number of children reporting any solicited systemic AE was significantly higher following Ad26.ZEBOV (47/108, 44%) compared to MVA-BN-Filo (20/108, 19%, *p*-value < 0.001) (**Table D in** S1 Supporting information). The number of children reporting any solicited systemic AE was also numerically higher following Ad26.ZEBOV than first placebo (4/24, 17%) or second placebo (7/23, 30%) (Fig 2A and 2B; **Table A in** S1 Supporting information). The number of children reporting any solicited systemic AE was also significantly (*p*-value = 0.02) higher following Ad26.ZEBOV than first placebo (4/24, 17%). The number of solicited AEs following MVA-BN-Filo (20/108, 19%) was not significantly different (*p-*value = 0.25) compared to second placebo (7/23, 30%) (**Table C in** S1 Supporting information). Compared to the adolescents, the rates were not significantly different following Ad26.ZEBOV (*p*-value = 0.14) but were different following MVA-BN-Filo (*p*-value < 0.001; **Table B in** S1 Supporting information). Pyrexia was the most frequently reported solicited systemic AE in children following Ad26.ZEBOV (24/108, 22%) and was significantly higher than the frequency reported following MVA-BN-Filo (22% versus 4%, *p*-value < 0.001) (**Table D in** S1 Supporting information). Pyrexia was not reported in any children following the first placebo. Pyrexia was reported in 4/108 (4%) and 2/23 (9%) children following MVA-BN-Filo and second placebo injections (*p*-value = 0.28; **Table C in** S1 Supporting information), respectively. Irritability was the most frequently reported solicited systemic AE in children following MVA-BN-Filo (12/108, 11%) and second placebo (4/23, 17%). Except for one case of severely decreased activity and appetite plus irritability, all other solicited systemic AEs were mild or moderate.

#### Unsolicited AEs—Children (4–11 years)

The frequency of children reporting unsolicited AEs was similar following Ad26.ZEBOV (42/108, 39%), MVA-BN-Filo (42/108, 39%), and first placebo injection (8/24, 33%) but was higher following the second placebo injection (15/23, 65%) (Fig 2C; **Table A in** S1 Supporting information): *p*-value = 0.65 for the events following Ad26.ZEBOV compared to first placebo injection, and *p*-value = 0.04 for the events following MVA-BN-Filo compared to second placebo injection (**Table C in** S1 Supporting information). Compared to the adolescents, the rates were significantly different following Ad26.ZEBOV (*p*-value = 0.03) but not after MVA-BN-Filo (*p*-value = 0.89; **Table B in** S1 Supporting information). One severe unsolicited AE was reported following Ad26.ZEBOV injection (neutropaenia), and two were reported following MVA-BN-Filo injection (increased transaminases [*n =* 1]; gastroenteritis [*n* = 1]).

### Immunogenicity

#### Binding antibody responses against EBOV GP

**Fig 3:**
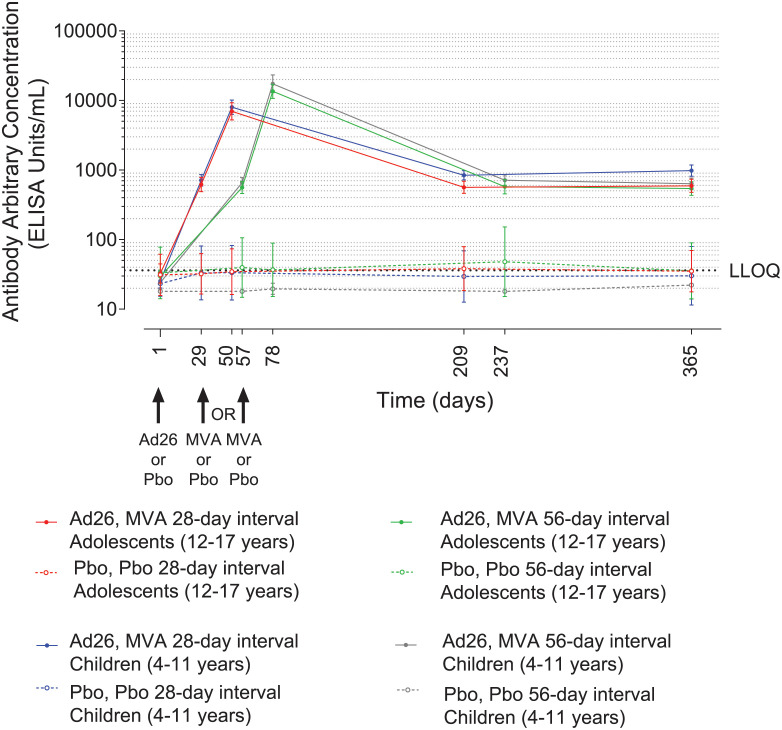
GMCs of EBOV-specific binding antibodies (FANG ELISA, 95% CI) in adolescents and children. Participants administered with Ad26.ZEBOV or placebo on day 1 and MVA-BN-Filo or placebo 28 or 56 days later as indicated. Responses are expressed as GMCs (EU/mL, 95% CI). Responses in placebo groups are shown as open symbols. Grey dotted line represents the LLOQ. The points (symbols) denote GMCs, and error bars denote 95% CIs. Ad26, Ad26.ZEBOV; CI, confidence interval; EBOV, Ebola virus; EU, ELISA units; FANG ELISA, Filovirus Animal Nonclinical Group enzyme-linked immunosorbent assay; GMC, geometric mean concentration; LLOQ, lower limit of quantification; MVA, MVA-BN-Filo; Pbo, placebo.

All vaccinees displayed robust EBOV GP-specific binding antibody responses. In placebo recipients, EBOV GP-specific binding antibody levels were either low or not quantifiable at all assessed time points (Fig 3; **Table G in** S1 Supporting information). No relevant differences in EBOV GP-specific binding antibody responses in either adolescents or children were observed among countries (**Table J in** S1 Supporting information).

#### Binding antibody responses against EBOV GP—Adolescents (12–17 years)

After Ad26.ZEBOV and prior to MVA-BN-Filo injection, 50/54 (93%) and 50/53 (94%) vaccinees showed an EBOV GP-specific binding response at day 29 and at day 57 in the 28-day and 56-day interval groups, respectively. At 21 days post-MVA-BN-Filo, 100% of vaccinees responded, with GMCs of 6,993 EU/mL (95% CI, 5,256 to 9,303) and 13,532 EU/mL (95% CI, 10,732 to 17,061) in the 28-day (*n =* 53) and 56-day (*n* = 53) interval groups, respectively **(**Fig 3; **Table G in** S1 Supporting information) (GMC ratio [95% CI] = 0.5 [0.4 to 0.7], *p*-value < 0.001; **Table H in** S1 Supporting information). At 6 months post-MVA-BN-Filo, responses were observed in 38/41 (93%) vaccinees in both the 28-day (GMC, 565 EU/mL [95% CI, 463 to 689]) and the 56-day (GMC, 577 EU/mL [95% CI, 454 to 734]) interval groups. Responses persisted up to day 365 in 46/50 (92%; GMC, 593 EU/mL [95% CI, 477 to 738]) and in 47/52 (90%; GMC, 541 EU/mL [95% CI, 433 to 678]) vaccinees in the 28-day and 56-day interval groups, respectively (Fig 3; **Table G in** S1 Supporting information).

#### Binding antibody responses against EBOV GP—Children (4–11 years)

After Ad26.ZEBOV and prior to MVA-BN-Filo vaccination, 51/53 (96%) and 51/52 (98%) vaccinees showed a response at day 29 and at day 57 in the 28-day and 56-day interval groups, respectively. At 21 days post-MVA-BN-Filo, 100% of vaccinees responded, with GMCs of 8,007 EU/mL (95% CI, 6,321 to 10,142) and 17,388 EU/mL (95% CI, 12,973 to 23,306) in the 28-day (*n =* 53) and 56-day (*n* = 51) interval groups, respectively (Fig 3; **Table G in** S1 Supporting information) (GMC ratio [95% CI] = 0.5 [0.3 to 0.7], *p*-value < 0.001; **Table H in** S1 Supporting information). Compared to the adolescents, the GMCs of binding antibodies at 21 days post-dose 2 were not significantly different for the children (GMC ratio [95% CI] = 1.1 [0.8 to 1.7], *p*-value = 0.47 for 28-day interval; GMC ratio [95% CI] = 1.3 [0.9 to 1.9], *p*-value = 0.18 for 56-day interval; **Table I in** S1 Supporting information). At 6 months post-MVA-BN-Filo, responses were observed in 51/52 (98%) vaccinees in both the 28-day interval group (GMC, 841 EU/mL [95% CI, 721 to 980]) and the 56-day interval group (GMC, 715 EU/mL [95% CI, 602 to 851]). Responses persisted up to day 365 in 51/53 (96%; 981 EU/mL [95% CI, 814 to 1,183]) and 51/52 (98%; 637 EU/mL [95% CI, 529 to 767]) vaccinees in the 28-day and 56-day interval groups, respectively (Fig 3; **Table G in** S1 Supporting information).

#### Neutralising antibody responses against EBOV GP

Most (96% to 100%) vaccinees displayed robust EBOV GP-specific neutralising antibody responses; EBOV GP-specific neutralising antibodies were not detected in any placebo recipients (**Fig A in** S1 Supporting information; **Table K in** S1 Supporting information). No relevant differences in EBOV GP-specific neutralising antibody responses in either adolescents or children were observed between countries (**Table L in** S1 Supporting information).

#### Neutralising antibody responses against EBOV GP—Adolescents (12–17 years)

At 21 days post-MVA-BN-Filo, responses were observed in 51/53 (96%) vaccinees in the 28-day interval group and all 53/53 (100%) vaccinees in the 56-day interval group, with GMTs of 1,879 IC_50_ titre (95% CI, 1,424 to 2,478) and 6,403 IC_50_ titre (95% CI, 5,289 to 7,751), respectively (**Fig A and Table K in** S1 Supporting information). At day 365, responses persisted in 27/50 (54%; GMT, 251 IC_50_ titre [95% CI, 191 to 331]) vaccinees in the 28-day interval group and 23/52 (44%; GMT, 218 IC_50_ titre [95% CI, 174 to 273]) vaccinees in the 56-day interval group.

#### Neutralising antibody responses against EBOV GP—Children (4–11 years)

At 21 days post-MVA-BN-Filo, responses were observed in all 53/53 (100%) vaccinees in the 28-day interval group and in 51/53 (96%) vaccinees in the 56-day interval group, with GMTs of 2,506 IC_50_ titre (95% CI, 1,903 to 3,300) and 8,352 IC_50_ titre (95% CI, 6,025 to 11,577), respectively (**Fig A and Table K in** S1 Supporting information). At day 365, responses persisted in 44/52 (85%; GMT, 447 IC_50_ titre [95% CI, 371 to 539]) and 32/54 (59%; GMT, 275 IC_50_ titre [95% CI, 224 to 338]) vaccinees in the 28- and 56-day interval groups, respectively.

A strong correlation between EBOV GP-specific binding antibody concentrations and neutralising antibody titres was observed across both age cohorts and vaccine dosing interval groups at 21 days post-MVA-BN-Filo (Spearman correlation coefficient: 0.660 to 0.829) (**Fig B in** S1 Supporting information) and at day 365 (Spearman correlation coefficient: 0.537 to 0.841) (**Fig B in** S1 Supporting information).

#### Ad26 neutralising antibodies

Prior to vaccination, most participants were positive for preexisting Ad26 neutralising antibodies (Ad26 VNA) (**Table M in** S1 Supporting information). In adolescents, 111/127 (87%) participants were positive (95/107 vaccinees; 16/20 placebo recipients), and GMT was 112 IC_90_ titre (95% CI, 86 to 145). In children, 92/130 (71%) participants were positive (76/107 vaccinees; 16/23 placebo recipients), and GMT was 86 IC_90_ titre (95% CI, 61 to 121). No correlations were observed between baseline Ad26 VNA titres and EBOV GP-specific binding antibody concentrations (FANG ELISA) at 21 days post-MVA-BN-Filo (Spearman correlation coefficients in adolescent vaccinees: −0.20 in the 28-day and −0.06 in the 56-day interval groups; in children vaccinees: 0.10 in the 28-day and 0.01 in the 56-day interval groups) (**Fig C in** S1 Supporting information). Similarly, no correlations were observed between preexisting Ad26-specific VNA titres and EBOV GP-specific neutralising antibody titres at 21 days post-MVA-BN-Filo (Spearman correlation coefficients in adolescent vaccinees: −0.18 in the 28-day and −0.09 in the 56-day interval groups; in children vaccinees: 0.05 in the 28-day and 0.09 in the 56-day interval groups) (**Fig C in Supporting information**).

#### Cellular immune responses against EBOV GP

No apparent differences in cellular immune responses were observed between adolescents and children, although the sample sizes were small. CD4+ and CD8+ data (**Figs D and E in** S1 Supporting information; **Tables N and O in** S1 Supporting information) and IFN-γ ELISpot data (**Fig F in** S1 Supporting information; **Table P in** S1 Supporting information) are described in **Text B in** S1 Supporting information.

## Discussion

We have previously reported that in adults, the heterologous 2-dose Ad26.ZEBOV, MVA-BN-Filo vaccine regimen against EVD is safe, well tolerated, and immunogenic in several Phase I and II trials [11–16]. The present report confirms those observations in 12- to 17-year-old and 4- to 11-year-old participants. The vaccine regimen was well tolerated in these younger age groups, with no vaccine-related SAEs or vaccine-related dropouts. The majority of solicited local AEs and systemic AEs were mild or moderate, with few reports of severe AEs. The frequency of pyrexia after vaccine or placebo was higher in children than in adolescents, in line with a previously reported study [21].

Robust EBOV GP-specific binding and neutralising antibody levels were observed in these younger age groups, consistent with those reported in adults [11–16], with higher responses observed with a longer time interval between the 2 doses (56 versus 28 days). Although not statistically significant, greater responses in the younger age cohort were also detected, in line with our previous observations in an Ad26.ZEBOV, MVA-BN-Filo study in Sierra Leone [21,22]. Ad26 preexisting immunity was observed in the majority of participants without having an impact on the EBOV GP-specific antibody responses postvaccination, which was consistent with our previous reports for adults and children [16,21,22]. Similar antibody responder rates and levels at all time points were found across the 4 different countries. In most participants, antibodies persisted at least up to 1 year after the first vaccination; this is an encouraging result for the long-term response [23]. The relative contribution of cellular versus humoral immune responses to protection from EVD following vaccination is under debate [24–28], but both cellular and humoral immune responses were elicited by Ad26.ZEBOV, MVA-BN-Filo vaccine regimens in the current study, and T cell–mediated responses in this study are consistent with the results of 2 previous studies conducted in African adults [12,13]. Although a mechanistic correlate of protection is currently not known, studies performed in an established animal EBOV challenge model demonstrate that binding and neutralising antibodies correlate strongly with protection [29,30]. It is therefore reassuring to observe that the 2-dose heterologous regimen elicits robust humoral responses in younger age groups, and it is particularly important that this was established in these relevant populations from 4 African countries.

Although the proportion of children with EVD is usually lower compared with adults during outbreaks [31,32], infected children are more severely affected by EVD [33]. During the 2014 to 2016 EVD outbreak in West Africa, the mortality among children and adolescents younger than 15 years of age was 73% compared with the overall case fatality rate of 71% [32]. Children demonstrated shorter incubation periods and shorter intervals between symptom onset and hospitalisation or death [32]. Therefore, vaccination of children would be very relevant in an overall prophylactic vaccination strategy in countries vulnerable to future EVD outbreaks.

Study limitations include that due to PBMC shipment losses, assay failure, and/or low sample viability, fewer children and adolescents were analysed for cellular immune responses than originally planned. Also, the sample sizes were particularly small for analysis of ICS results. Another limitation is that the follow-up period was limited to 365 days, and so it was not possible to determine whether immune responses persisted beyond this time period, although modelling results are encouraging [23]. This limitation could not be avoided as a follow-up period had to be determined prior to study start. No formal statistical testing of safety or immune response data was originally planned for this study. Although post hoc statistical comparisons were performed, no direct conclusions on these comparisons between regimens or between adolescents (12 to 17 years) and children (4 to 11 years) can be made; a clinically meaningful difference in terms of binding antibody levels is not known.

In conclusion, the data from this study suggest that the same heterologous vaccine regimen, dosage, and schedule used in adults can be safely administered to children aged ≥4 years, with acceptable reactogenicity and robust immune responses. These results have contributed to the inclusion of the paediatric population in the indication of the 2-dose heterologous regimen [8–10].

## Supporting information

S1 Study Protocol (PDF)

S1 CONSORT Checklist (DOCX)

S1 Supporting information Text A. Supplementary methodology. Text B. Supplementary results. Text C. EBL2002 study group (in addition to authors). Table A. Solicited local adverse events, solicited systemic adverse events, and unsolicited adverse events; full analysis set. Table B. Comparison (children [4–11 years] versus adolescents [12–17 years]) of solicited and unsolicited adverse events after each vaccination dose based on Fisher’s exact test; full analysis set. Table C. Comparison (vaccinees versus placebo recipients) of solicited and unsolicited adverse events after each vaccination dose based on Fisher’s exact test; full analysis set. Table D. Comparison (Ad26.ZEBOV versus MVA-BN-Filo) of solicited and unsolicited adverse events in the full analysis set based on Fisher’s exact test. (Study VAC52150EBL2002; full analysis set). Table E. Duration of solicited local adverse events and solicited systemic adverse events; full analysis set. Table F. Serious adverse events, full analysis set. Table G. EBOV GP-specific binding antibody responses (ELISA units/mL): geometric mean concentrations and responder rates; per protocol set. Table H. Comparison of EBOV-GP-specific binding antibodies in adolescents [12–17 years] and children [4–11 years] in the Ebola vaccine groups; per protocol set. Table I. Comparison of EBOV-GP-specific binding antibodies in children [4–11 years] versus adolescents [12–17 years] in the Ebola vaccine groups; per protocol set. Table J. EBOV GP-specific binding antibody responses (ELISA units/mL): geometric mean concentrations and responder rates by country; per protocol analysis set. Table K. EBOV GP-specific neutralising antibody responses (psVNA; IC_50_ titre); per protocol analysis set. Table L. EBOV GP-specific neutralising antibody responses (psVNA; IC_50_ titre) by country; per protocol analysis set. Table M. Ad26 neutralising antibodies (Ad26 VNA; IC_90_ titre); per protocol analysis set. Table N. EBOV GP-specific CD4+ T cell cytokine responses (ICS, % of subset); per protocol analysis set. Table O. EBOV GP-specific CD8+ T cell cytokine responses (ICS, % of subset); per protocol analysis set. Table P. EBOV GP-specific IFN-γ producing T cell responses (IFN-γ ELISpot, SFU/106 PBMC); per protocol analysis set. Fig A. EBOV GP-specific neutralising antibody responses—Regimen plot (psVNA; IC_50_ titre); per protocol analysis set. Fig B. Spearman correlation between EBOV GP-specific binding and neutralising antibody responses 21 days post-MVA-BN-Filo; per protocol analysis set. (A) 21 days post-dose 2. (B) 364 days post-dose 1. Fig C. Correlations between Ad26-specific neutralising antibody titres at baseline and EBOV GP-specific binding and neutralising antibodies 21 days post-dose 2. (A) Anti-EBOV GP IgG ELISA at 21 days post-dose 2 by Ad26 neutralisation assay at baseline. (B) EBOV GP neutralisation assay at 21 days post-dose 2 by Ad26 neutralisation assay at baseline. Fig D. CD4+ and CD8+ T cell responses in adolescents (ICS). Fig E. CD4+ and CD8+ T cell responses in children (ICS). Fig F. EBOV GP-specific IFN-γ producing T cell responses (ELISpot). (A) Adolescents (12–17 years). (B) Children (4–11 years).
(DOCX)

## Abbreviations

AE: adverse event
CI: confidence interval
EBOV: Ebola virus
ELISpot: enzyme-linked immunospot
EU: ELISA units
EVD: Ebola virus disease
FANG: Filovirus Animal Nonclinical Group
GMC: geometric mean concentration
GMT: geometric mean titre
GP: glycoprotein
IC_50_: half maximal inhibitory titre
IC_90_: 90% inhibitory concentration
ICS: intracellular cytokine staining
IFN: interferon
IL: interleukin
Inf.U: infectious units
IWRS: interactive web response system
LLOQ: lower limit of quantification
PBMC: peripheral blood mononuclear cell
psVNA: pseudovirion neutralisation assay
SAE: serious adverse event
SFU: spot-forming units
TNF: tumour necrosis factor
