# Supplementary material for: Safety and immunogenicity of 2-dose heterologous Ad26.ZEBOV, MVA-BN-Filo Ebola vaccination in children and adolescents in Africa: A randomised, placebo-controlled, multicentre Phase II clinical trial
Source: PLoS Med. 2022 Jan 11;19(1):e1003865. doi: 10.1371/journal.pmed.1003865 (PMC8752100; doi:10.1371/journal.pmed.1003865)
Supplement: S1 Study Protocol — (PDF) [file pmed.1003865.s001.pdf]

Janssen Vaccines & Prevention B.V.*

Clinical Protocol

A Randomized, Observer-blind, Placebo-controlled, Phase 2 Study to Evaluate the Safety, Tolerability and Immunogenicity of Different Prime-boost Regimens of the Candidate Prophylactic Vaccines for Ebola Ad26.ZEBOV and MVA-BN-Filo in Healthy Adults, Including Elderly Subjects, HIV-infected Subjects, and Healthy Children in Two Age Strata in Africa

---

Protocol VAC52150EBL2002; Phase 2
AMENDMENT 3

Innovative Medicines Initiative-2
EBOVAC2 Consortium Partners
(London School of Hygiene and Tropical Medicine,
Institut National de la Santé et de la Recherche Médicale,
University of Oxford, Le Centre MURAZ and Janssen Vaccines & Prevention B.V.)

VAC52150 (Ad26.ZEBOV/MVA-BN-Filo [MVA-mBN226B])

*Janssen Vaccines & Prevention B.V. (formerly known as Crucell Holland B.V.) is a Janssen pharmaceutical company of Johnson & Johnson and is hereafter referred to as the sponsor of the study.

Status: Approved
Date: 4 May 2017
Prepared by: Janssen Vaccines & Prevention B.V.
EDMS number: EDMS-ERI-97109067, 12.0

GCP Compliance: This study will be conducted in compliance with Good Clinical Practice, and applicable regulatory requirements.

Confidentiality Statement
The information in this document contains trade secrets and commercial information that are privileged or confidential and may not be disclosed unless such disclosure is required by applicable law or regulations. In any event, persons to whom the information is disclosed must be informed that the information is privileged or confidential and may not be further disclosed by them. These restrictions on disclosure will apply equally to all future information supplied to you that is indicated as privileged or confidential.

TABLE OF CONTENTS

TABLE OF CONTENTS 2

LIST OF ATTACHMENTS 5

LIST OF IN-TEXT TABLES AND FIGURES 5

PROTOCOL AMENDMENTS 6

SYNOPSIS 20

TIME AND EVENTS SCHEDULE 27
Cohort 1 (Groups 1-3) and Cohorts 2a and 2b (Groups 1-2) 27
Pediatric Cohort 3 (Groups 1-2) 31

ADDITIONAL TIME AND EVENTS SCHEDULE 34
Subjects Who Do Not Receive a Boost Vaccination Because of a Study Pause 34

COHORT 1 SUBSTUDY TIME AND EVENTS SCHEDULE 37
Subjects at Selected Sites Receiving a Third Vaccination (Cohort 1, Groups 1-2) 37

ABBREVIATIONS 39

DEFINITIONS OF TERMS 40

1. INTRODUCTION 41
1.1. Background 41
1.1.1. Nonclinical Studies 42
1.1.2. Clinical Studies 43
1.2. Benefit/Risk Section 48
1.2.1. Known Benefits 48
1.2.2. Potential Benefits 48
1.2.3. Known Risks 48
1.2.4. Potential Risks 48
1.2.5. Overall Benefit/Risk Assessment 50
1.3. Overall Rationale for the Study 51

2. OBJECTIVES AND HYPOTHESIS 53
2.1. Objectives 53
2.2. Hypothesis 54

3. STUDY DESIGN AND RATIONALE 54
3.1. Overview of Study Design 54
3.2. Study Design Rationale 59

4. SUBJECT POPULATION 61
4.1. Inclusion Criteria 62
4.1.1. Inclusion Criteria for Healthy Adult and Elderly Subjects 62
4.1.2. Additional Inclusion Criteria for HIV-infected Subjects 64
4.1.3. Inclusion Criteria for Pediatric Subjects 65
4.2. Exclusion Criteria 67
4.2.1. Exclusion Criteria for Healthy Adult and Elderly Subjects 67
4.2.2. Additional Exclusion Criteria for HIV-infected Subjects 69
4.2.3. Exclusion Criteria for Pediatric Subjects 69
4.3. Prohibitions and Restrictions 71

5. TREATMENT ALLOCATION AND BLINDING 72

6. DOSAGE AND ADMINISTRATION 74
6.1. General Instructions and Procedures 74
6.2. Criteria for Postponement of Vaccination 76
6.3. Contraindications to Boost and Third Vaccinations 76

7. TREATMENT COMPLIANCE 77

8. PRESTUDY AND CONCOMITANT THERAPY 77

9. STUDY EVALUATIONS 78
9.1. Study Procedures 78
9.1.1. Overview 78
9.1.2. Screening Phase 80
9.1.3. Vaccination Phase (Prime and Boost) 81
9.1.4. Post-vaccination Phase 82
9.1.5. Long-term Follow-up 83
9.1.6. Procedures in Case of a Study Pause 83
9.1.7. Cohort 1 Substudy (Third Vaccination) 86
9.1.8. VAC52150 Vaccine Development Roll-over Study 87
9.2. Endpoints 87
9.3. Safety Evaluations 88
9.3.1. Safety Assessments 88
9.3.2. Pausing Rules 92
9.4. Immunogenicity Evaluations 93
9.5. Vaccine-induced Seropositivity 95
9.6. Sample Collection and Handling 95

10. SUBJECT COMPLETION/DISCONTINUATION OF STUDY VACCINE/WITHDRAWAL FROM THE STUDY 95
10.1. Completion 95
10.2. Discontinuation of Study Vaccine/Withdrawal From the Study 95
10.3. Withdrawal From the Use of Research Samples 97

11. STATISTICAL METHODS 97
11.1. Endpoints 98
11.1.1. Primary Endpoints 98
11.1.2. Secondary Endpoint 98
11.1.3. Exploratory Endpoints 99
11.2. Sample Size Determination 99
11.3. Analysis Sets 100
11.4. Subject Information 101
11.5. Safety Analyses 101
11.6. Immunogenicity Analyses 102
11.7. Interim Analyses 102
11.8. Independent Data Monitoring Committee 102
11.9. Trial Management Team and Clinical Steering Committee 103

12. ADVERSE EVENT REPORTING 103
12.1. Definitions 103
12.1.1. Adverse Event Definitions and Classifications 103
12.1.2. Attribution Definitions 105
12.1.3. Severity Criteria 106
12.2. Special Reporting Situations 106
12.3. Procedures 107
12.3.1. All Adverse Events 107
12.3.2. Serious Adverse Events 108
12.3.3. Immediate Reportable Events 109
12.3.4. Pregnancy 109
12.4. Contacting Sponsor Regarding Safety 110

13. PRODUCT QUALITY COMPLAINT HANDLING 110
13.1. Procedures 110
13.2. Contacting Sponsor Regarding Product Quality 110

14. STUDY VACCINE INFORMATION 111
14.1. Description of Study Vaccines 111
14.2. Packaging and Labeling 111
14.3. Preparation, Handling, and Storage 112
14.4. Study Vaccine Accountability 112

15. STUDY-SPECIFIC MATERIALS 113

16. ETHICAL ASPECTS 113
16.1. Study-specific Design Considerations 113
16.2. Regulatory Ethics Compliance 114
16.2.1. Investigator Responsibilities 114
16.2.2. Independent Ethics Committee or Institutional Review Board 115
16.2.3. Informed Consent and Assent Form 116
16.2.4. Privacy of Personal Data 117
16.2.5. Long-term Retention of Samples for Additional Future Research 118
16.2.6. Country Selection 118

17. ADMINISTRATIVE REQUIREMENTS 118
17.1. Protocol Amendments 118
17.2. Regulatory Documentation 119
17.2.1. Regulatory Approval/Notification 119
17.2.2. Required Prestudy Documentation 119
17.3. Subject Identification, Enrollment, and Screening Logs 120
17.4. Source Documentation 120
17.5. Case Report Form Completion 121
17.6. Data Quality Assurance/Quality Control 121
17.7. Record Retention 121
17.8. Monitoring 122
17.9. Study Completion/Termination 123
17.9.1. Study Completion/End of Study 123
17.9.2. Study Termination 123
17.10. On-site Audits 123
17.11. Use of Information and Publication 124

REFERENCES 126

ATTACHMENTS 128

INVESTIGATOR AGREEMENT 139

LAST PAGE 139

LIST OF ATTACHMENTS

Attachment 1: Test of Understanding (TOU) 128
Attachment 2: Toxicity Tables for Use in Trials Enrolling Healthy Adults 129
Attachment 3: Toxicity Tables for Use in Trials Enrolling Children Greater Than 3 Months of Age 134
Attachment 4: Hemoglobin Cut-off Values 138

LIST OF IN-TEXT TABLES AND FIGURES

TABLES

Table 1: Study Vaccination Schedules Groups 1-3 74
Table 2: Cohort 1 Substudy Vaccination Schedule (Groups 1-2, Selected Sites) 74
Table 3: Visit Windows 79
Table 4: Visit Windows for Subjects Who Elect to Receive a Late Boost Vaccination 84
Table 5: Visit Windows for Subjects Who are Not Willing or Not Allowed to Receive the Late Boost Vaccination 85
Table 6: Visit Windows 86
Table 7: Whole Blood Volumes for Immunogenicity Assessments 94
Table 8: Summary of Immunologic Assessments 94
Table 9: Probability of Observing at Least One Adverse Event Given a True Adverse Event Incidence 100
Table 10: Magnitude of Pairwise Difference to be Detected Between Vaccine Regimens for a Given Sample Size 100

FIGURES

Figure 1: Schematic Overview of the Study 56

PROTOCOL AMENDMENTS

Protocol Version	Issue Date
Original Protocol	30 March 2015
Amendment 1	26 June 2015
Amendment 2	9 September 2016
Amendment 3	4 May 2017

Amendments below are listed beginning with the most recent amendment.

Amendment_3 (this document)

The overall reason for the amendment: This amendment is developed in response to emerging clinical data and changes to the global clinical development plan. Immunogenicity data from a Phase 1 study of the monovalent vaccine program (VAC52150EBL1002) demonstrated stability of the binding antibody response out to 1 year following prime-boost regimens with Ad26.ZEBOV/MVA-BN-Filo (or the reverse). A third vaccination using Ad26.ZEBOV at $5x10^{10}$ vp was given at the 1-year time point leading to a marked and rapid increase in the binding antibody responses within 7 days (at least 10-fold). The marked and rapid rise was generally independent of the antecedent prime-boost regimen. The profile of the antibody strongly suggests that robust anamnestic responses can be induced after re-exposure to an EBOV antigen, in this case mimicked by a third vaccination. This amendment proposes administration of a third vaccination using Ad26.ZEBOV at least 1 year post prime at selected sites in approximately 90 healthy adults in Cohort 1 (Groups 1 and 2) in order to extend the findings to an African population and confirm the immunogenicity profile from the Phase 1 study.
This amendment also proposes to eliminate Cohort 4 (aged 1-3 years, inclusive) entirely in this protocol. Safety and immunogenicity data for this age group will be collected in 2 other studies within the program (Phase 2 and Phase 3 studies with approximately 1,400 and 976 children aged ≥1 year, respectively) reducing the need to collect these data within this study and allowing it to focus on healthy adult and elderly subjects, HIV-infected subjects and healthy children aged 4-17 years (all ages inclusive). The sponsor also proposes to remove the following immunogenicity assessments: pre-boost PBMC isolation in children from Cohort 3 and the 7-day post-boost immunogenicity assessments in children from Cohorts 2b and 3.
Furthermore, the enrollment of subjects from VAC52150EBL2002 to the VAC52150EBL4001 roll-over study is limited to female subjects who became pregnant with estimated conception within 28 days after vaccination with MVA-BN-Filo (or placebo) or within 3 months after vaccination with Ad26.ZEBOV (or placebo) and children born to vaccinated female subjects who became pregnant with estimated conception within 28 days after vaccination with MVA-BN-Filo (or placebo) or within 3 months after vaccination with Ad26.ZEBOV (or placebo), unless local regulations have additional requirements for follow-up. The original development plan (at the time of the ongoing Ebola epidemic in Africa) was an accelerated plan with the anticipation of conducting Phase 3 efficacy studies (with limited safety data collection) shortly after Phase 1 and in parallel with Phase 2. The sponsor designed the VAC52150EBL4001 study for the extended follow-up of serious adverse events (SAEs) to enhance the ability for signal detection of rare events. Since there is no longer an ongoing Ebola epidemic, it is currently not possible to conduct a parallel Phase 3 efficacy study as part of an accelerated development plan. The ongoing Phase 2 and 3 studies will continue to have long-term follow-up in a placebo-controlled manner until agreement to unblind has been achieved. Placebo-controlled blinded data during long-term follow-up will provide high quality data on long-term safety and will supplement the open-label registry data.

The changes made to the clinical protocol VAC52150EBL2002 Amendment 2 (dated 9 September 2016) are listed below, including the rationale of each change and a list of all applicable sections.

Rationale: A third vaccination with Ad26.ZEBOV or placebo will be administered to approximately 90 healthy adults in Cohort 1 (Groups 1 and 2) at selected sites at least 1 year post prime in order to confirm and extend the findings from a Phase 1 study. Information was added to clarify the procedures that need to be followed for these subjects.

SYNOPSIS

Time and Events Schedule
Cohort 1 Substudy Time and Events Schedule
1.2.4 Potential Risks
1.2.5 Overall Benefit/Risk Assessment
1.3 Overall Rationale for the Study
2.1 Objectives
3 STUDY DESIGN AND RATIONALE
4.2.1 Exclusion Criteria for Healthy Adult and Elderly Subjects
4.3 Prohibitions and Restrictions
5 TREATMENT ALLOCATION AND BLINDING
6 DOSAGE AND ADMINISTRATION
6.1 General Instructions and Procedures
6.2 Criteria for Postponement of Vaccination
6.3 Contraindications to Boost and Third Vaccination
8 PRESTUDY AND CONCOMITANT THERAPY
9.1 Study Procedures
9.1.5 Long-term Follow-up
9.1.7 Cohort 1 Substudy (Third Vaccination)
9.3.1 Safety Assessments
9.3.2 Pausing Rules
9.4 Immunogenicity Evaluations
10.1 Completion
11 STATISTICAL METHODS
11.1 Endpoints
11.3 Analysis Sets
11.5 Safety Analyses
11.6 Immunogenicity Analyses
11.7 Interim Analyses
12.3.1 All Adverse Events

Rationale: Cohort 4 (aged 1-3 years, inclusive) was eliminated. Safety and immunogenicity data for this age group will be collected in 2 other studies within the program (Phase 2 and Phase 3 studies with approximately 1,400 and 976 children aged ≥1 year, respectively), reducing the need to collect these data within this study and allowing it to focus on healthy adult and elderly subjects, HIV-infected subjects and healthy children aged 4-17 years (all ages inclusive). Consequently, the sample size was updated.

SYNOPSIS
Time and Events Schedule
1.3 Overall Rationale for the Study
2.1 Objectives
3.1 Overview of Study Design
4.1.3 Inclusion Criteria for Pediatric Subjects
4.2.3 Exclusion Criteria for Pediatric Subjects
5 TREATMENT ALLOCATION AND BLINDING
6 DOSAGE AND ADMINISTRATION
9.1.1 Overview
9.1.2 Screening Phase

9.1.3 Vaccination Phase (Prime and Boost)
9.3 Safety Evaluations
9.4 Immunogenicity Evaluations
11.2 Sample Size Determination
11.5 Safety Analyses
11.7 Interim Analyses
11.8 Independent Data Monitoring Committee
16.1 Study-specific Design Considerations

Rationale: The title of the study was updated to delete the third age stratum (Cohort 4) and to include the Cohort 1 substudy.

TITLE PAGE
SYNOPSIS

Rationale: The original development plan (at the time of the ongoing Ebola epidemic in Africa) was an accelerated plan with the anticipation of conducting Phase 3 efficacy studies (with limited safety data collection) shortly after Phase 1 and in parallel with Phase 2. The sponsor designed the VAC52150EBL4001 study for the extended follow-up of SAEs to enhance the ability for signal detection of rare events. Since there is no longer an ongoing Ebola epidemic, it is currently not possible to conduct a parallel Phase 3 efficacy study as part of an accelerated development plan. The ongoing Phase 2 and 3 studies will continue to have long-term follow-up in a placebo-controlled manner until agreement to unblind has been achieved. Placebo-controlled blinded data during long-term follow-up will provide high quality data on long-term safety and will supplement the open-label registry data. In this study, the requirement for entrance to the VAC52150EBL4001 roll-over study was limited to female subjects who became pregnant with estimated conception within 28 days after vaccination with MVA-BN-Filo (or placebo) or within 3 months after vaccination with Ad26.ZEBOV (or placebo) and children born to vaccinated female subjects who became pregnant with estimated conception within 28 days after vaccination with MVA-BN-Filo (or placebo) or within 3 months after vaccination with Ad26.ZEBOV (or placebo), unless local regulations have additional requirements for follow-up. Following these children for a total of 5 years was a commitment made to the relevant health authorities.

SYNOPSIS
3.1 Overview of Study Design
9.1.8 VAC52150 Vaccine Development Roll-over Study
12.3.4 Pregnancy

Rationale: The secondary objectives and endpoints were limited to immune responses measured by ELISA at 21 days post boost. Immune responses measured by ELISA at other relevant time points were shifted to the exploratory objectives and endpoints. As the exploratory endpoints can be presented in a separate biomarker report, there will be no delay in the preparation of the final clinical study report. Secondary safety objectives/endpoints and exploratory immunology objectives/endpoints with regard to the substudy were added.

SYNOPSIS
2.1 Objectives
11.1.2 Secondary Endpoint
11.1.3 Exploratory Endpoints

Rationale: As there is no analysis planned for the peripheral blood mononuclear cell (PBMC) sample taken on the day of the boost vaccination in children from Cohort 3 and for the immunogenicity sample taken on the 7-day post-boost visit in children from Cohorts 2b and 3, these samples were removed.

Time and Events Schedule

9.1.1 Overview

9.4 Immunogenicity Evaluations

16.1 Study-specific Design Considerations

Rationale: Exclusion criterion 29 was updated to specify that pediatric subjects with weight-per-height below 10th percentile according to Centers for Disease Control and Prevention growth charts should be excluded.

4.2.3 Exclusion Criteria for Pediatric Subjects

Rationale: The statistical methods for clinical laboratory tests, vital signs, and physical examination were revised. Since only small mean fluctuations are expected, limited interest will be on the summary statistics over time and the analyses will focus on most severe abnormalities and toxicity gradings.

SYNOPSIS

11.5 Safety Analyses

Rationale: Inclusion criterion 15 for HIV-infected subjects was updated to add the use of viral load assessments to establish effectiveness of viral suppression, as the HIV-infected subjects must be on a stable regimen of Highly Active Antiretroviral Therapy whose purpose is to reduce viral load to undetectable levels.

4.1.2 Additional Inclusion Criteria for HIV-infected Subjects

Rationale: Minor textual changes have been made, in addition to updates to be in line with other current protocols.

SYNOPSIS

Time and Events Schedule

Additional Time and Events Schedule

Definitions of Terms

3.1 Overview of Study Design

4 SUBJECT POPULATION

4.2.1 Exclusion Criteria for Healthy Adult and Elderly Subjects

5 TREATMENT ALLOCATION AND BLINDING

9.1.1 Overview

9.1.3 Vaccination Phase (Prime and Boost)

9.1.5 Long-term Follow-up

9.3.1 Safety Assessments

10.2 Discontinuation of Study Vaccine/Withdrawal From the Study

12.3.4 Pregnancy

Rationale: The protocol has been updated to be in line with the current protocol template (version 1 November 2016).

TITLE PAGE

Amendment_2 (9 September 2016)

This amendment is considered to be substantial based on the criteria set forth in Article 10(a) of Directive 2001/20/EC of the European Parliament and the Council of the European Union.

The overall reason for the amendment: This amendment includes the request of the Center for Biologics Evaluation and Research (CBER, a division of United States [US] Food and Drug Administration [FDA]) to change the age ranges of Cohorts 3 and 4 and to extend the safety follow-up to 6 months post-boost. The latter request was originally made for other VAC52150 protocols and has now also been implemented for this protocol. The sponsor halted vaccinations following a case of Miller Fisher syndrome after receipt of MVA-BN-Filo or placebo in a clinical study. This interruption continued until a revised informed consent form (ICF) containing updated safety language for the current study VAC52150EBL2002 was prepared and approval to restart the study was granted by the relevant competent authority. As a result of the pause, some subjects will be outside the protocol-defined boost vaccination window. Information was added to clarify the procedures that need to be followed for these subjects. Based on the request of the Agence Nationale de Sécurité du Médicament et des produits de santé (ANSM) for study VAC52150EBL2001, the sponsor has decided to implement the collection of neurologic and autoimmune events ("Immediate Reportable Events") throughout the entire clinical development plan.

The changes made to the clinical protocol VAC52150EBL2002 Amendment 1 (dated 26 June 2015) are listed below, including the rationale of each change and a list of all applicable sections.

Rationale: As requested by the CBER (US FDA), the age ranges of Cohort 3 and Cohort 4 have been changed to 4 to 11 years (Cohort 3) and 1 to 3 years (Cohort 4) to align with World Health Organization (WHO)-defined age cohorts which effectively increases the number of subjects aged 1 to 3 years. Since this age group has a different immune response to vaccination than older children and adults, the increased sample size of Cohort 4 will provide deeper understanding of the immunogenicity of the vaccine.

SYNOPSIS
Time and Events Schedule
3.1 Overview of Study Design
4.1.3 Inclusion Criteria for Pediatric Subjects
6 DOSAGE AND ADMINISTRATION
9.3.1 Safety Assessments
9.4 Immunogenicity Evaluations

Rationale: As requested by the CBER (US FDA), the 6-month post-prime visit has been changed to 6-month post-boost visit (6-month post-prime visit will be retained for subjects who are outside the protocol-defined boost vaccination window and elect not to receive a late boost vaccination). In addition, a statement has been added that subjects who complete the 6-month post-prime visit (which has been replaced with the 6-month post-boost visit per the current protocol amendment) prior to approval of Amendment 2 will be required to also attend the 6-month post-boost visit after approval of Amendment 2.

SYNOPSIS
Time and Events Schedule
1.2.4 Potential Risks
3.1 Overview of Study Design
4.3 Prohibitions and Restrictions
5 TREATMENT ALLOCATION AND BLINDING
9.1.1 Overview
9.1.4 Post-vaccination Phase
9.1.5 Long-term Follow-up
11.6 Immunogenicity Analyses

Rationale: Information was added to clarify the procedures that need to be followed for subjects who are affected by a study pause. A separate Time and Events Schedule was created for subjects who were primed prior to a pause and do not/will not receive a boost vaccination because of a study pause. Because there will be subjects who do not receive a boost vaccination, some post-boost vaccination visits have been changed to post-last vaccination visits (eg, for collection of adverse events).

SYNOPSIS
Additional Time and Events Schedule
1.2.4 Potential Risks
3 STUDY DESIGN AND RATIONALE
4.2.1 Exclusion Criteria for Healthy Adult and Elderly Subjects
4.3 Prohibitions and Restrictions
9.1 Study Procedures
9.1.6 Procedures in Case of a Study Pause
9.3.1 Safety Assessments
10.1 Completion
11 STATISTICAL METHODS
12.3.1 All Adverse Events

Rationale: As requested by the CBER (US FDA), the safety laboratory assessments at screening are to be performed within 28 days prior to the prime vaccination and may be repeated if they fall outside this time window. Screening safety laboratory assessments can also be done on Day 1 before vaccination and subjects may be randomized on the same day when they satisfy all selection criteria; for example, if a woman returns for a repeat urinalysis due to menses, she may be randomized on that same day provided all selection criteria are met and results are known prior to randomization. If rescreening is required, all screening procedures (except TOU) should be repeated. As a result of the pause, subjects whose screening period was longer than the protocol-defined 8 weeks will be allowed to rescreen once, but must have new safety laboratory assessments (including HIV, follicle stimulating hormone [FSH] [if applicable], electrocardiogram [ECG], hematology, biochemistry, urinalysis, vital signs and physical examination) within 28 days of the prime vaccination. The TOU does not need to be repeated.

Time and Events Schedule
4.1.1 Inclusion Criteria for Healthy Adult and Elderly Subjects
4.1.3 Inclusion Criteria for Pediatric Subjects
9.1.2 Screening Phase

Rationale: A change in approximate whole blood volumes in Cohorts 3 and 4 is necessitated by a number of factors: (i) each site requires more than the previously allotted volumes to perform hematology and serum chemistry tests and each site requires slightly different volumes (hence, the blood volumes for safety endpoints have been deleted from the Time and Events Schedule); (ii) immunogenicity assays require a minimum blood volume to provide a sufficient sample for analysis; reducing the volumes any further jeopardizes the secondary endpoint in the study; (iii) there are technical issues with reducing the blood volumes with availability of appropriate phlebotomy tubes and additional problems associated with dividing a single sample into multiple aliquots in a sterile manner. The minimum whole blood volume that satisfies both the safety and immunogenicity endpoints will be taken.

Time and Events Schedule
9.1.1 Overview
9.3.1 Safety Assessments
9.4 Immunogenicity Evaluations
16.1 Study-specific Design Considerations

Rationale: The time of unblinding of sponsor personnel for regulatory submission has been changed to when the last subject in a cohort has completed the 6-month post-boost visit or discontinued earlier. All subjects within a cohort will be followed in a blinded manner by the site until the last subject in that cohort has completed the study. Any subject who completes either the 6-month post-prime and/or the Day 365 visit prior to approval of Amendment 2 will be required to attend the 6-month post-boost and the Day 365 visit after approval of Amendment 2. A subject will be considered to have completed the study if he or she has completed all assessments at the 6-month post-boost vaccination visit or at the Day 365 visit, whichever occurs later.

SYNOPSIS

Time and Events Schedule

1.2.4 Potential Risks

3.1 Overview of Study Design

5 TREATMENT ALLOCATION AND BLINDING

9.1.5 Long-term Follow-up

10.1 Completion

11.7 Interim Analyses

Rationale: The primary analysis has been changed to when all subjects in Cohort 1 have completed the 6-month post-boost visit or discontinued earlier.

SYNOPSIS

3.1 Overview of Study Design

11 STATISTICAL METHODS

Rationale: The interim analysis on safety and selected immunogenicity data in each cohort has been changed to when all subjects have completed the 6-month post-boost visit. Additional interim analyses during the study for the purpose of informing future vaccine-related decisions in a timely manner, were deleted.

SYNOPSIS

3.1 Overview of Study Design

5 TREATMENT ALLOCATION AND BLINDING

11.7 Interim Analyses

Rationale: As requested by the ANSM, wording on the collection of "Immediate Reportable Events" was added after one subject in study VAC52150EBL2001 experienced a serious and very rare condition called "Miller Fisher syndrome" about a month after boost vaccination with either MVA-BN-Filo or placebo.

SYNOPSIS
Time and Events Schedule
1.2.5 Overall Benefit/Risk Assessment
3.1 Overview of Study Design
8 PRESTUDY AND CONCOMITANT THERAPY
9.1.3 Vaccination Phase (Prime and Boost)
9.1.4 Post-vaccination Phase
9.1.5 Long-term Follow-up
9.1.6 Procedures in Case of a Study Pause
9.3.1 Safety Assessments
11.1.1 Primary Endpoints
12.1.1 Adverse Event Definitions and Classifications
12.2 Special Reporting Situations
12.3.1 All Adverse Events
12.3.3 Immediate Reportable Events

Rationale: Adverse events of special interest (cardiovascular events) will no longer be collected as no cardiovascular events have been associated with the current MVA-BN-Filo vaccine. Information was added on the procedure that needs to be followed in case any cardiac sign or symptom develops after the boost vaccination.

1.2.5 Overall Benefit/Risk Assessment
9.3.1 Safety Assessments
12.2 Special Reporting Situations
12.3.1 All Adverse Events

Rationale: The statement that a male subject's study vaccine should be permanently discontinued if his partner becomes pregnant, has been removed. The current biodistribution and reprotoxicity data support the recommendation that there is a negligible risk to the partner of a male vaccinated subject if she becomes pregnant.

10.2 Discontinuation of Study Vaccine

Rationale: The inclusion criteria have been modified to specify that subjects with hemoglobin lower than the institutional normal reference range should not be included in the study.

4.1.1 Inclusion Criteria for Healthy Adult and Elderly Subjects
4.1.3 Inclusion Criteria for Pediatric Subjects

Rationale: Criteria regarding contraceptive requirements have been clarified. In addition, the statement that the birth control method used by the female partner of a male subject should be documented, has been removed. The current biodistribution data suggest that there is no vaccine present in tissues other than the inoculated muscle and regional lymph nodes. Therefore, the risk to the partner of a male participant is negligible and it is unnecessary to confirm additional contraceptive use by the partner. Consistent condom use in the male participant will continue to be emphasized.

4.1.1 Inclusion Criteria for Healthy Adult and Elderly Subjects
4.1.3 Inclusion Criteria for Pediatric Subjects
4.3 Prohibitions and Restrictions

Rationale: One case of chest pain, that might be indicative of pericarditis and that was not finally confirmed, was observed in the MVA-BN clinical trial program and has been added to the Potential Risks section.

1.2.4 Potential Risks

Rationale: The analysis of response patterns over time for the immunologic parameters was deleted, since this analysis will not be performed.

SYNOPSIS

11.6 Immunogenicity Analyses

Rationale: Information regarding the marketing authorization of MVA-BN and the Phase 3 clinical study POX-MVA-013 has been updated. Safety information following MVA-BN-Filo vaccine administration based on the pooled safety data from studies VAC52150EBL1001 and VAC52150EBL1002 has been included.

1.1 Background

REFERENCES

Rationale: The VAC52150 Vaccine Development Registry was replaced by a roll-over study. The statement '(non-participating) partners of male subjects who become pregnant during the study and up to 3 months after the prime vaccination or up to 1 month after the boost vaccination (whichever is longer) will be approached to consent for enrollment of their children into the registry' (added per Amendment 1) has been removed, since these children will no longer be followed. Further details regarding enrollment have been added, such as the inclusion of placebo subjects before unblinding of the current study.

SYNOPSIS

3.1 Overview of Study Design

9.1.8 VAC52150 Vaccine Development Roll-over Study

12.3.4 Pregnancy

Rationale: Immunogenicity objectives, endpoints and assessments have been revised and corrected. All secondary immunogenicity objectives and endpoints were shifted to exploratory objectives and endpoints, except for immune responses measured by ELISA.

SYNOPSIS

2.1 Objectives

9.4 Immunogenicity Evaluations

11.1.2 Secondary Endpoint

11.1.3 Exploratory Endpoints

Rationale: Information on remote audits was added.

17.10 On-site Audits

Rationale: The name of the sponsor changed from Crucell Holland B.V. to Janssen Vaccines & Prevention B.V. (formerly known as Crucell Holland B.V.).

TITLE PAGE

1 INTRODUCTION

14.1 Description of Study Vaccines

INVESTIGATOR AGREEMENT

Rationale: The protocol has been updated to be in line with the current protocol template (version 6 June 2016).

4 SUBJECT POPULATION
9.1.3 Vaccination Phase (Prime and Boost)
9.1.4 Post-vaccination Phase
9.3.1 Safety Assessments
10 SUBJECT COMPLETION/DISCONTINUATION OF STUDY VACCINE/WITHDRAWAL FROM THE STUDY
12.1.1 Adverse Event Definitions and Classifications
12.2 Special Reporting Situations
12.3.1 All Adverse Events
12.3.4 Pregnancy
12.4 Contacting Sponsor Regarding Safety
13.2 Contacting Sponsor Regarding Product Quality
16.1 Study-specific Design Considerations
16.2.2 Independent Ethics Committee or Institutional Review Board
16.2.3 Informed Consent and Assent Form
16.2.5 Long-term Retention of Samples for Additional Future Research
16.2.6 Country Selection
17.1 Protocol Amendments
17.4 Source Documentation
17.5 Case Report Form Completion
17.6 Data Quality Assurance/Quality Control
17.7 Record Retention
17.8 Monitoring
17.9.1 Study Completion/End of Study
17.10 On-site Audits
17.11 Use of Information and Publication

Rationale: Minor textual changes have been made, in addition to modifications for clarity and updates to be in line with other current protocols.

SYNOPSIS
Time and Events Schedule
ABBREVIATIONS
Definitions of Terms
1 INTRODUCTION
3 STUDY DESIGN AND RATIONALE
4.1.1 Inclusion Criteria for Healthy Adult and Elderly Subjects
4.1.3 Inclusion Criteria for Pediatric Subjects
4.2.3 Exclusion Criteria for Pediatric Subjects
5 TREATMENT ALLOCATION AND BLINDING
6.1 General Instructions and Procedures
6.3 Contraindications to Boost Vaccination
9.1.1 Overview
9.1.2 Screening Phase
9.1.5 Long-term Follow-up
9.3 Safety Evaluations
9.4 Immunogenicity Evaluations
10.1 Completion
10.2 Discontinuation of Study Vaccine/Withdrawal From the Study
11.2 Sample Size Determination
11.3 Analysis Sets
12.1.1 Adverse Event Definitions and Classifications
17.10 On-site Audits
ATTACHMENTS

Amendment_1 (26 June 2015)

The overall reason for the amendment: This amendment was written in response to the Assisted Review of the Janssen Ebola Zaire Vaccine Clinical Trials Application meeting of 9-11 June 2015. A few additional changes (not triggered by the meeting) have been implemented. Further, modifications were made throughout the protocol to correct minor errors, inconsistencies and/or for clarity.

The table below gives an overview of the rationale for each change and all applicable sections.

The following changes were made following the meeting:

Rationale: The 84-day prime-boost interval has been dropped in Cohorts 2 to 4. The sample size of the dropped regimen in Cohort 2 to 4 has been redistributed to the 28- and 56-day prime-boost intervals in Cohort 1 to increase the number of adult or elderly subjects. Consequently, the primary analysis will be performed when all subjects in Groups 1 and 2 in Cohort 1 have completed the 42-day post-boost visit or discontinued earlier.

SYNOPSIS
Time and Events Schedule
2.1 Objectives
3.1 Overview of Study Design
3.2 Study Design Rationale
5 TREATMENT ALLOCATION AND BLINDING
6 DOSAGE AND ADMINISTRATION
11 STATISTICAL METHODS
11.2 Sample Size Determination
11.7 Interim Analyses

Rationale: Further text has been added to clarify the process for progression to the next cohort, and a change has been implemented for Cohort 4. An interim analysis will be performed after the first 36 subjects in Cohort 3 have completed their 21-day post-boost visit on all available safety and immunogenicity data collected to this point in the study, for the purpose of enrolling Cohort 4.

SYNOPSIS
3.1 Overview of Study Design
11.7 Interim Analyses
11.8 Independent Data Monitoring Committee

Rationale: Also partners of male subjects who become pregnant during the study and up to 3 months after the prime vaccination or up to 1 month after the boost vaccination (whichever is longer) will be approached to consent for enrollment of their children into the registry. These children will be followed for a total period of 5 years.

9.1.8 VAC52150 Vaccine Development
12.3.4 Pregnancy

Rationale: The wording 'health care worker' has been replaced with language appropriate for the different sites: '*For illiterate subjects or those having difficulty completing the diary independently, the sites will make arrangements to have the diary completed according to their local practice*'.

SYNOPSIS
Time and Events Schedule
1.2.5 Overall Benefit/Risk Assessment
3.1 Overview of Study Design
9.1.3 Vaccination Phase (Prime and Boost)

Rationale: Follow-up of CD4+ cell count in the HIV-infected cohort will be part of the clinical laboratory assessments with sampling at screening, on Day 1, on the day of boost vaccination and at 42 days after the boost vaccination.

Time and Events Schedule

9.3.1 Safety Assessments

Rationale: The test of understanding (TOU) will be administered after reading, but before signing, the informed consent form (ICF)/assent.

Time and Events Schedule

9.1.2 Screening Phase

16.1 Study-specific Design Considerations

Rationale: Further clarification on the approximate blood volumes collected for the different study procedures at each scheduled visit and the cumulative blood draws have been added.

Time and Events Schedule

9.1.1 Overview

16.1 Study-specific Design Considerations

Rationale: The inclusion criteria have been modified to specify that subjects with hemoglobin outside the local laboratory's reference range should not be included in the study. Appropriate hemoglobin cut-off values for all subjects have been added in case reference ranges for the local laboratories do not exist.

4.1.1 Inclusion Criteria for Healthy Adult and Elderly Subjects

4.1.3 Inclusion Criteria for Pediatric Subjects

ATTACHMENTS

Rationale: Clarification is added that subjects with malignancies who are currently being treated or are not surgical cures are excluded to ensure that a subject's health status is adequate.

4.2.1 Exclusion Criteria for Healthy Adult and Elderly Subjects

---

Rationale: Text concerning the investigator's responsibility to ensure adequate counseling and follow-up will be made available if the HIV or pregnancy test is positive in order to maintain subject confidentiality has been added.

4.1.1 Inclusion Criteria for Healthy Adult and Elderly Subjects

4.2.1 Exclusion Criteria for Healthy Adult and Elderly Subjects

4.1.3 Inclusion Criteria for Pediatric Subjects

4.2.3 Exclusion Criteria for Pediatric Subjects

Rationale: The language on the monitoring of subjects following each vaccination has been clarified. In addition, text concerning the investigator's responsibility to provide emergency medical care during the post-vaccination observation period and to make arrangements for ongoing care has been added.

SYNOPSIS

1.2.5 Overall Benefit/Risk Assessment

3.1 Overview of Study Design

6.1 General Instructions and Procedures

9.3.1 Safety Assessments

Rationale: Any safety reports/interim data reviewed by the IDMC and the recommendations of the IDMC will be shared with the local health authorities and the IECs/IRBs.

11.8 Independent Data Monitoring Committee

Rationale: The statement that '*the results from Ebola virus serological screening tests should be interpreted with caution as the subject could have a false positive test (not truly infected with Ebola virus) as a result of an immune response to the candidate Ebola vaccine'* has been specified on the 'wallet (study) card'.

12.3.1 All Adverse Events

Rationale: The sponsor will establish a Material Transfer Agreement between all relevant parties taking into account national regulations.

16.2.5 Long-term Retention of Samples for Additional Future Research

The following additional changes were made:

Rationale: The diary should be completed at approximately the same time each day.

SYNOPSIS
Time and Events Schedule
1.2.5 Overall Benefit/Risk Assessment
3.1 Overview of Study Design
9.1.3 Vaccination Phase (Prime and Boost)
9.3.1 Safety Assessments
12.3.1 All Adverse Events

Rationale: An absolute number (instead of a proportion) for subjects contributing to the PBMC samples has been provided.

SYNOPSIS
Time and Events Schedule
3.1 Overview of Study Design
9.4 Immunogenicity Evaluations

Rationale: All urinalyses should be collected as freshly voided, mid-stream, clean catch samples obtained after proper cleansing.

4.1.1 Inclusion Criteria for Healthy Adult and Elderly Subjects
4.1.3 Inclusion Criteria for Pediatric Subjects

Rationale: Any vaccines received during the study should be recorded.

8 PRESTUDY AND CONCOMITANT THERAPY

Rationale: Modifications to correct minor errors, inconsistencies and/or for clarity throughout the document. The selection criteria have been reorganized in order to group all inclusion and all exclusion criteria.

SYNOPSIS

A Randomized, Observer-blind, Placebo-controlled, Phase 2 Study to Evaluate the Safety, Tolerability and Immunogenicity of Different Prime-boost Regimens of the Candidate Prophylactic Vaccines for Ebola Ad26.ZEBOV and MVA-BN-Filo in Healthy Adults, Including Elderly Subjects, HIV-infected Subjects, and Healthy Children in Two Age Strata in Africa

The sponsor, in collaboration with Bavarian Nordic (BN) and in conjunction with an Innovative Medicines Initiative (IMI) consortium led by the Institut National de la Santé et de la Recherche Médicale (INSERM), is investigating the potential of a prophylactic Ebola vaccine regimen comprised of the following 2 candidate Ebola vaccines:

Ad26.ZEBOV is a monovalent vaccine expressing the full length Ebola virus (EBOV, formerly known as *Zaire ebolavirus*) Mayinga glycoprotein (GP), and is produced in the human cell line PER.C6.

MVA-mBN226B, further referred to as Modified Vaccinia Ankara (MVA)-BN-Filo, is a multivalent vaccine expressing the Sudan virus (SUDV) GP, the EBOV GP, the Marburg virus (MARV) Musoke GP, and the Tai Forest virus (TAFV, formerly known as *Côte d'Ivoire ebolavirus*) nucleoprotein (NP), and is produced in chicken embryo fibroblast cells. The EBOV GP expressed by MVA-BN-Filo has 100% homology to the one expressed by Ad26.ZEBOV**.**

In this Phase 2 study, the sponsor's adenovirus serotype 26 (Ad26) vector expressing the EBOV Mayinga GP (Ad26.ZEBOV) and the MVA-BN vector with EBOV, SUDV and MARV GP inserts and TAFV NP insert (MVA-BN-Filo) will be evaluated as a heterologous prime-boost regimen, in which one study vaccine (Ad26.ZEBOV) is used to prime a filovirus-specific immune response and the other study vaccine (MVA-BN-Filo) is used to boost the immune response 28, 56 or 84 days later. In a substudy, Ad26.ZEBOV is also used as a third vaccination. The EBOV GP that is currently circulating in West Africa has 97% homology to the EBOV GP used in this vaccine regimen.

OBJECTIVES AND HYPOTHESIS

Primary Objective

The primary objective is to assess the safety and tolerability of different vaccination schedules of Ad26.ZEBOV and MVA-BN-Filo administered intramuscularly (IM) as heterologous prime-boost regimens on Days 1 and 29, Days 1 and 57, or Days 1 and 85, in healthy adults, including elderly subjects, and on Days 1 and 29 and Days 1 and 57 in human immunodeficiency virus (HIV)-infected subjects and healthy children in 2 age strata.

Secondary Objectives

The secondary objectives are:

- To assess humoral immune responses, as measured by enzyme-linked immunosorbent assay (ELISA), to the EBOV GP at 21 days post boost of different vaccination schedules of Ad26.ZEBOV and MVA-BN-Filo administered IM as heterologous prime-boost regimens on Days 1 and 29, Days 1 and 57, or Days 1 and 85, in healthy adults, including elderly subjects, and on Days 1 and 29 and Days 1 and 57 in HIV-infected subjects and healthy children in 2 age strata.
- To assess the safety and tolerability of a third vaccination with Ad26.ZEBOV administered at least 1 year post prime in a subset of approximately 90 healthy adults, including elderly subjects, in Cohort 1 (Groups 1 and 2).

Exploratory Objectives

The exploratory objectives are:

- To assess humoral immune responses, as measured by ELISA, to the EBOV GP at other relevant time points of different vaccination schedules of Ad26.ZEBOV and MVA-BN-Filo administered IM as heterologous prime-boost regimens on Days 1 and 29, Days 1 and 57, or Days 1 and 85, in healthy adults, including elderly subjects, and on Days 1 and 29 and Days 1 and 57 in HIV-infected subjects and healthy children in 2 age strata.
- To assess the neutralizing capacity of the EBOV GP-specific humoral immune responses, as measured by virus neutralization assay, to the EBOV GP of different vaccination schedules of Ad26.ZEBOV and MVA-BN-Filo administered IM as heterologous prime-boost regimens on Days 1 and 29, Days 1 and 57, or Days 1 and 85, in healthy adults, including elderly subjects, and on Days 1 and 29 and Days 1 and 57 in HIV infected subjects and healthy children in 2 age strata.
- To explore humoral and cellular immune responses to different EBOV GPs and/or the adenovirus and/or MVA backbones of the various vaccination schedules tested.
- To explore humoral and cellular immune responses to filovirus GPs and/or TAFV NP, if assays are available.
- To assess humoral immune responses, as measured by ELISA, to the EBOV GP at various time points following a third vaccination with Ad26.ZEBOV at least 1 year post prime in approximately 90 healthy adults, including elderly subjects, in Cohort 1 (Groups 1 and 2).

HYPOTHESIS

As this study is designed to provide descriptive information regarding safety and immunogenicity without formal treatment comparisons, no formal statistical hypothesis testing is planned.

OVERVIEW OF STUDY DESIGN

This is a randomized, observer-blind, placebo-controlled, parallel-group, multicenter, Phase 2 study in Africa to evaluate the safety, tolerability and immunogenicity of different heterologous prime-boost regimens using Ad26.ZEBOV at a dose of $5x10^{10}$ viral particles (vp) as prime and MVA-BN-Filo at a dose of $1x10^{8}$ infectious units (Inf U, nominal titer) as boost at a 28-, 56- or 84-day interval in healthy adult and elderly subjects. The same prime-boost schedules, except for the 84-day interval, will be evaluated in HIV-infected subjects and healthy children in 2 age strata. At selected sites in Cohort 1 (Groups 1 and 2), a third vaccination with Ad26.ZEBOV will be administered at least 1 year post prime to subjects who consent to this (Cohort 1 substudy). Subjects who received a late boost vaccination or did not receive the boost vaccination at all due to a study pause will not be included in the Cohort 1 substudy.

This study will enroll a planned total number of 1,056 subjects, who have never received a candidate Ebola vaccine and have no prior exposure to Ebola virus (including travel to epidemic Ebola areas less than 1 month prior to screening) or a diagnosis of Ebola virus disease.

The study is comprised of 3 cohorts, which will be enrolled sequentially: healthy adult and elderly subjects (aged 18 years up to 70 years inclusive, Cohort 1), HIV-infected subjects (aged 18 to 50 years inclusive, Cohort 2a) and healthy children in 2 age strata (adolescents aged 12 to 17 years inclusive, Cohort 2b; children aged 4 to 11 years inclusive, Cohort 3). The prime-boost regimens that will be evaluated in this study will differ only in the timing of the boost vaccination (ie, 28, 56 or 84 days after prime, respectively referred to as Groups 1, 2 and 3), while the dose of each study vaccine (Ad26.ZEBOV, MVA-BN-Filo or placebo) and the sequence of prime-boost vaccination will be identical. Groups 1 and 2 will be evaluated in each cohort, while Group 3 (84-day interval) will be evaluated in Cohort 1 only.

In Cohort 1, subjects will be enrolled in parallel and randomized in a 1:1:1 ratio to Groups 1, 2 and 3 at baseline until a target of 132 subjects have been included in Group 3. Afterwards, randomization in this cohort will proceed in a 1:1 ratio to Groups 1 and 2. In Cohorts 2a, 2b, and 3, subjects will be enrolled in parallel and randomized in a 1:1 ratio to Groups 1 and 2 at baseline. Within each group (for all cohorts), subjects will be randomized in a 5:1 ratio to receive Ad26.ZEBOV and MVA-BN-Filo versus placebo.

Available interim analysis results of 7-day post-prime safety and immunogenicity data from the ongoing and/or planned Phase 1 studies (studies in Europe, United States [US] and Africa) will be provided before the start of the present study, which will start with Cohort 1. Progression to the next cohort will proceed following favorable Independent Data Monitoring Committee (IDMC) review:

- Cohorts 2a and 2b can start when 25% of subjects in Cohort 1 have completed their 7-day post-prime visit.
- Cohort 3 can start when 50% of subjects in Cohort 2b have completed their 7-day post-prime visit.

The real-time monitoring of blinded data will be conducted by the sponsor. The interim data (unblinded) will be provided to and reviewed by the IDMC. The decision to open Cohorts 2a, 2b, and 3 will be based on the sponsor's and the IDMC's review of the interim safety data. Progression to the next cohort will also take into account pre-specified pausing rules.

For each cohort, unblinding of sponsor personnel to study vaccine allocation will occur after the last subject in that cohort has completed the 6-month post-boost visit or discontinued earlier. All subjects within a cohort will be followed in a blinded manner by the site until the last subject in that cohort has completed the study. A subject will be considered to have completed the study if he or she has completed all assessments at the 6-month post-boost vaccination visit or at the Day 365 visit (1-year post third vaccination visit for Cohort 1 substudy), whichever occurs later.

All subjects will receive the study vaccine through IM injection in the deltoid muscle, either Ad26.ZEBOV ($5x10^{10}$ vp) on Day 1, followed by a boost vaccination of MVA-BN-Filo ($1x10^{8}$ Inf U, nominal titer) on Day 29, 57 or 85; or placebo (0.9% saline) on Day 1, followed by a boost vaccination of placebo (0.9% saline) on Day 29, 57 or 85.

In a substudy at selected sites, approximately 90 healthy adult subjects in Cohort 1 (Groups 1 and 2) will receive a third vaccination with Ad26.ZEBOV at $5x10^{10}$ vp or placebo at least 1 year post prime vaccination. Subjects who received Ad26.ZEBOV and MVA-BN-Filo will receive Ad26.ZEBOV as third vaccination. Subjects who received placebo will receive placebo as third vaccination.

The study consists of a screening phase of up to 8 weeks (starting from the moment the subject signs the informed consent form [ICF] and/or assent), a vaccination phase in which subjects will be vaccinated at baseline (Day 1) followed by a boost vaccination on Day 29, 57 or 85, a post-vaccination phase and long-term follow-up phase until Day 365. The Cohort 1 substudy consists of a vaccination phase in which subjects will be vaccinated at least 1 year post prime vaccination, a post-vaccination phase, and a long-term follow-up phase until the 1-year post third vaccination visit. All subjects within a cohort will be followed in a blinded manner by the site until the last subject in that cohort has completed the study. Any subject who completed either the 6-month post-prime and/or the Day 365 visit prior to approval of Amendment 2 was required to attend the 6-month post-boost and the Day 365 visit after approval of Amendment 2.

While Cohort 1 was enrolling, the sponsor halted all vaccinations in this study due to the occurrence of a serious and very rare condition, Miller Fisher syndrome, reported in study VAC52150EBL2001, until a revised ICF was prepared and approval to restart the study was granted by the relevant competent authority. This interruption in dosing affected over 200 consented subjects, some who were awaiting prime vaccination and some awaiting boost vaccination. When approval was granted to restart the study under Amendment 1, a late boost vaccination has been offered to those subjects who were out-of-window

for the boost vaccination, unless it was prohibited. Those subjects who agreed to the late boost have been following the procedures as outlined in Amendment 1.

All subjects (including placebo) who were primed prior to the pause and did not/will not receive a late boost vaccination are being followed every 3 months after the prime vaccination for safety until Day 365 (see Additional Time and Events Schedule for subjects who do not receive a boost vaccination because of a study pause). Therefore, non-serious adverse events will be reported until the 42-day post-**last** vaccination (excluding third vaccination) visit rather than the 42-day post-boost vaccination visit. Serious adverse events and immediate reportable events (IREs) will be reported throughout the study until Day 365.

Female subjects who became pregnant with estimated conception within 28 days after vaccination with MVA-BN-Filo (or placebo) or within 3 months after vaccination with Ad26.ZEBOV (or placebo) and children born to vaccinated female subjects who became pregnant with estimated conception within 28 days after vaccination with MVA-BN-Filo (or placebo) or within 3 months after vaccination with Ad26.ZEBOV (or placebo) (unless local regulations have additional requirements for follow-up) will be eligible for enrollment into the VAC52150 Vaccine Development Roll-over study (VAC52150EBL4001) for long-term surveillance (for a total of up to 60 months after the prime vaccination). After unblinding, only female subjects and the children of female subjects who received Ad26.ZEBOV and/or MVA-BN-Filo will remain in the VAC52150 Vaccine Development Roll-over study for long-term surveillance. After unblinding, female subjects and the children of female subjects who received placebo and have already been enrolled into the VAC52150 Vaccine Development Roll-over study will be discontinued from further participation in the roll-over study.

SUBJECT POPULATION

Screening of subjects for eligibility will be performed within 8 weeks before administration of study vaccine on Day 1. The study population will consist of a planned total number of 1,056 subjects, including healthy adult and elderly subjects (aged 18 to 70 years inclusive, Cohort 1), HIV-infected subjects (aged 18 to 50 years inclusive, Cohort 2a), and healthy children in 2 age strata (adolescents aged 12 to 17 years inclusive, Cohort 2b; children aged 4 to 11 years inclusive, Cohort 3) in Africa.

DOSAGE AND ADMINISTRATION

Study vaccines (Ad26.ZEBOV, MVA-BN-Filo or placebo) will be administered as 0.5-mL IM injections into the deltoid muscle. The boost vaccination should be administered in the opposite deltoid from the prime vaccination. The third vaccination can be administered in either deltoid.

All subjects will receive a vaccination, according to randomization, on Day 1 (Groups 1 to 3) and on Day 29 (Group 1), Day 57 (Group 2) or Day 85 (Group 3) at the following dose levels:

- Ad26.ZEBOV: $5x10^{10}$ vp, supplied in a single use vial (0.5 mL extractable);
- MVA-BN-Filo: $1x10^{8}$ Inf U (nominal titer; target fill is $1.9x10^{8}$ Inf U per dose, range: $1.27\text{-}2.67x10^{8}$ Inf U), supplied in a single use vial (0.5 mL extractable);
- Placebo: 0.9% saline (0.5 mL extractable).

At selected sites, approximately 90 healthy adult subjects in Cohort 1 (Groups 1 and 2) will receive a third vaccination with Ad26.ZEBOV at $5x10^{10}$ vp or placebo at least 1 year post prime vaccination (substudy).

Subjects will remain at the site for a total of 60 (±15) minutes (after prime and boost vaccinations) or 30 (±15) minutes (after the third vaccination) post-vaccination to monitor for the development of any acute reactions, or longer if deemed necessary by the investigator (eg, in case of grade 3 adverse events).

Criteria for postponement of vaccination at the scheduled time for vaccine administration and contraindications to boost and third vaccinations have been defined and will be applied by the investigator (not including those occurring because of study hold).

SAFETY EVALUATIONS

Safety evaluations will be performed as specified in the Time and Events Schedule.

Safety will be assessed by collection of solicited local and systemic adverse events (reactogenicity), unsolicited adverse events, serious adverse events, and IREs. The subjects will be closely observed by study-site personnel for the first 30 (±10) minutes after each vaccination, and again at 60 (±15) minutes after prime and boost vaccinations, and any unsolicited, solicited local or systemic adverse events will be documented during this period. Upon discharge from the site, subjects will receive a diary, a thermometer and a ruler to measure body temperature and solicited local reactions. Subjects will be instructed to record solicited local and systemic adverse events in the diary in the evening after each vaccination and then daily for the next 7 days at approximately the same time each day. Diaries should be completed at home by the subject (or parent, legal guardian or caregiver, as applicable). For illiterate subjects or those having difficulty completing the diary independently, the sites will make arrangements to have the diary completed according to their local practice. The investigator will document unsolicited adverse events from signing of the ICF/assent onwards until 42 days post-last vaccination (excluding the third vaccination), and again from the day of the third vaccination until 28 days thereafter for subjects in the Cohort 1 substudy (note: events that started before the third vaccination but are still present at the time of third vaccination should also be recorded). The investigator will document serious adverse events and IREs from signing of the ICF/assent onwards until the end of the study. The primary (or secondary for Cohort 1 substudy) endpoints are adverse events, serious adverse events, IREs, and solicited local and systemic adverse events. Adverse events that are ongoing at 42 days post-last vaccination (excluding third vaccination), or at 28 days post third vaccination for subjects in the Cohort 1 substudy, will be followed until resolution or stabilization.

Other safety assessments include an electrocardiogram (ECG; for subjects ≥18 years of age, performed at screening), physical examination, vital signs (blood pressure, pulse/heart rate, body temperature), clinical laboratory and pregnancy testing.

The investigators, together with the sponsor's medical monitor, will be responsible for the safety monitoring of the study, and will halt vaccination of further subjects in case any of the pre-specified pausing rules have been met.

IMMUNOGENICITY EVALUATIONS

The investigator will collect samples for immunogenicity assessments as specified in the Time and Events Schedule, for evaluation of secondary and exploratory endpoints. Samples to assess humoral responses will be taken from all subjects; samples to assess cellular immune responses will be taken from subjects at selected sites with the capabilities to process peripheral blood mononuclear cells (PBMC) (targeted at 165 subjects [138 Ad26.ZEBOV and MVA-BN-Filo, and 27 placebo] in Cohort 1 and 33 subjects [28 Ad26.ZEBOV and MVA-BN-Filo, and 5 placebo] in each of the other cohorts). PBMC sampling at selected sites will continue until the targeted number of subjects is reached. Samples to assess cellular immune responses are optional for subjects in the Cohort 1 substudy and may only be collected in a subset of subjects. Subjects giving informed consent for the study will be informed that their leftover blood samples will be stored for potential future research. Subjects participating at selected sites where PBMC samples are collected will be asked explicitly to consent for potential future genetic research to be performed on PBMC samples. Subjects can withdraw consent for their samples to be used for future research at any time.

STATISTICAL METHODS

An overall planned sample size of 1,056 subjects includes 880 subjects to receive Ad26.ZEBOV and MVA-BN-Filo to substantially contribute to an overall safety database of the Ad26.ZEBOV and MVA-BN-Filo prime-boost regimen.

In Cohort 1, a total of 550 subjects are planned to be vaccinated with Ad26.ZEBOV and MVA-BN-Filo across the vaccination schedules (220 in Groups 1 and 2, 110 in Group 3). In the other cohorts (2a, 2b, and 3), 55 subjects per vaccination schedule (Groups 1 and 2) are planned to be vaccinated with Ad26.ZEBOV and MVA-BN-Filo, or a total of 110 subjects per subpopulation.

In case a specific adverse event is not observed, the one-sided 97.5% upper confidence limit of the true incidence rate of this adverse event is less than 6.5%, 3.3%, 1.7%, 0.8%, 0.7% and 0.5%, for sample sizes of 55, 110, 220, 440, 550 and 880 subjects, respectively.

The primary analysis will be conducted when all subjects in Cohort 1 have completed the 6-month post-boost visit or discontinued earlier, to aid in the assessment of which vaccination schedule will be the best candidate for future studies and implementation in the field. Study-site personnel, subjects and sponsor personnel involved in subject level data review will remain blinded until the last subject in that cohort has completed the study. Sponsor personnel involved in the conduct of the primary analysis and in making future decisions for the program will be unblinded to the data of Cohort 1, but will remain blinded to the data of the other cohorts.

For the Cohort 1 substudy of subjects who received the third vaccination, an interim analysis may be performed when all subjects have completed the 6-month post third vaccination visit, or discontinued earlier.

In each cohort, after completion of the 6-month post-boost visit by all subjects, an interim analysis will be conducted on safety and selected immunogenicity data and the cohort will be unblinded to the sponsor. Subjects who completed the 6-month post-prime visit prior to approval of Amendment 2 were required to attend the 6-month post-boost visit after approval of Amendment 2.

The final analysis will be performed when all subjects have completed the last study-related visit or discontinued earlier (including subjects participating in the Cohort 1 substudy).

Specific details will be provided in the Statistical Analysis Plan (SAP).

An IDMC will be established to monitor data on a regular basis to ensure the continuing safety of the subjects enrolled in the study.

Safety Analyses

No formal statistical testing of safety data is planned. Safety data will be analyzed descriptively (including 95% confidence intervals, if applicable). For each adverse event, the number and percentage of subjects who experience at least 1 occurrence of the given event will be summarized by group. Summaries, listings, datasets, or subject narratives may be provided, as appropriate, for those subjects who die, who discontinue study vaccine due to an adverse event, or who experience a severe or a serious adverse event. The most severe laboratory abnormalities following vaccination will be tabulated. Abnormalities in vital signs and physical examination following vaccination will be tabulated by most severe abnormality grade. Similar safety summaries will be created for the subjects who received a third vaccination.

Immunogenicity Analyses

Descriptive statistics (actual values and changes from baseline, including 95% confidence intervals, if applicable) will be calculated for continuous immunologic parameters at each time point analyzed. Graphical representations of changes in immunologic parameters will be prepared, as applicable. Frequency tabulations will be calculated for discrete (qualitative) immunologic parameters at each time point analyzed. In addition, differences between the vaccination schedules will be described at the 21-day post-boost, 6-month post-boost, and Day 365 visits. Similar summaries will be provided for the time points after the third vaccination for the Cohort 1 substudy population.

TIME AND EVENTS SCHEDULE

Cohort 1 (Groups 1-3) and Cohorts 2a and 2b (Groups 1-2)

**Group**	**Screening Phase[a] (≤8 weeks)**	**Vaccination Phase and Post-vaccination Phase[b]**									**Long-term Follow-up Phase[b,c]**	
**Group 1 (Cohorts 1, 2a, 2b)**		**D1**	**D2-D7**	**D8**	**-**	**D29**	**D30-D35**	**D36**	**D50**	**D71[d]**	**D209**	**D365**
**Group 2 (Cohorts 1, 2a, 2b)**					**D15**	**D57**	**D58-D63**	**D64**	**D78**	**D99[d]**	**D237**	
**Group 3 (Cohort 1 only)**					**D15**	**D85**	**D86-D91**	**D92**	**D106**	**D127[d]**	**D265**	
**Study procedures**		**Prime**		+7d pp	+14d pp	**Boost**		+7d pb	+21d pb	+42d pb	+6m pb[e]	+1y pp
Screening/Administrative												
Informed consent/assent[f]	X											
Inclusion/exclusion criteria	X[g]											
Medical history and demographics	X											
Prestudy therapies[h]	X											
Serum pregnancy test[i]	X											
HIV-1/2 serology (Cohorts 1 and 2b only)	X											
Follicle-stimulating hormone (FSH)[j]	X											
Test of Understanding (TOU) [f]	X											
Check clinical status + available data		X[k]										
Randomization		X										
Study Vaccine Administration[l]		▲				▼						
Safety Assessments												
Urine Pregnancy test[i]		X[m]				X[m]						
Electrocardiogram (ECG)[n]	X											
Physical examination[o]	X	X[m]		X		X[m]		X	X	X	X	X
Vital signs[p]	X	X[m]				X[m]						
Distribution of subject diary		X				X						
Completion of diary at home[q]			X				X					
Review of diary by site staff				X				X				
Adverse events[r]	Continuous											
Serious adverse events and immediate reportable events[s]	Continuous											
Concomitant medications	X	X	X	X	X[t]	X	X	X	X	X	X[u]	X[u]

**Group**	**Screening Phase[a] (≤8 weeks)**	**Vaccination Phase and Post-vaccination Phase[b]**									**Long-term Follow-up Phase[b,c]**	
**Group 1 (Cohorts 1, 2a, 2b)**		**D1**	**D2-D7**	**D8**	**-**	**D29**	**D30-D35**	**D36**	**D50**	**D71[d]**	**D209**	**D365**
**Group 2 (Cohorts 1, 2a, 2b)**					**D15**	**D57**	**D58-D63**	**D64**	**D78**	**D99[d]**	**D237**	
**Group 3 (Cohort 1 only)**					**D15**	**D85**	**D86-D91**	**D92**	**D106**	**D127[d]**	**D265**	
**Study procedures**		**Prime**		+7d pp	+14d pp	**Boost**		+7d pb	+21d pb	+42d pb	+6m pb[e]	+1y pp
Clinical Laboratory Assessments												
Chemistry, hematology (10 mL)[v]	X	X[m]		X		X[m]		X				
CD4+ cell count (2 mL)[w]	X[w]	X[w]				X[w]				X[w]		
Urinalysis	X											
Immunogenicity Assessments[x]												
Blood sampling (serum) for assessment of humoral immune responses (10 mL)		X[m]			X[t]	X[m]		X[y]	X		X	X
Blood sampling (PBMC) for assessment of cellular immune responses (40 or 20 mL)[z]		X[m]			X[t]	X[m]		X[y]	X		X	X
Approximate Blood Volumes[aa] with [without] PBMC												
- By visit (mL)[bb]												
Cohort 1 (Group 1)	10	60 [20]		10	0	60 [20]		60 [20]	50 [10]	0	50 [10]	50 [10]
Cohort 1 (Groups 2 and 3)	10	60 [20]		10	50[t] [10]	60 [20]		60 [20]	50 [10]	0	50 [10]	50 [10]
Cohort 2a	12	62 [22]		10	0	62 [22]		60 [20]	50 [10]	2	50 [10]	50 [10]
Cohort 2b (12- to 17-year old)	10	40 [20]		10	0	40 [20]		10	30 [10]	0	30 [10]	30 [10]
- Cumulative (mL)[bb]												
Cohort 1 (Group 1)	10	70 [30]		80 [40]	80 [40]	140 [60]		200 [80]	250 [90]	250 [90]	300 [100]	350 [110]
Cohort 1 (Groups 2 and 3)	10	70 [30]		80 [40]	130[t] [50]	190 [70]		250 [90]	300 [100]	300 [100]	350 [110]	400 [120]
Cohort 2a	12	74 [34]		84 [44]	84 [44]	146 [66]		206 [86]	256 [96]	258 [98]	308 [108]	358 [118]
Cohort 2b (12- to 17-year old)	10	50 [30]		60 [40]	60 [40]	100 [60]		110 [70]	140 [80]	140 [80]	170 [90]	200 [100]

▲ *Ad26.ZEBOV 5x10^{10} vp or placebo* ▼ *MVA-BN-Filo 1x10^{8} Inf U or placebo*
d pp: days post-prime; d pb: days post-boost; m pb: months post-boost; y pp: year post-prime

NOTE: In case of early withdrawal from the study, early withdrawal assessments (ie, physical examination and immunogenicity assessments for serum and PBMCs) should be obtained. Subjects who wish to withdraw consent will be offered an optional visit for safety follow-up (before the formal withdrawal of consent); the subject has the right to refuse.

a. Screening may be split into multiple days or visits. Retesting of values (eg, safety laboratory) that lead to exclusion is allowed once using an unscheduled visit during the screening phase. The safety laboratory assessments at screening are to be performed within 28 days prior to the prime vaccination (including Day 1 before vaccination) and may be repeated if they fall outside this time window. If rescreening is required, all screening procedures (except TOU) should be repeated. Similarly, subjects that are rescreened due to the pause must have new safety laboratory assessments (including HIV, FSH [if applicable], ECG, hematology, biochemistry, urinalysis, vital signs and physical examination) within 28 days of the prime vaccination. The TOU does not need to be repeated.
b. For subjects who receive a late boost vaccination, the timings of the next post-boost visits will be determined relative to the actual day of the boost vaccination.
c. For each cohort, all subjects will be followed up in a blinded manner by the site for the collection of serious adverse event information, immediate reportable events, and for blood draws for immunogenicity assessments until the last subject in that cohort has completed the study. Any subject who completed either the 6-month post-prime and/or the Day 365 visit prior to approval of Amendment 2 was required to attend the 6-month post-boost and the Day 365 visit after approval of Amendment 2. At selected sites in Cohort 1 (Groups 1 and 2), a third vaccination with Ad26.ZEBOV will be administered at least 1 year post prime to subjects who consent to this (for details, refer to Cohort 1 Substudy Time and Events Schedule).
d. In addition to the assessments scheduled for the 42-day post-boost visit, subjects will be instructed to contact the investigator before the next visit if they experience any adverse event or intercurrent illness that they perceive as relevant and/or can be possibly related to study vaccine in their opinion. All subjects within a cohort will be followed in a blinded manner by the site until the last subject in that cohort has completed the study. Any subject who completed either the 6-month post-prime and/or the Day 365 visit prior to approval of Amendment 2 was required to attend the 6-month post-boost and the Day 365 visit after approval of Amendment 2.
e. Subjects who completed the 6-month post-prime visit (which has been replaced with the 6-month post-boost visit per protocol Amendment 2) prior to approval of Amendment 2 were required to also attend the 6-month post-boost visit after approval of Amendment 2.
f. Signing of the informed consent form (ICF) and/or assent needs to be done before the first study-related activity. After reading but before signing the ICF/assent, the TOU will be administered.
g. The investigators should ensure that all study enrollment criteria have been met at the end of the screening phase. Minimum criteria for the availability of documentation supporting the eligibility criteria are described in Section 17.4.
h. Prestudy therapies up to 30 days prior to the start of screening and previous vaccinia/smallpox vaccination at any time prior to study entry must be recorded in the case report form (CRF). HIV-therapy should be recorded for HIV-infected subjects.
i. Only for women of childbearing potential.
j. Only for women >45 years of age with amenorrhea for less than 2 years or ≤45 years of age with amenorrhea for more than 6 months.
k. If a subject's clinical status changes (including available laboratory results or the receipt of additional medical records) after screening so the subject no longer meets the eligibility criteria, the subject should be excluded from further participation in the study.
l. After the prime and boost vaccinations, subjects will remain at the site for a total of 60 (±15) minutes post-vaccination to monitor for the development of any acute reactions, or longer if deemed necessary by the investigator. Solicited and unsolicited adverse events emerging during the observation period at the site will be recorded in the CRF.
m. Prior to study vaccine administration.
n. A single, 12-lead ECG (supine) after 5 minutes of rest will be performed for subjects ≥18 years of age (Cohorts 1 and 2a) at screening and interpreted locally. Additional ECG monitoring may be performed at other time points during the study if clinically indicated. If blood sampling or vital sign measurements are scheduled at the same time point as the ECG recording, the procedures should preferably be performed in the following order: vital signs, ECG, blood draw.
o. A full physical examination, including height and body weight, will be carried out at screening. A genitourinary examination is not required. At other visits, an abbreviated, symptom-directed examination will be performed as indicated by the investigator.
p. Includes blood pressure, pulse/heart rate (at rest) and body temperature.

q. Subjects will use the subject diary to document solicited local and systemic adverse events (reactogenicity) in the evening after prime and boost vaccination and then daily for the next 7 days at approximately the same time each day. Diaries should be completed at home by the subject. For illiterate subjects or those having difficulty completing the diary independently, the sites will make arrangements to have the diary completed according to their local practice.
r. Pregnancies will be reported from signing of the ICF until the end of the study.
s. For reporting of immediate reportable events, refer to Section 12.3.3.
t. Day 15 visit only for subjects in Groups 2 and 3 from Cohort 1.
u. Concomitant therapies must be recorded up to 42 days after the last vaccination. Thereafter, concomitant therapies should only be recorded if given in conjunction with serious adverse events and immediate reportable events.
v. Approximate blood volume expected to be drawn per visit for clinical laboratory assessments: 10 mL for Cohorts 1, 2a and 2b.
w. Only for HIV-infected subjects.
x. Blood volumes for immunogenicity assessments are indicated in Table 7.
y. Immunogenicity assessments on the 7-day post-boost visit will only be performed for Cohorts 1 and 2a, not for Cohort 2b.
z. PBMC samples will be obtained from subjects at selected sites with the capabilities to process PBMC samples (targeted at 165 subjects in Cohort 1 and 33 subjects in each of the other cohorts). Approximate blood volume for cellular immune response assessments: 40 mL for Cohorts 1 and 2a, but only 20 mL for Cohort 2b.
aa. The approximate blood volume excludes repeat or unscheduled samples taken for safety reasons or for technical issues with the samples.
bb. Values in square brackets indicate the approximate or cumulative blood volume without PBMC samples.

Pediatric Cohort 3 (Groups 1-2)

**Group**	**Screening Phase[a] (≤8 weeks)**	**Vaccination Phase and Post-vaccination Phase**								**Long-term Follow-up Phase[b]**	
**Group 1**		**D1**	**D2-D7**	**D8**	**D29**	**D30-D35**	**D36**	**D50**	**D71[c]**	**D209**	**D365**
**Group 2**					**D57**	**D58-D63**	**D64**	**D78**	**D99[c]**	**D237**	
**Study procedures**		**Prime**		+7d pp	**Boost**		+7d pb	+21d pb	+42d pb	+6m pb[d]	+1y pp
Screening/Administrative											
Informed consent/assent[e]	X										
Inclusion/exclusion criteria	X[f]										
Medical history and demographics	X										
Prestudy therapies[g]	X										
Serum pregnancy test[h]	X										
HIV 1/2 serology	X										
Test of Understanding (TOU)[e,i]	X										
Prevaccination symptoms		X									
Check clinical status + available data		X[j]									
Randomization		X									
Study Vaccine Administration[k]		▲			▼						
Safety Assessments											
Urine Pregnancy test[h]		X[l]			X[l]						
Body length/height and weight	X				X						
Physical examination[m]	X	X[l]		X	X[l]		X	X	X	X	X
Vital signs[n]	X	X[l]		X	X[l]		X	X			
Distribution of subject diary		X			X						
Completion of diary at home[o]			X			X					
Review of diary by site staff				X			X				
Adverse events[p]	Continuous										
Serious adverse events and immediate reportable events[q]	Continuous										
Concomitant medications	X	X	X	X	X	X	X	X	X	X[r]	X[r]
Clinical Laboratory Assessments											
Chemistry, hematology (2 to 6 mL)[s]	X	X[l]		X	X[l]		X				
Urinalysis	X										

**Group**	**Screening Phase**[a] **(≤8 weeks)**	**Vaccination Phase and Post-vaccination Phase**								**Long-term Follow-up Phase**[b]	
**Group 1**		**D1**	**D2-D7**	**D8**	**D29**	**D30-D35**	**D36**	**D50**	**D71**[c]	**D209**	**D365**
**Group 2**					**D57**	**D58-D63**	**D64**	**D78**	**D99**[c]	**D237**	
**Study procedures**		**Prime**		+7d pp	**Boost**		+7d pb	+21d pb	+42d pb	+6m pb[d]	+1y pp
Immunogenicity Assessments[t]											
Blood sampling (serum) for assessment of humoral immune responses (2.2 to 5 mL)[t]		X[l]			X[l]			X		X	X
Blood sampling (PBMC) for assessment of cellular immune responses (3 to 10 mL)[u]		X[l]						X		X	X
Approximate Blood Volumes[v] with [without] PBMC (for Immunogenicity Analyses)											
- By visit (mL)[w]<br>Cohort 3 (9- to 11-year old)<br>Cohort 3 (6- to 8-year old)<br>Cohort 3 (4- to 5-year old)		 15 [5]<br>8.5 [2.5]<br>6.5 [2.5]			 5<br>2.5<br>2.5			 15 [5]<br>8.5 [2.5]<br>6.5 [2.5]		 15 [5]<br>8.5 [2.5]<br>6.5 [2.5]	 15 [5]<br>8.5 [2.5]<br>6.5 [2.5]
- Cumulative (mL)[w]<br>Cohort 3 (9- to 11-year old)<br>Cohort 3 (6- to 8-year old)<br>Cohort 3 (4- to 5-year old)		 15 [5]<br>8.5 [2.5]<br>6.5 [2.5]			 20 [10]<br>11 [5]<br>9 [5]			 35 [15]<br>19.5 [7.5]<br>15.5 [7.5]		 50 [20]<br>28 [10]<br>22 [10]	 65 [25]<br>36.5 [12.5]<br>28.5 [12.5]

▲ *Ad26.ZEBOV 5x10^{10} vp or placebo* ▼ *MVA-BN-Filo 1x10^{8} Inf U or placebo*
d pp: days post-prime; d pb: days post-boost; m pb: months post-boost; y pp: year post-prime

NOTE: In case of early withdrawal from the study, early withdrawal assessments (ie, physical examination and immunogenicity assessments for serum and PBMCs) should be obtained. Subjects who wish to withdraw consent will be offered an optional visit for safety follow-up (before the formal withdrawal of consent); the subject has the right to refuse.

a. Screening may be split into multiple days or visits. Retesting of values (eg, safety laboratory) that lead to exclusion is allowed once using an unscheduled visit during the screening period, provided there is an alternative explanation for the out of range value. The safety laboratory assessments at screening are to be performed within 28 days prior to the prime vaccination (including Day 1 before vaccination) and may be repeated if they fall outside this time window. If rescreening is required, all screening procedures (except TOU) should be repeated.
b. For each cohort, all subjects will be followed up in a blinded manner by the site for the collection of serious adverse event information, immediate reportable events, and for blood draws for immunogenicity assessments until the last subject in that cohort has completed the study. Any subject who completed either the 6-month post-prime and/or the Day 365 visit prior approval of Amendment 2 was required to attend the 6-month post-boost and the Day 365 visit after approval of Amendment 2.
c. In addition to the assessments scheduled for the 42-day post-boost visit, subjects will be instructed to contact the investigator before the next visit if they experience any adverse event or intercurrent illness that they perceive as relevant and/or can be possibly related to study vaccine in their opinion. All subjects within a cohort will be followed in a blinded manner by the site until the last subject in that cohort has completed the study. Any subject who completed either

the 6-month post-prime and/or the Day 365 visit prior approval of Amendment 2 was required to attend the 6-month post-boost and the Day 365 visit after approval of Amendment 2.

d. Subjects who completed the 6-month post-prime visit (which has been replaced with the 6-month post-boost visit per protocol Amendment 2) prior to approval of Amendment 2 were required to also attend the 6-month post-boost visit after approval of Amendment 2.
e. Signing of the informed consent form (ICF) and/or assent needs to be done before the first study-related activity. After reading but before signing the ICF/assent, the TOU will be administered.
f. The investigators should ensure that all study enrollment criteria have been met at the end of the screening phase. Minimum criteria for the availability of documentation supporting the eligibility criteria are described in Section 17.4.
g. Prestudy therapies up to 30 days prior to the start of screening must be recorded in the case report form (CRF).
h. Only for female subjects of childbearing potential.
i. Test of Understanding (TOU) should be administered to the parent or guardian who gave consent, and to older children who provided assent as per local regulations and practice.
j. If a subject's clinical status changes (including available laboratory results or the receipt of additional medical records) after screening so the subject no longer meets the eligibility criteria, the subject should be excluded from further participation in the study.
k. After each vaccination, subjects will remain at the site for a total of 60 (±15) minutes post-vaccination to monitor for the development of any acute reactions, or longer if deemed necessary by the investigator. Solicited and unsolicited adverse events emerging during the observation period at the site will be recorded in the CRF.
l. Prior to study vaccine administration.
m. A full physical examination, including height and body weight, will be carried out at screening. A genitourinary examination is not required. At other visits, an abbreviated, symptom-directed examination will be performed as indicated by the investigator.
n. Includes blood pressure, pulse/heart rate (at rest) and body temperature.
o. Subjects will use the subject diary to document solicited local and systemic adverse events (reactogenicity) in the evening after each vaccination and then daily for the next 7 days at approximately the same time each day. Diaries should be completed at home by the subject (or parent, legal guardian or caregiver, as applicable). For illiterate subjects or those having difficulty completing the diary independently, the sites will make arrangements to have the diary completed according to their local practice.
p. Pregnancies will be reported from signing of the ICF until the end of the study.
q. For reporting of immediate reportable events, refer to Section 12.3.3.
r. Concomitant therapies must be recorded up to 42 days after the last vaccination. Thereafter, concomitant therapies should only be recorded if given in conjunction with serious adverse events and immediate reportable events.
s. Approximate blood volumes for clinical laboratory assessments as per site Standard Operating Procedure.
t. Blood volumes for immunogenicity assessments are indicated in Table 7.
u. PBMC samples will be obtained from subjects at selected sites with the capabilities to process PBMC samples (targeted at 165 subjects in Cohort 1 and 33 subjects in each of the other cohorts).
v. The approximate blood volumes exclude safety blood testing, repeat or unscheduled samples taken for safety reasons or for technical issues with the samples.
w. Values in square brackets indicate the approximate or cumulative blood volume without PBMC samples.

ADDITIONAL TIME AND EVENTS SCHEDULE

Subjects Who Do Not Receive a Boost Vaccination Because of a Study Pause

Group	Screening Phase[a] (≤8 weeks)	Vaccination Phase and Post-vaccination Phase				Long-term Follow-up Phase[b]			
Group 1		D1	D2-D7	D8[c]	-	D90	D180	D270	D365
Group 2					D15[c]				
Group 3					D15[c]				
**Study procedures**		**Prime**		+7d pp	+14d pp	+3m pp[d]	+6m pp	+9m pp[d]	+1y pp
Screening/Administrative									
Informed consent/assent[e]	X								
Inclusion/exclusion criteria	X[f]								
Medical history and demographics	X								
Prestudy therapies[g]	X								
Serum pregnancy test[h]	X								
HIV-1/2 serology (Cohorts 1 and 2b only)	X								
Follicle-stimulating hormone (FSH)[i]	X								
Test of Understanding (TOU)[e]	X								
Check clinical status + available data		X[j]							
Randomization		X							
Study Vaccine Administration[k]		▲							
Safety Assessments									
Urine Pregnancy test[h]		X[l]							
Electrocardiogram (ECG)[m]	X								
Physical examination[n]	X	X[l]		X		X[o]	X	X[o]	X
Vital signs[p]	X	X[l]				X[o]		X[o]	
Distribution of subject diary		X							
Completion of diary at home[q]			X						
Review of diary by site staff				X					
Adverse events[r]	Continuous								
Serious adverse events and immediate reportable events[s]	Continuous								
Concomitant medications	X	X	X	X	X[t]	X[u]	X[u]	X[u]	X[u]

Group	Screening Phase[a] (≤8 weeks)	Vaccination Phase and Post-vaccination Phase				Long-term Follow-up Phase[b]			
Group 1		D1	D2-D7	D8[c]	-	D90	D180	D270	D365
Group 2					D15[c]				
Group 3					D15[c]				
**Study procedures**		**Prime**		+7d pp	+14d pp	+3m pp[d]	+6m pp	+9m pp[d]	+1y pp
Clinical Laboratory Assessments									
Chemistry, hematology (10 mL)[v]	X	X[l]		X					
Urinalysis	X								
Immunogenicity Assessments[w]									
Blood sampling (serum) for assessment of humoral immune responses (10 mL)		X[l]			X[t]		X		X
Blood sampling (PBMC) for assessment of cellular immune responses (40 mL)[x]		X[l]			X[t]		X		X
Approximate Blood Volumes[y] with [without] PBMC									
- By visit (mL)[z]									
Cohort 1 (Group 1)	10	60 [20]		10	0		50 [10]		50 [10]
Cohort 1 (Groups 2 and 3)	10	60 [20]		10	50[t] [10]		50 [10]		50 [10]
- Cumulative (mL)[z]									
Cohort 1 (Group 1)	10	70 [30]		80 [40]	80 [40]		130 [50]		180 [60]
Cohort 1 (Groups 2 and 3)	10	70 [30]		80 [40]	130[t] [50]		180 [60]		230 [70]

▲ *Ad26.ZEBOV 5x10^{10} vp or placebo* ▼ *MVA-BN-Filo 1x10^{8} Inf U or placebo*
d pp: days post-prime; m pp: months post-prime; y pp: year post-prime

NOTE: In case of early withdrawal from the study, early withdrawal assessments (ie, physical examination and immunogenicity assessments for serum and PBMCs) should be obtained. Subjects who wish to withdraw consent will be offered an optional visit for safety follow-up (before the formal withdrawal of consent); the subject has the right to refuse.

a. Screening may be split into multiple days or visits. Retesting of values (eg, safety laboratory) that lead to exclusion is allowed once using an unscheduled visit during the screening phase. The safety laboratory assessments at screening are to be performed within 28 days prior to the prime vaccination (including Day 1 before vaccination) and may be repeated if they fall outside this time window.
b. All subjects will be followed up in a blinded manner by the site for the collection of serious adverse event information, immediate reportable events, and for blood draws for immunogenicity assessments until Day 365.
c. In addition to the assessments scheduled for the 7-day post-prime visit (or 14-day post-prime visit for subjects in Group 2 and 3 of Cohort 1), subjects will be instructed to contact the investigator before the next visit if they experience any adverse event or intercurrent illness that they perceive as relevant and/or can be possibly related to study vaccine in their opinion.
d. Information for these visits may be collected either by telephone contact, visit to the site or by a home visit, according to local practice.

e. Signing of the informed consent form (ICF) and/or assent needs to be done before the first study-related activity. After reading but before signing the ICF/assent, the TOU will be administered.
f. The investigators should ensure that all study enrollment criteria have been met at the end of the screening phase. Minimum criteria for the availability of documentation supporting the eligibility criteria are described in Section 17.4.
g. Prestudy therapies up to 30 days prior to the start of screening and previous vaccinia/smallpox vaccination at any time prior to study entry must be recorded in the case report form (CRF).
h. Only for women of childbearing potential.
i. Only for women >45 years of age with amenorrhea for less than 2 years or ≤45 years of age with amenorrhea for more than 6 months.
j. If a subject's clinical status changes (including available laboratory results or the receipt of additional medical records) after screening so the subject no longer meets the eligibility criteria, the subject should be excluded from further participation in the study.
k. After each vaccination, subjects will remain at the site for a total of 60 (±15) minutes post-vaccination to monitor for the development of any acute reactions, or longer if deemed necessary by the investigator. Solicited and unsolicited adverse events emerging during the observation period at the site will be recorded in the CRF.
l. Prior to study vaccine administration.
m. A single, 12-lead ECG (supine) after 5 minutes of rest will be performed for subjects ≥18 years of age at screening and interpreted locally. Additional ECG monitoring may be performed at other time points during the study if clinically indicated. If blood sampling or vital sign measurements are scheduled at the same time point as the ECG recording, the procedures should preferably be performed in the following order: vital signs, ECG, blood draw.
n. A full physical examination, including height and body weight, will be carried out at screening. A genitourinary examination is not required. At other visits, an abbreviated, symptom-directed examination will be performed as indicated by the investigator.
o. Physical examination and vital signs assessments are optional at these visits.
p. Includes blood pressure, pulse/heart rate (at rest) and body temperature.
q. Subjects will use the subject diary to document solicited local and systemic adverse events (reactogenicity) in the evening after each vaccination and then daily for the next 7 days at approximately the same time each day. Diaries should be completed at home by the subject. For illiterate subjects or those having difficulty completing the diary independently, the sites will make arrangements to have the diary completed according to their local practice.
r. Non-serious adverse events will be reported until the 42-day post-last vaccination visit. Pregnancies will be reported from signing of the ICF until the end of the study.
s. For reporting of immediate reportable events, refer to Section 12.3.3.
t. Day 15 visit only for subjects in Groups 2 and 3 from Cohort 1.
u. Concomitant therapies must be recorded up to 42 days after the last vaccination. Thereafter, concomitant therapies should only be recorded if given in conjunction with serious adverse events and immediate reportable events.
v. Approximate blood volume expected to be drawn per visit for clinical laboratory assessments: 10 mL for Cohort 1.
w. Blood volumes for immunogenicity assessments are indicated in Table 7.
x. PBMC samples will be obtained from subjects at selected sites with the capabilities to process PBMC samples (targeted at 165 subjects in Cohort 1). Approximate blood volume for cellular immune response assessments: 40 mL for Cohort 1.
y. The approximate blood volume excludes repeat or unscheduled samples taken for safety reasons or for technical issues with the samples.
z. Values in square brackets indicate the approximate or cumulative blood volume without PBMC samples.

COHORT 1 SUBSTUDY TIME AND EVENTS SCHEDULE

Subjects at Selected Sites Receiving a Third Vaccination (Cohort 1, Groups 1-2)

	Vaccination Phase and Post-vaccination Phase					Long-term Follow-up Phase[a]	
**Groups 1 & 2[b]**	**1 Year Post Prime + 3 months**	**D2-D7 Post 3rd Vaccination**	**D5 Post 3rd Vaccination**	**D8 Post 3rd Vaccination**	**D22 Post 3rd Vaccination**	**D180 Post 3rd Vaccination**	**D365 Post 3rd Vaccination**
**Study procedures**	**3rd Vaccination**		+4d p3v	+7d p3v	+21d p3v	+6m p3v	+1y p3v
Screening/Administrative							
Informed consent[c]	X						
Study vaccine administration	▲[d]						
Safety assessments							
Urine pregnancy test[e]	X[f]						
Physical examination[g]	X[f]		X	X	X	X[h]	X[h]
Vital signs[i]	X[f]						
Distribution of subject diary	X						
Completion of diary at home[j]		X					
Review of diary by site staff				X			
Adverse events[k]	Continuous until 28 days after the third vaccination[a,l]						
Serious adverse events and immediate reportable events [m]	Continuous[a,l]						
Concomitant medications	X		X	X	X	X[n]	X[n]
Clinical Laboratory Assessments							
Chemistry, hematology (10 mL)[o]	X[f]			X			
Immunogenicity Assessments[p]							
Blood sampling (serum) for assessment of humoral immune responses (10 mL)	X[f]		X	X	X	X	X
Blood sampling (PBMC) for assessment of cellular immune responses (40 mL)[q]	X[f]				X		X
Approximate Blood Volumes with [without] PBMC[r]							
- By visit (mL)[r] Cohort 1 (Group 1 and 2)	60 [20]		10	20	50 [10]	10	50 [10]
- Cumulative (mL)[r] Cohort 1 (Group 1 and 2)	60 [20]		70 [30]	90 [50]	140 [60]	150 [70]	200 [80]

▲ *Ad26.ZEBOV 5x10^{10} vp or placebo*

d p3v: days post third vaccination; m p3v: months post third vaccination; y p3v: year post third vaccination

NOTE: In case of early withdrawal from the study, early withdrawal assessments (ie, physical examination and immunogenicity assessments for serum) should be obtained. Subjects who wish to withdraw consent will be offered an optional visit for safety follow-up (before the formal withdrawal of consent); the subject has the right to refuse.

NOTE: The day of administration of the third vaccination is Day 1 of the Cohort 1 substudy. Similarly, D2 Post 3rd vaccination is the second day of the Cohort 1 substudy, ie, 1 day after the third vaccination.

a. All subjects will be followed up in a blinded manner by the site for the collection of serious adverse event information, immediate reportable events, and for blood draws for immunogenicity assessments until the last subject in that cohort has completed the study.
b. Subjects eligible for the third vaccination are members of Cohort 1 (Groups 1 or 2) who consented to participate in the substudy. Approximately 90 subjects from Cohort 1 will be recruited at selected sites. For subjects who receive the third vaccination, the timings of the next post third vaccination visits will be determined relative to the actual day of the third vaccination.
c. Informed consent form to enter in the Cohort 1 substudy must be provided and signed before the first substudy-related activity.
d. After the third vaccination, subjects will remain at the site for a total of 30 (±15) minutes post-vaccination to monitor for the development of any acute reactions, or longer if deemed necessary by the investigator. Solicited and unsolicited adverse events emerging during the observation period at the site will be recorded in the case report form (CRF).
e. Only for women of childbearing potential.
f. Prior to study vaccine administration.
g. An abbreviated, symptom-directed examination will be performed as indicated by the investigator.
h. During the long-term follow-up, a physical examination should only be performed when related to serious adverse events and immediate reportable events.
i. Includes blood pressure, pulse/heart rate (at rest) and body temperature.
j. Subjects will use the subject diary to document solicited local and systemic adverse events (reactogenicity) in the evening after the third vaccination and then daily for the next 7 days at approximately the same time each day. Diaries should be completed at home by the subject. For illiterate subjects or those having difficulty completing the diary independently, the sites will make arrangements to have the diary completed according to their local practice.
k. Non-serious adverse events will be reported until 28 days after the third vaccination. Pregnancies will be reported from signing of the informed consent form (ICF) until the end of the study.
l. Subjects will be instructed to contact the investigator before the next visit if they experience any adverse event or intercurrent illness that they perceive as relevant and/or can be possibly related to study vaccine in their opinion. All subjects within a cohort will be followed in a blinded manner by the site until the last subject in that cohort has completed the study.
m. For reporting of immediate reportable events, refer to Section 12.3.3.
n. Concomitant therapies must be recorded up to 28 days after the third vaccination. Thereafter, they should only be recorded if given in conjunction with serious adverse events and immediate reportable events.
o. Approximate blood volume expected to be drawn per visit for clinical laboratory assessments: 10 mL.
p. Blood volumes for immunogenicity assessments are indicated in Table 7.
q. These samples are optional and may only be collected in a subset of subjects in the substudy.
r. The approximate blood volume excludes repeat or unscheduled samples taken for safety reasons or for technical issues with the samples.

ABBREVIATIONS

Adxx	adenovirus serotype xx (vector)
Ad26.ZEBOV	adenovirus serotype 26 expressing the Ebola virus Mayinga glycoprotein
AIDS	acquired immunodeficiency syndrome
ARV	antiretroviral agent
β-hCG	β-human chorionic gonadotropin
BMI	body mass index
BN	Bavarian Nordic
CDC	Centers for Disease Control and Prevention
CRF	case report form
DMID	Division of Microbiology and Infectious Diseases
DNA	deoxyribonucleic acid
EBOV	Ebola virus
ECG	electrocardiogram
eDC	electronic data capture
EDTA	ethylenediaminetetraacetic acid
ELISA	enzyme-linked immunosorbent assay
ELISpot	enzyme-linked immunospot
EU	European Union
FSH	follicle stimulating hormone
GCP	Good Clinical Practice
GMP	Good Manufacturing Practice
GP	glycoprotein
HAART	Highly Active Antiretroviral Therapy
HIV	human immunodeficiency virus
ICF	informed consent form
ICH	International Council for Harmonisation
ICS	intracellular cytokine staining
IDMC	Independent Data Monitoring Committee
IEC	Independent Ethics Committee
IFN-γ	interferon-gamma
Ig	immunoglobulin
IL	interleukin
IM	intramuscular(ly)
IMI	Innovative Medicines Initiative
Inf U	infectious units
INSERM	Institut National de la Santé et de la Recherche Médicale
IRB	Institutional Review Board
IRE	immediate reportable event
IWRS	interactive web response system
kb	kilobase
MARV	Marburg virus
MedDRA	Medical Dictionary for Regulatory Activities
MVA	Modified Vaccinia Ankara
MVA-BN-Filo	Modified Vaccinia Ankara Bavarian Nordic vector expressing multiple filovirus proteins
NHP	nonhuman primate
NP	nucleoprotein
PBMC	peripheral blood mononuclear cell
PQC	Product Quality Complaint
RNA	ribonucleic acid
SAP	Statistical Analysis Plan
SUDV	Sudan virus
SUSAR	suspected unexpected serious adverse reaction
TAFV	Tai Forest virus
$TCID_{50}$	50% tissue culture infective dose
THAM	tris (hydroxymethyl)-amino methane
TNF-α	tumor necrosis factor-α

TOU	test of understanding
US	United States
VISP	vaccine-induced seropositivity
vp	viral particles
WHO	World Health Organization

DEFINITIONS OF TERMS

Study vaccine	Ad26.ZEBOV, MVA-BN-Filo or placebo.
Blinded study vaccine administrator	A blinded trained study nurse, medical doctor, or otherwise qualified health care provider.
Independent study vaccine monitor	An unblinded study vaccine monitor assigned to the study who is responsible for the unblinded interface between the sponsor and the investigational site pharmacy.
Solicited adverse events (reactogenicity)	Local and systemic adverse events that are common and known to occur after vaccination and that are usually collected in a standard, systematic format in vaccine clinical studies. For the list of solicited adverse events in this study, see Section 9.3. For the purpose of vaccine clinical studies, all other adverse events are considered unsolicited; however, this definition should be distinguished from definitions based on pharmacovigilance guidelines.
End of the study (for a subject)	For subjects in the Cohort 1 substudy: after completion of all assessments at the 1-year post third vaccination visit.<br>For all other subjects: after completion of all assessments at the 6-month post-boost vaccination visit or at the Day 365 visit, whichever occurs later.

1. INTRODUCTION

Janssen Vaccines & Prevention B.V. (formerly known as Crucell Holland B.V.) (hereafter referred to as the sponsor), in collaboration with Bavarian Nordic (BN), and in conjunction with an Innovative Medicines Initiative (IMI) consortium led by the Institut National de la Santé et de la Recherche Médicale (INSERM), is investigating the potential of a prophylactic Ebola vaccine regimen comprised of the following 2 candidate Ebola vaccines:

Ad26.ZEBOV is a monovalent vaccine expressing the full length Ebola virus (EBOV, formerly known as *Zaire ebolavirus*) Mayinga glycoprotein (GP), and is produced in the human cell line PER.C6.

MVA-mBN226B, further referred to as Modified Vaccinia Ankara (MVA)-BN-Filo, is a multivalent vaccine expressing the Sudan virus (SUDV) GP, the EBOV GP, the Marburg virus (MARV) Musoke GP, and the Tai Forest virus (TAFV, formerly known as *Côte d'Ivoire ebolavirus*) nucleoprotein (NP), and is produced in chicken embryo fibroblast cells. The EBOV GP expressed by MVA-BN-Filo has 100% homology to the one expressed by Ad26.ZEBOV.

For the most up-to-date nonclinical and clinical information regarding Ad26.ZEBOV and MVA-BN-Filo, refer to the latest versions of the Investigator's Brochures and Addenda (if applicable).[14,15] A brief summary of the nonclinical and clinical information is provided below.

The term "sponsor" used throughout this document refers to the entities listed in the Contact Information page(s), which will be provided as a separate document.

1.1. Background

Ebola viruses belong to the Filoviridae family and cause Ebola virus disease, which can induce severe hemorrhagic fever in humans and nonhuman primates (NHPs). Case fatality rates in Ebola disease range from 25% to 90% (average: 50%), according to the World Health Organization (WHO).[27] These viruses are highly prioritized by the United States (US) Government, who has defined them as 'Category A' agents, due to the high mortality rate of infected individuals. Currently, no licensed vaccine, treatment or cure exists for this disease.

Filoviruses are named for their long, filamentous shape. Within this filamentous virus, a single 19-kilobase (kb) negative-sense ribonucleic acid (RNA) genome encodes 7 proteins: the GP, the polymerase, the NP, the secondary matrix protein, the transcriptional activator, the polymerase cofactor, and the matrix protein. The virion surface is covered by homotrimers of the viral GP, which is believed to be the sole host attachment factor for filoviruses. Following cell entry, the viruses replicate their genomes and viral proteins in the cytoplasm using an RNA-dependent RNA polymerase, which is carried into the cell together with the virus.[11]

1.1.1. Nonclinical Studies

Immunogenicity and Efficacy

Immunogenicity and efficacy of the vaccine combination Ad26.ZEBOV and MVA-BN-Filo was evaluated in an NHP model (ie, Cynomolgus macaques, *Macaca fascicularis*). The combination was assessed in a multivalent filovirus setting in a small number (2 per regimen) of animals and the study included heterologous prime-boost regimens of adenovirus serotype 26 (Ad26), Ad35 and MVA-BN-Filo vectors expressing different Ebola and Marburg proteins. Full protection from Ebola virus disease and death after wild-type EBOV Kikwit 1995 challenge was obtained with all heterologous regimens, including the Ad26 and MVA vaccine regimen. All heterologous prime-boost regimens induced comparable immune responses against the EBOV Mayinga GP. Independently of the vaccine regimen, a strong boost effect was seen after heterologous prime-boost immunization. Two additional studies involving more animals are ongoing, to strengthen the robustness of the nonclinical efficacy data, and also to optimize the prime-boost schedule so as to obtain induction of protective immunity as quickly as possible, to specifically respond to the Ebola virus disease outbreak in West Africa.

Toxicology

A repeated-dose toxicity study in rabbits was performed with prime-boost combinations of Ad26.ZEBOV and MVA-BN-Filo. The different dose regimens were well tolerated when administered twice by intramuscular (IM) injection to New Zealand White rabbits with a 14-day interval period. Additionally, the objective was to assess the persistence, reversibility or delayed onset of any effects after a 14-day treatment-free period. In the heterologous prime-boost regimen, either vector or both were used to prime a filovirus-specific immune response and the other/same vector or both were used to boost the immune response 2 weeks later. All vaccine dosing regimens resulted in detectable EBOV GP-specific antibody titers. No significant toxicological effects (no adverse effects) were observed. The immune response was associated with transient increases in fibrinogen, C-reactive protein, globulin, decreases in hematocrit and hemoglobin, and microscopic findings in draining iliac lymph nodes, spleen and at the injection sites. The findings were noted to be recovering over a 2-week treatment-free period and were considered to reflect a physiological response associated with vaccination. There were no effects noted that were considered to be adverse.

Biodistribution

Single-dose biodistribution studies in rabbits were performed using the MVA-BN vector or the Ad26 vector in combination with another insert (Ad26.ENVA.01: an experimental, prophylactic Ad26 vector expressing the human immunodeficiency virus [HIV] type 1, Clade A envelope protein). MVA-BN distributed to the skin, muscle, blood, spleen, lung, liver, and pooled lymph nodes and was rapidly cleared (within 48 hours following vaccination). Ad26.ENVA.01 was primarily localized in the injection site muscle, the regional lymph nodes and the spleen. Three months after the single IM injection of Ad26.ENVA.01, the vaccine was cleared from most of the examined tissues. As biodistribution is dependent on the vector platform (MVA or Ad26) and not on the insert, it can be assumed that recombinant MVA-BN-Filo or Ad26.ZEBOV is distributed in the same way as the MVA-BN vector or Ad26.ENVA.01 vector, respectively.

1.1.2. Clinical Studies

1.1.2.1 Safety Profile of Ad26-based Vaccines

To date, no human clinical studies have been completed with Ad26.ZEBOV or MVA-BN-Filo. The safety/tolerability and immunogenicity of the Ad26.ZEBOV and MVA-BN-Filo vaccines are being assessed in the ongoing Phase 1 studies (VAC52150EBL1001 and VAC52150EBL1002), where monovalent Ad26.ZEBOV and multivalent MVA-BN-Filo are combined in homologous or heterologous prime-boost regimens in which each vector is used to prime a filovirus-specific immune response followed by a boost immunization with the same or the other vector 2 to 8 weeks later. In addition, 2 other Phase 1 studies are planned to be conducted in Africa (VAC52150EBL1003 and VAC52150EBL1004). Two additional Phase 1 studies investigating MVA-BN-Filo are also ongoing (EBL01 and CVD-Mali Ebola Vaccine #1000). Refer to the latest versions of the Ad26.ZEBOV and MVA-BN-Filo Investigator's Brochures and Addenda (if applicable) for more details.[14,15]

Limited data from the ongoing Phase 1 studies with Ad26.ZEBOV and MVA-BN-Filo are available.

VAC52150EBL1001, a first-in-human study, has enrolled 87 subjects for which 7-day post-prime safety data, blinded on a treatment group level, on 72 subjects (36 per treatment group) are available. Most of the adverse events reported were grade 1 or grade 2 in severity. Local injection site reactions were reported in 18 (50%) MVA/placebo subjects (all grade 1) and 28 (78%) Ad26/placebo subjects (grade 1 [22], grade 2 [5], grade 3 [1]). The most frequent local reaction was injection site pain, in 17 (47%) MVA/placebo subjects and 28 (78%) Ad26/placebo subjects, with one grade 3 case occurring in the Ad26/placebo group. Solicited systemic reactions were reported in 25 (69%) MVA/placebo subjects (grade 1 [24] and grade 2 [1]) and 31 (86%) Ad26/placebo subjects (grade 1 [22], grade 2 [7], grade 3 [1], unknown [1]). The most frequent systemic reactions were fatigue (50% overall), followed by headache (46%) and myalgia (35%). One Ad26/placebo subject experienced 3 grade 3 solicited systemic reactions (headache, myalgia and nausea). None of the subjects reported fever; however, 2 subjects had one temperature measurement missing. The most frequent unsolicited adverse events were decreased neutrophils, in 3 (8%) MVA/placebo subjects and 6 (17%) Ad26/placebo subjects, followed by activated partial thromboplastin time prolongation and hypokalemia, in 3 (8%) MVA/placebo subjects and 5 (14%) Ad26/placebo subjects each. All of these events were transient in nature and resolved without intervention. No deaths or serious adverse events and no adverse events leading to discontinuation of the study vaccination were reported.

Study VAC52150EBL1002 completed enrollment of 92 subjects; the blinded phase of the study is ongoing. No serious adverse events related to study vaccine have been reported and no safety issues have been identified to date.

Safety data generated with the 2 backbones containing different inserts are provided below.

Safety Data From Other Ad26-based Vaccine Programs

Ad26.ZEBOV is a monovalent, replication-incompetent Ad26-based vector. Only limited clinical data are available for Ad26.ZEBOV. However, adenovirus vaccine programs with other gene inserts revealed no significant safety issues. The data described below are based on the evaluation of the prototype vaccine Ad26.ENVA.01, which expresses the HIV envelope gene.[16]

Three randomized, placebo-controlled, Phase 1 studies (IPCAVD-001, IPCAVD-003, IPCAVD-004) have evaluated the safety and immunogenicity of the prototype vaccine Ad26.ENVA.01. This prototype vaccine has been administered to more than 200 healthy, HIV-negative subjects between the ages of 18 and 50 years in the United States and Africa.[16,17,18]

- In the dose-escalation study IPCAVD-001 (n=60), 2 or 3 IM doses of Ad26.ENVA.01 ($1x10^9$, $1x10^{10}$, $5x10^{10}$, $1x10^{11}$ viral particles [vp]) were given to Ad26 seronegative subjects. There were no deaths or vaccine-related serious adverse events. Ad26.ENVA.01 was generally well tolerated at all 4 dose levels with minimal reactogenicity observed in the $1x10^9$ and $1x10^{10}$ vp dose groups. Moderate to severe malaise, myalgia, fatigue and chills occurred in the majority of subjects 12 to 18 hours after the first dose of $1x10^{11}$ vp, but were resolved within 24 to 36 hours and were not seen after the second injection at this dose level. Two subjects in the $1x10^{11}$ vp dose group chose not to have the second injection, however, one of them decided to have the 6-month injection. Envelope-specific humoral and cell-mediated immune responses were induced at all 4 dose levels of vaccine.[2,4]
- In the single-dose study IPCAVD-003 (n=24), an IM dose of Ad26.ENVA.01 ($5x10^{10}$ vp) or placebo was given to subjects, who were stratified according to baseline Ad26 immune status, to evaluate the safety, mucosal immunogenicity and innate immune responses. Local reactogenicity comprised moderate injection site pain/tenderness and/or moderate to severe erythema which resolved within 3 days of vaccination. Transient systemic reactogenicity comprised headache, chills, joint pain, myalgia, malaise/fatigue, and fever. No deaths or vaccine-related serious adverse events were observed. Vaccination elicited both systemic and mucosal envelope-specific humoral and cellular immune responses. No increased activated total or vector-specific mucosal CD4+ T-lymphocytes following vaccination were detected in the colorectal mucosa, indicating that vaccination with Ad26 did not increase mucosal inflammation.[1]
- In study IPCAVD-004 (n=217), the safety and immunogenicity of IM doses of Ad26.ENVA.01 and Ad35.ENV (an Ad35 vector expressing an HIV envelope GP used in that study at a dose of $5x10^{10}$ vp), given in heterologous and homologous prime-boost regimens at 3- versus 6-month intervals, was evaluated. There were 452 adverse events reported by 84 of 176 Ad26-vaccine recipients (47.7%), the majority being mild (75.5%) in severity. The proportion of subjects with moderate or severe symptoms was not statistically significantly different between vaccine and placebo. There were 3 serious adverse events: 2 serious adverse events in placebo recipients (grade 3 peritonsillar abscess and grade 4 migraine headache, both resolved with no residual effects) and 1 serious adverse event in an Ad35/Ad26 vaccine recipient (grade 4 acute myelogenous leukemia, resolved with sequelae). No deaths or vaccine-related serious adverse events were reported. Overall, 97% to 100% of subjects developed anti-envelope binding antibodies (enzyme-linked immunosorbent assay [ELISA]) after a second dose, with heterologous and homologous regimens being comparable. Immune responses in groups who received 3- and 6-month

schedules were comparable. Four weeks post-vaccination, interferon (IFN)-γ enzyme-linked immunospot (ELISpot) assay showed response rates between 44% and 100%. The heterologous and homologous regimens were comparable. There was induction of Ad26-neutralizing antibodies in the majority of vaccine recipients after 2 immunizations with Ad26.ENVA.01.[20]

In addition, the sponsor performed a Phase 1/2a double-blind, randomized, placebo-controlled, dose-escalation study (MAL-V-A001) to evaluate the safety, tolerability and immunogenicity of 2 dose levels ($1x10^{10}$ and $5x10^{10}$ vp) of Ad35.CS.01/Ad26.CS.01 (both expressing the malaria *Plasmodium falciparum* circumsporozoite antigen) prime-boost regimens in healthy subjects. The dose-escalation phase was followed by an evaluation of efficacy of the higher dose level in an experimental malaria challenge. A total of 42 subjects were enrolled and were vaccinated. The analysis of adverse events did not show any consistent pattern suggestive of an association of Ad35.CS.01 or Ad26.CS.01 with specific adverse events. There were no serious adverse events reported during the study. No subject discontinued during a study phase (vaccination or challenge) due to adverse events. One subject in the high-dose group completed the vaccination phase and the final safety follow-up visit but did not take part in any challenge phase activities because of ongoing dyspnea. The most common related adverse events after each vaccination were injection site pain, malaise, headache, myalgia and chills. The incidence of vaccine-related adverse events was generally higher in the high-dose group than in the low-dose group. In general, incidence of malaise, headache, and myalgia were higher after the third dose (Ad26) than after the first or second doses (Ad35). Injection site pain was more commonly reported in the low and high-dose groups than by placebo subjects. There were no clinically significant changes in laboratory test parameters or vital signs data.[6]

Recent data indicate that administration of a deoxyribonucleic acid (DNA) vaccine expressing EBOV Mayinga GP, the same GP as in the Ad26.ZEBOV component, was safe, well tolerated and immunogenic in a Phase 1 clinical study. During this study, 9 subjects received three 4-weekly IM doses of vaccine (4 mg/dose), followed by a boost at ≥32 weeks in 8 subjects.[23]

Based on the previous clinical experience of Ad26 vector with different inserts, there has been no impact of Ad26 seropositivity on subjects' safety and only limited impact on immunogenicity results. Therefore, there are no safety concerns with regard to the inclusion of Ad26 seropositive subjects in the study, and the study subjects will not be screened for Ad26 seropositivity as part of the study eligibility criteria. The purpose of the Ad26 seropositivity assessment at baseline is to evaluate its impact, if any, on vaccine immunogenicity.

1.1.2.2 Safety Profile of MVA-BN-based Vaccines

MVA-BN is a further attenuated version of the MVA virus, which in itself is a highly attenuated strain of the poxvirus Chorioallantois Vaccinia Virus Ankara. MVA-BN induces strong cellular activity as well as a humoral (antibody) immune response and has demonstrated an ability to stimulate a response even in individuals with pre-existing immunity against Vaccinia. One of the advantages of MVA-BN is the virus' inability to replicate in a vaccinated individual. The replication cycle is blocked at a very late stage, which ensures that new viruses are not generated and released. This means that the virus cannot spread in the vaccinated person and none of the serious side effects normally associated with replicating Vaccinia viruses have been seen with MVA-BN.

MVA-BN (MVA-BN, trade name IMVAMUNE outside the European Union [EU], invented name IMVANEX in the EU) has received marketing authorization in the EU for active immunization against smallpox in adults, and in Canada for persons 18 years of age and older who have a contraindication to the first or second generation smallpox vaccines including individuals with immune deficiencies and skin disorders.[13] A Phase 3 clinical study (POX-MVA-013) has been completed (ClinicalTrials.gov Identifier: NCT01144637).[7] Results of completed and ongoing clinical studies of MVA-BN-based vaccines in more than 8,100 individuals, including elderly, children and immunocompromised subjects in whom replicating vaccines are contraindicated, have shown that the platform displays high immunogenicity and a favorable safety profile.[21] Across all clinical studies, no trends for unexpected or serious adverse reactions due to the product were detected.

Safety information was pooled from the first 2 studies of MVA-BN-Filo (VAC52150EBL1001 and VAC52150EBL1002). In general, MVA-BN-Filo has been shown to be well tolerated.[15]

Three fifths of the subjects reported at least one local site reaction (injection site pain, tenderness, warmth, redness, swelling and/or itching) following administration of MVA-BN-Filo; mostly of mild severity. The most commonly reported local site reaction was pain at the injection site. All the local reactions resolved to normal without any lasting effects.

At least one general symptom was reported in two fifths of the subjects following MVA-BN-Filo administration. The most common general symptoms were fatigue, headache, myalgia (muscle pain) and nausea. All general symptoms were transient and resolved without lasting effects.

Changes in laboratory tests were reported following MVA-BN-Filo administration which included hypokalemia and decreased numbers of neutrophils (neutropenia). Both changes in laboratory tests were seen in similar numbers of participants following MVA-BN-Filo and the dummy (placebo) vaccine. Less frequently, events of decreased hemoglobin levels were reported. The changes in laboratory tests were not associated with any complaints or symptoms.

Extensive nonclinical studies support the safety profile of the MVA-BN strain.[24,25]

1.1.2.3 Relevant Safety Information from Ongoing VAC52150 Studies

One subject in the study VAC52150EBL2001 experienced a serious and very rare condition called "Miller Fisher syndrome". This condition consists of double vision, pain on moving the eye, and difficulty with balance while walking. Miller Fisher syndrome most commonly occurs following a recent infection. The subject experienced these symptoms about a week after suffering from a common cold and fever. The event happened about a month after boost vaccination with either MVA-BN-Filo or placebo. This subject had to go to the hospital for treatment and has recovered. After an extensive investigation, the event has been considered to be doubtfully related to vaccine and most likely related to the previous common cold.

In the ongoing clinical studies with more than 2,000 participants, there have been a few reports of mild to moderate tingling especially in the hands and feet or a sensation of mild to moderate muscle weakness in subjects vaccinated with Ad26.ZEBOV or placebo. These symptoms have been observed to last no more than 24 to 48 hours in the majority of cases but can last for several weeks before going away on their own. These types of symptoms have also been reported following administration of other licensed vaccines and following acute viral infections of various types. One serious case of probable peripheral sensory neuropathy of moderate severity has occurred and has been ongoing for several months, interfering with some of the subject's daily activities.

1.1.2.4 Viral Shedding

Viral shedding information is available from 6 clinical studies with Ad-vectored vaccines against HIV type 1 (using Ad26 and Ad35: Ad26.ENVA.01 and Ad35.ENV) and *Mycobacterium tuberculosis* (using Ad35: AERAS-402). Viral shedding was not observed in any of these clinical studies. In a clinical study evaluating viral shedding of Ad26.ENVA.01 and Ad35.ENV (study IPCAVD-004), all cultures from oropharyngeal swabs and urine were negative for adenovirus; in 5 clinical studies evaluating viral shedding of AERAS-402 (studies C-001-402, C-003-402, C-008-402, C-009-402, C-017-402), no shedding of AERAS-402 was seen in any of the urine or throat cultures.[14]

MVA-BN-Filo is an attenuated recombinant MVA incapable of replication in human cells with a block in the late stage of virus replication. In human cells, upon infection, viral genes are expressed, but no infectious progeny virus is produced. Given the inability of virus assembly and very limited host range of the vector, no viral shedding studies were performed.

1.2. Benefit/Risk Section

1.2.1. Known Benefits

The clinical benefit of prime-boost combinations of Ad26.ZEBOV and MVA-BN-Filo is to be established.

1.2.2. Potential Benefits

Subjects may benefit from clinical testing and physical examination; others may benefit from the knowledge that they may aid in the development of an Ebola vaccine. There is no direct individual benefit from vaccination for the subjects at the current development stage.

1.2.3. Known Risks

To date, there are only limited data from the Phase 1 studies with Ad26.ZEBOV and MVA-BN-Filo available. However, Ad26- and Ad35-based vaccines with other gene inserts have been administered to a limited number of human volunteers in clinical studies. These other vaccines mainly elicited some solicited local and systemic reactions, as expected with injectable vaccines, and no serious safety concerns in study participants. MVA-BN-based vaccines have been administered to more than 8,100 individuals without unexpected or serious adverse reactions reported. For details, see the safety data presented in Section 1.1.

1.2.4. Potential Risks

The following potential risks for Ad26.ZEBOV and MVA-BN-Filo will be monitored during the study and are specified in the protocol:

Risks Related to Vaccines

Subjects may exhibit general signs and symptoms associated with administration of a vaccine, or a placebo vaccination, including nausea/vomiting, headache, myalgia, arthralgia, fever, fatigue/malaise and chills. In addition, subjects may experience local (injection site) reactions such as pain/tenderness, erythema, induration/swelling and itching at the injection site. These events will be monitored, but are generally short-term and do not require treatment.

Subjects may have an allergic reaction to the vaccination. An allergic reaction may cause a rash, hives or even difficulty in breathing (anaphylaxis). Severe reactions are rare. Medications must be available in the clinic to treat serious allergic reactions.

The risks related to vaccine-induced seropositivity (VISP) are discussed in Section 9.5.

Risk of Myo/Pericarditis

While replicating smallpox vaccines have been associated with an increased risk to develop myo/pericarditis,[22] this has not been observed with MVA-BN and is not expected with this highly attenuated, non-replicating vaccine. Based on observations with first- and second-generation replication-competent smallpox vaccines, particular attention has been placed on the monitoring for cardiac signs and symptoms in all clinical studies using MVA-BN. Despite the close cardiac monitoring, no event indicating a case of pericarditis has been observed in any

completed MVA-BN study. There has been 1 case of chest pain that might be indicative of pericarditis (consisting of chest pain only with no other cardiac findings suggestive of pericarditis) with previous MVA use although this diagnosis was not finally confirmed and the subject fully recovered. In a review of prospective surveillances for cardiac adverse events in 6 different clinical studies in 382 subjects receiving MVA vaccines, only 1 subject (0.3%) met the criteria for vaccine-induced myocarditis and eventually the subject was found to suffer from exercise-induced palpitations. Self-limited mild elevations in troponin I were recorded in 3 subjects (0.8%) without evidence of myo/pericarditis.[8] Based on the current exposure data in more than 8,100 subjects vaccinated with MVA-BN and other MVA-BN recombinant products, the safety profile of MVA-BN has shown to be comparable with other licensed, live attenuated vaccines.

Pregnancy and Birth Control

The effect of the study vaccines on a fetus or nursing baby is unknown, as well as the effect on semen, so female subjects of childbearing potential, and male subjects having sexual intercourse with females, are required to agree to practice adequate birth control measures for sexual intercourse from at least 28 days before the prime vaccination (and boost vaccination if a late boost vaccination will be given for subjects affected by a study pause) (or prior to enrollment for men) until at least 3 months after the prime vaccination or up to 1 month after the boost vaccination (whichever takes longer) (see Section 4.3). Women who are pregnant or breast-feeding, or are planning to become pregnant while enrolled in the study until 3 months after the prime vaccination or up to 1 month after the boost vaccination (whichever takes longer), will be excluded from enrollment into the study. Subjects participating in the Cohort 1 substudy will be asked to use adequate birth control measures for sexual intercourse from at least 14 days before to at least 3 months after the third vaccination.

After completion of the 6-month post-last vaccination visit in the present study, subjects who received placebo may stop birth control measures or resume their pre-study birth control measures.

Risks from Blood Draws

Blood draws may cause pain/tenderness, bruising, bleeding, and, rarely, infection at the site where the blood is taken.

Unknown Risks

There are no clinical data on the use of either vaccine (Ad26.ZEBOV or MVA-BN-Filo) in:

- Pediatric subjects (<18 years);
- Pregnant or nursing women;
- Adults >50 years;
- Immunocompromised subjects (including those with HIV infection).

There may be other serious risks that are not known.

1.2.5. Overall Benefit/Risk Assessment

Based on the available data and proposed safety measures, the overall benefit/risk assessment for this clinical study is considered acceptable for the following reasons:

- Preliminary safety data from the ongoing Phase 1 studies and safety data generated with the 2 vaccines with different inserts revealed no significant safety issues (see Sections 1.1 and 1.2.3). Further experience from Ad26.ZEBOV or MVA-BN-Filo will be gained from currently ongoing clinical studies.
- Only subjects who meet all inclusion criteria and none of the exclusion criteria (specified in Section 4) will be allowed to participate in this study. The selection criteria include adequate provisions to minimize the risk and protect the well-being of subjects in the study.
- Safety will be closely monitored throughout the study:
  - Subjects will remain at the site for a total of 60 (±15) minutes (after prime and boost vaccinations) or 30 (±15) minutes (after the third vaccination) post-vaccination to monitor the development of any acute reactions, or longer if deemed necessary by the investigator (eg, in case of grade 3 adverse events). Refer to Section 6.1 for more information on emergency care. The subjects will be closely observed by study-site personnel for the first 30 (±10) minutes after each vaccination, and again at 60 (±15) minutes after prime and boost vaccinations, and any unsolicited, solicited local or systemic adverse events will be documented during this period. Subjects will use a diary to document solicited local and systemic adverse events in the evening after each vaccination and then daily for the next 7 days at approximately the same time each day. Diaries should be completed at home by the subject (or parent, legal guardian or caregiver, as applicable). For illiterate subjects or those having difficulty completing the diary independently, the sites will make arrangements to have the diary completed according to their local practice.
  - The investigator or the designee will document unsolicited adverse events from signing of the informed consent form (ICF)/assent onwards until 42 days post-last vaccination (excluding the third vaccination), and again from the day of the third vaccination until 28 days thereafter for subjects in the Cohort 1 substudy (note: events that started before the third vaccination but are still present at the time of third vaccination should also be recorded). The investigator or the designee will document serious adverse events and immediate reportable events (IREs) from signing of the ICF/assent onwards until the end of the study.
  - Safety measures, including an electrocardiogram (ECG; for subjects ≥18 years, performed at screening), physical examinations, vital sign measurements, clinical laboratory and pregnancy testing, will be performed at scheduled visits during the study, which lasts up to 1 year after the prime vaccination in subjects who received Ad26.ZEBOV or MVA-BN-Filo.

- Any clinically significant abnormalities (including those persisting at the end of the study/early withdrawal) will be followed by the investigator until resolution or until a clinically stable endpoint is reached.

- Several safety measures are included in this protocol to minimize the potential risk to subjects, including the following:
  - The neuroinflammatory disorders listed in Section 12.1.1 should be categorized as IREs and should be reported to the sponsor as described in Section 12.3.3.
  - There are pre-specified pausing rules that would result in pausing of further vaccination if predefined conditions occur, preventing exposure of new subjects to study vaccine until an Independent Data Monitoring Committee (IDMC) reviews all safety data (see Section 9.3.2).
  - Subjects will discontinue study vaccine for the reasons included in Section 10.2.
  - If acute illness (excluding minor illnesses such as diarrhea or mild upper respiratory tract infection) or fever (body temperature ≥38.0 °C) occur at the scheduled time for vaccination, the subject may be vaccinated up to 10 days beyond the window allowed for the scheduled vaccination, or be withdrawn from that vaccination at the discretion of the investigator and after consultation with the sponsor (see Section 6.2).
  - Contraindications to boost and third vaccinations are included in Section 6.3.
  - If a subject withdraws from the study (withdrawal of consent), he/she maintains the option to participate in the safety follow-up (see Section 10.2).

1.3. Overall Rationale for the Study

In nonclinical studies in the Cynomolgus macaque model, heterologous prime-boost regimens of a multivalent mixture of Ad26 vectors (each expressing EBOV Mayinga, SUDV or MARV GP) and MVA-BN-Filo provided complete protection against the highly pathogenic wild-type EBOV Kikwit 1995 variant (report pending). Further nonclinical studies are ongoing to evaluate the protection of the multivalent vaccine regimen in additional animals and to assess the protective efficacy of a combination regimen of Ad26.ZEBOV and MVA-BN-Filo (either a simultaneous administration or as prime-boost regimen).

In humans, both Ad26- and MVA-based vaccines containing various antigenic inserts have been shown to be safe and immunogenic (see Section 1.1). To date, more than 230 subjects have received the sponsor's Ad26-based vaccines in completed clinical studies (based on the adenoviral vaccine safety database report [dated 20 March 2015]). Up to 28 October 2015, 227 subjects received Ad26.ZEBOV in ongoing studies. The MVA-BN platform is the basis of the non-replicating smallpox vaccine registered in Canada and Europe, and has been safely used in more than 7,600 humans.[21] Although routinely used by the subcutaneous route, MVA-BN at a dose of $1x10^8$ 50% Tissue Culture Infective Dose ($TCID_{50}$) has been demonstrated to be as safe and immunogenic when used by the IM route.[10,26] The IM route has been chosen for the present study.

The unprecedented size and scale of the ongoing Ebola disease outbreak that started in December 2013 in Guinea and subsequently spread to Sierra Leone, Nigeria and Liberia, led to the outbreak being declared a public health emergency of international concern in August 2014 by the WHO. This study is one of a series of studies to evaluate the heterologous combination of Ad26.ZEBOV and MVA-BN-Filo as a possible vaccine regimen to prevent Ebola disease. It will test schedules that are being evaluated in ongoing NHP challenge and Phase 1 clinical studies.

In this Phase 2 study, the sponsor's Ad26 vector expressing the EBOV Mayinga GP (Ad26.ZEBOV) and the MVA-BN vector with EBOV, SUDV and MARV GP inserts and TAFV NP insert (MVA-BN-Filo) will be evaluated as a heterologous prime-boost regimen, in which one study vaccine (Ad26.ZEBOV) is used to prime a filovirus-specific immune response and the other study vaccine (MVA-BN-Filo) is used to boost the immune response 28, 56 or 84 days later. At selected sites in Cohort 1 (Groups 1 and 2), a third vaccination (Ad26.ZEBOV) will be given at least 1 year post prime (substudy). The EBOV GP that is currently circulating in West Africa has 97% homology to the EBOV GP used in this vaccine regimen.[3] The concept of a prime-boost regimen that will be evaluated in the Phase 2 studies with the candidate prophylactic Ebola vaccines Ad26.ZEBOV and MVA-BN-Filo is supported by the results of clinical studies with candidate malaria vaccines which have demonstrated that Ad-based prime immunization followed by MVA-vector boost induced high levels of immunity.

For the prevention of Ebola virus disease, short vaccination schedules may be relevant in the context of the epidemic and suitable for use during acute outbreaks of Ebola. When the current outbreak is under control, longer vaccination schedules may be more relevant than the shorter prime-boost intervals provided a more persistent immune response is observed. The 14-, 28- and 56-day prime-boost intervals are being evaluated in the current Phase 1 studies. An interval longer than 56 days may provide better protection than shorter schedules. Therefore, the objective of this study is to evaluate the immunogenicity of intervals that are slightly shorter as well as slightly longer relative to the 56-day schedule (ie, the schedule that yielded 100% protection in NHP).

The 3 different time intervals (of 28, 56 or 84 days) between the prime and the boost vaccination will be evaluated for safety and tolerability as well as for immunogenicity. These 3 prime-boost regimens will differ only in the timing of the boost vaccination, while the dose of each vaccine and the sequence of vaccination will be identical. The prime vaccine consists of Ad26.ZEBOV at a dose of $5x10^{10}$ vp and the boost vaccine consists of MVA-BN-Filo at a dose of $1x10^{8}$ infectious units (Inf U, nominal titer). The MVA-BN-Filo dose to be used corresponds to the dose of $1x10^{8}$ $TCID_{50}$ that is used in the current Phase 1 studies. In the Cohort 1 substudy, the third vaccination consists of Ad26.ZEBOV at a dose of $5x10^{10}$ vp.

This Phase 2 study will be conducted to enlarge the safety and immunogenicity database for the Ad26.ZEBOV and MVA-BN-Filo prime-boost regimens and to extend the population studied in Phase 1 to include elderly subjects (>50 to 70 years inclusive), HIV-infected subjects, and healthy children (in 2 age strata) in order to cover a large spectrum of the population that would receive a vaccine in a future outbreak setting (ie, mass or ring vaccination). In this Phase 2 study, healthy adult subjects will also be included, ie, the population studied in the Phase 1 studies and in the Phase 2 study performed in Europe (VAC52150EBL2001). In addition, administration of a third vaccination with Ad26.ZEBOV in the Cohort 1 substudy will extend the findings to an African population and confirm the immunogenicity profile from a Phase 1 study (see Section 3.2).

2. OBJECTIVES AND HYPOTHESIS

2.1. Objectives

Primary Objective

The primary objective is to assess the safety and tolerability of different vaccination schedules of Ad26.ZEBOV and MVA-BN-Filo administered IM as heterologous prime-boost regimens on Days 1 and 29, Days 1 and 57, or Days 1 and 85, in healthy adults, including elderly subjects, and on Days 1 and 29 and Days 1 and 57 in HIV-infected subjects and healthy children in 2 age strata.

Secondary Objectives

The secondary objectives are:

- To assess humoral immune responses, as measured by ELISA, to the EBOV GP at 21 days post boost of different vaccination schedules of Ad26.ZEBOV and MVA-BN-Filo administered IM as heterologous prime-boost regimens on Days 1 and 29, Days 1 and 57, or Days 1 and 85, in healthy adults, including elderly subjects, and on Days 1 and 29 and Days 1 and 57 in HIV-infected subjects and healthy children in 2 age strata.
- To assess the safety and tolerability of a third vaccination with Ad26.ZEBOV administered at least 1 year post prime in a subset of approximately 90 healthy adults, including elderly subjects, in Cohort 1 (Groups 1 and 2).

Exploratory Objectives

The exploratory objectives are:

- To assess humoral immune responses, as measured by ELISA, to the EBOV GP at other relevant time points of different vaccination schedules of Ad26.ZEBOV and MVA-BN-Filo administered IM as heterologous prime-boost regimens on Days 1 and 29, Days 1 and 57, or Days 1 and 85, in healthy adults, including elderly subjects, and on Days 1 and 29 and Days 1 and 57 in HIV-infected subjects and healthy children in 2 age strata.

- To assess the neutralizing capacity of the EBOV GP-specific humoral immune responses, as measured by virus neutralization assay, to the EBOV GP of different vaccination schedules of Ad26.ZEBOV and MVA-BN-Filo administered IM as heterologous prime-boost regimens on Days 1 and 29, Days 1 and 57, or Days 1 and 85, in healthy adults, including elderly subjects, and on Days 1 and 29 and Days 1 and 57 in HIV-infected subjects and healthy children in 2 age strata.
- To explore humoral and cellular immune responses to different EBOV GPs and the adenovirus and/or MVA backbone of the various vaccination schedules tested.
- To explore humoral and cellular immune responses to filovirus GPs and/or TAFV NP, if assays are available.
- To assess humoral immune responses, as measured by ELISA, to the EBOV GP at various time points following a third vaccination with Ad26.ZEBOV at least 1 year post prime in approximately 90 healthy adults, including elderly subjects, in Cohort 1 (Groups 1 and 2).

2.2. Hypothesis

As this study is designed to provide descriptive information regarding safety and immunogenicity without formal treatment comparisons, no formal statistical hypothesis testing is planned.

3. STUDY DESIGN AND RATIONALE

3.1. Overview of Study Design

This is a randomized, observer-blind, placebo-controlled, parallel-group, multicenter, Phase 2 study in Africa to evaluate the safety, tolerability and immunogenicity of different heterologous prime-boost regimens using Ad26.ZEBOV at a dose of $5x10^{10}$ vp as prime and MVA-BN-Filo at a dose of $1x10^{8}$ Inf U (nominal titer) as boost at a 28-, 56- or 84-day interval in healthy adult and elderly subjects. The same prime-boost schedules, except for the 84-day interval, will be evaluated in HIV-infected subjects and healthy children in 2 age strata. At selected sites in Cohort 1 (Groups 1 and 2), a third vaccination with Ad26.ZEBOV will be administered at least 1 year post prime to subjects who consent to this (Cohort 1 substudy). Subjects who received a late boost vaccination or did not receive the boost vaccination at all due to a study pause will not be included in the Cohort 1 substudy.

This study will enroll a planned total number of 1,056 subjects, who have never received a candidate Ebola vaccine and have no prior exposure to Ebola virus (including travel to epidemic Ebola areas less than 1 month prior to screening) or a diagnosis of Ebola virus disease.

The study is comprised of 3 cohorts, which will be enrolled sequentially (see Figure 1): healthy adult and elderly subjects (aged 18 to 70 years inclusive, Cohort 1), HIV-infected subjects (aged 18 to 50 years inclusive, Cohort 2a) and healthy children in 2 age strata (adolescents aged 12 to 17 years inclusive, Cohort 2b; children aged 4 to 11 years inclusive, Cohort 3). The prime-boost regimens that will be evaluated in this study will differ only in the timing of the boost vaccination (ie, 28, 56 or 84 days after prime, respectively referred to as Groups 1, 2 and 3), while the dose of each study vaccine (Ad26.ZEBOV, MVA-BN-Filo or placebo) and the

sequence of prime-boost vaccination will be identical. Groups 1 and 2 will be evaluated in each cohort, while Group 3 (84-day interval) will be evaluated in Cohort 1 only.

In Cohort 1, subjects will be enrolled in parallel and randomized in a 1:1:1 ratio to Groups 1, 2 and 3 at baseline until a target of 132 subjects have been included in Group 3. Afterwards, randomization in this cohort will proceed in a 1:1 ratio to Groups 1 and 2. In Cohorts 2a, 2b, and 3, subjects will be enrolled in parallel and randomized in a 1:1 ratio to Groups 1 and 2 at baseline. Within each group (for all cohorts), subjects will be randomized in a 5:1 ratio to receive Ad26.ZEBOV and MVA-BN-Filo versus placebo.

Available interim analysis results of 7-day post-prime safety and immunogenicity data from the ongoing and/or planned Phase 1 studies (studies in Europe, US and Africa) will be provided before the start of the present study, which will start with Cohort 1. Progression to the next cohort will proceed following favorable IDMC review:

- Cohorts 2a and 2b can start when 25% of subjects in Cohort 1 have completed their 7-day post-prime visit.
- Cohort 3 can start when 50% of subjects in Cohort 2b have completed their 7-day post-prime visit.

The real-time monitoring of blinded data will be conducted by the sponsor. The interim data (unblinded) will be provided to and reviewed by the IDMC. The decision to open Cohorts 2a, 2b, and 3 will be based on the sponsor's and the IDMC's review of the interim safety data. Progression to the next cohort will also take into account pre-specified pausing rules (see Section 9.3.2).

A schematic overview of the study design is presented in Figure 1.

Figure 1: Schematic Overview of the Study

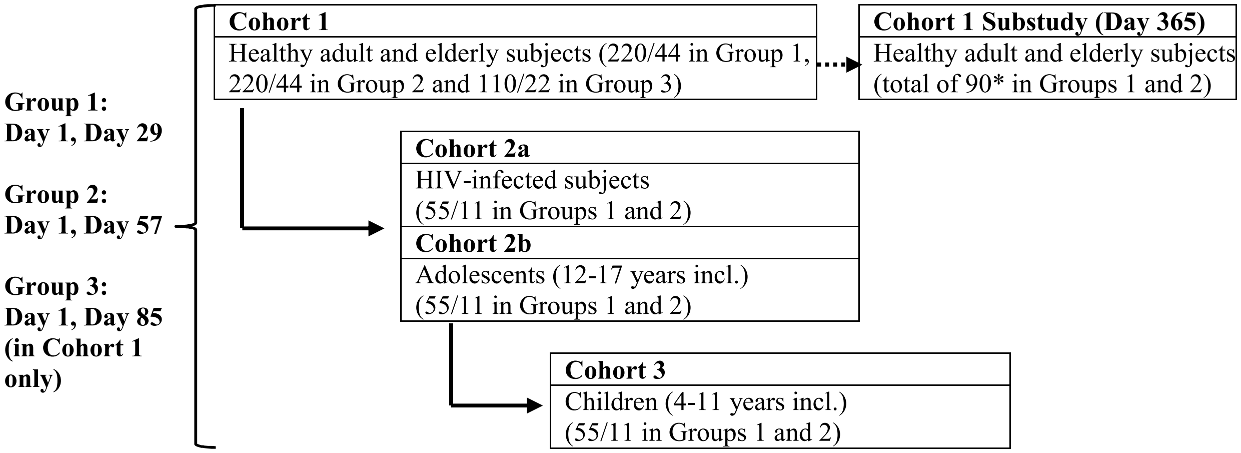

Number/number: number of subjects per group randomized to Ad26.ZEBOV and MVA-BN-Filo/placebo.

- Cohorts 2a and 2b: starts when 25% of subjects from Cohort 1 have reached the 7-day post-prime visit
- Cohort 3: starts when 50% of subjects from Cohort 2b have reached the 7-day post-prime visit

* Subjects who received Ad26.ZEBOV and MVA-BN-Filo will receive Ad26.ZEBOV as third vaccination at least 1 year post prime. Subjects who received placebo will receive placebo as third vaccination at least 1 year post prime.

Within each cohort, study-site personnel (except for those with primary responsibility for study vaccine preparation and dispensing), and subjects will be blinded to the study vaccine allocation until the last subject in that cohort has completed the study. Refer to Section 5 for further details on blinding in case of interim analyses.

The study consists of a screening phase of up to 8 weeks (starting from the moment the subject signs the ICF and/or informed assent), a vaccination phase in which subjects will be vaccinated at baseline (Day 1) followed by a boost vaccination on Day 29, 57 or 85, and a post-vaccination phase and long-term follow-up phase until Day 365. The Cohort 1 substudy consists of a vaccination phase in which subjects will be vaccinated at least 1 year post prime vaccination, a post-vaccination phase, and a long-term follow-up phase until the 1-year post third vaccination visit. All subjects within a cohort will be followed in a blinded manner by the site until the last subject in that cohort has completed the study. Any subject who completed either the 6-month post-prime and/or the Day 365 visit prior to approval of Amendment 2 was required to attend the 6-month post-boost and the Day 365 visit after approval of Amendment 2. Refer to Section 9.1.6 for study procedures that need to be followed in case of a study pause.

The baseline visit may be scheduled as soon as the results of all screening assessments are known (but should occur within 8 weeks from screening, see Section 9.1.2) and show that the subject is eligible for inclusion. The administration of the prime vaccination will occur on Day 1 (baseline), after the completion of all baseline assessments.

All subjects will receive the study vaccine (Ad26.ZEBOV, MVA-BN-Filo or placebo) through IM injection (0.5 mL) in the deltoid muscle:

- Ad26.ZEBOV ($5x10^{10}$ vp) on Day 1, followed by a boost vaccination of MVA-BN-Filo ($1x10^{8}$ Inf U) on Day 29, 57 or 85; *OR*
- Placebo (0.9% saline) on Day 1, followed by a boost vaccination of placebo (0.9% saline) on Day 29, 57 or 85.

In a substudy at selected sites, approximately 90 healthy adult subjects in Cohort 1 (Groups 1 and 2) will receive a third vaccination with Ad26.ZEBOV at $5x10^{10}$ vp or placebo at least 1 year post prime vaccination.

Refer to Section 6 for further details on dosage and administration. Subjects will remain at the site for a total of 60 (±15) minutes (after prime and boost vaccinations) or 30 (±15) minutes (after the third vaccination) post-vaccination to monitor for the development of any acute reactions, or longer if deemed necessary by the investigator (eg, in case of grade 3 adverse events).

The investigators, together with the sponsor's medical monitor, will be responsible for the safety monitoring of the study, and will halt vaccination of further subjects in case any of the pre-specified pausing rules described in Section 9.3.2 have been met. Criteria for postponement of vaccination at the scheduled time for vaccine administration and contraindications to boost and third vaccinations have been defined and will be applied by the investigator (not including those occurring because of study hold) (see Sections 6.2 and 6.3 respectively). Discontinuation of study vaccine should occur in any subject meeting the criteria outlined in Section 10.2.

Safety will be assessed by collection of solicited local and systemic adverse events (reactogenicity), unsolicited adverse events, serious adverse events, and IREs. The subjects will be closely observed by study-site personnel for the first 30 (±10) minutes after each vaccination, and again at 60 (±15) minutes after prime and boost vaccinations, and any unsolicited, solicited local or systemic adverse events will be documented during this period. Upon discharge from the site, subjects will receive a diary, a thermometer and a ruler to measure solicited local reactions and body temperature. Subjects will be instructed to record solicited local and systemic adverse events in the diary in the evening after each vaccination and then daily for the next 7 days at approximately the same time each day. Diaries should be completed at home by the subject (or parent, legal guardian or caregiver, as applicable). For illiterate subjects or those having difficulty completing the diary independently, the sites will make arrangements to have the diary completed according to their local practice. The investigator will document unsolicited adverse events from signing of the ICF/assent onwards until 42 days post-last vaccination (excluding the third vaccination), and again from the day of the third vaccination until 28 days thereafter for subjects in the Cohort 1 substudy (note: events that started before the third vaccination but are still present at the time of third vaccination should also be recorded). The investigator will document serious adverse events and IREs from signing of the ICF/assent onwards until the end of the study. The primary (or secondary for Cohort 1 substudy) endpoints are adverse events,

serious adverse events, IREs and solicited local and systemic adverse events (see Section 11.1). Other safety assessments include an ECG for subjects ≥18 years of age (performed at screening), physical examination, vital signs (blood pressure, pulse/heart rate, body temperature), clinical laboratory and pregnancy testing.

In addition, the investigator will collect samples for immunogenicity assessments at the time points indicated in the Time and Events Schedule. Samples to assess humoral responses will be taken from all subjects; samples to assess cellular immune responses will be taken from subjects at selected sites (targeted at 165 subjects [138 Ad26.ZEBOV and MVA-BN-Filo, and 27 placebo] in Cohort 1 and 33 subjects [28 Ad26.ZEBOV and MVA-BN-Filo, and 5 placebo] in each of the other cohorts). Peripheral blood mononuclear cell (PBMC) sampling at selected sites will continue until the targeted number of subjects is reached. Samples to assess cellular immune responses are optional for subjects in the Cohort 1 substudy and may only be collected in a subset of subjects. Subjects giving informed consent for the study will be informed that their leftover blood samples will be stored for potential future research. Subjects participating at selected sites where PBMC samples are collected will be asked explicitly to consent for potential future genetic research to be performed on PBMC samples. Subjects can withdraw consent for their samples to be used for future research at any time (see Section 16.2.5).

The primary analysis will be conducted when all subjects in Cohort 1 have completed the 6-month post-boost visit or discontinued earlier, to aid in the assessment of which vaccination schedule will be the best candidate for future studies and implementation in the field. Study-site personnel, subjects and sponsor personnel involved in subject level data review will remain blinded until the last subject in that cohort has completed the study. Sponsor personnel involved in the conduct of the primary analysis and in making future decisions for the program will be unblinded to the data of Cohort 1, but will remain blinded to the data of the other cohorts.

For the Cohort 1 substudy of subjects who received the third vaccination, an interim analysis may be performed when all subjects have completed the 6-month post third vaccination visit, or discontinued earlier.

In each cohort, after completion of the 6-month post-boost visit by all subjects, an interim analysis will be conducted on safety and selected immunogenicity data and the cohort will be unblinded to the sponsor. Subjects who completed the 6-month post-prime visit prior to approval of Amendment 2 were required to attend the 6-month post-boost visit after approval of Amendment 2.

The final analysis will be performed when all subjects have completed the last study-related visit or discontinued earlier (including subjects participating in the Cohort 1 substudy).

While Cohort 1 was enrolling, the sponsor halted all vaccinations in this study due to the occurrence of a serious and very rare condition, Miller Fisher syndrome, reported in study VAC52150EBL2001 (described in Section 1.1.2.3) until a revised ICF was prepared and approval to restart the study was granted by the relevant competent authority. This interruption in dosing affected over 200 consented subjects, some who were awaiting prime vaccination and

some awaiting boost vaccination. When approval was granted to restart the study under Amendment 1, a late boost vaccination has been offered to those subjects who were out-of-window for the boost vaccination, unless it was prohibited. Those subjects who agreed to the late boost have been following the procedures as outlined in Amendment 1.

All subjects (including placebo) who were primed prior to the pause and did not/will not receive a late boost vaccination are being followed every 3 months after the prime vaccination for safety until Day 365 (see Additional Time and Events Schedule for subjects who do not receive a boost vaccination because of a study pause). Therefore, non-serious adverse events will be reported until the 42-day post-**last** vaccination (excluding third vaccination) visit rather than the 42-day post-boost vaccination visit. Serious adverse events and IREs will be reported throughout the study until Day 365 (for details, see Section 9.3.1).

Female subjects who became pregnant with estimated conception within 28 days after vaccination with MVA-BN-Filo (or placebo) or within 3 months after vaccination with Ad26.ZEBOV (or placebo) and children born to vaccinated female subjects who became pregnant with estimated conception within 28 days after vaccination with MVA-BN-Filo (or placebo) or within 3 months after vaccination with Ad26.ZEBOV (or placebo) (unless local regulations have additional requirements for follow-up) will be eligible for enrollment into the VAC52150 Vaccine Development Roll-over study (VAC52150EBL4001) for long-term surveillance (for a total of up to 60 months after the prime vaccination) (see Section 9.1.8 for details). After unblinding, only female subjects and the children of female subjects who received Ad26.ZEBOV and/or MVA-BN-Filo will remain in the VAC52150 Vaccine Development Roll-over study for long-term surveillance. After unblinding, female subjects and the children of female subjects who received placebo and have already been enrolled into the VAC52150 Vaccine Development Roll-over study will be discontinued from further participation in the roll-over study.

3.2. Study Design Rationale

The study design is largely driven by the accelerated development approach of the Ad26.ZEBOV and MVA-BN-Filo prime-boost combination. Based on the growing availability of data from the entire development program as the study proceeds, modifications to the study design may be considered. For instance, if the results from the 28-day prime-boost interval (or any other interval) are not favorable based on the evaluation of the NHP and Phase 1 data (safety and immunogenicity) as soon as these become available, the enrollment in this group will be stopped (if not yet completed). When enrollment in one of the regimens will be stopped, the number of subjects still to be enrolled for this regimen will be randomized to the other regimen(s) to ensure that the same number of subjects can be included in an overall safety database of the Ad26.ZEBOV and MVA-BN-Filo prime-boost regimen.

The interruption in dosing and subsequent restart has required changes to the management of the subjects affected by the pause. All subjects (including placebo) who were primed prior to the pause and did not/will not receive a late boost vaccination will be followed for safety. Subjects

who did/will receive a late boost vaccination will follow the same post-boost vaccination schedule as those subjects unaffected by the pause (for details, see Section 9.1.6).

Immunogenicity data from a Phase 1 study (VAC52150EBL1002) demonstrated stability of the binding antibody response out to 1 year following prime-boost regimens with Ad26.ZEBOV/MVA-BN-Filo (or the reverse). A third vaccination using Ad26.ZEBOV at $5x10^{10}$ vp was given at the 1-year time point leading to a marked and rapid increase in the binding antibody responses within 7 days (at least 10-fold). The marked and rapid rise was generally independent of the antecedent prime-boost regimen. The profile of the antibody strongly suggests that robust anamnestic responses can be induced after re-exposure to an EBOV antigen, in this case mimicked by a third vaccination. Administration of a third vaccination with Ad26.ZEBOV at least 1 year post prime for approximately 90 subjects in Cohort 1 will extend the findings to an African population and confirm the immunogenicity profile from the Phase 1 study.

Control and Blinding

Randomization will be used to minimize bias in the assignment of subjects to vaccination schedules (groups), to increase the likelihood that known and unknown subject attributes (eg, demographic and baseline characteristics) are evenly balanced across groups, and to enhance the validity of possible statistical comparisons across groups.

In addition, randomization will be used to minimize bias in the assignment of subjects to study vaccine (Ad26.ZEBOV and MVA-BN-Filo versus placebo). Placebo recipients are included for blinding purposes and safety analyses, and will provide control specimens for immunogenicity assays. For the purposes of regulatory submission, a placebo control will be used up to 6 months post-boost to establish the frequency and magnitude of changes in clinical and immunologic endpoints that may occur in the absence of Ad26.ZEBOV or MVA-BN-Filo. The nature of the study vaccine (Ad26.ZEBOV and MVA-BN-Filo versus placebo) will be blind to reduce potential bias during data collection and evaluation of clinical safety endpoints. Blinding will be guaranteed by preparation of study vaccine by unblinded qualified study-site personnel not involved in any other study-related procedure, and by the administration of vaccine in a masked syringe by a blinded vaccine administrator (see Definitions of Terms). No additional placebo injections will be administered to mask the vaccination schedule across groups.

Study Groups

The 3 vaccination schedules (Groups 1, 2, and 3) will only differ in the timing of the boost vaccination (ie, 28-, 56-, or 84-day interval), while the dose of each vaccine and the sequence of vaccination will be identical. The prime vaccine consists of Ad26.ZEBOV at a dose of $5x10^{10}$ vp and the boost vaccine consists of MVA-BN-Filo at a dose of $1x10^{8}$ Inf U, which corresponds to the dose of $1x10^{8}$ $TCID_{50}$ that is used in the current Phase 1 studies. The safety, tolerability and immunogenicity results for the 3 vaccination schedules will be evaluated in the study. The 84-day interval may be evaluated in the special populations at a later point in time of the development program. The safety, tolerability and immunogenicity results of a third vaccination

with Ad26.ZEBOV at a dose of $5x10^{10}$ vp (at least 1 year post prime vaccination) will be evaluated in the Cohort 1 substudy.

Future Research

Subjects giving informed consent for the study will be informed that their leftover blood samples (serum and/or PBMC) will be stored for potential future research (see Section 16.2.5). Future scientific research may be conducted to further investigate Ebola vaccine- and disease-related questions. This may include the development of new or the improvement of existing techniques to characterize EBOV-directed immune responses or diagnostic tests. Subjects participating at selected sites where PBMC samples are collected will be asked explicitly to consent for potential future genetic research to be performed on PBMC samples. Subjects can withdraw consent for their samples to be used for future research at any time.

4. SUBJECT POPULATION

Screening of subjects for eligibility will be performed within 8 weeks before administration of study vaccine on Day 1. Subjects (or parent[s]/legal guardian for pediatric subjects less than 18 years of age) must sign an ICF indicating that he or she understands the purpose of and procedures required for the study and is willing to participate in the study. Assent should be obtained from children capable of understanding the nature of the study, typically subjects 7 years of age and older, depending on local regulations and practice (see also Sections 1.1 and 16.2.3). Signing of the ICF and/or informed assent needs to be done before the first study-related activity.

The inclusion and exclusion criteria for enrolling subjects in this study are described in the following 2 subsections. If there is a question about the inclusion or exclusion criteria below, the investigator must consult with the appropriate sponsor representative and resolve any issues before enrolling a subject in the study. Waivers are not allowed.

For a discussion of the statistical considerations of subject selection, refer to Section 11.2, Sample Size Determination.

NOTE: The investigator should ensure that all study enrollment criteria have been met at the end of the screening period. If a subject’s clinical status changes (including any available laboratory results or the receipt of additional medical records) after screening but before Day 1 so the subject no longer meets the eligibility criteria, then the subject should be excluded from further participation in the study. Section 17.4 describes the required documentation to support meeting the enrollment criteria.

4.1. Inclusion Criteria

4.1.1. Inclusion Criteria for Healthy Adult and Elderly Subjects

Each potential subject must satisfy all of the following criteria to be enrolled in the study.

1. Each subject must sign an ICF indicating that he or she understands the purpose of, and procedures required for, the study and is willing to participate in the study. In case the subject cannot read or write, the procedures must be explained and informed consent must be witnessed by a literate third party not involved with the conduct of the study.
2. Subject must be a man or woman aged 18 (or the legal age of consent in the jurisdiction in which the study is taking place) to 70 years of age, inclusive at randomization.
3. Subject must be healthy in the investigator’s clinical judgment on the basis of medical history, ECG, physical examination and vital signs performed at screening.
4. Criterion modified per Amendment 2

4.1. Subject must be healthy on the basis of clinical laboratory tests performed at screening. If the results of the laboratory screening tests are outside the institutional normal reference ranges, the subject may be included only if the investigator judges the abnormalities or deviations from normal to be not clinically significant or to be appropriate and reasonable for the population under study. This determination must be recorded in the subject's source documents and initialed by the investigator. However, the subject should not be included when hemoglobin is lower than the institutional normal reference range (or below the values in Attachment 4 when that range is not available).

Note: The safety laboratory assessments at screening are to be performed within 28 days prior to the prime vaccination on Day 1 (including Day 1 before vaccination) and may be repeated if they fall outside this time window.

Note: In case of menstruation, urinalysis must be postponed but a result should be available before the prime vaccination. All urinalyses should be collected as freshly voided, mid-stream, clean catch samples obtained after proper cleansing.

Note: If laboratory screening tests are out of range and deemed clinically significant, repeat of screening tests is permitted once using an unscheduled visit during the screening period to assess eligibility.

5. Criterion modified per Amendment 2

5.1. Before randomization (on Day 1), a sexually active woman must be either:

– Of childbearing potential and practicing a highly effective method of birth control consistent with local regulations regarding the use of birth control methods for subjects participating in clinical studies, beginning at least 28 days prior to vaccination. The sponsor considers the following methods of birth control to be highly effective: established use of oral, injected or implanted hormonal methods of contraception; placement of an intrauterine device or intrauterine system; barrier methods (condom or occlusive cap [diaphragm or cervical/vault caps] with spermicidal foam/gel/film/cream/suppository); male partner sterilization (the vasectomized partner should be the sole partner for that subject). More restrictive measures may be required by the site.

Note: Abstinence or natural family planning are not acceptable birth control methods.

OR

– Not of childbearing potential: postmenopausal (>45 years of age with amenorrhea for at least 2 years or ≤45 years of age with amenorrhea for at least 6 months and a serum follicle stimulating hormone (FSH) level >40 mIU/mL); permanently sterilized (eg, bilateral tubal occlusion [which includes tubal ligation procedures as consistent with local regulations], hysterectomy, bilateral salpingectomy, bilateral oophorectomy); or otherwise be incapable of pregnancy.

Note: If the social situation of a woman changes after start of the study (eg, woman who is not heterosexually active becomes active), she must begin a highly effective method of birth control, as described above.

Note: Women ≤45 years of age with amenorrhea for ≤6 months are considered of childbearing potential and do not need FSH testing.

6. A woman of childbearing potential must have a negative serum β-human chorionic gonadotropin (β-hCG) pregnancy test at screening and a negative urine β-hCG pregnancy test immediately prior to each study vaccine administration.

Note: If the pregnancy test result is positive, in order to maintain subject confidentiality, the investigator will ensure adequate counseling and follow-up will be made available particularly for adolescent subjects.

7. Criterion modified per Amendment 2

7.1. A man who is sexually active with a woman of childbearing potential must be willing to use condoms for sexual intercourse beginning prior to enrollment, unless a vasectomy was performed more than 1 year prior to screening.

8. Subject must be available and willing to participate for the duration of the study visits and follow-up.

9. Subject must be willing and able to comply with the protocol requirements, including the Prohibitions and Restrictions specified in Section 4.3.

10. Subject must be willing to provide verifiable identification.

11. Subject must have a means to be contacted.

12. Criterion modified per Amendment 2

12.1. Subject must pass the test of understanding (TOU) (Attachment 1).

Note: If subjects fail the TOU test on the first attempt, subjects must be retrained on the purpose of the study and must take the test again (2 repeats are allowed). If subjects fail on the third attempt, they should not continue with screening or consenting procedures.

4.1.2. Additional Inclusion Criteria for HIV-infected Subjects

All of the inclusion criteria for healthy adult and elderly subjects (see Section 4.1.1) must be met by adults who are HIV-infected with the following additions/clarifications:

13. Subject must be a man or woman aged 18 (or the legal age of consent in the jurisdiction in which the study is taking place) to 50 years of age, inclusive at randomization.

14. Subject must have a documented HIV-infection for at least 6 months prior to screening.

15. Criterion modified per Amendment 3

15.1. Subject must be on a stable regimen of Highly Active Antiretroviral Therapy (HAART), taking into account the following criteria:

a. HAART is defined as potent anti-HIV treatment including a combination of ≥3 antiretroviral agents (ARVs; low-dose ritonavir does not count as an ARV) whose purpose is to reduce viral load to undetectable levels. Mono- or bitherapy will not be allowed.

b. HAART is considered stable if subjects did not change their ARVs within the last 4 consecutive weeks prior to start of screening. Changes in formulations are allowed.

c. A subject entering the study on HAART should have a screening CD4+ cell count >350 cells/μL. Two documented results from at least 4 weeks apart are preferred. Viral load assessments may be used to establish effectiveness of viral suppression.

d. Subject must be willing to continue their HAART throughout the study as directed by their local physician.

16. Subject must be in an otherwise reasonably good medical condition (absence of acquired immunodeficiency syndrome [AIDS]-defining illnesses or clinically significant disease), diagnosed on the basis of physical examination, medical history and the investigator's clinical judgment (refer to Section 4.1.1 for details).

4.1.3. Inclusion Criteria for Pediatric Subjects

Each potential subject must satisfy all of the following criteria to be enrolled in the study:

17. Parent(s)/legal guardian must have signed an ICF indicating that they understand the purpose of, and procedures required for, the study, are willing/able to adhere to the prohibitions and restrictions specified in the protocol and study procedures, and are willing for their child to participate in the study. Informed assent must be obtained from adolescents and older children, depending on local regulations and practice (see also Section 4).

    Note: *Consent from both parents might be required according to local regulations and practice.*

18. Criterion modified per Amendment 3

18.1. Criterion modified per Amendment 2

18.2. Subject must be male or female whose age on the day of randomization is within one of the 2 age strata in the study: 12-17 years or 4-11 years (all ages inclusive).

19. Subject must be healthy in the investigator's clinical judgment (and the parent(s)/legal guardian) on the basis of medical history, physical examination and vital signs performed at screening.

20. Criterion modified per Amendment 2

20.1. Subject must be healthy on the basis of clinical laboratory tests performed at screening. If the results of the laboratory screening tests are outside the institutional normal reference ranges, the subject may be included only if the investigator judges the abnormalities or deviations from normal to be not clinically significant or to be appropriate and reasonable for the population under study. This determination must be recorded in the subject's source documents and initialed by the investigator. However, the subject should not be included when hemoglobin is lower than the institutional normal reference range (or below the values in Attachment 4 when that range is not available).

    Note: *The safety laboratory assessments at screening are to be performed within 28 days prior to the prime vaccination on Day 1 (including Day 1 before vaccination) and may be repeated if they fall outside this time window.*

    Note: *In case of menstruation, urinalysis must be postponed but a result should be available before the prime vaccination. All urinalyses should be collected as freshly voided, mid-stream, clean catch samples obtained after proper cleansing.*

    Note: *If laboratory screening tests are out of range and deemed clinically significant, repeat of screening tests is permitted once using an unscheduled visit during the screening period to assess eligibility.*

21. Criterion modified per Amendment 2

21.1. Before randomization (on Day 1), a female subject must be either:

- Of childbearing potential and practicing a highly effective method of birth control consistent with local regulations regarding the use of birth control methods for subjects participating in clinical studies, beginning at least 28 days prior to vaccination. The sponsor considers the following methods of birth control to be highly effective: established use of oral, injected or implanted hormonal methods of contraception; placement of an intrauterine device or intrauterine system; barrier methods (condom or occlusive cap [diaphragm or cervical/vault caps] with spermicidal foam/gel/film/cream/suppository); male partner sterilization (the vasectomized partner should be the sole partner for that subject). More restrictive measures may be required by the site.

<u>Note</u>: *Abstinence or natural family planning are not acceptable birth control methods for sexually active women of any age.*

OR

- Not of childbearing potential: premenarchal.

<u>Note</u>: *If the social situation of a female subject changes after start of the study (eg, female subject who is not heterosexually active becomes active), she must begin a highly effective method of birth control, as described above.*

22. A female subject of childbearing potential must have a negative serum β-hCG pregnancy test at screening and a negative urine β-hCG pregnancy test immediately prior to each study vaccine administration.

<u>Note</u>: *If the pregnancy test result is positive, in order to maintain subject confidentiality, the investigator will ensure adequate counseling and follow-up will be made available.*

23. Criterion modified per Amendment 2

23.1. A male subject who is sexually active with a female subject of childbearing potential must be willing to use condoms for sexual intercourse beginning prior to enrollment.

24. Subject and parent(s)/legal guardian are available and willing to participate for the duration of the study visits and follow-up.

25. Subject and parent(s)/legal guardian must be willing and able to comply with the protocol requirements, including the Prohibitions and Restrictions specified in Section 4.3.

26. Subject and parent(s)/legal guardian must have a means to be contacted.

27. Criterion modified per Amendment 2

27.1. The parent(s)/legal guardian must pass the TOU (Attachment 1). Older children and adolescents who provided assent according to local regulations and practice, must also pass the TOU.

Note: If the subject/parent(s)/legal guardian fails the TOU test on the first attempt, he/she must be retrained on the purpose of the study and must take the test again (2 repeats are allowed). If subjects fail on the third attempt, they should not continue with screening or consenting procedures.

28. Subject has received all routine immunizations appropriate for his or her age as reported by the parent(s)/legal guardian, according to local routine vaccination schedules. Subjects are allowed to catch up on routine immunizations if needed (support for beneficial vaccines may be offered to participants).

4.2. Exclusion Criteria

4.2.1. Exclusion Criteria for Healthy Adult and Elderly Subjects

Any potential subject who meets any of the following criteria will be excluded from participating in the study.

1. Has received any candidate Ebola vaccine.
2. Diagnosed with Ebola virus disease, or prior exposure to Ebola virus, including travel to epidemic Ebola areas less than 1 month prior to screening.

   Note: Participation of international volunteers to Ebola operations is allowed, but they should comply with the prohibitions and restrictions as specified in Section 4.3.
3. Has received any experimental candidate Ad26- or MVA-based vaccine in the past.

   Note: Receipt of any approved vaccinia/smallpox vaccine or Ad-based candidate vaccine other than Ad26 at any time prior to study entry is allowed.
4. Known allergy or history of anaphylaxis or other serious adverse reactions to vaccines or vaccine products (including any of the constituents of the study vaccines [eg, polysorbate 80, ethylenediaminetetraacetic acid (EDTA) or L-histidine for Ad26.ZEBOV vaccine; tris (hydroxymethyl)-amino methane (THAM) for MVA-BN-Filo vaccine]), including known allergy to egg, egg products and aminoglycosides.
5. Subject with acute illness (this does not include minor illnesses such as diarrhea or mild upper respiratory tract infection) or body temperature ≥38.0ºC on Day 1 will be excluded from enrollment at that time, but may be rescheduled for enrollment at a later date.
6. HIV type 1 or type 2 infection.

   Note: If the HIV test result is positive, in order to maintain subject confidentiality, the investigator will ensure adequate counseling and follow-up will be made available especially in adolescent subjects.

7. Criterion modified per Amendment 3

7.1. A woman who is pregnant, breast-feeding or planning to become pregnant while enrolled in the study or within at least 3 months after the prime vaccination, up to 1 month after the boost vaccination (whichever takes longer), or within at least 3 months after the third vaccination.

8. Presence of significant conditions (eg, history of seizure disorders, (auto)immune disease or deficiency, any spleen disease, active malignancy, ongoing tuberculosis treatment, other systemic infections) or clinically significant findings during screening of medical history, ECG (for subjects ≥18 years of age), physical examination, vital signs or laboratory testing for which, in the opinion of the investigator, participation would not be in the best interest of the subject (eg, compromise the safety or well-being) or that could prevent, limit, or confound the protocol-specified assessments.

   Note: Subjects who have recently received treatment for acute, uncomplicated malaria are eligible for participation if at least 3 days have elapsed from the conclusion of a standard, recommended course of therapy for malaria; subjects who are acutely ill with malaria at the time of screening should complete therapy and wait an additional 3 days after completion before screening for the study.

   Note: Subjects with sickle cell trait can be included.

9. Criterion modified per Amendment 3

9.1. History of or underlying liver or renal insufficiency, or significant cardiac, vascular, pulmonary (eg, persistent asthma), gastrointestinal, endocrine, neurologic, hematologic, rheumatologic, psychiatric or metabolic disturbances.

10. History of malignancy other than squamous cell or basal cell skin cancer, unless there has been surgical excision that is considered cured. Subjects with malignancies who are currently being treated or are not surgical cures are excluded.

11. Major surgery (per the investigator's judgment) within the 4 weeks prior to screening or planned major surgery through the course of the study (from screening until completion of the study, see Section 10.1).

12. Post-organ and/or stem cell transplant whether or not with chronic immunosuppressive therapy.

13. Receipt of any disallowed therapies as noted in Section 8 before the planned administration of the prime vaccine on Day 1.

14. Received an investigational drug or investigational vaccine or used an invasive investigational medical device within 3 months prior to screening, or current or planned participation in another clinical study during the study.

    Note: Participation in an observational clinical study is allowed.

15. Criterion modified per Amendment 2

15.1. Donation of a unit of blood within 8 weeks before Day 1 or plans to donate blood until 42 days post-last vaccination.

16. Receipt of blood products or immunoglobulin within 3 months prior to screening and during participation in the study.
17. Current or past abuse of alcohol, recreational or narcotic drugs, which in the investigator's opinion would compromise the subject's safety and/or compliance with the study procedures.
18. History of chronic urticaria (recurrent hives).
19. Subject cannot communicate reliably with the investigator.
20. Subject who, in the opinion of the investigator, is unlikely to adhere to the requirements of the study.
21. Employee of the investigator or study site, with direct involvement in the proposed study or other studies under the direction of that investigator or study site, as well as family members of the employees or the investigator.

4.2.2. Additional Exclusion Criteria for HIV-infected Subjects

HIV-infected subjects must not meet any of the exclusion criteria for healthy adult and elderly subjects (see Section 4.2.1) with the exception of #6 which is not applicable to this population.

4.2.3. Exclusion Criteria for Pediatric Subjects

Any potential subject who meets any of the following criteria will be excluded from participating in the study:

22. Has received any candidate Ebola vaccine.
23. Diagnosed with Ebola virus disease, or prior exposure to Ebola virus, including travel to epidemic Ebola areas less than 1 month prior to screening.
24. Has received any experimental candidate Ad26- or MVA-based vaccine in the past.

    Note: Receipt of any approved vaccinia/smallpox vaccine or Ad-based candidate vaccine other than Ad26 at any time prior to study entry is allowed.

25. Known allergy or history of anaphylaxis or other serious adverse reactions to vaccines or vaccine products (including any of the constituents of the study vaccines [eg, polysorbate 80, EDTA or L-histidine for Ad26.ZEBOV vaccine; THAM for MVA-BN-Filo vaccine]), including known allergy to egg, egg products and aminoglycosides, or to neomycin or streptomycin.
26. Subject with acute illness (this does not include minor illnesses such as diarrhea or mild upper respiratory tract infection) or body temperature ≥38.0ºC on Day 1 will be excluded from enrollment at that time, but may be rescheduled for enrollment at a later date.
27. Criterion modified per Amendment 3

27.1. HIV type 1 or type 2 infection.

Note: If the HIV test result is positive, in order to maintain subject confidentiality, the investigator will ensure adequate counseling and follow-up will be made available.

28. A female subject who is pregnant, breast-feeding or planning to become pregnant while enrolled in the study or within 3 months after the prime vaccination or up to 1 month after the boost vaccination (whichever takes longer).

29. Criterion modified per Amendment 3

29.1. Weight-per-height below 10th percentile according to the Centers for Disease Control and Prevention (CDC) growth charts (4- to 11-year-olds).[5]

30. Presence of significant conditions (eg, history of seizure disorders, (auto)immune disease or deficiency, any spleen disease, active malignancy, ongoing tuberculosis treatment, other systemic infections) or clinically significant findings during screening of medical history, physical examination, laboratory testing or vital signs for which, in the opinion of the investigator, participation would not be in the best interest of the subject (eg, compromise the safety or well-being) or that could prevent, limit, or confound the protocol-specified assessments.

    Note: Subjects who have recently received treatment for acute, uncomplicated malaria are eligible for participation if at least 3 days have elapsed from the conclusion of a standard, recommended course of therapy for malaria; subjects who are acutely ill with malaria at the time of screening should complete therapy and wait an additional 3 days after completion before screening for the study.

31. Criterion modified per Amendment 3

31.1 History of or underlying liver or renal insufficiency, or significant cardiac, vascular, pulmonary (eg, persistent asthma), gastrointestinal, endocrine, neurologic, hematologic, rheumatologic, psychiatric or metabolic disturbances.

32. Major congenital anomalies or known cytogenetic disorders (eg, Down's syndrome). Subjects with sickle cell trait can be included.

33. Major surgery (per the investigator's judgment) within the 4 weeks prior to screening or planned major surgery through the course of the study (from screening until completion of the study, see Section 10.1).

34. Receipt of any disallowed therapies as noted in Section 8 before the planned administration of the prime vaccine on Day 1.

35. Received an investigational drug or investigational vaccine or used an invasive investigational medical device within 3 months prior to screening, or current or planned participation in another clinical study during the study.

    Note: Participation in an observational clinical study is allowed.

36. Receipt of blood products or immunoglobulin within 3 months prior to screening and during participation in the study.

37. History of chronic urticaria (recurrent hives), eczema and/or atopic dermatitis.

38. Criterion modified per Amendment 2

38.1. Subject lives in an orphanage.

39. Subject's parent(s)/legal guardian cannot communicate reliably with the investigator.
40. Subject or parent(s)/legal guardian who, in the opinion of the investigator, is unlikely to adhere to the requirements of the study.

4.3. Prohibitions and Restrictions

Potential subjects must be willing and able to adhere to the following prohibitions and restrictions during the course of the study to be eligible for participation:

1. Criterion modified per Amendment 3

1.1. Criterion modified per Amendment 2

1.2. Female subjects of childbearing potential must remain on a highly effective method of birth control consistent with local regulations regarding the use of birth control methods for subjects participating in clinical studies (see inclusion criteria) until at least 3 months after the prime vaccination or up to 1 month after the boost vaccination (whichever takes longer). If the social situation of a female subject changes after start of the study (eg, female subject who is not heterosexually active becomes active), she must begin a highly effective method of birth control, as described above in Sections 4.1 and 4.2, until at least 3 months after the prime vaccination or up to 1 month after the boost vaccination (whichever takes longer).* Female subjects of childbearing potential participating in the Cohort 1 substudy will be asked to use adequate birth control measures for sexual intercourse from at least 14 days before to at least 3 months after the third vaccination.

Note: A period of 3 months after vaccination with Ad26.ZEBOV and 1 month after vaccination with MVA-BN-Filo should be respected, whichever takes longer.

Note: Prior to each study vaccine administration, a urine pregnancy test should be performed for female subjects of childbearing potential.

2. Criterion modified per Amendment 3

2.1. Criterion modified per Amendment 2

2.2. Male subjects who did not have a vasectomy performed more than 1 year prior to screening and who are sexually active with a female subject of childbearing potential must use condoms for sexual intercourse until at least 3 months after the prime vaccination or up to 1 month after the boost vaccination (whichever takes longer).* Male subjects participating in the Cohort 1 substudy who did not have a vasectomy performed more than 1 year prior to the third vaccination and who are sexually active with a female subject of childbearing potential must use condoms for sexual intercourse until at least 3 months after the third vaccination.

***After unblinding (see Sections 3.1 and 11 for details), subjects who received placebo may stop birth control measures or resume their pre-study birth control measures.**

3. Criterion modified per Amendment 3

3.1. Women should not breast-feed while enrolled in the study until at least 3 months after the prime vaccination or up to 1 month after the boost vaccination (whichever takes longer). Women participating in the Cohort 1 substudy should not breast-feed while enrolled in the study until at least 3 months after the third vaccination.

4. Criterion modified per Amendment 3

4.1. Criterion modified per Amendment 2

4.2. Traveling to epidemic Ebola areas is prohibited while enrolled in the study from the start of screening onwards until the 42-day post-last vaccination (excluding third vaccination) or 21-day post third vaccination visit. Subjects who subsequently travel to these areas should have returned at least 1 month before the long-term follow-up visits (6 months post-last vaccination and Day 365 [1 year post third vaccination for Cohort 1 substudy]). Any traveling to epidemic Ebola areas should be documented in the case report form (CRF).

Note: Subjects travelling to epidemic Ebola areas will be excluded from follow-up collection of blood for immunogenicity assessments if they contract Ebola virus disease (see also exclusion criterion #2 and #23 in Sections 4.2.1 and 4.2.3, respectively).

5. Subjects should not use any disallowed therapies as described in Section 8.

5. TREATMENT ALLOCATION AND BLINDING

Vaccination Schedule Allocation

Based on the subject's demographics, a subject will be enrolled in one of the cohorts at study entry. Central randomization will be implemented in this study. In Cohort 1, subjects will be enrolled in parallel and randomized in a 1:1:1 ratio to Groups 1, 2 and 3 at baseline until a target of 132 subjects have been included in Group 3. Afterwards, randomization in this cohort will proceed in a 1:1 ratio to Groups 1 and 2. In Cohorts 2a, 2b, and 3, subjects will be enrolled in parallel and randomized in a 1:1 ratio to Groups 1 and 2 at baseline. Subjects will be randomly assigned to groups within cohorts (stratified by PBMC sampling capability of the selected sites), and within groups and age strata randomly assigned to Ad26.ZEBOV and MVA-BN-Filo, or placebo in a 5:1 ratio, based on a computer-generated randomization schedule prepared before the study by or under the supervision of the sponsor. The randomization within each group will be balanced by using randomly permuted blocks. Within Cohort 1, there will be stratification by age group (adults: ≥18 to ≤50 years of age, versus elderly: >50 years of age). The interactive web response system (IWRS) will assign a unique code, which will dictate the assignment and matching vaccination schedule for the subject. The requestor must use his or her own user identification and personal identification number when contacting the IWRS, and will then give the relevant subject details to uniquely identify the subject. For participants of the substudy, subjects who received Ad26.ZEBOV and MVA-BN-Filo will receive Ad26.ZEBOV as third vaccination. Subjects who received placebo will receive placebo as third vaccination.

Blinding

For each cohort, subjects and study-site personnel will be blinded to the study vaccine allocation within groups until the last subject in that cohort has completed the study. A subject will be considered to have completed the study if he or she has completed all assessments at the 6-month post-boost vaccination visit or at the Day 365 (1-year post third vaccination for Cohort 1 substudy) visit, whichever occurs later. Qualified study-site personnel with primary responsibility for study vaccine preparation and dispensing, and not involved in any other study-related procedure will be unblinded to the study vaccine allocation. The study vaccines will be administered by a blinded study vaccine administrator (see Definitions of Terms).

For each cohort, sponsor personnel will be blinded to study vaccine allocation within groups until the last subject in that cohort has completed the 6-month post-boost visit or discontinued earlier. All subjects within a cohort will be followed in a blinded manner by the site until the last subject in that cohort has completed the study. Any subject who completed either the 6-month post-prime and/or the Day 365 visit prior to approval of Amendment 2 was required to attend the 6-month post-boost and the Day 365 visit after approval of Amendment 2. Refer to Sections 3.1 and 11 for further details on unblinding and primary and interim analyses.

The investigator will not be provided with randomization codes. The codes will be maintained within the IWRS, which has the functionality to allow the investigator to break the blind for an individual subject.

Data that may potentially unblind the study vaccine assignment (ie, study vaccine preparation/accountability data, or other specific laboratory data) will be handled with special care to ensure that the integrity of the blind is maintained and the potential for bias is minimized. This can include making special provisions, such as segregating the data in question from view by the investigators, clinical team, or others as appropriate until the time of database lock and unblinding. The pharmacy and preparation of study vaccines will be monitored by an independent study vaccine monitor (see Section 17.8).

Under normal circumstances, the blind for the sponsor should not be broken within a cohort until the last subject in that cohort has completed the 6-month post-boost visit or discontinued earlier. Otherwise, the blind should be broken only if specific emergency treatment/course of action would be dictated by knowing the treatment status of the subject. In such cases, the investigator may in an emergency determine the identity of the study vaccine by contacting the IWRS. It is recommended that the investigator contacts the sponsor or its designee if possible to discuss the particular situation, before breaking the blind. Telephone contact with the sponsor or its designee will be available 24 hours per day, 7 days per week. In the event the blind is broken, the sponsor must be informed as soon as possible. The date and reason for the unblinding must be documented by the IWRS, in the appropriate section of the CRF, and in the source document. The documentation received from the IWRS indicating the code break must be retained with the subject's source documents in a secure manner.

If the randomization code is broken by the investigator or the study-site personnel, the subject must discontinue further study vaccine administration and must be followed as appropriate (see

Section 10.2 for details). If the randomization code is broken by the sponsor for safety reporting purposes, the subject should not discontinue further study vaccine administration and may remain in the study (if the randomization code is still blinded to the study-site personnel and the subject).

6. DOSAGE AND ADMINISTRATION

An overview of the study vaccination schedules is provided in Table 1.

Table 1: Study Vaccination Schedules Groups 1-3

Cohort	N (per group)		Prime (D1)	Boost (D29, D57 or D85)
1	Healthy adult and elderly subjects (18-70 years inclusive)			
	264 (Groups 1 & 2)	220	Ad26.ZEBOV 5x10^{10} vp	MVA-BN-Filo 1x10^{8} Inf U
		44	placebo	placebo
	132 (Group 3)	110	Ad26.ZEBOV 5x10^{10} vp	MVA-BN-Filo 1x10^{8} Inf U
		22	placebo	placebo
2a	HIV-infected subjects (18-50 years inclusive)			
	66	55	Ad26.ZEBOV 5x10^{10} vp	MVA-BN-Filo 1x10^{8} Inf U
		11	placebo	placebo
2b	Adolescents (12-17 years inclusive)			
	66	55	Ad26.ZEBOV 5x10^{10} vp	MVA-BN-Filo 1x10^{8} Inf U
		11	placebo	placebo
3	Children (4-11 years inclusive)			
	66	55	Ad26.ZEBOV 5x10^{10} vp	MVA-BN-Filo 1x10^{8} Inf U
		11	placebo	placebo

Inf U: infectious units; N: number of subjects per group to receive study vaccine; vp: viral particles

Table 2: Cohort 1 Substudy Vaccination Schedule (Groups 1-2, Selected Sites)

Cohort	N (total Groups 1-2)	Third Vaccination (at least 1 year post prime)
1	Healthy adult and elderly subjects (18-70 years inclusive)	
	90*	Ad26.ZEBOV 5x10^{10} vp
		placebo

N: total number of subjects in Groups 1 and 2 to receive study vaccine; vp: viral particles
* Subjects who received Ad26.ZEBOV and MVA-BN-Filo will receive Ad26.ZEBOV as third vaccination. Subjects who received placebo will receive placebo as third vaccination.

6.1. General Instructions and Procedures

All subjects will receive a vaccination, according to randomization, on Day 1 (Groups 1 to 3) and on Day 29 (Group 1), Day 57 (Group 2) or Day 85 (Group 3) at the following dose levels:

- Ad26.ZEBOV: 5x10^{10} vp, supplied in a single use vial (0.5 mL extractable).
- MVA-BN-Filo: 1x10^{8} Inf U (nominal titer; target fill is 1.9x10^{8} Inf U per dose, range: 1.27-2.67x10^{8} Inf U), supplied in a single use vial (0.5 mL extractable).
- Placebo: 0.9% saline (0.5 mL extractable).

At selected sites, approximately 90 healthy adult subjects in Cohort 1 (Groups 1 and 2) will receive a third vaccination with Ad26.ZEBOV at $5x10^{10}$ vp or placebo at least 1 year post prime vaccination (substudy).

Study vaccines (Ad26.ZEBOV, MVA-BN-Filo or placebo) will be administered as 0.5-mL IM injections into the deltoid muscle by a blinded study vaccine administrator (see Definitions of Terms). In each subject, the boost vaccination should preferably be administered in the opposite deltoid from the prime vaccination (unless local site reaction cannot be assessed reliably in the opposite arm) and it should be recorded in the CRF in which arm the vaccination has been administered. The injection site should be free from any injury, local skin problem, significant tattoo or other issue that might interfere with evaluating the arm after injection (eg, subjects with a history of skin cancer must not be vaccinated at the previous tumor site). No local or topical anesthetic will be used prior to the injection. The third vaccination can be administered in either deltoid.

Discontinuation of study vaccine administration should occur in any subject meeting the criteria outlined in Section 10.2. Criteria for postponement of vaccination and contraindications to boost and third vaccinations have been defined in Sections 6.2 and 6.3, respectively. Refer to Section 9.3.2 for details on the pre-specified pausing rules to halt vaccination of further subjects.

Subjects will remain at the site for a total of 60 (±15) minutes (after prime and boost vaccinations) or 30 (±15) minutes (after the third vaccination) post-vaccination to monitor for the development of any acute reactions, or longer if deemed necessary by the investigator (eg, in case of grade 3 adverse events). As with any vaccine, allergic reactions following vaccination with the study vaccine are possible. Therefore, appropriate drugs and medical equipment to treat acute anaphylactic reactions must be immediately available and a medically qualified member of study-site personnel trained to recognize and treat anaphylaxis must be present in the clinic during the entire vaccination procedure and post-vaccination monitoring period.

The investigator must provide emergency care as needed for any subject who experiences a life-threatening event. All sites will have facilities, equipment and the ability to manage an anaphylactic reaction. If additional therapy is required, the investigator will arrange for transport to the closest appropriate facility for continuing care.

The Site Investigational Product Procedures Manual specifies the maximum time that will be allowed between preparation and administration of the study vaccine.

6.2. Criteria for Postponement of Vaccination

A subject will not be given the prime, boost, or third (Cohort 1 substudy only) vaccination if he/she experiences any of the following events at the scheduled time for vaccination:

- Acute illness at the time of vaccination (this does not include minor illnesses such as diarrhea or mild upper respiratory tract infection);

  Note: If a subject is acutely ill with malaria at the time of either prime, boost, or third vaccination, at least 3 days must elapse from the conclusion of a standard, recommended course of therapy for malaria before vaccination.

- Fever (body temperature ≥38.0°C) at the time of vaccination.

Subjects experiencing any of the events described above may be vaccinated up to 10 days beyond the window allowed for the scheduled vaccination, or be withdrawn from that vaccination at the discretion of the investigator and after consultation with the sponsor (see Section 10.2).

Note: In case the boost or third vaccination is postponed, the timing of the post-boost or post third vaccination visits will be planned relative to the actual vaccination day (see Sections 9.1.1 and 9.1.7).

6.3. Contraindications to Boost and Third Vaccinations

A subject will not be given the boost or third vaccination if he or she experiences any of the following events at any time after the prime or boost vaccination, respectively:

1. Anaphylaxis clearly attributable to vaccination with study vaccine; *OR*
2. Generalized urticaria within 72 hours of vaccination considered to be at least possibly related to study vaccine; *OR*
3. A serious adverse event considered to be at least possibly related to study vaccine; *OR*
4. Injection site ulceration, abscess or necrosis considered to be at least possibly related to the study vaccine; *OR*
5. Any other safety concern threatening the subject's safety or persisting clinically significant abnormality considered to be related to prime vaccination.

Subjects experiencing any of the events described above must not receive any further study vaccine, but should be monitored for safety and for immunogenicity according to the protocol as described in Section 10.2.

An ad hoc IDMC meeting may be requested via the sponsor for any single event or combination of multiple events which are considered to jeopardize the safety of the subjects.

7. TREATMENT COMPLIANCE

All study vaccines will be administered on site by a blinded study vaccine administrator (see Definitions of Terms). The date and time of each study vaccine administration will be recorded in the CRF.

8. PRESTUDY AND CONCOMITANT THERAPY

Prestudy therapies administered up to 30 days prior to the start of screening and previous vaccinia/smallpox vaccination at any time prior to study entry must be recorded in the CRF.

Concomitant therapies must be recorded from screening onwards until 42 days after the last vaccination (excluding third vaccination). For subjects in the Cohort 1 substudy, concomitant therapies should be recorded again from the day of the third vaccination until 28 days post third vaccination. Thereafter, concomitant therapies are only to be recorded if given in conjunction with serious adverse events and IREs that meet the criteria outlined in Sections 12.3.2 and 12.3.3, respectively. Any vaccines received during the study should be recorded.

All therapies (prescription or over-the-counter medications, including vaccines, vitamins, herbal supplements; non-pharmacologic therapies such as electrical stimulation, acupuncture, special diets, exercise regimens) must be recorded in the CRF. Recorded information will include a description of the type of the drug, treatment period, dosing regimen, route of administration, and its indication.

Subjects must use adequate birth control measures prior to randomization as described in Section 4.

Subjects are allowed to receive all routine immunizations (including rotavirus for pediatric subjects) according to local vaccination schedules, taking into account the following restrictions:

- Routine immunizations with inactivated vaccines (including rotavirus for pediatric subjects) should be administered at least 15 days before or after administration of any study vaccine to avoid any potential interference in efficacy of the routine immunizations or the interpretation of immune responses to study vaccines, as well as to avoid potential confusion with regard to attribution of adverse reactions.
- Routine immunizations with live attenuated vaccines are prohibited in the period from 30 days before baseline (Day 1) to 30 days after the last vaccination.

However, if a vaccine is indicated in a post-exposure setting (eg, rabies or tetanus), it must take priority over the study vaccine.

Analgesic/antipyretic medications and nonsteroidal anti-inflammatory drugs may be used post-vaccination only in case of medical need (eg, body temperature ≥38.0°C or pain) and their use must be documented. Use of these medications as routine prophylaxis prior to study vaccine administration is prohibited.

Chronic or recurrent use of medications that modify the host immune response (eg, cancer chemotherapeutic agents, systemic corticosteroids, immunomodulators) are prohibited.

HIV-infected subjects must be on a stable HAART regimen for at least 4 weeks prior to screening. These medications must be documented in the concomitant medication form as a prior medication and any changes to their regimen must also be recorded.

The sponsor must be notified in advance (or as soon as possible thereafter) of any instances in which prohibited therapies are administered. Prohibited therapies will be captured as protocol deviations.

9. STUDY EVALUATIONS

9.1. Study Procedures

Refer to Section 9.1.6 for the procedures to be followed in case of a study pause and to Section 9.1.7 for procedures to be followed for subjects participating in the Cohort 1 substudy.

9.1.1. Overview

The Time and Events Schedule summarizes the frequency and timing of safety, tolerability and immunogenicity measurements and evaluations applicable to this study. Details for all study procedures are provided in the following sections. Additional unscheduled study visits may be required if in the investigator's opinion, further clinical or laboratory evaluation is needed.

Visit Windows

The screening visit has to be performed within 8 weeks prior to the baseline visit (ie, the day of the subject's prime vaccination, Day 1). If a subject did not receive study vaccine on the planned day of vaccination, the timings of the next visits post-vaccination (see Time and Events Schedule) will be determined relative to the actual day of vaccination. Visit windows that will be allowed are summarized in Table 3. The subject should be encouraged to come within these time windows.

Table 3: Visit Windows

Visit Description	Day	Window
Seven Days Post-prime Vaccination	Day 8 for all groups	±2 days
Fourteen Days Post-prime Vaccination (only in Groups 2 and 3 from Cohort 1)	Day 15 for Group 2 and Group 3	±2 days
Boost Vaccination	Day 29 for Group 1<br>Day 57 for Group 2<br>Day 85 for Group 3	±3 days
Seven Days Post-boost Vaccination	Day 36 for Group 1<br>Day 64 for Group 2<br>Day 92 for Group 3	±2 days
Twenty-one Days Post-boost Vaccination	Day 50 for Group 1<br>Day 78 for Group 2<br>Day 106 for Group 3	±3 days
Forty-two Days Post-boost Vaccination	Day 71 for Group 1<br>Day 99 for Group 2<br>Day 127 for Group 3	±3 days
Follow-up 6 Months Post-boost[a]	Day 209 for Group 1<br>Day 237 for Group 2<br>Day 265 for Group 3	±15 days
Follow-up 1 Year Post-prime	Day 365 for all groups	±30 days

Refer to Table 6 (Section 9.1.7) for the visit windows that will be allowed for the Cohort 1 substudy.

[a] Subjects who completed the 6-month post-prime visit (which has been replaced with the 6-month post-boost visit per protocol Amendment 2) prior to approval of Amendment 2 were required to also attend the 6-month post-boost visit after approval of Amendment 2.

Blood Sampling Volumes

Approximately 200 to 400 mL of blood (including PBMC samples) will be drawn from adults and adolescents (12-17 years of age inclusive) over a period of 1 year, and remains well below the limits of standard blood donation.

For children (<12 years), the study-related blood volumes obtained (including any losses during phlebotomy) will not exceed 3% of the total blood volume during a period of 4 weeks. In order to meet the planned safety, serologic and cellular endpoints, it might be necessary to slightly exceed the guideline of 1% of the total blood volume at any single time. The sites will be instructed that the procedures should preferably be performed in the following order, according to their importance: safety, serology and cellular. The total volume of blood is estimated at 80 to 90 mL/kg body weight; 3% is 2.4 mL blood per kg body weight.[9] The allowable blood volume calculations are based on the 10th percentile for growth charts for 4- to 11-year-old children.[5]

For details on the approximate blood sampling volumes collected by visit and the cumulative blood volumes in Cohorts 1 and 2, refer to the Time and Events Schedule. For Cohort 3, the approximate blood sampling volumes collected by visit and the cumulative blood volumes for the immunogenicity assessments are provided in the Time and Events Schedule, while the site's Standard Operating Procedure will be followed with regard to the blood volumes for the clinical laboratory assessments.

Additional serum or urine pregnancy tests may be performed, as determined necessary by the investigator or required by local regulation, to establish the absence of pregnancy at any time during the subject's participation in the study.

Repeat or unscheduled samples may be taken for safety reasons or for technical issues with the samples.

9.1.2. Screening Phase

Up to 8 weeks before baseline (the day of the subject’s prime vaccination, Day 1) and after signing and dating the ICF and/or assent (see Section 16.2.3), screening assessments will be performed as indicated in the Time and Events Schedule. Screening may be split into multiple days or visits. In exceptional cases, the screening phase can be extended if discussed with and approved (documented) by the sponsor, eg, if not all the test results become available during the allocated 8 weeks; this will be evaluated on a case-by-case basis.

For male subjects and female subjects of non-childbearing potential, there will be no minimum duration of the screening period and it will last only for the time required to verify eligibility criteria. For female subjects of childbearing potential, it should be confirmed that adequate birth control measures were used from at least 28 days before the prime vaccination with a negative serum β-hCG pregnancy test at screening and a negative urine test immediately prior to each study vaccination (see Section 4). All male and female subjects, except for female subjects of non-childbearing potential, will be asked to use adequate birth control for sexual intercourse until at least 3 months after the prime vaccination or up to 1 month after the boost vaccination (whichever takes longer) (see Section 4.3).

Only subjects complying with the criteria specified in Section 4 will be included in the study. The investigator will provide detailed information on the study to the subject and will obtain written informed consent/assent prior to each subject’s study participation. The procedures indicated in the Time and Events Schedule will only be performed after the subject’s written informed consent/assent has been obtained.

After reading but before signing the ICF/assent, the TOU will be administered. Subjects who fail may repeat the test twice (and have to pass the third time to be eligible). Subjects must pass the TOU before starting the screening procedures. The TOU is a questionnaire to document the subject’s understanding of the study (for details, see Section 16.1).

The following is performed to determine eligibility requirements as specified in the inclusion and exclusion criteria:

- Review of all inclusion and exclusion criteria;
- Review of medical history (including concomitant diseases) and demographics;
- Review of prestudy therapies (up to 30 days prior to the start of screening), previous vaccinia/smallpox vaccination if known (at any time prior to study entry; not applicable for Cohort 3) and concomitant therapies;

- Serum pregnancy test (for female subjects of childbearing potential);
- Blood sampling for hematology and chemistry (fasting or non-fasting);
- Urine sampling for urinalysis;
- Serology testing: HIV type 1 or type 2 (for Cohorts 1, 2b, and 3 only);
- CD4+ cell count for HIV-infected subjects;
- FSH assessment (for women >45 years of age with amenorrhea for less than 2 years or ≤45 years of age with amenorrhea for more than 6 months);
- ECG recording for subjects ≥18 years of age;
- Full physical examination (including height and body weight; a genitourinary examination is not required);
- Measurement of vital signs (blood pressure, pulse/heart rate, body temperature).

All adverse events and pregnancies will be collected from the time a signed and dated ICF is obtained.

The overall eligibility of the subject to participate in the study will be assessed once all screening values and results of any other required evaluations are available. Retesting of values (eg, safety laboratory) that lead to exclusion is allowed once using an unscheduled visit during screening to assess eligibility. The safety laboratory assessments are to be performed within 28 days prior to the prime vaccination (including Day 1 before vaccination) and may be repeated once if they fall outside this time window. If rescreening is required, all screening procedures (except TOU) should be repeated. Study subjects who qualify for inclusion will be contacted and scheduled for enrollment and prime vaccination within 8 weeks.

9.1.3. Vaccination Phase (Prime and Boost)

If eligible, the subject will come for the baseline visit (Day 1). The investigator should ensure that all enrollment criteria have been met during screening. If a subject's clinical status changes (including available laboratory results or receipt of additional medical records) after screening but before the prime vaccination (Day 1) such that the subject no longer meets all enrollment criteria, then the subject should be excluded from further participation in the study. If the initial laboratory sampling occurred more than 8 weeks before baseline (Day 1), sampling will need to be repeated.

Eligible subjects will be allocated (by central randomization) to a vaccination schedule as described in Section 5.

The subjects will be vaccinated as described in Section 6, with prime vaccinations administered at Day 1 for all groups, and boost vaccinations at Day 29 (Group 1), Day 57 (Group 2) or Day 85 (Group 3) unless any of the pre-specified criteria not to proceed with vaccination are met (refer to Sections 6.2, 6.3 and 10.2 for details) or if a pause for vaccination of further subjects has been installed (see Section 9.3.2).

Before each vaccination (prime and boost), a urine pregnancy test (for female subjects of childbearing potential), a targeted physical examination and measurements of vital signs will be performed and blood will be drawn for safety and immunogenicity assessments. Refer to Section 9.4 for further details on the immunogenicity evaluations.

Study vaccine will be prepared on-site by unblinded qualified study-site personnel not involved in any other study-related procedure who will place a blinding tape on the syringe to mask its content and send the vaccine to a blinded study vaccine administrator (see Definitions of Terms) for administration to the subject (see Section 14.3 for details). Refer to Section 6 for further details on dosage and administration and post-vaccination monitoring.

All adverse events, serious adverse events, IREs and pregnancies will be collected and documented on the CRF, together with the information on any concomitant medications. For reporting of IREs, refer to Section 12.3.3.

Upon discharge from the site, subjects will be provided with a diary, a thermometer, and a ruler to measure and record local solicited adverse events and body temperature. Subjects will also record solicited local and systemic adverse events (reactogenicity) in the diary in the evening after vaccination and then daily for the next 7 days at approximately the same time each day. Diaries should be completed at home by the subject (or parent, legal guardian or caregiver, as applicable). For illiterate subjects or those having difficulty completing the diary independently, the sites will make arrangements to have the diary completed according to their local practice. A modified diary is available for pre-verbal children. Subjects will be instructed to contact the investigator immediately in case they experience an unsolicited adverse event (not listed on the diary card) or for any severe (grade 3) solicited adverse event (listed on the diary card).

Subjects in Groups 2 and 3 from Cohort 1 will come for an additional visit on Day 15, and will have blood drawn for immunogenicity assessments.

In all groups, subjects will come to the site at 7 days after each vaccination as indicated in the Time and Events Schedule. The subject's diary will be reviewed by study-site personnel. The investigator will examine the injection site for occurrences of erythema, induration/swelling, pain/tenderness or itching at these visits in order to complete the relevant parts of the CRF. Blood samples will be taken for safety (all subjects, after each vaccination) and immunogenicity (Cohorts 1 and 2a only, after boost vaccination) evaluations.

9.1.4. Post-vaccination Phase

In all groups, subjects will come to the site at 21 and 42 days after the boost vaccination as indicated in the Time and Events Schedule. The investigator will examine the injection site for occurrences of erythema, induration/swelling, pain/tenderness or itching at these visits in order to complete the relevant parts of the CRF.

All adverse events, serious adverse events, IREs and pregnancies will be collected and documented on the CRF, together with the information on any concomitant medications. For reporting of IREs, refer to Section 12.3.3.

Subjects will also have blood drawn for immunogenicity assessments at the specified visits in the Time and Events Schedule. Refer to Section 9.4 for further details on the immunogenicity evaluations.

Subjects will be instructed to contact the investigator before the next visit (ie, 6 months post-boost) if they experience any adverse event or intercurrent illness that they perceive as relevant and/or can be possibly related to study vaccine in their opinion.

9.1.5. Long-term Follow-up

When all subjects in a cohort have completed their 6-month post-boost visit or discontinued earlier, that cohort will be unblinded to the sponsor. All subjects within a cohort will be followed in a blinded manner by the site until the last subject in that cohort has completed the study. Any subject who completed either the 6-month post-prime and/or the Day 365 visit prior to approval of Amendment 2 was required to attend the 6-month post-boost and the Day 365 visit after approval of Amendment 2.

Serious adverse event information and IREs will be collected until the end of the study, and concomitant therapies should only be recorded if given in conjunction with serious adverse events and IREs. Pregnancies will be reported until the end of the study. Subjects will have blood drawn for immunogenicity assessments at the 6-month post-boost and Day 365 visits. Refer to Section 9.4 for further details on the immunogenicity evaluations. Physical examinations will be performed as indicated in the Time and Events Schedule.

Approximately 90 healthy adult subjects in Cohort 1 (Groups 1 and 2, at selected sites) will participate in a substudy. Refer to Section 9.1.7 for procedures to be followed for subjects participating in the Cohort 1 substudy.

9.1.6. Procedures in Case of a Study Pause

A study pause can affect subjects that are either awaiting prime vaccination or boost vaccination. After approval is granted to restart the study, subjects who are awaiting prime vaccination and whose screening period is longer than the protocol-defined 8 weeks as a result of the study pause, will be allowed to rescreen once (following the screening procedures described in Section 9.1.2, excluding TOU). These subjects need to sign a new ICF/assent. Subjects that are rescreened due to the pause must have new safety laboratory assessments (including HIV, FSH [if applicable], ECG, hematology, biochemistry, urinalysis, vital signs and physical examination) within 28 days of the prime vaccination. The TOU does not need to be repeated. After screening, these subjects will follow the same study procedures as those subjects who were unaffected by the study pause (described in Sections 9.1.3 to 9.1.5).

Subjects who are outside the protocol-defined boost vaccination window due to the study pause may choose to receive a late boost vaccination, if allowed by the sponsor and/or the relevant oversight authorities. Visit windows for the subjects who elect to receive a late boost vaccination are summarized in Table 4. These subjects need to sign a specific ICF/assent. Subjects who agree

to the late boost vaccination will follow the same post-boost vaccination schedule as those subjects whose boost vaccination was unaffected by the study pause (see Section 9.1.4).

Note: For female subjects of childbearing potential, it should be confirmed that adequate birth control measures were used from at least 28 days before the prime vaccination **and boost vaccination,** with a negative serum β-hCG pregnancy test at screening and a negative urine test immediately prior to each study vaccination (see Section 4).

Table 4: Visit Windows for Subjects Who Elect to Receive a Late Boost Vaccination

Visit Description	Day	Window
Seven Days Post-prime Vaccination	Day 8 for all groups	±2 days
Fourteen Days Post-prime Vaccination (only in Groups 2 and 3 from Cohort 1)	Day 15 for Group 2 and Group 3	±2 days
Boost Vaccination		
Seven Days Post-boost Vaccination		±2 days
Twenty-one Days Post-boost Vaccination		±3 days
Forty-two Days Post-boost Vaccination		±3 days
Follow-up 6 Months Post-boost		±15 days
Follow-up 1 Year Post-prime	Day 365 for all groups	±30 days

Visit windows for the subjects who are not willing or not allowed to receive a late boost vaccination are summarized in Table 5. These subjects need to sign a specific ICF/assent. All post-prime vaccination visits need to be performed despite the interruption in dosing. After the 7-day post-prime and 14-day post-prime (only in Groups 2 and 3 from Cohort 1) visits, these subjects will be followed every 3 months after the prime vaccination for safety only (and immunogenicity on the 6-month and 1-year post-prime visits), until Day 365 (see Additional Time and Events Schedule for subjects who do not receive a boost vaccination because of a study pause). Information for these time points (except for the 6-month and 1-year post-prime visits) may be collected either by telephone contact, visit to the site or by a home visit, according to local practice. For subjects in Groups 2 and 3 from Cohort 1, failure to obtain the blood samples for immunogenicity assessments on the additional visit on Day 15 will be considered as a minor protocol deviation.

Table 5: Visit Windows for Subjects Who are Not Willing or Not Allowed to Receive the Late Boost Vaccination

Visit Description[a]	Day	Window
Seven Days Post-prime Vaccination	Day 8 for all groups	±2 days
Fourteen Days Post-prime Vaccination (only in Groups 2 and 3 from Cohort 1)	Day 15 for Group 2 and Group 3	±2 days
Three Months Post-prime Vaccination[b]	Day 90 for all groups	±30 days
Six Months Post-prime Vaccination	Day 180 for all groups	±30 days
Nine Months Post-prime Vaccination[b]	Day 270 for all groups	±30 days
One Year Post-prime vaccination	Day 365 for all groups	±30 days

[a] Subjects will be asked to return to the site approximately every 3 months after the prime vaccination for safety assessments, until the 1-year post-prime follow-up visit (note: the 3-month and 9-month post-prime visits may be conducted by best local practice, see footnote b). In case the subject is unable to return for these visits, this will not constitute a major protocol deviation and subjects will be considered lost to follow-up.

[b] The 3-month and 9-month post-prime visits may be conducted by best local practice: either by telephone contact, site visit or home visit.

Adverse events, whether serious or non-serious, will be collected at all visits from signing of the ICF onwards until 42 days after the last vaccination (ie, Day 42 post-prime vaccination for subjects who did not receive a boost vaccination). Thereafter, reporting will be limited to all serious adverse events and IREs up to the subject's last study-related procedure. Solicited local and systemic adverse events (reactogenicity, see below) will be reported by the subject until 7 days after each administration of study vaccine. Adverse events will be followed by the investigator as specified in Section 12. Subjects will be instructed to contact the investigator before the next visit (ie, 6 months post-boost visit, or 3 months post-prime visit for subjects who are not willing or not allowed to receive a late boost vaccination) if they experience any adverse event or intercurrent illness that they perceive as relevant and/or can be possibly related to study vaccine in their opinion.

Subjects will have blood drawn for immunogenicity assessments as indicated in the Time and Events Schedule and Additional Time and Events Schedule (for subjects who do not receive a boost vaccination because of a study pause). Refer to Section 9.4 for further details on the immunogenicity evaluations. Physical examinations and vital signs will be performed as indicated in the Time and Events Schedule and Additional Time and Events Schedule (for subjects who do not receive a boost vaccination because of a study pause).

9.1.7. Cohort 1 Substudy (Third Vaccination)

The Cohort 1 Substudy Time and Events Schedule summarizes the frequency and timing of safety, tolerability and immunogenicity measurements and evaluations applicable to this substudy. Details for all study procedures are provided in the following paragraphs. Additional unscheduled study visits may be required if in the investigator's opinion, further clinical or laboratory evaluation is needed.

Subjects who received a late boost vaccination or did not receive the boost vaccination at all due to a study pause will not be included in the Cohort 1 substudy.

Visit Windows

If a subject did not receive study vaccine on the planned day of vaccination, the timings of the next visits post-vaccination (see Cohort 1 Substudy Time and Events Schedule) will be determined relative to the actual day of vaccination. Visit windows that will be allowed are summarized in Table 6. The subject should be encouraged to come within these time windows.

Table 6: Visit Windows

Visit Description	Day[a]	Window
Third Vaccination (12-15m pp)	3rd Vaccination	
Four Days Post Third Vaccination	Day 5 Post 3rd Vaccination	±1 days
Seven Days Post Third Vaccination	Day 8 Post 3rd Vaccination	±2 days
Twenty-one Days Post Third Vaccination	Day 22 Post 3rd Vaccination	±3 days
Follow-up 6 Months Post Third Vaccination	Day 180 Post 3rd Vaccination	±15 days
Follow-up 1 Year Post Third Vaccination	Day 365 Post 3rd Vaccination	±30 days

[a] The day of administration of the third vaccination is Day 1 of the substudy. Similarly, D2 Post 3rd vaccination is the second day of the substudy, ie, 1 day after the third vaccination.

Blood Sampling Volumes

During the Cohort 1 substudy, approximately 80 mL (or 200 mL, in case optional PBMC samples are taken) of blood will be drawn over a period of 1 year, which remains well below the limits of standard blood donation.

For details on the approximate blood sampling volumes collected by visit and the cumulative blood volumes in the Cohort 1 substudy, refer to the Cohort 1 Substudy Time and Events Schedule.

Additional serum or urine pregnancy tests may be performed, as determined necessary by the investigator or required by local regulation, to establish the absence of pregnancy at any time during the subject's participation in the Cohort 1 substudy.

Repeat or unscheduled samples may be taken for safety reasons or for technical issues with the samples.

Subjects participating in the Cohort 1 substudy need to sign an additional ICF before the first substudy-related activity.

All male and female subjects participating in the Cohort 1 substudy, except for female subjects of non-childbearing potential, will be asked to use adequate birth control measures for sexual intercourse from at least 14 days before to at least 3 months after the third vaccination.

Before the third vaccination, a urine pregnancy test (for female subjects of childbearing potential), a targeted physical examination and measurements of vital signs will be performed. Subjects will have blood drawn for immunogenicity assessments as indicated in the Cohort 1 Substudy Time and Events Schedule. Refer to Section 9.4 for further details on the immunogenicity evaluations. Physical examinations will be performed as indicated in the Cohort 1 Substudy Time and Events Schedule.

Study vaccine will be prepared on-site by unblinded qualified study-site personnel not involved in any other study-related procedure who will place a blinding tape on the syringe to mask its content and send the vaccine to a blinded study vaccine administrator (see Definitions of Terms) for administration to the subject (see Section 14.3 for details). Refer to Section 6 for further details on dosage and administration and post-vaccination monitoring.

Adverse events, whether serious or non-serious, will be collected at all visits from the day of the third vaccination onwards until 28 days thereafter. Thereafter, reporting will be limited to all serious adverse events and IREs up to the subject's last study-related procedure. Solicited local and systemic adverse events (reactogenicity, see below) will be reported by the subject until 7 days after each administration of study vaccine. Adverse events will be followed by the investigator as specified in Section 12. Subjects will be instructed to contact the investigator before the next visit if they experience any adverse event or intercurrent illness that they perceive as relevant and/or can be possibly related to study vaccine in their opinion.

9.1.8. VAC52150 Vaccine Development Roll-over Study

Female subjects who became pregnant with estimated conception within 28 days after vaccination with MVA-BN-Filo (or placebo) or within 3 months after vaccination with Ad26.ZEBOV (or placebo) and children born to vaccinated female subjects who became pregnant with estimated conception within 28 days after vaccination with MVA-BN-Filo (or placebo) or within 3 months after vaccination with Ad26.ZEBOV (or placebo) (unless local regulations have additional requirements for follow-up) will be eligible for enrollment into the VAC52150 Vaccine Development Roll-over study for long-term surveillance (for a total of up to 60 months after the prime vaccination). After unblinding, only female subjects and the children of female subjects enrolled in this study who received Ad26.ZEBOV and/or MVA-BN-Filo will remain in the VAC52150 Vaccine Development Roll-over study for long-term safety surveillance. After unblinding, female subjects and the children of female subjects who received placebo and have already been enrolled into the VAC52150 Vaccine Development Roll-over study will be discontinued from further participation in the roll-over study.

9.2. Endpoints

Refer to Section 11.1 for an overview of safety and immunogenicity endpoints.

9.3. Safety Evaluations

9.3.1. Safety Assessments

The study will include the following evaluations of safety and tolerability as described below and according to the time points provided in the Time and Events Schedule. Any clinically significant abnormalities occurring from signing of the ICF onwards until 42 days after the last vaccination (excluding third vaccination), and again from the day of the third vaccination until 28 days thereafter for subjects in the Cohort 1 substudy, must be recorded on the Adverse Event section of the CRF. Thereafter, reporting will be limited to all serious adverse events and IREs. Any clinically significant abnormalities (including those persisting at the end of the study/early withdrawal) will be followed by the investigator until resolution or until a clinically stable endpoint is reached (see Section 12). For reporting of IREs, refer to Section 12.3.3.

The investigators, together with the sponsor's medical monitor, will be responsible for the safety monitoring of the study, and will halt vaccination of further subjects in case any of the pre-specified pausing rules described in Section 9.3.2 have been met. Further safety measures with regards to vaccination are described in Sections 6.2 and 6.3.

An IDMC will be appointed by the sponsor formed with recommendations from the Clinical Steering Committee before the start of the study to perform regular review of the safety data during the study. Details regarding the IDMC are provided in Section 11.8.

Adverse Events

All adverse events, whether serious or non-serious, will be collected at all visits from signing of the ICF onwards until 42 days after the last vaccination (excluding third vaccination), and again from the day of the third vaccination until 28 days thereafter for subjects in the Cohort 1 substudy. Thereafter, reporting will be limited to all serious adverse events and IREs up to the subject's last study-related procedure. Solicited local and systemic adverse events (reactogenicity, see below) will be reported by the subject until 7 days after each administration of study vaccine. Adverse events will be followed by the investigator as specified in Section 12.

Solicited Adverse Events

Solicited adverse events (see Definitions of Terms) are precisely defined events that subjects are specifically asked about and which are noted by subjects in the diary. The subjects will be closely observed by study-site personnel for the first 30 (±10) minutes after each administration of study vaccine and again at 60 (±15) minutes after prime and boost vaccinations, and any unsolicited, solicited local or systemic adverse events will be documented during this period. Upon discharge from the site, subjects will receive a diary, a thermometer and a ruler to measure body temperature and solicited local reactions. Subjects will be instructed to record solicited local and systemic adverse events in the diary in the evening after each administration of study vaccine and then daily for the next 7 days (until the 7-day post-vaccination visits) at approximately the same time each day to serve as a reminder to the subject for the next visit. On the 7-day post-vaccination visits, the diary needs to be completed on site before the subject leaves the site. The investigator should discuss the information from the diary with the subject,

document the relevant information in the clinic chart, and complete the relevant parts of the CRF as described in the CRF Completion Guidelines.

On-site and diary reported solicited adverse events will be captured on a separate CRF page as described in the CRF Completion Guidelines, in contrast to the unsolicited adverse events which will be reported on the Adverse Event page of the CRF. The investigator must record in the CRF his/her opinion concerning the relationship of the adverse event to study vaccine.

Solicited Local (Injection Site) Adverse Events

Subjects will also be instructed on how to note occurrences of erythema, induration/swelling (measured using the ruler supplied), pain/tenderness and itching at the injection site in the evening after each administration of study vaccine and then daily for the next 7 days in the diary at approximately the same time each day.

Solicited Systemic Adverse Events

Subjects will be instructed on how to record daily body temperature using a thermometer provided for home use. Subjects should record the body temperature in the evening after each vaccination and then daily for the next 7 days in the diary. Body temperature should be measured at approximately the same time each day. If more than one measurement is made on any given day, the highest value will be recorded in the CRF.

Subjects will also be instructed on how to note the following symptoms in the evening after each administration of study vaccine and then daily for the next 7 days in the diary at approximately the same time each day:

- Nausea/vomiting
- Headache
- Myalgia
- Arthralgia
- Fatigue/malaise
- Chills
- Fever

If a ***solicited local or systemic adverse event*** is not resolved the 7-day post-vaccination visit, the follow-up will be captured on the diary. The subject will be instructed to record the date of last symptoms and maximum severity in the diary after resolution.

Cardiac Events

In case any cardiac sign or symptom develops after the boost vaccination, the subject should be referred to a local cardiologist where the appropriate work-up which might include an ECG and/or troponin I assessments, if available, should be performed.

Clinical Laboratory Tests

Blood samples for serum chemistry and hematology, and for other tests (see below) and a urine sample for urinalysis will be collected at the time points indicated in the Time and Events Schedule. The investigator must review the laboratory results, document this review, and record any clinically relevant changes occurring during the study in the Adverse Event section of the CRF until 42 days after the last vaccination (excluding third vaccination), and again from the day

of the third vaccination until 28 days thereafter for subjects in the Cohort 1 substudy. Thereafter, only serious adverse events and IREs need to be recorded. The laboratory reports must be filed with the source documents. For reporting of IREs, refer to Section 12.3.3.

Approximate blood volume expected to be drawn per visit for clinical laboratory assessments:

- 10 mL for Cohorts 1, 2a and 2b. Note: in Cohort 2a (HIV-infected subjects), an additional 2 mL will be taken for monitoring of CD4+ cell count at screening, on Day 1, on the day of the boost vaccination and 42 days after the boost vaccination.
- 6 mL at screening and 4 mL at other visits for Cohort 3 (4 to 11 years of age). These blood volumes are approximate and the site should follow their own Standard Operating Procedures for phlebotomy in children.

The following tests will be performed by the local laboratory, unless otherwise specified:

- Hematology Panel
    - hemoglobin
    - hematocrit
    - red blood cell count
    - white blood cell count with differential
    - platelet count
- Serum Chemistry Panel
    - sodium
    - potassium
    - blood urea nitrogen
    - aspartate aminotransferase
    - alanine aminotransferase
    - total bilirubin
    - creatinine
    - FSH (in women >45 years of age with amenorrhea for less than 2 years or ≤45 years of age with amenorrhea for more than 6 months)
- Urinalysis – Dipstick
    - specific gravity
    - pH
    - glucose
    - protein
    - blood
    - ketones

In case of positive urinalysis dipstick results for >1+ protein or blood, the sediment will be examined microscopically (only RBC will be documented).

Additional clinical laboratory assessments to be performed are as follows:

- Serum pregnancy test for female subjects of childbearing potential at screening;
- Urine pregnancy test for female subjects of childbearing potential before each study vaccination;
- CD4+ cell count for HIV-infected subjects (Cohort 2a) at screening, on Day 1, on the day of the boost vaccination and at 42 days after the boost vaccination;
- Serology: HIV type 1 and type 2.

Laboratory values will be determined according to the Toxicity Table for Use in Trials Enrolling Healthy Adults (Attachment 2) or the Toxicity Table for Use in Trials Enrolling Children Greater Than 3 Months of Age (Attachment 3).

Electrocardiogram

A single, 12-lead ECG will be performed for subjects ≥18 years of age at screening and interpreted locally. Additional ECG monitoring may be performed at other time points if clinically indicated. During the collection of ECGs, subjects should be in a quiet setting without distractions (eg, television, cell phones). Subjects should rest in a supine position for at least 5 minutes before ECG collection and should refrain from talking or moving arms or legs. If blood sampling or vital sign measurement is scheduled at the same time point as ECG recording, the procedures should be performed in the following order: vital signs, ECG, blood draw.

Vital Signs (blood pressure, pulse/heart rate, body temperature)

Vital sign measurements will be performed at the time points indicated in the Time and Events Schedule. Blood pressure and pulse/heart rate measurements will be assessed (at rest) with a completely automated device. Manual techniques will be used only if an automated device is not available. Confirmatory measurements can be performed if inconsistent with a prior measurement.

Body temperature is preferably measured axillary. If the body temperature was measured at another site this needs to be captured in the CRF.

Physical Examination

A full physical examination including height and body weight will be performed at screening. Calculation of the body mass index (BMI; Cohorts 1 and 2) or growth percentiles according to CDC growth charts (Cohort 3; see Section 4.2.3), will be recorded in the CRF. At other visits, an abbreviated, symptom-directed physical examination will be performed as indicated based on any clinically relevant issues, clinically relevant symptoms and medical history. The symptom-directed physical examination may be repeated if deemed necessary by the investigator. Physical examinations will be performed by the investigator or by a designated medically-trained clinician.

A full physical examination includes the following: general appearance, eyes, ears, nose, throat, cardiovascular system, respiratory system, gastrointestinal system, and skin and mucous

membranes. A genitourinary exam is not required. A neurological and musculoskeletal examination as well as an examination of the lymph nodes will also be performed. An abbreviated, symptom-directed physical examination includes examination of the injection site(s), heart, lungs and abdomen.

The height should be measured barefooted at the screening visit. To obtain the actual body weight, subjects must be weighed lightly clothed.

9.3.2. Pausing Rules

The investigators and the sponsor's medical monitor will review the safety of enrolled subjects on an ongoing basis. The sponsor's medical monitor will be involved in all discussions and decisions. These pausing rules are not applicable for the third vaccination.

Any cohort

If any of the following events occur in any subject in any cohort who received at least one dose of study vaccine in the study (at any site), the site investigator will halt the vaccination of further subjects in all cohorts and the sponsor's medical monitor will be notified immediately. The sponsor's medical monitor will inform all the other investigators to halt further vaccination as well.

- Death in any subject, considered at least possibly related to the study vaccine; *OR*
- An anaphylactic reaction within 24 hours of vaccination or the presence of generalized urticaria within 72 hours of vaccination in any subject, considered at least possibly related to the study vaccine; *OR*
- A life-threatening or other serious adverse event in any subject, considered at least possibly related to the study vaccine.

Cohorts 1, 2 and 3

If any of the following events occur in any subject from Cohorts 1, 2 or 3 who received at least one dose of study vaccine in the study (across all sites), the sponsor's medical monitor will notify all investigators to halt vaccination of further subjects:

- Three or more subjects experience a severe (grade 3) (non-serious) unsolicited adverse event (of the same type) considered to be related to any of the study vaccines that persists for 3 or more days; *OR*
- Three or more subjects experience a persistent (upon repeat testing) severe (grade 3) (non-serious) abnormality (including unexplained hematuria) related to the same laboratory parameter and considered to be related to any of the study vaccines; *OR*
- Three or more subjects experience the same severe (grade 3) (non-serious) solicited systemic reaction considered to be related to any of the study vaccines that persists for 3 or more days (subjective systemic reaction corroborated by study personnel).

In case of occurrence of any of the events described above, the sponsor's medical monitor will notify the IDMC immediately. Dosing will be halted and health authorities will be notified. Within 3 business days, the IDMC will convene to review the available safety data as outlined in the charter and to discuss study suspension or discontinuation of further vaccination or to decide that vaccination may resume. The sites will be allowed to resume activities upon receipt of a written notification from the sponsor. The criteria for pausing will be re-set each time and the same criteria have to be met again to halt further vaccination.

9.4. Immunogenicity Evaluations

Venous blood samples will be collected for the determination of immune responses at the time points indicated in the Time and Events Schedule. Samples for assessment of humoral immune responses will be taken from all subjects. Samples for assessment of cellular immunity will be taken from subjects at selected sites with the capabilities to process PBMC (targeted at 165 subjects [138 Ad26.ZEBOV and/or MVA-BN-Filo, and 27 placebo] in Cohort 1 and 33 subjects [28 Ad26.ZEBOV and/or MVA-BN-Filo, and 5 placebo] in each of the other cohorts). Samples to assess cellular immune responses are optional for subjects in the Cohort 1 substudy and may only be collected in a subset of subjects. Blood volumes are presented in Table 7 below.

Sample collection and processing will be performed by the study-site personnel according to current versions of approved standard operating procedures. The Laboratory Manual contains further details regarding the collection, handling, labeling, and shipment of blood samples to the respective laboratories.

Table 7: Whole Blood Volumes for Immunogenicity Assessments

Cohort		Humoral Immune Response (mL)	Cellular Immune Response (mL)	Approximate Total Blood Volume for Immunogenicity Assays (mL)
1	Healthy adult and elderly subjects	10	40	200[b]/300/350[c]
	Cohort 1 substudy subjects	*10*	*40*[a]	*350*[d]*/360/400*[c,d]*/410*[c] *[430*[d]*/480/480*[c,d]*/530*[c]*]*
2a	HIV-infected subjects	10	40	300
2b	Adolescents (12-17 years inclusive)	10	20	150
3	Children (4-11 years inclusive)			
	• 9-, 10- and 11-year old:	5	10	65
	• 6-, 7- and 8-year old:	2.5	6	36.5
	• 4- and 5-year old:	2.5	4	28.5

[a] These samples are optional and may only be collected in a subset of subjects.
[b] Only for subjects who do not receive a boost vaccination because of a study pause.
[c] Subjects in Groups 2 and 3 from Cohort 1 have an extra visit on Day 15, to assess the immune response after prime vaccination.
[d] In case the pre-third vaccination blood sample coincides with the Day 365 blood sample.
* Approximate total blood volumes including optional PBMC samples.

The immunologic assessments and purposes are summarized in Table 8. The exploratory assay package may include, but might not be limited to, the listed assays.

Table 8: Summary of Immunologic Assessments

Sample	Purpose (non-exhaustive)
Serum, taken from all subjects (N=1,056)	- Analysis of antibodies binding to EBOV GP (ELISA), possibly using different EBOV GPs<br>- Analysis of neutralizing antibodies to EBOV GP (Virus neutralization assay), possibly using different EBOV GPs<br>- Binding and/or neutralizing antibodies to adenovirus and/or MVA (Adenovirus and/or MVA ELISA and/or neutralization assay)<br>- Analysis of anti-EBOV GP, filovirus GPs and/or TAFV NP antibody characteristics, including IgG subtyping (molecular antibody characterization)<br>- …
PBMC, taken from subjects at selected sites*	- T-cell IFN-γ responses to EBOV GP (ELISpot)<br>- Analysis of T-cell responses to EBOV GP, filovirus GPs and/or TAFV NP (including CD4/8, IL-2, IFN-γ, TNF-α and/or activation markers) (ICS)<br>- …

IgG: immunoglobulin G; ICS: intracellular cytokine staining; IFN: interferon; IL: interleukin; TNF: tumor necrosis factor
* (targeted at 165 subjects [138 Ad26.ZEBOV and/or MVA-BN-Filo, and 27 placebo] in Cohort 1 and 33 subjects [28 Ad26.ZEBOV and/or MVA-BN-Filo and 5 placebo] in each of the other cohorts). PBMC samples are optional for subjects in the Cohort 1 substudy and may only be collected in a subset of subjects.

9.5. Vaccine-induced Seropositivity

In general, uninfected subjects who participate in Ebola vaccine studies may develop Ebola-specific antibodies as a result of an immune response to the candidate Ebola vaccine, referred to as VISP. These antibodies may be detected in Ebola serologic tests, causing the test to appear positive even in the absence of actual Ebola infection. VISP may become evident during the study, or after the study has been completed. The potential of a study participant becoming PCR-positive after vaccination is being assessed in a Phase 1 study (VAC52150EBL1002).

Subjects should not donate blood during participation in the study (from the start of screening onwards; see Sections 4.1.2 and 4.2.3).

Consent will be obtained to contact the doctors that the subject sees regularly, to let them know that the subject is taking part in this study. It is important for all of the subject's doctors to know that the subject may be administered experimental vaccines. Subjects participating in the study will be provided with a "wallet (study) card" and instructed to carry this card with them for the duration of the study (see Section 12.3.1).

9.6. Sample Collection and Handling

The actual dates and times of sample collection must be recorded in the CRF or laboratory requisition form.

Refer to the Time and Events Schedule for the timing and frequency of all sample collections.

Instructions for the collection, handling, storage, and shipment of samples are found in the laboratory manual that will be provided. Collection, handling, storage, and shipment of samples must be under the specified, and where applicable, controlled temperature conditions as indicated in the laboratory manual.

10. SUBJECT COMPLETION/DISCONTINUATION OF STUDY VACCINE/WITHDRAWAL FROM THE STUDY

10.1. Completion

A subject will be considered to have completed the study if he or she has completed all assessments at the 6-month post-boost vaccination visit or at the Day 365 visit, whichever occurs later. A subject in the Cohort 1 substudy will be considered to have completed the study if he or she has completed all assessments at the 1-year post third vaccination visit.

10.2. Discontinuation of Study Vaccine/Withdrawal From the Study

Discontinuation of Study Vaccine

If a subject's study vaccine must be discontinued before the end of the vaccination schedule, this will not result in automatic withdrawal of the subject from the study.

A subject’s study vaccine (prime or boost) must be discontinued at the discretion of the investigator and after consultation with the sponsor for any of the events in Section 6.2.

A subject's study vaccine should be **permanently** discontinued if:

- The investigator believes that for safety reasons (eg, adverse event) it is in the best interest of the subject to discontinue study vaccine;
- The subject becomes pregnant;
- The subject has confirmed Ebola virus disease through natural exposure to the virus (eg, by travel to an affected country);
- The subject experiences any of the events described in Section 6.3;
- The randomization code is broken by the investigator or the study-site personnel.

Subjects meeting any of the reasons listed above must not receive any further study vaccine, but should continue to be monitored for safety and for immunogenicity according to the protocol if this does not result in safety risks for the subject. In case of early discontinuation of study vaccine due to an adverse event, the investigator will collect all information relevant to the adverse event and safety of the subject, and will follow the subject to resolution, or until reaching a clinically stable endpoint. In case of a study pause, subjects who were primed prior to the pause and did not yet receive a boost vaccination need to follow the visits as described in Table 5. Once they receive the late boost vaccination, these subjects need to follow the visits as described in Table 4.

Withdrawal From the Study

Each subject has the right to withdraw from the study at any time for whatever reason. The investigator should make an attempt to contact subjects who did not return for scheduled visits or follow-up. Although the subject is not obliged to give reason(s) for withdrawing early, the investigator should make a reasonable effort to ascertain the reason(s) while fully respecting the subject’s rights.

A subject will be withdrawn from the study for any of the following reasons:

- Decision by the investigator to withdraw a subject for repeated failure to comply with protocol requirements;
- Decision by the sponsor to stop or cancel the study;
- Decision by local regulatory authorities and Independent Ethics Committee (IEC)/Institutional Review Board (IRB) to stop or cancel the study;
- Lost to follow-up;
- Withdrawal of consent;
- Death.

If a subject withdraws early from the study for any of the reasons listed above (except in case of death), early withdrawal assessments should be obtained (ie, physical examination and

immunogenicity assessments for serum and PBMCs). A subject who wishes to withdraw consent from participation in the study will be offered an optional visit for safety follow-up (before formal withdrawal of consent), but the subject has the right to refuse.

If a subject is lost to follow-up, every reasonable effort must be made by the study site personnel to contact the subject and determine the reason for discontinuation/withdrawal. The measures taken to follow up must be documented.

When a subject withdraws before completing the study, the reason for withdrawal is to be documented in the CRF and in the source document. Study vaccine assigned to the withdrawn subject may not be assigned to another subject. For subjects who withdraw from the study after randomization but before the prime vaccination, an additional subject will be enrolled who will receive the same vaccination regimen as the withdrawn subject. Subjects who withdraw from the study after receiving the prime vaccination will not be replaced.

10.3. Withdrawal From the Use of Research Samples

A subject who withdraws from the study will have the following options for storage of samples for potential future use:

- The collected samples will be retained and used in accordance with the subject's original informed consent for storage of samples for future use.
- The subject may withdraw consent for storage of samples for potential future use (see Section 16.2.5), in which case the samples will be destroyed and no further testing will take place. To initiate the sample destruction process, the investigator must notify the sponsor study site contact of withdrawal of consent for the storage of leftover samples for future research and request sample destruction. The sponsor study site contact will, in turn, contact the biomarker representative to execute sample destruction. If requested, the investigator will receive written confirmation from the sponsor that the samples have been destroyed. Details of the sample retention for research are presented in the ICF.

11. STATISTICAL METHODS

Statistical analysis will be done by the sponsor or under the authority of the sponsor. A general description of the statistical methods to be used to analyze the safety and immunogenicity data is outlined below. Specific details will be provided in the Statistical Analysis Plan (SAP).

The primary analysis will be conducted when all subjects in Cohort 1 have completed the 6-month post-boost visit or discontinued earlier, to aid in the assessment of which vaccination schedule will be the best candidate for future studies and implementation in the field. Study-site personnel, subjects and sponsor personnel involved in subject level data review will remain blinded until the last subject in that cohort has completed the study. Sponsor personnel involved in the conduct of the primary analysis and in making future decisions for the program will be unblinded to the data of Cohort 1, but will remain blinded to the data of the other cohorts.

Interim analyses may be performed as described in Section 11.7.

The final analysis will be performed when all subjects have completed the last study-related visit or discontinued earlier (including subjects participating in the Cohort 1 substudy).

A general description of the statistical methods to be used to analyze the safety and immunogenicity data is outlined below.

Details on how to handle the data from subjects with a late boost will be described in the SAP, developed before unblinding.

Subjects who are outside the protocol-defined boost vaccination window due to a study pause and elect not to receive a late boost vaccination will not be replaced.

11.1. Endpoints

11.1.1. Primary Endpoints

- Adverse events, collected until the 42-day post-last vaccination (excluding third vaccination) visit.
- Serious adverse events and IREs, collected from signing of the ICF onwards until the end of the study.
- Solicited local and systemic adverse events (reactogenicity), collected until 7 days after each study vaccine administration.

11.1.2. Secondary Endpoint

Humoral immune response

- Binding antibody levels elicited by vaccination using EBOV GP ELISA at 21 days post boost.

Safety and tolerability (Cohort 1 substudy)

- Adverse events, collected from the day of the third vaccination onwards until 28 days thereafter (note: events that started before the third vaccination but are still present at the time of third vaccination should also be recorded).
- Serious adverse events and IREs, collected from signing of the original ICF onwards until the end of the study (including Cohort 1 substudy).

11.1.3. Exploratory Endpoints

Additional exploratory analyses may be performed to further investigate study vaccine-elicited immune responses. These may include, but might not be limited to, the following assays:

Humoral immune response

- Binding antibody levels elicited by vaccination using EBOV GP ELISA at other relevant time points (including Cohort 1 substudy).
- Neutralizing antibody responses against the EBOV GP defined as the serum titer that is able to inhibit viral infection by a certain percentage (IC_{50}, IC_{80} and/or IC_{90}) using virus neutralization assay.
- Binding and/or neutralizing antibody responses against the Ad26 and/or MVA vector, using adenovirus and/or MVA ELISA and/or neutralization assays.
- Humoral responses to different EBOV GPs and/or filovirus GPs and/or TAFV NP if assays are available.
- Molecular characterization of study vaccine-elicited antibodies which may include, but will not be limited to: Fc characterization, isotype analysis and epitope mapping.

Cellular immune response

- The presence and functional capacity of T cells may be determined using pathogen-specific stimulation of PBMC with EBOV GP peptides using IFN-γ ELISpot assays. Cytokine-producing T cells can be quantified using ELISpot technology.
- Activation of CD4+ and CD8+ T cell subsets and their cytokine expression patterns may be determined by flow cytometry after EBOV GP-specific stimulation (including, but not limited to, IFN-γ, interleukin [IL]-2, and tumor necrosis factor [TNF]-α) using intracellular cytokine staining (ICS). Exploratory phenotypic and functional analysis may be included.
- Cellular responses to filovirus GPs and/or TAFV NP if assays are available.

11.2. Sample Size Determination

An overall planned sample size of 1,056 subjects includes 880 subjects to receive Ad26.ZEBOV and MVA-BN-Filo prime-boost vaccination to substantially contribute to an overall safety data base of the Ad26.ZEBOV and MVA-BN-Filo prime-boost regimen.

In Cohort 1, a total of 550 subjects are planned to be vaccinated with Ad26.ZEBOV and MVA-BN-Filo across the vaccination schedules (220 in Groups 1 and 2, 110 in Group 3). In the other cohorts (2a, 2b, and 3), 55 subjects per vaccination schedule (Groups 1 and 2) are planned to be vaccinated with Ad26.ZEBOV and MVA-BN-Filo, or a total of 110 subjects per subpopulation.

The probability of detecting an adverse event in each of the populations, and total, is given in Table 9, for various true incidence rates. In case a specific adverse event is not observed, the one-sided 97.5% upper confidence limit of the true incidence rate of this adverse event is less than 6.5%, 3.3%, 1.7%, 0.8%, 0.7% and 0.5%, for sample sizes of 55, 110, 220, 440, 550 and 880 subjects, respectively.

Table 9: Probability of Observing at Least One Adverse Event Given a True Adverse Event Incidence

True Adverse Event Incidence (%)	N=55	N=110	N=220	N=440	N=550	N=880
0.1	5%	10%	20%	36%	42%	59%
0.5	24%	42%	67%	89%	94%	99%
1.0	42%	67%	89%	99%	100%	100%
2.5	75%	94%	100%	100%	100%	100%
5.0	94%	100%	100%	100%	100%	100%
10.0	100%	100%	100%	100%	100%	100%

Having immunogenicity data available for 55, 110 or 220 subjects per group on Ad26.ZEBOV or MVA-BN-Filo within a cohort, the following pairwise differences in immune response (ELISA antibody levels against EBOV GP) between vaccine schedules can be detected at any given time point, for a given variability in ELISA antibody levels (Table 10).

Table 10: Magnitude of Pairwise Difference to be Detected Between Vaccine Regimens for a Given Sample Size

Variability ELISA antibody levels (SD, log_{10} scale)	Detectable difference, log_{10} scale *		
	n1=n2=55	n3=110, n1,n2=220	n1=n2=220
0.3	0.19	0.13	0.11
0.4	0.25	0.18	0.14
0.5	0.31	0.22	0.18

SD: standard deviation

* 90% power, 5% significant level for 2-sided hypothesis, accounting for multiple testing in Cohort 1

11.3. Analysis Sets

The Full Analysis set includes all subjects who were randomized and received at least one dose of study vaccine, regardless of the occurrence of protocol deviations. Safety data will be analyzed based on the Full Analysis set.

The Immune Response Analysis set includes all randomized and vaccinated subjects, who have data from baseline and at least one post-vaccination immunogenicity blood draw.

The Per Protocol Analysis set for the main study includes all randomized and vaccinated subjects, who received both the prime and boost (administered not more than 10 days outside the visit window) vaccinations, have immunogenicity data from baseline and at least one post-vaccination evaluable immunogenicity sample, and have no major protocol violations influencing the immune response.

A similar Per Protocol Analysis set will be used for the Cohort 1 substudy for the time points after the third vaccination.

The immunogenicity analysis will be based on the Per Protocol Analysis set. As sensitivity analysis, the immunogenicity data will also be analyzed based on the Immune Response Analysis set, provided more than 10% of subjects in the Immune Response Analysis set are excluded from the Per Protocol Analysis set.

Details on how to handle the data from subjects with a late boost will be described in the SAP, developed before unblinding.

11.4. Subject Information

For all subjects, demographic characteristics (eg, age, height, weight, BMI, race, and sex) and screening/baseline characteristics (eg, physical examination, medical history) will be tabulated and summarized with descriptive statistics.

11.5. Safety Analyses

No formal statistical testing of safety data is planned. Safety data will be analyzed descriptively (including 95% confidence intervals, if applicable).

Similar safety summaries will be created for the subjects who received a third vaccination, details will be provided in the SAP.

Baseline for all safety parameters will be defined as the last value before the prime vaccination.

Adverse Events (Including Reactogenicity)

The verbatim terms used in the CRF by investigators to report adverse events will be coded using the Medical Dictionary for Regulatory Activities (MedDRA). All reported adverse events after vaccination (solicited local, solicited systemic, and unsolicited) will be included in the analysis. For each adverse event, the number and percentage of subjects who experience at least one occurrence of the given event will be summarized by group. Summaries, listings, datasets and/or subject narratives may be provided as appropriate, for those subjects who die, discontinue study vaccinations due to an adverse event, or experience a severe or serious adverse event. The analysis for solicited adverse events will be done on those subjects in the Full Analysis set for whom reactogenicity assessments are available in the database. The analysis of unsolicited adverse events will be done based on the Full Analysis set.

Physical Examination

Physical examination abnormalities following vaccination will be tabulated by most severe abnormality grade.

For Cohorts 1 and 2, BMI will be calculated using the recording of height at screening. Body weight and BMI results will be tabulated and summarized descriptively.

For Cohort 3, percentiles of weight and height (according to CDC growth charts, see Section 4.2.3) will be tabulated and summarized descriptively.

Vital signs

The percentage of subjects with values beyond clinically important limits will be summarized. Vital signs abnormalities following vaccination will be tabulated by most severe abnormality grade.

Clinical Laboratory Tests

Laboratory abnormalities will be determined according to the toxicity grading tables, and in accordance with the normal ranges of the clinical laboratory. The most severe laboratory abnormalities following vaccination will be tabulated per regimen.

11.6. Immunogenicity Analyses

Descriptive statistics (actual values and changes from baseline, including 95% confidence intervals, if applicable) will be calculated for continuous immunologic parameters by time point. Graphical representations of changes in immunologic parameters will be prepared, as applicable. Frequency tabulations will be calculated for discrete (qualitative) immunologic parameters by time point. In addition, differences between the vaccination schedules will be described at the 21-day post-boost, 6-month post-boost, and Day 365 visits. Similar summaries will be provided for the time points after the third vaccination for the Cohort 1 substudy population.

Details on the statistical analysis of the immunogenicity data and on the data for subjects who received a late boost vaccination will be provided in the SAP.

11.7. Interim Analyses

For the Cohort 1 substudy of subjects who received the third vaccination, an interim analysis may be performed when all subjects have completed the 6-month post third vaccination visit, or discontinued earlier.

In each cohort, after completion of the 6-month post-boost visit by all subjects, an interim analysis will be conducted on safety and selected immunogenicity data and the cohort will be unblinded to the sponsor.

A separate interim SAP will be prepared before the conduct of an interim analysis.

11.8. Independent Data Monitoring Committee

An IDMC will be established to monitor data on an ongoing basis to ensure the continuing safety of the subjects enrolled in this study. The committee will meet periodically to review interim data. Ad hoc IDMC meetings may be requested via the sponsor for any single event or combination of multiple events which are considered to jeopardize the safety of the subjects. After the review, the IDMC will make recommendations regarding the continuation of the study. The details will be provided in a separate IDMC charter.

The IDMC will be appointed by the sponsor with recommendations from the Clinical Steering Committee (see Section 11.9) before the start of the study. The IDMC will consist of at least one medical expert in the relevant therapeutic area and at least one statistician. The IDMC responsibilities, authorities, and procedures will be documented in its charter. Any safety reports/interim data from the cohorts in this study reviewed by the IDMC and the recommendations of the IDMC will be shared with the local health authorities and the IECs/IRBs.

11.9. Trial Management Team and Clinical Steering Committee

Oversight of the study will be conducted by the Trial Management Team which will consist of members from the sponsor as well as key collaborators from participating countries. This Trial Management Team will be responsible for protocol development and ensuring proper study execution. As part of the consortium agreement between the sponsor, INSERM and the London School of Hygiene and Tropical Medicine, a Clinical Steering Committee with representatives from each consortium partner has been established to guide the overall clinical development plan, including this study. The Clinical Steering Committee is responsible for overseeing the EBOVAC2 collaboration activities and for making decisions on specific issues.

12. ADVERSE EVENT REPORTING

Timely, accurate, and complete reporting and analysis of safety information from clinical studies are crucial for the protection of subjects, investigators, and the sponsor, and are mandated by regulatory agencies worldwide. The sponsor has established Standard Operating Procedures in conformity with regulatory requirements worldwide to ensure appropriate reporting of safety information; all clinical studies conducted by the sponsor or its affiliates will be conducted in accordance with those procedures.

12.1. Definitions

12.1.1. Adverse Event Definitions and Classifications

Adverse Event

An adverse event is any untoward medical occurrence in a clinical study subject administered a medicinal (investigational or non-investigational) product. An adverse event does not necessarily have a causal relationship with the treatment. An adverse event can therefore be any unfavorable and unintended sign (including an abnormal finding), symptom, or disease temporally associated with the use of a medicinal (investigational or non-investigational) product, whether or not related to that medicinal (investigational or non-investigational) product. (Definition per International Council for Harmonisation [ICH])

This includes any occurrence that is new in onset or aggravated in severity or frequency from the baseline condition, or abnormal results of diagnostic procedures, including laboratory test abnormalities.

Note: The sponsor collects adverse events starting with the signing of the ICF (refer to Section 12.3.1, for time of last adverse event recording).

Serious Adverse Event

A serious adverse event based on ICH and EU Guidelines on Pharmacovigilance for Medicinal Products for Human Use is any untoward medical occurrence that at any dose:

- Results in death
- Is life-threatening (The subject was at risk of death at the time of the event. It does not refer to an event that hypothetically might have caused death if it were more severe.)
- Requires inpatient hospitalization or prolongation of existing hospitalization
- Results in persistent or significant disability/incapacity
- Is a congenital anomaly/birth defect
- Is a suspected transmission of any infectious agent via a medicinal product
- Is Medically Important*

*Medical and scientific judgment should be exercised in deciding whether expedited reporting is also appropriate in other situations, such as important medical events that may not be immediately life-threatening or result in death or hospitalization but may jeopardize the subject or may require intervention to prevent one of the other outcomes listed in the definition above. These should usually be considered serious.

If a serious and unexpected adverse event occurs for which there is evidence suggesting a causal relationship between the study vaccine and the event (eg, death from anaphylaxis), the event must be reported as a suspected unexpected serious adverse reaction (SUSAR) (even after the study is over, if the sponsor, IDMC or investigator becomes aware of them) by the sponsor to the Health Authorities and by the investigator to the IEC/IRB according to regulatory and local requirements.

Unlisted (Unexpected) Adverse Event/Reference Safety Information

An adverse event is considered unlisted if the nature or severity is not consistent with the applicable product reference safety information. For Ad26.ZEBOV and MVA-BN-Filo, the expectedness of an adverse event will be determined by whether or not it is listed in the Investigator's Brochures and Addenda, if applicable.[14,15]

Adverse Event Associated with the Use of the Study Vaccine

An adverse event is considered associated with the use of the study vaccine if the attribution is **possibly, probably**, or **very likely** by the definitions listed in Section 12.1.2, Attribution Definitions.

Immediate Reportable Events

The following list of neuroinflammatory disorders are categorized as IREs, and should be reported to the sponsor within 24 hours of becoming aware of the event using the IRE Form. Relevant data from the IRE Form will be captured in the clinical database.

- Cranial nerve disorders, including paralyses/paresis (eg, Bell's palsy)
- Optic neuritis
- Multiple sclerosis
- Transverse myelitis
- Guillain-Barré syndrome, including Miller Fisher syndrome, Bickerstaff's encephalitis and other variants
- Acute disseminated encephalomyelitis, including site specific variants: eg, non-infectious encephalitis, encephalomyelitis, myelitis, myeloradiculomyelitis
- Myasthenia gravis and Lambert-Eaton myasthenic syndrome
- Immune-mediated peripheral neuropathies and plexopathies (including chronic inflammatory demyelinating polyneuropathy, multifocal motor neuropathy and polyneuropathies associated with monoclonal gammopathy).
- Narcolepsy
- Isolated paresthesia of >7 days duration

Symptoms, signs or conditions which might (or might not) represent the above diagnoses, should be recorded and reported as IREs even if the final or definitive diagnosis has not yet been determined, and alternative diagnoses have not yet been eliminated or shown to be less likely. Follow-up information and final diagnoses, if applicable, should be submitted as soon as they become available.

If the IRE is also serious (serious adverse event), it will be reported using the same process as for other serious adverse events.

12.1.2. Attribution Definitions

Every effort should be made by the investigator to explain any adverse event and to assess its potential causal relationship, ie, to administration of the study vaccine or to alternative causes, eg, natural history of underlying disease(s), concomitant drug(s). This applies to all adverse events, whether serious or non-serious. Assessment of causality must be done by a licensed study physician (the investigator or designee).

The investigator will use the following guidelines to assess the causal relationship of an adverse event to study vaccine:

Not Related

An adverse event that is not related to the use of the vaccine.

Doubtful

An adverse event for which an alternative explanation is more likely, eg, concomitant drug(s), concomitant disease(s), or the relationship in time suggests that a causal relationship is unlikely.

Possible

An adverse event that might be due to the use of the vaccine. An alternative explanation, eg, concomitant drug(s), concomitant disease(s), is inconclusive. The relationship in time is reasonable; therefore, the causal relationship cannot be excluded.

Probable

An adverse event that might be due to the use of the vaccine. The relationship in time is suggestive. An alternative explanation is less likely, eg, concomitant drug(s), concomitant disease(s).

Very Likely

An adverse event that is listed as a possible adverse reaction and cannot be reasonably explained by an alternative explanation, eg, concomitant drug(s), concomitant disease(s). The relationship in time is very suggestive.

12.1.3. Severity Criteria

Adverse events and laboratory data will be coded for severity using the toxicity grading tables in Attachment 2 and Attachment 3. For adverse events not identified in the table, the following guidelines will apply:

**Mild**	Grade 1	Symptoms causing no or minimal interference with usual social and functional activities
**Moderate**	Grade 2	Symptoms causing greater than minimal interference with usual social and functional activities
**Severe**	Grade 3	Symptoms causing inability to perform usual social and functional activities

Note: Only clinically significant abnormalities in laboratory data occurring from signing of the ICF onwards will be reported as adverse events and graded using the table above.

12.2. Special Reporting Situations

Safety events of interest on a sponsor study vaccine that may require expedited reporting or safety evaluation include, but are not limited to:

- Administration of an overdose of study vaccine
- Accidental or occupational exposure to a study vaccine
- Administration error involving a study vaccine (with or without subject/patient exposure to the study vaccine, eg, name confusion)
- IREs

Special reporting situations should be recorded in the CRF. For reporting of IREs, refer to Section 12.3.3. Any special reporting situation that meets the criteria of a serious adverse event should be recorded on the Serious Adverse Event page of the CRF.

12.3. Procedures

12.3.1. All Adverse Events

All adverse events and special reporting situations, whether serious or non-serious, will be reported from the time a signed and dated ICF/assent is obtained until the 42-day post-last vaccination visit (excluding third vaccination), and again from the day of the third vaccination until 28 days thereafter for subjects in the Cohort 1 substudy. Serious adverse events and IREs will be collected from signing of the ICF onwards until the end of the study. Subjects will be instructed to record solicited local and systemic adverse events (reactogenicity) in the diary in the evening after each vaccination and then daily for the next 7 days at approximately the same time each day.

Serious adverse events must be reported by the investigator using the Serious Adverse Event Form. SUSARs will be reported even after the study is over, if the sponsor, the IDMC or the investigator becomes aware of them. The sponsor will evaluate any safety information that is spontaneously reported by an investigator beyond the time frame specified in the protocol.

The investigator will monitor and analyze the study data including all adverse events and clinical laboratory data as they become available and will make determinations regarding the severity of the adverse experiences and their relation to study vaccine. All adverse events will be deemed related to study vaccine or not related to study vaccine, according to Section 12.1.2. To ensure that all adverse events are captured in a timely manner, the CRF will be entered in real-time and subjected to review to identify adverse events which may invoke pausing rules.

The investigator must review both post-injection reactogenicity and other adverse event CRF to insure the prompt and complete identification of all events that require expedited reporting as serious adverse events, invoke pausing rules or are other serious and unexpected events.

All adverse events, regardless of seriousness, severity, or presumed relationship to study vaccine, must be recorded using medical terminology in the source document and the CRF. Whenever possible, diagnoses should be given when signs and symptoms are due to a common etiology (eg, cough, runny nose, sneezing, sore throat, and head congestion should be reported as "upper respiratory infection"). Investigators must record in the CRF their opinion concerning the relationship of the adverse event to study therapy. All measures required for adverse event management must be recorded in the source document and reported according to sponsor instructions. For reporting of IREs, refer to Section 12.3.3.

The sponsor assumes responsibility for appropriate reporting of adverse events to the regulatory authorities. The sponsor will also report to the investigator (and the head of the investigational institute where required) all SUSARs. The investigator (or sponsor where required) must report SUSARs to the appropriate IEC/IRB that approved the protocol unless otherwise required and documented by the IEC/IRB. A SUSAR will be reported to regulatory authorities unblinded. Participating investigators and IEC/IRB will receive a blinded SUSAR summary, unless otherwise specified.

For all studies with an outpatient phase, including open-label studies, the subject must be provided with a "wallet (study) card" and instructed to carry this card with them for the duration of the study indicating the following:

- Study number
- Statement, in the local language(s), that the subject is participating in a clinical study and that results from Ebola virus serological screening tests should be interpreted with caution as the subject could have a false positive test (not truly infected with Ebola virus) as a result of an immune response to the candidate Ebola vaccine.
- Investigator's name and 24-hour contact telephone number
- Local sponsor's name and 24-hour contact telephone number (for medical staff only)
- Site number
- Subject number
- Any other information that is required to do an emergency breaking of the blind

12.3.2. Serious Adverse Events

All serious adverse events occurring during the study must be reported to the appropriate sponsor contact person by study-site personnel within 24 hours of their knowledge of the event.

Information regarding serious adverse events will be transmitted to the sponsor using the Serious Adverse Event Form, which must be completed and signed by a physician from the study site, and transmitted to the sponsor within 24 hours. The initial and follow-up reports of a serious adverse event should be made by facsimile (fax).

All serious adverse events that have not resolved by the end of the study, or that have not resolved upon discontinuation of the subject's participation in the study, must be followed until any of the following occurs:

- The event resolves
- The event stabilizes
- The event returns to baseline, if a baseline value/status is available
- The event can be attributed to agents other than the study vaccine or to factors unrelated to study conduct
- It becomes unlikely that any additional information can be obtained (subject or health care practitioner refusal to provide additional information, lost to follow-up after demonstration of due diligence with follow-up efforts)

Suspected transmission of an infectious agent by a medicinal product will be reported as a serious adverse event. Any event requiring hospitalization (or prolongation of hospitalization) that occurs during the course of a subject's participation in a study must be reported as a serious adverse event, except hospitalizations for the following:

- Hospitalizations not intended to treat an acute illness or adverse event (eg, social reasons such as pending placement in long-term care facility)
- Surgery or procedure planned before entry into the study (must be documented in the CRF)

  Note: Hospitalizations that were planned before the signing of the ICF, and where the underlying condition for which the hospitalization was planned has not worsened, will not be considered serious adverse events. Any adverse event that results in a prolongation of the originally planned hospitalization is to be reported as a new serious adverse event.

The cause of death of a subject in a study, whether or not the event is expected or associated with the study vaccine, is considered a serious adverse event.

12.3.3. Immediate Reportable Events

One subject in the study VAC52150EBL2001 experienced a serious and very rare condition called "Miller Fisher syndrome". This condition consists of double vision, pain on moving the eye, and difficulty with balance while walking. Miller Fisher syndrome most commonly occurs following a recent infection. The subject experienced these symptoms about a week after suffering from a common cold and fever. The event happened about a month after boost vaccination with either MVA-BN-Filo or placebo. This subject had to go to the hospital for treatment and has recovered. After an extensive investigation, the event has been considered to be doubtfully related to vaccine and most likely related to the previous common cold.

Any events of neuroimmunologic significance (listed in Section 12.1.1) should be categorized as IREs and should be reported throughout the study using the IRE form provided **within 24 hours to the sponsor**. Events suggestive of the disorders considered IREs should be reported even if the final diagnosis has not been yet determined, and follow-up information and final diagnosis should be submitted to the sponsor as soon as they become available.

If an event meets serious adverse event criteria (see above), it should be documented as such using the Serious Adverse Event Form, as well as the relevant CRF Adverse Event page and the complete IRE Form page 3 to be included as part of the Serious Adverse Event report.

12.3.4. Pregnancy

Pregnancies will be reported from signing of the ICF until the end of the study.

All initial reports of pregnancy in female subjects or partners of male subjects must be reported to the sponsor by the study-site personnel within 24 hours of their knowledge of the event using the appropriate pregnancy notification form. Abnormal pregnancy outcomes (eg, spontaneous abortion, fetal death, stillbirth, congenital anomalies, ectopic pregnancy) are considered serious adverse events and must be reported using the Serious Adverse Event Form. Any subject who

becomes pregnant during the study must be promptly withdrawn from further study vaccination but should continue participation in the study for safety follow-up.

Because the effect of the study vaccine on sperm is unknown, pregnancies in partners of male subjects included in the study will be reported as noted above.

Follow-up information regarding the outcome of the pregnancy and any postnatal sequelae in the infant will be required to be sent to the sponsor.

The parent(s)/legal guardian of children born to vaccinated female subjects who become pregnant with estimated conception within 28 days after vaccination with MVA-BN-Filo (or placebo) or within 3 months after vaccination with Ad26.ZEBOV (or placebo), will be approached to consent for enrollment of their children into the VAC52150 Vaccine Development Roll-over study (see Section 9.1.8).

12.4. Contacting Sponsor Regarding Safety

The names (and corresponding telephone numbers) of the individuals who should be contacted regarding safety issues or questions regarding the study are listed in the Contact Information page(s), which will be provided as a separate document.

13. PRODUCT QUALITY COMPLAINT HANDLING

A product quality complaint (PQC) is defined as any suspicion of a product defect related to manufacturing, labeling, or packaging, ie, any dissatisfaction relative to the identity, quality, durability, or reliability of a product, including its labeling or package integrity. A PQC may have an impact on the safety and efficacy of the product. Timely, accurate, and complete reporting and analysis of PQC information from studies are crucial for the protection of subjects, investigators, and the sponsor, and are mandated by regulatory agencies worldwide. The sponsor has established procedures in conformity with regulatory requirements worldwide to ensure appropriate reporting of PQC information; all studies conducted by the sponsor or its affiliates will be conducted in accordance with those procedures.

13.1. Procedures

All initial PQCs must be reported to the sponsor by the study-site personnel within 24 hours after being made aware of the event.

If the defect is combined with a serious adverse event, the study-site personnel must report the PQC to the sponsor according to the serious adverse event reporting timelines (refer to Section 12.3.2). A sample of the suspected product should be maintained for further investigation if requested by the sponsor.

13.2. Contacting Sponsor Regarding Product Quality

The names (and corresponding telephone numbers) of the individuals who should be contacted regarding product quality issues are listed in the Contact Information page(s), which will be provided as a separate document.

14. STUDY VACCINE INFORMATION

14.1. Description of Study Vaccines

Ad26.ZEBOV

Ad26.ZEBOV is a monovalent, replication-incompetent Ad26-based vector that expresses the full length EBOV Mayinga GP and is produced in the human cell line PER.C6.

The Ad26.ZEBOV vaccine will be supplied at a concentration of $1x10^{11}$ vp/mL in 2-mL single-use glass vials as a frozen liquid suspension to be thawed before use. Each vial contains an extractable volume of 0.5 mL. Refer to the Investigator's Brochure for a list of excipients.[14]

The Ad26.ZEBOV vaccine is manufactured by IDT Biologika GmbH for Janssen Vaccines & Prevention B.V., The Netherlands.

MVA-BN-Filo

MVA-BN-Filo is a recombinant multivalent vaccine intended for active immunization against Ebola and Marburg virus infection. MVA-BN-Filo is strongly attenuated; the vaccine is propagated in primary chicken embryo fibroblast cells and does not replicate in human cells.

The MVA-BN-Filo vaccine is supplied at a concentration of $2x10^8$ Inf U/mL (nominal titer) in 2-mL glass vials as a frozen liquid suspension to be thawed before use. Each vial contains an extractable volume of 0.5 mL. Refer to the Investigator's Brochure for a list of excipients.[15]

The MVA-BN-Filo vaccine is manufactured by IDT Biologika GmbH for Janssen Vaccines & Prevention B.V., The Netherlands.

Placebo

The placebo supplied for this study will be formulated as a sterile 0.9% saline for injection (as commercially available).

14.2. Packaging and Labeling

All study vaccines will be manufactured and packaged in accordance with Good Manufacturing Practice (GMP). All study vaccines will be packaged and labeled under the responsibility of the sponsor. No study vaccine can be repacked or relabeled on site without prior approval from the sponsor.

Further details for study vaccine packaging and labeling can be found in the Site Investigational Product Procedures Manual.

14.3. Preparation, Handling, and Storage

Study vaccine must be stored at controlled temperatures: Ad26.ZEBOV vials must be stored at ≤-65°C and MVA-BN-Filo vials must be stored at ≤–20°C.

Vials must be stored in a secured location with no access for unauthorized personnel. All study product storage equipment (including refrigerators, freezers), must be equipped with a continuous temperature monitor and alarm, and with back-up power systems. In the event that study vaccine is exposed to temperatures outside the specified temperature ranges, all relevant data will be sent to the sponsor to determine if the affected study vaccine can be used or will be replaced. The affected study vaccine must be quarantined and not used until further instruction from the sponsor is received.

Blinding will be achieved by preparation of study vaccine by unblinded qualified study-site personnel not involved in any other study-related procedure, and by the administration of vaccine in a masked syringe by a blinded study vaccine administrator (see Definitions of Terms).

Details on the preparation, the holding time and storage conditions from the time of preparation to administration of Ad26.ZEBOV and MVA-BN-Filo are provided in the Site Investigational Product Procedures Manual.

14.4. Study Vaccine Accountability

The investigator is responsible for ensuring that all study vaccine received at the site is inventoried and accounted for throughout the study. The study vaccine administered to the subject must be documented on the study vaccine accountability form. All study vaccine will be stored and disposed of according to the sponsor's instructions. Study-site personnel must not combine contents of the study vaccine containers.

Study vaccine must be handled in strict accordance with the protocol and the container label, and must be stored at the study site in a limited-access area or in a locked cabinet under appropriate environmental conditions. Unused study vaccine must be available for verification by the sponsor's study site monitor during on-site monitoring visits. The return to the sponsor of unused study vaccine will be documented on the study vaccine return form. When the study site is an authorized destruction unit and study vaccine supplies are destroyed on-site, this must also be documented on the study vaccine return form.

Potentially hazardous materials such as used ampules, needles, syringes and vials containing hazardous liquids, should be disposed of immediately in a safe manner and therefore will not be retained for study vaccine accountability purposes.

Study vaccine should be dispensed under the supervision of the investigator or a qualified member of the study-site personnel, or by a qualified staff member. Study vaccine will be supplied only to subjects participating in the study. Returned study vaccine must not be dispensed again, even to the same subject. Study vaccine may not be relabeled or reassigned for use by other subjects. The investigator agrees neither to dispense the study vaccine from, nor store it at, any site other than the study sites agreed upon with the sponsor.

15. STUDY-SPECIFIC MATERIALS

The investigator will be provided with the following supplies:

- Investigator's Brochures and Addenda (if applicable) for Ad26.ZEBOV and MVA-BN-Filo
- Site Investigational Product Procedures Manual
- Laboratory Manual
- IWRS Manual
- Electronic Data Capture (eDC) Manual/electronic CRF Completion Guidelines and Randomization Instructions
- Sample ICF
- Subject diaries
- TOU
- Rulers, thermometers
- Subject wallet cards
- Recruitment tools, as applicable

16. ETHICAL ASPECTS

16.1. Study-specific Design Considerations

Potential subjects will be fully informed of the risks and requirements of the study and, during the study, subjects will be given any new information that may affect their decision to continue participation. They will be told that their consent to participate in the study is voluntary and may be withdrawn at any time with no reason given and without penalty or loss of benefits to which they would otherwise be entitled. Only subjects who are fully able to understand the risks, benefits, and potential adverse events of the study, and provide their consent voluntarily will be enrolled.

When referring to the signing of the ICF, the terms legal guardian and legally acceptable representative refer to the legally appointed guardian of the child with authority to authorize participation in research. For each subject, his or her parent(s) (preferably both parents, if available) or legally acceptable representative(s), as required by local regulations, must give written consent (permission) according to local requirements after the nature of the study has been fully explained and before the performance of any study-related assessments. Assent should be obtained from children (minors) capable of understanding the nature of the study, typically subjects 7 years of age and older, depending on the local regulations and practice. For the purposes of this study, all references to subjects who have provided consent (and assent as applicable) refers to the subjects and his or her parent(s) or the subject's legal guardian(s) or legally acceptable representative(s) who have provided consent according to this process. Minors who assent to a study and later withdraw that assent should not be maintained in the study against their will, even if their parents still want them to participate.

Approximately 200 to 400 mL of blood (including PBMC samples) will be drawn from adults and adolescents (12-17 years of age inclusive) over a period of 1 year, and remains well below the limits of standard blood donation.

For children (<12 years), the trial-related blood volumes obtained (including any losses during phlebotomy) will not exceed 3% of the total blood volume during a period of 4 weeks. In order to meet the planned safety, serologic and cellular endpoints, it might be necessary to slightly exceed the guideline of 1% of the total blood volume at any single time. The sites will be instructed that the procedures should preferably be performed in the following order, according to their importance: safety, serology and cellular. The total volume of blood is estimated at 80 to 90 ml/kg body weight: 3% is 2.4 ml blood per kg body weight.[9] The allowable blood volume calculations are based on the 10th percentile for growth charts for 4- to 11-year-old children.[5]

Test of Understanding

The TOU is a short assessment of the subject's understanding of key aspects of the study (Attachment 1). The test will help the study staff to determine how well the subject understands the study and the requirements for participation.

The TOU must be completed after reading but before signing the ICF/assent by all subjects (as required per local regulations and practice, see Section 4), prior to enrollment into the study. The TOU is reviewed one-on-one with the subject and a member of the study team. Subjects are allowed to retake the test twice to achieve the passing score (≥90%) required for participation in the study. If a subject fails to achieve the passing score, further information and counseling will be provided by the study team member.

Any subject not capable of understanding the key aspects of the study, and their requirements for participation, should not be enrolled.

16.2. Regulatory Ethics Compliance

16.2.1. Investigator Responsibilities

The investigator is responsible for ensuring that the study is performed in accordance with the protocol, current ICH guidelines on Good Clinical Practice (GCP), and applicable regulatory and country-specific requirements.

GCP is an international ethical and scientific quality standard for designing, conducting, recording, and reporting studies that involve the participation of human subjects. Compliance with this standard provides public assurance that the rights, safety, and well-being of study subjects are protected, consistent with the principles that originated in the Declaration of Helsinki, and that the study data are credible.

16.2.2. Independent Ethics Committee or Institutional Review Board

Before the start of the study, the investigator (or sponsor where required) will provide the IEC/IRB with current and complete copies of the following documents (as required by local regulations):

- Final protocol and, if applicable, amendments
- Sponsor-approved ICF (and any other written materials to be provided to the subjects)
- Investigator's Brochure (or equivalent information) and amendments/addenda
- TOU
- Sponsor-approved subject recruiting materials
- Information on compensation for study-related injuries or payment to subjects for participation in the study, if applicable
- Investigator's curriculum vitae or equivalent information (unless not required, as documented by the IEC/IRB)
- Information regarding funding, name of the sponsor, institutional affiliations, other potential conflicts of interest, and incentives for subjects
- Any other documents that the IEC/IRB requests to fulfill its obligation

This study will be undertaken only after the IEC/IRB has given full approval of the final protocol, amendments (if any, excluding the ones that are purely administrative, with no consequences for subjects, data or study conduct, unless required locally), the ICF, applicable recruiting materials, and subject compensation programs, and the sponsor has received a copy of this approval. This approval letter must be dated and must clearly identify the IEC/IRB and the documents being approved.

During the study the investigator (or sponsor where required) will send the following documents and updates to the IEC/IRB for their review and approval, where appropriate:

- Protocol amendments (excluding the ones that are purely administrative, with no consequences for subjects, data or study conduct)
- Revision(s) to ICF and any other written materials to be provided to subjects
- If applicable, new or revised subject recruiting materials approved by the sponsor
- Revisions to compensation for study-related injuries or payment to subjects for participation in the study, if applicable
- New edition(s) of the Investigator's Brochure and amendments/addenda
- Summaries of the status of the study at intervals stipulated in guidelines of the IEC/IRB (at least annually)
- Reports of adverse events that are serious, unlisted/unexpected, and associated with the study vaccine

- New information that may adversely affect the safety of the subjects or the conduct of the study
- Deviations from or changes to the protocol to eliminate immediate hazards to the subjects
- Report of deaths of subjects under the investigator's care
- Notification if a new investigator is responsible for the study at the site
- Development Safety Update Report and Line Listings, where applicable
- Any other requirements of the IEC/IRB

For all protocol amendments (excluding the ones that are purely administrative, with no consequences for subjects, data or study conduct), the amendment and applicable ICF revisions must be submitted promptly to the IEC/IRB for review and approval before implementation of the change(s).

At least once a year, the IEC/IRB will be asked to review and reapprove this study, where required.

At the end of the study, the investigator (or sponsor where required) will notify the IEC/IRB about the study completion.

16.2.3. Informed Consent and Assent Form

Each subject (or a legally acceptable representative) must give written consent according to local requirements after the nature of the study has been fully explained. The ICF(s) must be signed before performance of any study-related activity. The ICF(s) and assent form that is/are used must be approved by both the sponsor and by the reviewing IEC/IRB and be in a language that the subject can understand. The informed consent should be in accordance with principles that originated in the Declaration of Helsinki, current ICH and GCP guidelines, applicable regulatory requirements, and sponsor policy.

Before enrollment in the study, the investigator or an authorized member of the study-site personnel must explain to potential subjects or their legally acceptable representatives the aims, methods, reasonably anticipated benefits, and potential hazards of the study, and any discomfort participation in the study may entail. Subjects will be informed that their participation is voluntary and that they may withdraw consent to participate at any time. They will be informed that choosing not to participate will not affect the care the subject will receive. Finally, they will be told that the investigator will maintain a subject identification register for the purposes of long-term follow-up if needed and that their records may be accessed by health authorities and authorized sponsor personnel without violating the confidentiality of the subject, to the extent permitted by the applicable law(s) or regulations. By signing the ICF the subject or legally acceptable representative is authorizing such access. It also denotes that the subject agrees to allow his or her study physician to recontact the subject for the purpose of obtaining consent for additional safety evaluations, if needed.

The subject or legally acceptable representative will be given sufficient time to read the ICF and the opportunity to ask questions. After this explanation and before entry into the study, consent should be appropriately recorded by means of either the subject's or his or her legally acceptable representative's personally dated signature. After having obtained the consent, a copy of the ICF must be given to the subject.

If the subject or legally acceptable representative is unable to read or write, an impartial witness should be present for the entire informed consent process (which includes reading and explaining all written information) and should personally date and sign the ICF after the oral consent of the subject or legally acceptable representative is obtained.

Children (minors) or subjects who are unable to comprehend the information provided can be enrolled only after obtaining consent of a legally acceptable representative. Assent must be obtained from children (minors) capable of understanding the nature of the study, typically subjects 7 years of age and older, depending on local regulations and practice. Written assent should be obtained from subjects who are able to write. A separate assent form written in language the subject can understand should be developed for adolescents. After having obtained the assent, a copy of the assent form must be given to the subject, and to the subject's parent or if applicable legally acceptable representative.

16.2.4. Privacy of Personal Data

The collection and processing of personal data from subjects enrolled in this study will be limited to those data that are necessary to fulfill the objectives of the study.

These data must be collected and processed with adequate precautions to ensure confidentiality and compliance with applicable data privacy protection laws and regulations. Appropriate technical and organizational measures to protect the personal data against unauthorized disclosures or access, accidental or unlawful destruction, or accidental loss or alteration must be put in place. Sponsor personnel whose responsibilities require access to personal data agree to keep the identity of subjects confidential.

The informed consent obtained from the subject (or his or her legally acceptable representative) includes explicit consent for the processing of personal data and for the investigator/institution to allow direct access to his or her original medical records (source data/documents) for study-related monitoring, audit, IEC/IRB review, and regulatory inspection. This consent also addresses the transfer of the data to other entities and to other countries.

The subject has the right to request through the investigator access to his or her personal data and the right to request rectification of any data that are not correct or complete. Reasonable steps will be taken to respond to such a request, taking into consideration the nature of the request, the conditions of the study, and the applicable laws and regulations.

Exploratory immunogenicity research is not conducted under standards appropriate for the return of data to subjects. In addition, the sponsor cannot make decisions as to the significance of any findings resulting from exploratory research. Therefore, exploratory research data will not be

returned to subjects or investigators, unless required by law or local regulations. Privacy and confidentiality of data generated in the future on stored samples will be protected by the same standards applicable to all other clinical data.

16.2.5. Long-term Retention of Samples for Additional Future Research

Samples collected in this study, for which consent has been obtained and for which additional material is available after study-specific testing is complete, may be stored for up to 15 years (or according to local regulations) for potential future research. Subjects participating at selected sites where PBMC samples are collected will be asked explicitly to consent for potential future genetic research to be performed on PBMC samples. Samples will only be used to understand Ebola vaccine- and disease-related questions and to develop tests/assays related to the characterization of EBOV-directed immune responses or diagnostic tests. The research may begin at any time during the study or the post-study storage period.

Stored samples will be coded throughout the sample storage and analysis process and will not be labeled with personal identifiers. Subjects may withdraw consent for their samples to be stored for potential future research at any time (see Section 10.3, Withdrawal From the Use of Research Samples). In such case, their blood samples will be destroyed after all the tests specified for this study have been concluded.

The sponsor will be responsible for the overall management of the sample inventory, shipping plan, allocation and storage of samples. The sponsor will establish a Material Transfer Agreement between all relevant parties (site, sponsor and the central laboratory charged with shipment and distribution of the samples). These Material Transfer Agreements will take into account national regulations and will be submitted for ethics approval and be in place before a site will be activated. Samples will be stored securely at facilities as directed by the sponsor.

16.2.6. Country Selection

This study will only be conducted in those countries where the intent is to launch or otherwise help ensure access to the developed product if the need for the product persists, unless explicitly addressed as a specific ethical consideration in Section 16.1, Study-Specific Design Considerations.

17. ADMINISTRATIVE REQUIREMENTS

17.1. Protocol Amendments

Neither the investigator nor the sponsor will modify this protocol without a formal amendment by the sponsor. All protocol amendments must be issued by the sponsor, and signed and dated by the investigator. Protocol amendments must not be implemented without prior IEC/IRB approval, or when the relevant competent authority has raised any grounds for non-acceptance, except when necessary to eliminate immediate hazards to the subjects, in which case the amendment must be promptly submitted to the IEC/IRB and relevant competent authority. Documentation of amendment approval by the investigator and IEC/IRB must be provided to the

sponsor. When the change(s) involve(s) only logistic or administrative aspects of the study, the IEC/IRB (where required) only needs to be notified.

During the course of the study, in situations where a departure from the protocol is unavoidable, the investigator or other physician in attendance will contact the appropriate sponsor representative (listed in the Contact Information page(s), which will be provided as a separate document). Except in emergency situations, this contact should be made before implementing any departure from the protocol. In all cases, contact with the sponsor must be made as soon as possible to discuss the situation and agree on an appropriate course of action. The data recorded in the CRF and source documents will reflect any departure from the protocol, and the source documents will describe this departure and the circumstances requiring it.

17.2. Regulatory Documentation

17.2.1. Regulatory Approval/Notification

This protocol and any amendment(s) must be submitted to the appropriate regulatory authorities in each respective country, if applicable. A study may not be initiated until all local regulatory requirements are met.

17.2.2. Required Prestudy Documentation

The following documents must be provided to the sponsor before shipment of study vaccine to the study site:

- Protocol and amendment(s), if any, signed and dated by the principal investigator
- A copy of the dated and signed (or sealed, where appropriate per local regulations), written IEC/IRB approval of the protocol, amendments, ICF, any recruiting materials, and if applicable, subject compensation programs. This approval must clearly identify the specific protocol by title and number and must be signed (or sealed, where appropriate per local regulations) by the chairman or authorized designee.
- Name and address of the IEC/IRB, including a current list of the IEC/IRB members and their function, with a statement that it is organized and operates according to GCP and the applicable laws and regulations. If accompanied by a letter of explanation, or equivalent, from the IEC/IRB, a general statement may be substituted for this list. If an investigator or a member of the study-site personnel is a member of the IEC/IRB, documentation must be obtained to state that this person did not participate in the deliberations or in the vote/opinion of the study.
- Regulatory authority approval or notification, if applicable.
- Signed and dated statement of investigator (eg, Form FDA 1572), if applicable.
- Documentation of investigator qualifications (eg, curriculum vitae).
- Completed investigator financial disclosure form from the principal investigator, where required.

- Signed and dated clinical trial agreement, which includes the financial agreement.
- Any other documentation required by local regulations.

The following documents must be provided to the sponsor before enrollment of the first subject:

- Completed investigator financial disclosure forms from all subinvestigators.
- Documentation of subinvestigator qualifications (eg, curriculum vitae).
- Name and address of any local laboratory conducting tests for the study, and a dated copy of current laboratory normal ranges for these tests, if applicable.
- Local laboratory documentation demonstrating competence and test reliability (eg, accreditation/license), if applicable.

17.3. Subject Identification, Enrollment, and Screening Logs

The investigator agrees to complete a subject identification and enrollment log to permit easy identification of each subject during and after the study. This document will be reviewed by the sponsor study-site contact for completeness.

The subject identification and enrollment log will be treated as confidential and will be filed by the investigator in the study file. To ensure subject confidentiality, no copy will be made. All reports and communications relating to the study will identify subjects by subject identification and date of birth. In cases where the subject is not randomized into the study, the date seen and date of birth will be used.

The investigator must also complete a subject screening log, which reports on all subjects who were seen to determine eligibility for inclusion in the study.

17.4. Source Documentation

At a minimum, source documents consistent in the type and level of detail with that commonly recorded at the study site as a basis for standard medical care, must be available for the following: subject identification, eligibility, and study identification; study discussion and date of signed informed consent; dates of visits; results of safety and immunogenicity parameters as required by the protocol; record of all adverse events and follow-up of adverse events; concomitant medication; study vaccine receipt/dispensing/return records; study vaccine administration information; and date of study completion and reason for early discontinuation of study vaccine or withdrawal from the study, if applicable. The author of an entry in the source documents should be identifiable.

Specific details required as source data for the study and source data collection methods will be reviewed with the investigator before the study and will be described in the monitoring guidelines (or other equivalent document).

The information contained in the subject diary is primary data. Either the physical diary or diary information collected in the subject's medical record will be considered a source document. Information from the diary provided to subjects to record solicited local and systemic adverse

events until 7 days after each vaccination, will be reviewed by the investigator or designee to transcribe into the relevant parts of the CRF as described in the CRF Completion Guidelines.

17.5. Case Report Form Completion

Case report forms are prepared and provided by the sponsor for each subject in electronic format. All CRF entries, corrections, and alterations must be made by the investigator or authorized study-site personnel. The investigator must verify that all data entries in the CRF are accurate and correct.

The study data will be transcribed by study-site personnel from the source documents onto an electronic CRF, if applicable. Study-specific data will be transmitted in a secure manner to the sponsor.

Data must be entered into the CRF in English. The CRF must be completed as soon as possible after a subject visit and the forms should be available for review at the next scheduled monitoring visit.

If necessary, queries will be generated in the eDC tool. If corrections to a CRF are needed after the initial entry into the CRF, this can be done in either of the following ways:

- Investigator and study-site personnel can make corrections in the eDC tool at their own initiative or as a response to an auto query (generated by the eDC tool).
- Sponsor or sponsor delegate can generate a query for resolution by the investigator and study-site personnel.

17.6. Data Quality Assurance/Quality Control

Steps to be taken to ensure the accuracy and reliability of data include the selection of qualified investigators and appropriate study sites, review of protocol procedures with the investigator and study-site personnel before the study, and periodic monitoring visits by the sponsor. Written instructions will be provided for collection, handling, storage, and shipment of samples.

Guidelines for CRF completion will be provided and reviewed with study-site personnel before the start of the study. The sponsor will review CRF for accuracy and completeness during on-site monitoring visits and after transmission to the sponsor; any discrepancies will be resolved with the investigator or designee, as appropriate. After upload of the data into the study database they will be verified for accuracy and consistency with the data sources.

17.7. Record Retention

In compliance with the ICH/GCP guidelines, the investigator/institution will maintain all CRF and all source documents that support the data collected from each subject, as well as all study documents as specified in ICH/GCP Section 8, Essential Documents for the Conduct of a Clinical Trial, and all study documents as specified by the applicable regulatory requirement(s). The investigator/institution will take measures to prevent accidental or premature destruction of these documents.

Essential documents must be retained until at least 2 years after the last approval of a marketing application in an ICH region and until there are no pending or contemplated marketing applications in an ICH region or until at least 2 years have elapsed since the formal discontinuation of clinical development of the investigational product. These documents will be retained for a longer period if required by the applicable regulatory requirements or by an agreement with the sponsor. It is the responsibility of the sponsor to inform the investigator/institution as to when these documents no longer need to be retained.

If the responsible investigator retires, relocates, or for other reasons withdraws from the responsibility of keeping the study records, custody must be transferred to a person who will accept the responsibility. The sponsor must be notified in writing of the name and address of the new custodian. Under no circumstance shall the investigator relocate or dispose of any study documents before having obtained written approval from the sponsor.

If it becomes necessary for the sponsor or the appropriate regulatory authority to review any documentation relating to this study, the investigator/institution must permit access to such reports.

17.8. Monitoring

The sponsor will perform on-site monitoring visits as frequently as necessary. The monitor will record dates of the visits in a study site visit log that will be kept at the study site. The first post-initiation visit will be made as soon as possible after enrollment has begun. At these visits, the monitor will compare the data entered into the CRF with the source documents (eg, hospital/clinic/physician's office medical records). The nature and location of all source documents will be identified to ensure that all sources of original data required to complete the CRF are known to the sponsor and study-site personnel and are accessible for verification by the sponsor study-site contact. If electronic records are maintained at the study site, the method of verification must be discussed with the study-site personnel.

Direct access to source documents (medical records) must be allowed for the purpose of verifying that the recorded data are consistent with the original source data. Findings from this review will be discussed with the study-site personnel. The sponsor expects that, during monitoring visits, the relevant study-site personnel will be available, the source documents will be accessible, and a suitable environment will be provided for review of study-related documents. The monitor will meet with the investigator on a regular basis during the study to provide feedback on the study conduct.

There will be independent monitoring of the pharmacy and preparation of study vaccines by an unblinded monitor (independent study vaccine monitor); regular monitors will be blinded.

In addition to on-site monitoring visits, remote contacts can occur. It is expected that during these remote contacts, study-site personnel will be available to provide an update on the progress of the study at the site.

17.9. Study Completion/Termination

17.9.1. Study Completion/End of Study

The study is considered completed with the last visit for the last subject participating in the study. The final data from the study site will be sent to the sponsor (or designee) after completion of the final subject visit at that study site, in the time frame specified in the Clinical Trial Agreement.

17.9.2. Study Termination

The sponsor reserves the right to close the study site or terminate the study at any time for any reason at the sole discretion of the sponsor. Study sites will be closed upon study completion. A study site is considered closed when all required documents and study supplies have been collected and a study-site closure visit has been performed.

The investigator may initiate study-site closure at any time, provided there is reasonable cause and sufficient notice is given in advance of the intended termination.

Reasons for the early closure of a study site by the sponsor or investigator may include but are not limited to:

- Failure of the investigator to comply with the protocol, the requirements of the IEC/IRB or local health authorities, the sponsor's procedures, or GCP guidelines;
- Inadequate recruitment of subjects by the investigator;
- Discontinuation of further study vaccine development.

17.10. On-site Audits

Representatives of the sponsor's clinical quality assurance department may visit the study site at any time during or after completion of the study to conduct an audit of the study in compliance with regulatory guidelines and company policy. These audits will require access to all study records, including source documents, for inspection. Subject privacy must, however, be respected. The investigator and study-site personnel are responsible for being present and available for consultation during routinely scheduled study-site audit visits conducted by the sponsor or its designees.

The sponsor representatives (ie, auditors) may use an electronic tool to access your Protected Health Information remotely. This electronic tool provides a secure electronic gateway between the doctor’s computer and the computer of the sponsor representatives and ensures that nobody else will be able to access the information that is being transmitted. This secure electronic gateway will only be opened for a limited timeframe after which it will be closed and all contents will be deleted.

Similar auditing procedures may also be conducted by agents of any regulatory body, either as part of a national GCP compliance program or to review the results of this study in support of a

regulatory submission. The investigator should immediately notify the sponsor if he or she has been contacted by a regulatory agency concerning an upcoming inspection.

17.11. Use of Information and Publication

All information, including but not limited to information regarding Ad26.ZEBOV and MVA-BN-Filo or the sponsor's operations (eg, patent application, formulas, manufacturing processes, basic scientific data, prior clinical data, formulation information) supplied by the sponsor to the investigator and not previously published, and any data, including exploratory research data, generated as a result of this study, are considered confidential and remain the sole property of the sponsor and may be apportioned between the consortium members, if contemplated and as detailed in the Consortium Agreement. The investigator agrees to maintain this information in confidence and use this information only to accomplish this study, and will not use it for other purposes without the sponsor's prior written consent.

The investigator understands that the information developed in the study will be used by the sponsor in connection with the continued development of Ad26.ZEBOV and MVA-BN-Filo, and thus may be disclosed as required to other clinical investigators or regulatory agencies. To permit the information derived from the clinical studies to be used, the investigator is obligated to provide the sponsor with all data obtained in the study.

The results of the study will be reported in a Clinical Study Report generated by the sponsor and will contain data from all study sites that participated in the study as per protocol. Recruitment performance or specific expertise related to the nature and the key assessment parameters of the study will be used to determine a coordinating investigator. Results of exploratory analyses performed after the Clinical Study Report has been issued will be reported in a separate report and will not require a revision of the Clinical Study Report. Study subject identifiers will not be used in publication of results. Any work created in connection with performance of the study and contained in the data that can benefit from copyright protection (except any publication by the investigator as provided for below) shall be the property of the sponsor as author and owner of copyright in such work. Further details regarding ownership and access rights by consortium members to the data and results of the study are detailed in the Consortium Agreement.

Consistent with Good Publication Practices and International Committee of Medical Journal Editors guidelines, the sponsor and consortium members shall have the right to publish such primary (multicenter) data and information as detailed in the Consortium Agreement without approval from the individual investigators. The individual investigators have the right to publish study site-specific data after the primary data are published. Further details regarding publications by consortium members and individual investigators are detailed in the Clinical Trial Agreement and Consortium Agreement. The relevant publication sections of the Consortium Agreement will be shared with the investigator once executed by all the consortium partners. For multicenter study designs and substudy approaches, secondary results generally should not be published before the primary endpoints of a study have been published. Similarly, consortium members and investigators will recognize the integrity of a multicenter study by not submitting for publication data derived from the individual study site until the combined results

from the completed study have been submitted for publication, within 12 months of the availability of the final data (tables, listings, graphs), or the sponsor confirms there will be no multicenter study publication. Authorship of publications resulting from this study will be based on the guidelines on authorship, such as those described in the Consortium Agreement and the Uniform Requirements for Manuscripts Submitted to Biomedical Journals, which state that the named authors must have made a significant contribution to the design of the study or analysis and interpretation of the data, provided critical review of the paper, and given final approval of the final version.

Registration of Clinical Studies and Disclosure of Results

The sponsor will register and disclose the existence of and the results of clinical studies as required by law.

REFERENCES

1. Baden LR, Liu J, Li H, et al. Induction of HIV-1-specific mucosal immune responses following intramuscular recombinant adenovirus serotype 26 HIV-1 vaccination of humans. J Infect Dis. 2015;211(4):518-528.
2. Baden LR, Walsh SR, Seaman MS, et al. First-in-human evaluation of the safety and immunogenicity of a recombinant adenovirus serotype 26 HIV-1 Env vaccine (IPCAVD 001). J Infect Dis. 2013;207:240-247.
3. Baize S, Pannetier D, Oestereich L, et al. Emergence of Zaire Ebola virus disease in Guinea. N Engl J Med. 2014;371(15):1418-1425.
4. Barouch DH, Liu J, Peter L, et al. Characterization of humoral and cellular immune responses elicited by a recombinant adenovirus serotype 26 HIV-1 Env vaccine in healthy adults (IPCAVD 001). J Infect Dis. 2013;207:248-256.
5. Centers for Disease Control and Prevention. Clinical Growth Chart. Available at: htttp://www.cdc.gov/growthcharts/clinical_charts.htm. Accessed 10 March 2015.
6. Clinical Study Report MAL-V-A001. A Phase I/IIa, double-blind, randomized, placebo-controlled, dose-escalation clinical study evaluating safety, tolerability and immunogenicity of two dose levels of recombinant adenoviral serotype Ad35 and serotype Ad26 vectors expressing the malaria *Plasmodium falciparum* circumsporozoite antigen administered as heterologous prime-boost regimen, and assessing protective efficacy of the higher dose in a malaria challenge model in unblinded conditions. Crucell Holland B.V. (Aug 2014).
7. Clinical Study Report POX-MVA-013. A randomized, double-blind, placebo-controlled Phase III trial to evaluate immunogenicity and safety of three consecutive production lots of IMVAMUNE (MVA-BN) smallpox vaccine in healthy, Vaccinia-naïve subjects. Bavarian Nordic (Aug 2015).
8. Elizaga ML, Vasan S, Marovich MA, et al.; MVA Cardiac Safety Working Group. Prospective surveillance for cardiac adverse events in healthy adults receiving modified vaccinia Ankara vaccines: a systematic review. PLoS One. 2013;8(1):e54407.
9. Ethical considerations for clinical trials on medicinal products conducted with the pediatric population: Recommendations of the Ad hoc group for the development of implementing guidelines for Directive 2001/20/EC relating to good clinical practice in the conduct of clinical trials on medicinal products for human use. 2008.
10. Frey SE, Newman FK, Kennedy JS, et al. Clinical and immunologic responses to multiple doses of IMVAMUNE (Modified Vaccinia Ankara) followed by Dryvax challenge. Vaccine. 2007;25(51):8562-8573.
11. Friedrich B, Trefry J, Biggins J, et al. Potential vaccines and post-exposure treatments for filovirus infections. Viruses. 2012;4(9):1619-1650.
12. Geisbert TW, Bausch DG, Feldmann H. Prospects for immunisation against Marburg and Ebola viruses. Rev Med Virol. 2010;20(6):344-357.
13. IMVANEX suspension for injection. UK Summary of Product Characteristics. Available at: http://www.ema.europa.eu/ema/index.jsp?curl=pages/medicines/human/medicines/002596/human_med_001666.jsp&mid=WC0b01ac058001d124. Accessed 24 March 2015.
14. Investigator's Brochure: JNJ-61210474 (Ad26.ZEBOV), Edition 2. Crucell Holland B.V. (Feb 2015). For updated information, refer to Edition 4 (23 June 2016) and Addendum 1 to Edition 4 (30 August 2016).
15. Investigator's Brochure: MVA-BN-Filo (MVA-mBN226B), Edition 5. Bavarian Nordic A/S (Mar 2015). For updated information, refer to Edition 10 (11 July 2016).
16. Investigator's Brochure: Ad26.ENVA.01 HIV-1 Vaccine, Edition 3. Crucell Holland B.V. (June 2013).
17. Investigator's Brochure: JNJ-55471468-AAA, JNJ-55471494-AAA, JNJ-55471520-AAA (Ad26.Mos.HIV) HIV 1 vaccine, Edition 2. Crucell Holland B.V. (Nov 2014).
18. Investigator's Brochure: JNJ-55471468-AAA, JNJ-55471494-AAA, JNJ-55471520-AAA (Ad26.Mos.HIV) HIV 1 vaccine, Addendum to Edition 2. Crucell Holland B.V. (Dec 2014).

19. Jones SM, Feldmann H, Ströher U, et al. Live attenuated recombinant vaccine protects nonhuman primates against Ebola and Marburg viruses. Nat Med. 2005;11(7):786-790.

20. Karita E, Mutua G, Bekker LG, et al. Safety in a Phase 1 randomized, double-blind, placebo-controlled trial evaluating two adenovirus hiv vaccines in three different geographic regions (IAVI-B003/IPCAVD-004/HVTN091 trial). Poster presented at AIDS Vaccine 2013.

21. MVA-BN (Modified Vaccinia Ankara - Bavarian Nordic). Available at: http://id.bavarian-nordic.com/pipeline/technology-platform/mva-bn.aspx. Accessed 24 March 2015.

22. Neff J, Modlin J, Birkhead GS, et al. Monitoring the safety of a smallpox vaccination program in the United States: report of the joint Smallpox Vaccine Safety Working Group of the advisory committee on immunization practices and the Armed Forces Epidemiological Board. Clin Infect Dis. 2008;46 Suppl.3:S258-S270.

23. Sarwar UN, Costner P, Enama ME, et al. Safety and immunogenicity of DNA vaccines encoding ebolavirus and marburgvirus wild type glycoproteins in a phase I clinical trial. J Infect Dis. 2015;211(4):549-557.

24. Stittelaar KJ, Kuiken T, de Swart RL, et al. Safety of Modified Vaccinia Virus Ankara (MVA) in immune-suppressed macaques. Vaccine. 2001;19(27):3700-3709.

25. Verheust C, Goossens M, Pauwels K, Breyer D. Biosafety aspects of Modified Vaccinia Virus Ankara (MVA)-based vectors used for gene therapy or vaccination. Vaccine. 2012;30(16):2623-2632.

26. Vollmar J, Arndtz N, Eckl KM, et al. Safety and immunogenicity of IMVAMUNE, a promising candidate as a third generation smallpox vaccine. Vaccine. 2006;24(12):2065-2070.

27. WHO Child Growth Standards (birth to 24 months). Available at: http://www.who.int/childgrowth/en. Accessed 10 March 2015.

28. WHO Fact Sheet N°103 Ebola Virus Disease 2014. Available at: http://www.who.int/mediacentre/factsheets/fs103/en/. Accessed 26 February 2015.

ATTACHMENTS

Attachment 1: Test of Understanding (TOU)

Note: A culturally appropriate translation will be made available to participating subjects.

Please read each question and answer whether the statement is **True** or **False**.

True	False	
True ☐	False ☐	1. The vaccines you will receive in this study will definitely protect against Ebola.
True ☐	False ☐	2. Depending on which group you are randomized to, you will need to come to the clinic for 7 to up to 12 visits, excluding the screening period, over the next months.
True ☐	False ☐	3. The vaccines in this study can give you Ebola.
True ☐	False ☐	4. One purpose of this study is to determine if these vaccines are safe to administer to humans.
True ☐	False ☐	5. Participants in this study will need to avoid engaging in activities that may expose them to Ebola virus.
True ☐	False ☐	6. You may take other experimental (test) products while you are taking part in this study.
True ☐	False ☐	7. You may withdraw from the study at any time if you choose.
True ☐	False ☐	8. Women participating in this study are permitted to become pregnant during the study.
True ☐	False ☐	9. A participant in this study may experience side effects after vaccination.
True ☐	False ☐	10. Some participants in this study may develop a positive Ebola test result, despite the fact that they do not have Ebola disease.

Attachment 2: Toxicity Tables for Use in Trials Enrolling Healthy Adults

The abbreviations used in the following tables are:

ALT: alanine aminotransferase; aPTT: activated partial thromboplastin time; AST: aspartate aminotransferase; AV block: atrioventricular block; bpm: beats per minute; CK: creatine kinase; FEV_1: forced expiratory volume in 1 second; g: gram; HI: high; HPF: high power field; INR: international normalized ratio; IV: intravenous; LO: low; mEq: milliequivalent; mm Hg: millimeter of mercury; N: not graded; PT: prothrombin time; PTT: partial thromboplastin time; QTc: QT-interval corrected for heart rate; QTcB: Bazett's corrected QT interval; QTcF: Fridericia's corrected QT interval; RBC: red blood cell; Rx: therapy; ULN: upper limit of normal

CLINICAL ADVERSE EVENTS

Grading scale used for clinical adverse events is adapted from the Division of Microbiology and Infectious Diseases (DMID) Toxicity Tables (2014). For adverse events not included in the tables below, refer to the severity criteria guidelines in Section 12.1.3.

**Cardiovascular**	**Grade 1**	**Grade 2**	**Grade 3**
Arrhythmia		Asymptomatic, transient signs, no Rx required	Recurrent/persistent; symptomatic Rx required
Hemorrhage, blood loss	Estimated blood loss ≤100 mL	Estimated blood loss >100 mL, no transfusion required	Transfusion required
QTcF (Fridericia's correction) [a] or QTcB (Bazett's correction)	Asymptomatic, QTc interval 450-479 ms, *OR* Increase in interval <30 ms above baseline	Asymptomatic, QTc interval 480-499 ms, *OR* Increase in interval 30-60 ms above baseline[b]	Asymptomatic, QTc interval ≥500 ms, *OR* Increase in interval ≥60 ms above baseline
PR interval (prolonged)	PR interval 0.21-0.25 s	PR interval >0.25 s	Type II 2nd degree AV block *OR* Ventricular pause >3.0 s
**Respiratory**	**Grade 1**	**Grade 2**	**Grade 3**
Cough	Transient; no treatment	Persistent cough	Interferes with daily activities
Bronchospasm, acute	Transient; no treatment; FEV_1 71%-80% of peak flow	Requires treatment; normalizes with bronchodilator; FEV_1 60%-70% (of peak flow)	No normalization with bronchodilator; FEV_1 <60% of peak flow
Dyspnea	Does not interfere with usual and social activities	Interferes with usual and social activities, no treatment	Prevents daily and usual social activity or requires treatment

[a] Inclusion dependent upon protocol requirements.

[b] The Grade 2 increase in interval is changed from 30-50 ms to 30-60 ms since the original DMID Toxicity Tables (2014) did not cover the increase in interval between 50 and 60 ms.

Gastrointestinal	Grade 1	Grade 2	Grade 3
Nausea/vomiting	Minimal symptoms; caused minimal or no interference with work, school or self-care activities	Notable symptoms; required modification in activity or use of medications; did not result in loss of work or cancellation of social activities	Incapacitating symptoms; required bed rest and/or resulted in loss of work or cancellation of social activities
Diarrhea	2-3 loose or watery stools or <400 g/24 hours	4-5 loose or watery stools or 400-800 g/24 hours	6 or more loose or watery stools or >800 g/24 hours or requires IV hydration
**Reactogenicity**	**Grade 1**	**Grade 2**	**Grade 3**
Local reactions			
Pain/tenderness at injection site	Aware of symptoms but easily tolerated; does not interfere with activity; discomfort only to touch	Notable symptoms; required modification in activity or use of medications; discomfort with movement	Incapacitating symptoms; inability to do work or usual activities; significant discomfort at rest
Erythema/redness[a]	2.5-5 cm	5.1-10 cm	>10 cm
Induration/swelling[b]	2.5-5 cm and does not interfere with activity	5.1-10 cm or interferes with activity	>10 cm or prevents daily activity
Itching at the injection site	Minimal symptoms; caused minimal or no interference with work, school or self-care activities	Notable symptoms; required modification in activity or use of medications; did not result in loss of work or cancellation of social activities	Incapacitating symptoms; required bed rest and/or resulted in loss of work or cancellation of social activities
Systemic reactions			
Allergic reaction	Pruritus without rash	Localized urticaria	Generalized urticaria; angioedema or anaphylaxis
Headache	Minimal symptoms; caused minimal or no interference with work, school or self-care activities	Notable symptoms; required modification in activity or use of medications; did not result in loss of work or cancellation of social activities	Incapacitating symptoms; required bed rest and/or resulted in loss of work or cancellation of social activities
Fatigue/malaise	Minimal symptoms; caused minimal or no interference with work, school or self-care activities	Notable symptoms; required modification in activity or use of medications; did not result in loss of work or cancellation of social activities	Incapacitating symptoms; required bed rest and/or resulted in loss of work or cancellation of social activities
Myalgia	Minimal symptoms; caused minimal or no interference with work, school or self-care activities	Notable symptoms; required modification in activity or use of medications; did not result in loss of work or cancellation of social activities	Incapacitating symptoms; required bed rest and/or resulted in loss of work or cancellation of social activities

[a] In addition to grading the measured local reaction at the greatest single diameter, the measurement should be recorded as a continuous variable.

[b] Induration/swelling should be evaluated and graded using the functional scale as well as the actual measurement.

Arthralgia	Minimal symptoms; caused minimal or no interference with work, school or self-care activities	Notable symptoms; required modification in activity or use of medications; did not result in loss of work or cancellation of social activities	Incapacitating symptoms; required bed rest and/or resulted in loss of work or cancellation of social activities
Chills	Minimal symptoms; caused minimal or no interference with work, school or self-care activities	Notable symptoms; required modification in activity or use of medications; did not result in loss of work or cancellation of social activities	Incapacitating symptoms; required bed rest and/or resulted in loss of work or cancellation of social activities

LABORATORY TOXICITY GRADING

Grading scale used for lab assessments is based on 'FDA's Toxicity Grading Scale for Healthy Adult and Adolescent Volunteers Enrolled in Preventive Vaccine Clinical Trials', but grade 3 and 4 are pooled below, consistent with the 3 scale toxicity grading used throughout the protocol. If a laboratory value falls within the grading as specified below but also within the local laboratory normal limits, the value is considered as normal. For hemoglobin only the change from reference is used for the grading. The FDA table does not include toxicity grading for hematocrit, RBC counts or INR.

Blood, Serum, or Plasma Chemistries[a]	LO/HI/N [b]	Mild (Grade 1)	Moderate (Grade 2)	Severe (Grade 3)
Sodium (mEq/L or mmol/L)	LO	132-134	130-131	≤129
	HI	144-145	146-147	≥148
Potassium (mEq/L or mmol/L)	LO	3.5-3.6	3.3-3.4	≤3.2
	HI	5.1-5.2	5.3-5.4	≥5.5
Glucose (mg/dL)	LO	65-69	55-64	≤54
	HI [c]	100-110	111-125	>125
	HI [d]	110-125	126-200	>200
Blood urea nitrogen	HI	23-26 (mg/dL) or 8.3-9.4 (mmol/L)	27-31 (mg/dL) or 9.5- 11.2 (mmol/L)	>31 (mg/dL) or >11.2 (mmol/L)
Creatinine	N	1.5-1.7 (mg/dL) or 133-151 (µmol/L)	1.8-2.0 (mg/dL) or 152-177 (µmol/L)	>2.0 (mg/dL) or > 177 (µmol/L)
Calcium (mg/dL)	LO	8.0-8.4	7.5-7.9	<7.5
	HI	10.5-11.0	11.1-11.5	>11.5
Magnesium (mg/dL)	LO	1.3-1.5	1.1-1.2	<1.1
Phosphorus (mg/dL)	LO	2.3-2.5	2.0-2.2	<2.0
CK (mg/dL)	N	1.25-1.5 x ULN	1.6-3.0 x ULN	≥3.1 x ULN

[a] Depending upon the laboratory used, reference ranges, eligibility ranges and grading may be split out by sex and/or age.
[b] Low, High, Not Graded.
[c] Fasting.
[d] Non-fasting.

**Blood, Serum, or Plasma Chemistries**[a]	**LO/HI/N** [b]	**Mild (Grade 1)**	**Moderate (Grade 2)**	**Severe (Grade 3)**
Albumin (g/dL)	LO	2.8-3.1	2.5-2.7	<2.5
Total protein (g/dL)	LO	5.5-6.0	5.0-5.4	<5.0
Alkaline phosphatase (U/L)	N	1.1-2 x ULN	2.1-3 x ULN	>3 x ULN
AST (U/L)	HI	1.1-2.5 x ULN	2.6-5 x ULN	>5 x ULN
ALT (U/L)	HI	1.1-2.5 x ULN	2.6-5 x ULN	>5 x ULN
Bilirubin, serum total (mg/dL) – when accompanied by any increase in Liver Function Test		1.1–1.25 x ULN	1.2 –1.5 x ULN	>1.5 x ULN
Bilirubin, serum total (mg/dL) – when Liver Function Test is normal		1.1–1.5 x ULN	1.6–2.0 x ULN	>2.0 x ULN
Amylase (U/L)	N	1.1-1.5 x ULN	1.6-2.0 x ULN	>2.0 x ULN
Lipase (U/L)	N	1.1-1.5 x ULN	1.6-2.0 x ULN	>2.0 x ULN

**Hematology**	**LO/HI/N** [a]	**Mild (Grade 1)**	**Moderate (Grade 2)**	**Severe (Grade 3)**
Hemoglobin (women) change from baseline (g/dL)	LO	Any decrease-1.5	1.6-2.0	>2.0
Hemoglobin (men) change from baseline (g/dL)	LO	Any decrease-1.5	1.6-2.0	>2.0
White blood cell count (cell/mm^3)	HI	10,800-15,000	15,001-20,000	>20,000
	LO	2,500-3,500	1,500-2,499	<1,500
Lymphocytes (cell/mm^3)	LO	750-1,000	500-749	< 500
Neutrophils (cell/mm^3)	LO	1,500-2,000	1,000-1,499	< 1000
Eosinophils (cell/mm^3)	HI	650-1500	1501-5000	> 5000
Platelets (cell/mm^3)	LO	125,000-140,000	100,000-124,000	<100,000
**Coagulation**				
PT (seconds)	HI	1.0-1.10 x ULN	1.11-1.20 x ULN	>1.20 x ULN
International Normalized Ratio (INR)[b]	HI	1.1-1.5 x ULN	1.6-2.0 x ULN	>2.0 x ULN
PTT or aPTT (seconds)	HI	1.0-1.2 x ULN	1.21-1.4 x ULN	>1.4 x ULN
Fibrinogen (mg/dL)	HI	400-500	501-600	>600
	LO	150-200	125-149	<125
**Urine**				
Protein (dipstick)	HI	Trace	1+	2+
Glucose (dipstick)	HI	Trace	1+	2+
Blood (microscopic) - red blood cells per high power field (RBC/HPF)	HI	1-10	11-50	>50 and/or gross blood

[a] Low, High, Not Graded.
[b] For INR, the values in the table are based on the Division of AIDS Table for Grading the Severity of Adult and Pediatric Adverse Events, 2009.

VITAL SIGNS TOXICITY GRADING

Grading scale used for vital signs is according to DMID Toxicity Tables (2014)

Vital Signs	LO/HI/N [a]	Mild (Grade 1) [b]	Moderate (Grade 2)	Severe (Grade 3)
Fever (°C)[c]	HI	38.0-38.4	38.5-38.9	>38.9
Fever (°F)	HI	100.4-101.1	101.2-102.0	>102.0
Tachycardia	HI	101-115 bpm	116-130 bpm	>130 bpm or ventricular dysrhythmias
Bradycardia	LO	50-54 or 45-50 bpm if baseline <60 bpm	45-49 or 40-44 bpm if baseline <60 bpm	<45 or <40 bpm if baseline <60 bpm
Hypertension (systolic) - mm Hg[d]	HI	141-150	151-160	>160
Hypertension (diastolic) - mm Hg	HI	91-95	96-100	>100
Hypotension (systolic) - mm Hg	LO	85-89	80-84	<80
Tachypnea - breaths per minute	HI	23-25	26-30	>30

[a] Low, High, Not Graded.

[b] If initial bound of grade 1 has gap from reference range or eligibility range, calculations based on the New England Journal of Medicine (NEJM) reference ranges.

[c] Axillary temperature. A protocol should select either °C or °F for inclusion.

[d] Assuming subject is awake, resting, and supine; for adverse events, 3 measurements on the same arm with concordant results.

Attachment 3: Toxicity Tables for Use in Trials Enrolling Children Greater Than 3 Months of Age

The abbreviations used in the following tables are:

ALT: alanine aminotransferase; AST: aspartate aminotransferase; CNS: central nervous system; CK: creatine kinase; hpf: high power field; GGT: gamma glutamyltransferase; mEq: milliequivalent; PT: prothrombin time; PTT: partial prothrombin time; ULN: upper limit of normal

CLINICAL ADVERSE EVENTS

Grading scale used for clinical adverse events is adapted from the DMID Pediatric Toxicity Tables for Children Greater Than 3 Months of Age (2007). For adverse events not included in the tables below, refer to the severity criteria guidelines in Section 12.1.3.

**Gastrointestinal**	**Grade 1**	**Grade 2**	**Grade 3**
Nausea/vomiting	Minimal symptoms; caused minimal or no interference with work, school or self-care activities	Notable symptoms; required modification in activity or use of medications; did not result in loss of work or cancellation of social activities	Incapacitating symptoms; required bed rest and/or resulted in loss of work or cancellation of social activities
Diarrhea	Slight change in consistency and/or frequency of stools	Liquid stools	Liquid stools greater than 4x the amount or number normal for the child
Appetite	-	Decreased appetite	Appetite very decreased, no solid food taken
Abdominal Pain	Mild	Moderate; no treatment needed	Moderate; treatment needed
Constipation	Slight change in consistency/frequency of stool	Hard, dry stools with a change in frequency	Abdominal pain
**Reactogenicity**	**Grade 1**	**Grade 2**	**Grade 3**
Local reactions			
Pain/tenderness at injection site	Aware of symptoms but easily tolerated; does not interfere with activity; discomfort only to touch	Notable symptoms; required modification in activity or use of medications; discomfort with movement	Incapacitating symptoms; inability to do work or usual activities; significant discomfort at rest
Erythema/redness	< 10 mm	10-25 mm	26-50 mm
Induration/swelling	< 10 mm	10-25 mm	26-50 mm
Itching at the injection site	Infrequent, brief episode of scratching, easily distracted from scratching	Frequent, longer episodes of scratching, difficult to distract	Near constant scratching, or scratching during sleep; excoriation of skin
Edema	< 10 mm	10-25 mm	26-50 mm
Rash at the injection site	< 10 mm	10-25 mm	26-50 mm
Systemic reactions			
Allergic reaction	Pruritus without rash	Pruritic rash	Mild urticaria

Headache	Minimal symptoms; caused minimal or no interference with work, school or self-care activities	Notable symptoms; required modification in activity or use of medications; did not result in loss of work/school or cancellation of social activities	Incapacitating symptoms; required bed rest and/or resulted in loss of work/school or cancellation of social activities
Fatigue/malaise	Minimal symptoms; caused minimal or no interference with work, school or self-care activities	Notable symptoms; required modification in activity or use of medications; did not result in loss of work/school or cancellation of social activities	Incapacitating symptoms; required bed rest and/or resulted in loss of work/school or cancellation of social activities
Myalgia	Minimal symptoms; caused minimal or no interference with work, school or self-care activities	Notable symptoms; required modification in activity or use of medications; did not result in loss of work/school or cancellation of social activities	Incapacitating symptoms; required bed rest and/or resulted in loss of work/school or cancellation of social activities
Arthralgia	Minimal symptoms; caused minimal or no interference with work, school or self-care activities	Notable symptoms; required modification in activity or use of medications; did not result in loss of work/school or cancellation of social activities	Incapacitating symptoms; required bed rest and/or resulted in loss of work/school or cancellation of social activities
Chills	Minimal symptoms; caused minimal or no interference with work, school or self-care activities	Notable symptoms; required modification in activity or use of medications; did not result in loss of work/school or cancellation of social activities	Incapacitating symptoms; required bed rest and/or resulted in loss of work/school or cancellation of social activities
**Central Nervous System (CNS)**	**Grade 1**	**Grade 2**	**Grade 3**
Generalized CNS Symptoms			Dizziness
Level of activity		Slightly irritable OR slightly subdued	Very irritable OR lethargic
Visual		Blurriness, diplopia, or horizontal nystagmus of <1 hour duration, with spontaneous resolution	More than 1 episode of Grade 2 symptoms per week, or an episode of Grade 2 symptoms lasting more than 1 hour with spontaneous resolution by 4 hours, or vertical nystagmus
Myelopathy		None	None
**Peripheral Nervous System**	**Grade 1**	**Grade 2**	**Grade 3**
Neuropathy/Lower Motor Neuropathy		Mild transient paresthesia only	Persistent or progressive paresthias, burning sensation in feet, or mild dyesthesia; no weakness; mild to moderate deep tendon reflex changes; no sensory loss
Myopathy or Neuromuscular Junction Impairment	Normal or mild (<2 x ULN) CK elevation	Mild proximal weakness and/or atrophy not affecting gross motor function. Mild myalgias with/without mild CK elevation (<2 x ULN)	Proximal muscle weakness and/or atrophy affecting motor function with/without CK elevation; or severe myalgias with CK>2 x ULN

Other	Grade 1	Grade 2	Grade 3
Fever	38.0-38.4 °C or 100.4-101.1 °F	38.5-40 °C or 101.2-104.0 °F	Greater than 40 °C or 104.0 °F
Cutaneous	Localized rash	Diffuse maculopapular rash	Generalized urticaria
Stomatitis	Mild discomfort	Painful, difficulty swallowing, but able to eat and drink	Painful: unable to swallow solids
Clinical symptom not otherwise specified in this table	No therapy; monitor condition	May require minimal intervention and monitoring	Requires medical care and possible hospitalization
Laboratory values not otherwise specified in this table	Abnormal, but requiring no immediate intervention; monitor	Sufficiently abnormal to require evaluation as to causality and perhaps mild therapeutic intervention, but not of sufficient severity to warrant immediate changes in study vaccine	Sufficiently severe to require evaluation and treatment, including at least temporary suspension of study vaccine

LABORATORY TOXICITY GRADING

Serum chemistry	Mild (Grade 1)	Moderate (Grade 2)	Severe (Grade 3)
Bilirubin (when accompanied by any increase in other liver tests)	1.1-<1.25 x ULN	1.25-<1.5 x ULN	1.5-1.75 x ULN
Bilirubin (when other liver function tests are in normal range)	1.1-<1.5 x ULN	1.5-<2.0 x ULN	2.0-3.0 x ULN
AST	1.1-<2.0 x ULN	2.0-<3.0 x ULN	3.0-8.0 x ULN
ALT	1.1-<2.0 x ULN	2.0-<3.0 x ULN	3.0-8.0 x ULN
GGT	1.1-<2.0 x ULN	2.0-<3.0 x ULN	3.0-8.0 x ULN
Pancreatic amylase	1.1-1.4 x ULN	1.5-1.9 x ULN	2.0-3.0 x ULN
Uric acid	7.5-9.9 mg/dL	10-12.4 mg/dL	12.5-15.0 mg/dL
CK	See Neuromuscular Toxicity		
Creatinine 3 months – 2 years of age	0.6-0.8 x ULN	0.9-1.1 x ULN	1.2-1.5 x ULN
Creatinine 2 – 12 years of age	0.7-1.0 x ULN	1.1-1.6 x ULN	1.7-2.0 x ULN
Creatinine greater than 12 years of age	1.0-1.7 x ULN	1.8-2.4 x ULN	2.5-3.5 x ULN
Hypernatremia	-	<145-149 mEq/L	150-155 mEq/L
Hyponatremia	-	130-135 mEq/L	129-124 mEq/L
Hyperkalemia	5.0-5.9 mEq/L	6.0-6.4 mEq/L	6.5-7.0 mEq/L
Hypokalemia	3.0-3.5 mEq/L	2.5-2.9 mEq/L	2.0-2.4 mEq/L
Hypercalcemia	10.5-11.2 mg/dL	0.9-1.1 mg/dL	12.0-12.9 mg/dL
Hypocalcemia	7.8-8.4 mg/dL	7.0-7.7 mg/dL	6.0-6.9 mg/dL

Hypomagnesemia	1.2-1.4 mEq/L	0.9-1.1 mEq/L	0.6-0.8 mEq/L
Hyperglycemia	116-159 mg/dL	160-249 mg/dL	250-400 mg/dL
Hypoglycemia	55-65 mg/dL	40-54 mg/dL	30-39 mg/dL
**Hematology**	**Mild<br>(Grade 1)**	**Moderate<br>(Grade 2)**	**Severe<br>(Grade 3)**
Hemoglobin for children greater than 3 months and less than 2 years of age	9.0-9.9 mg/dL	7.0-8.9 mg/dL	<7.0 mg/dL
Hemoglobin for children greater than 2 years of age	10.0-10.9 mg/dL	7.0-9.9 mg/dL	<7.0 mg/dL
Absolute neutrophil count	750-1200/mm^3	400-749/mm^3	250-399/mm^3
Platelets	-	50,000-75,000/mm^3	25,000-49,999/mm^3
PT	1.1-1.2 x ULN	1.3-1.5 x ULN	1.6-3.0 x ULN
PTT	1.1-1.6 x ULN	1.7-2.3 x ULN	2.4-3.0 x ULN
**Urinalysis**	**Mild<br>(Grade 1)**	**Moderate<br>(Grade 2)**	**Severe<br>(Grade 3)**
Proteinuria	1+ or < 150 mg/day	2+ or 150-499 mg/day	3+ or 500-1000 mg/day
Hematuria	Microscopic <25 cells/hpf	Microscopic >25 cells/hpf	-

Attachment 4: Hemoglobin Cut-off Values

Where no institutional normal reference ranges is available for hemoglobin, the following cut-off values are proposed. It is imperative to note that there are no standard accepted normative values for hemoglobin in most African countries and therefore, the following recommendations are based on the review of several published sources and in consultation with the sites involved with the study. In the table below, 'simplified' means that the number of cut-off categories has been reduced to decrease complexity and facilitate understanding about eligibility. Similarly, 'adjusted for safety' means that the references may quote lower average or -2 SD values for hemoglobin but these values are considered to be too low for these subjects.

Group	Value (g/dL)		Reference	Outcome
Adult and HIV+	Male 12.1	Female 9.5	LLN value for local sites in Kenya and Uganda, current values being used in Phase 1	Values kept for consistency
Adolescent 16-18 yrs	Male 12.1	Female 9.5	Robins reference 10.4 g/dL for girls and 12.4 g/dL for boys	Values simplified to correspond to adult cut-offs
Adolescent 11-15 yrs	Male 11.0	Female 9.5	Robins reference 11.0 g/dL for girls and boys	Value adjusted down for boys, no change for girls
Children 6-10 yrs	11.0		Robins reference 10.7 g/dL for girls and boys	Value simplified
Children 2-5 yrs	11.0		Robins reference 10.4 g/dL for girls and boys, LLN value for local lab in Kenya 11.5 g/dL for girls and 14.5 g/dL for boys, Schellenberg reference 8.2-9.3 g/dL average anemic defined as <11.0 g/dL	Value simplified and adjusted for safety to 11.0 g/dL
Children 1-2 yrs	11.0		Schellenberg reference 8.0 g/dL average anemic defined as <11.0 g/dL, DMID toxicity table 11.0 g/dL	Value simplified and adjusted for safety to 11.0 g/dL

Robins E and Blum S. Hematologic Reference Values for African American Children and Adolescents. American J Hematology. 2007:82, 611-614.

Schellenberg D, et al. The silent burden of anaemia in Tanzanian children: a community-based study. Bulletin of the World Health Organization 2003;81:581-590.

DMID US FDA Guidance document DIVISION OF MICROBIOLOGY AND INFECTIOUS DISEASES (DMID) PEDIATRIC TOXICITY TABLES NOVEMBER 2007

INVESTIGATOR AGREEMENT

I have read this protocol and agree that it contains all necessary details for carrying out this study. I will conduct the study as outlined herein and will complete the study within the time designated.

I will provide copies of the protocol and all pertinent information to all individuals responsible to me who assist in the conduct of this study. I will discuss this material with them to ensure that they are fully informed regarding the study vaccine, the conduct of the study, and the obligations of confidentiality.

Coordinating Investigator (where required):

Name (typed or printed): ______________________

Institution and Address: ______________________

____

____

____

Signature: ______________________ Date: ______________________

(Day Month Year)

Principal (Site) Investigator:

Name (typed or printed): ______________________

Institution and Address: ______________________

____

____

____

Telephone Number: ______________________

Signature: ______________________ Date: ______________________

(Day Month Year)

Sponsor's Responsible Medical Officer:

Name (typed or printed): [redacted]

Institution: [redacted]

Signature: [electronic signature appended at the end of the protocol] Date: ______________________

(Day Month Year)

Note: If the address or telephone number of the investigator changes during the course of the study, written notification will be provided by the investigator to the sponsor, and a protocol amendment will not be required.

LAST PAGE

SIGNATURES

Signed by	Date	Justification
[redacted]	[redacted]	Document Approval
