# Supplementary material for: Safety and immunogenicity of 2-dose heterologous Ad26.ZEBOV, MVA-BN-Filo Ebola vaccination in children and adolescents in Africa: A randomised, placebo-controlled, multicentre Phase II clinical trial
Source: PLoS Med. 2022 Jan 11;19(1):e1003865. doi: 10.1371/journal.pmed.1003865 (PMC8752100; doi:10.1371/journal.pmed.1003865)
Supplement: S1 CONSORT Checklist — (DOCX) [file pmed.1003865.s002.docx]

**CONSORT 2010 checklist of information to include when reporting a randomised trial***

| **Section/Topic** | **Item No** | **Checklist item** | **Reported on page No** |
| --- | --- | --- | --- |
| **Title and abstract** | | | |
|  | 1a | Identification as a randomised trial in the title | Title |
|  | 1b | Structured summary of trial design, methods, results, and conclusions (for specific guidance see CONSORT for abstracts) | Abstract |
| **Introduction** | | | |
| Background and objectives | 2a | Scientific background and explanation of rationale | Introduction (para 1-3) |
|  | 2b | Specific objectives or hypotheses | Introduction (para 3), Methods (Study overview para 2) |
| **Methods** | | | |
| Trial design | 3a | Description of trial design (such as parallel, factorial) including allocation ratio | Methods (Study overview para 1; Study participants, randomisation and blinding para 2) |
|  | 3b | Important changes to methods after trial commencement (such as eligibility criteria), with reasons | NA |
| Participants | 4a | Eligibility criteria for participants | Methods (Study participants, randomisation and blinding para 1), S1 Study Protocol |
|  | 4b | Settings and locations where the data were collected | Methods (Study overview para 1; Study participants, randomisation and blinding para 1) |
| Interventions | 5 | The interventions for each group with sufficient details to allow replication, including how and when they were actually administered | Methods (Vaccines para 1), S1 Study Protocol |
| Outcomes | 6a | Completely defined pre-specified primary and secondary outcome measures, including how and when they were assessed | Methods (Study overview para 2; Safety and tolerability assessments para 1; Immunogenicity assessments para 1-4), S1 Study Protocol |
|  | 6b | Any changes to trial outcomes after the trial commenced, with reasons | N/A |
| Sample size | 7a | How sample size was determined | Methods (Sample size determinationpara 1) |
|  | 7b | When applicable, explanation of any interim analyses and stopping guidelines | N/A |
| Randomisation: |  |  |  |
| Sequence generation | 8a | Method used to generate the random allocation sequence | Methods (Study participants, randomisation and blinding para 2) |
|  | 8b | Type of randomisation; details of any restriction (such as blocking and block size) | Methods (Study participants, randomisation and blinding para 2) |
| Allocation concealment mechanism | 9 | Mechanism used to implement the random allocation sequence (such as sequentially numbered containers), describing any steps taken to conceal the sequence until interventions were assigned | NA |
| Implementation | 10 | Who generated the random allocation sequence, who enrolled participants, and who assigned participants to interventions | Methods (Study participants, randomisation and blinding para 2) |
| Blinding | 11a | If done, who was blinded after assignment to interventions (for example, participants, care providers, those assessing outcomes) and how | Methods (Study participants, randomisation and blinding para 2) |
|  | 11b | If relevant, description of the similarity of interventions | Methods (Study participants, randomisation and blinding para 2) |
| Statistical methods | 12a | Statistical methods used to compare groups for primary and secondary outcomes | Methods (Statistical analyses para 1-3) |
|  | 12b | Methods for additional analyses, such as subgroup analyses and adjusted analyses | NA |
| **Results** | | | |
| Participant flow (a diagram is strongly recommended) | 13a | For each group, the numbers of participants who were randomly assigned, received intended treatment, and were analysed for the primary outcome | Results (Participant enrolment and baseline demographics para 1); Fig1 |
|  | 13b | For each group, losses and exclusions after randomisation, together with reasons | Results (Participant enrolment and baseline demographics para 1); Fig1 |
| Recruitment | 14a | Dates defining the periods of recruitment and follow-up | Results (Participant enrolment and baseline demographics para 1); |
|  | 14b | Why the trial ended or was stopped | NA |
| Baseline data | 15 | A table showing baseline demographic and clinical characteristics for each group | Table 1 |
| Numbers analysed | 16 | For each group, number of participants (denominator) included in each analysis and whether the analysis was by original assigned groups | Results (Safety para 1-7) |
| Outcomes and estimation | 17a | For each primary and secondary outcome, results for each group, and the estimated effect size and its precision (such as 95% confidence interval) | Results (Safety-Immunogenicity) |
|  | 17b | For binary outcomes, presentation of both absolute and relative effect sizes is recommended | NA |
| Ancillary analyses | 18 | Results of any other analyses performed, including subgroup analyses and adjusted analyses, distinguishing pre-specified from exploratory | Results (Safety para 2-7; Immunogenicity para 2-9) ; Supplementary file |
| Harms | 19 | All important harms or unintended effects in each group (for specific guidance see CONSORT for harms) | Results (Safety) |
| **Discussion** | | | |
| Limitations | 20 | Trial limitations, addressing sources of potential bias, imprecision, and, if relevant, multiplicity of analyses | Discussion (para 4) |
| Generalisability | 21 | Generalisability (external validity, applicability) of the trial findings | Discussion (para 1-3, 5) |
| Interpretation | 22 | Interpretation consistent with results, balancing benefits and harms, and considering other relevant evidence | Discussion (para 1-3, 5) |
| **Other information** | | |  |
| Registration | 23 | Registration number and name of trial registry | Abstract, Methods (Study overview para 1) |
| Protocol | 24 | Where the full trial protocol can be accessed, if available | Methods (Study overview para 1), S1 Study Protocol |
| Funding | 25 | Sources of funding and other support (such as supply of drugs), role of funders | Supplemental Information |
