# Supplementary material for: Safety and immunogenicity of 2-dose heterologous Ad26.ZEBOV, MVA-BN-Filo Ebola vaccination in children and adolescents in Africa: A randomised, placebo-controlled, multicentre Phase II clinical trial
Source: PLoS Med. 2022 Jan 11;19(1):e1003865. doi: 10.1371/journal.pmed.1003865 (PMC8752100; doi:10.1371/journal.pmed.1003865)
Supplement: S1 Supporting information — Text A. Supplementary methodology. Text B. Supplementary results. Text C. EBL2002 study group (in addition to authors). Table A. Solicited local adverse events, solicited systemic adverse events, and unsolicited adverse events; full analysis set. Table B. Comparison (children [4–11 years] versus adolescents [12–17 years]) of solicited and unsolicited adverse events after each vaccination dose based on Fisher’s exact test; full analysis set. Table C. Comparison (vaccinees versus placebo recipients) of solicited and unsolicited adverse events after each vaccination dose based on Fisher’s exact test; full analysis set. Table D. Comparison (Ad26.ZEBOV versus MVA-BN-Filo) of solicited and unsolicited adverse events in the full analysis set based on Fisher’s exact test. (Study VAC52150EBL2002; full analysis set). Table E. Duration of solicited local adverse events and solicited systemic adverse events; full analysis set. Table F. Serious adverse events, full analysis set. Table G. EBOV GP-specific binding antibody responses (ELISA units/mL): geometric mean concentrations and responder rates; per protocol set. Table H. Comparison of EBOV-GP-specific binding antibodies in adolescents [12–17 years] and children [4–11 years] in the Ebola vaccine groups; per protocol set. Table I. Comparison of EBOV-GP-specific binding antibodies in children [4–11 years] versus adolescents [12–17 years] in the Ebola vaccine groups; per protocol set. Table J. EBOV GP-specific binding antibody responses (ELISA units/mL): geometric mean concentrations and responder rates by country; per protocol analysis set. Table K. EBOV GP-specific neutralising antibody responses (psVNA; IC50 titre); per protocol analysis set. Table L. EBOV GP-specific neutralising antibody responses (psVNA; IC50 titre) by country; per protocol analysis set. Table M. Ad26 neutralising antibodies (Ad26 VNA; IC90 titre); per protocol analysis set. Table N. EBOV GP-specific CD4+ T cell cytokine responses (ICS, % of subset); per p [file pmed.1003865.s003.docx]

**Supplementary Material**

Text A. Supplementary Methodology

Text B. Supplementary Results

Text C. EBL2002 study group (in addition to authors)

Table A. Solicited Local Adverse Events, Solicited Systemic Adverse Events, and Unsolicited Adverse Events; Full Analysis Set

Table B. Comparison (children [4-11 years] versus adolescents [12-17 years]) of solicited and unsolicited adverse events after each vaccination dose based on Fisher’s exact test; Full Analysis Set

Table C. Comparison (vaccinees versus placebo recipients) of solicited and unsolicited adverse events after each vaccination dose based on Fisher’s exact test. Full Analysis Set

Table D. Comparison (Ad26.ZEBOV versus MVA-BN-Filo) of solicited and unsolicited adverse events in the Full Analysis Set based on Fisher’s exact test. (Study VAC52150EBL2002; Full Analysis Set).

Table E. Duration of Solicited Local Adverse Events and Solicited Systemic Adverse Events; Full Analysis Set

Table F. Serious Adverse Events, Full Analysis Set

Table G. EBOV GP-specific binding antibody responses (ELISA units/mL): geometric means concentrations and responder rates; per protocol set

Table H. Comparison of EBOV-GP-specific binding antibodies in adolescents [12-17 years] and children [4-11 years] in the Ebola vaccine groups; per protocol set

Table I. Comparison of EBOV-GP-specific binding antibodies in children [4-11 years] versus adolescents [12-17 years] in the Ebola vaccine groups; per protocol set

Table J. EBOV GP-specific Binding Antibody Responses (ELISA Units/mL): Geometric Means Concentrations and Responder Rates by Country; per Protocol Analysis Set

Table K. EBOV GP-specific Neutralising Antibody Responses (psVNA; IC50 Titre); per Protocol Analysis Set

Table L. EBOV GP-specific Neutralising Antibody Responses (psVNA; IC50 Titre) by Country; per Protocol Analysis Set

Table M. Ad26 Neutralising Antibodies (Ad26 VNA; IC90 Titre); per Protocol Analysis Set

Table N. EBOV GP-specific CD4+ T Cell Cytokine Responses (ICS, % of Subset); per Protocol Analysis Set

Table O. EBOV GP-specific CD8+ T Cell Cytokine Responses (ICS, % of Subset); per Protocol Analysis Set

Table P. EBOV GP-specific IFN-γ Producing T Cell Responses (IFN-γ ELISpot, SFU/106 PBMC); per Protocol Analysis Set

Fig A. EBOV GP-specific Neutralising Antibody Responses – Regimen Plot (psVNA; IC_50_ Titre); Per Protocol Analysis Set

Fig B. Spearman Correlation Between EBOV GP-specific Binding and Neutralising Antibody Responses 21 Days Post-MVA-BN-Filo; Per Protocol Analysis Set

1. 21 days post-dose 2
2. 364 days post-dose 1

Fig C: Correlations between Ad26-specific neutralising antibody titres at baseline and EBOV GP-specific binding and neutralising antibodies 21 days post-dose 2

A) Anti EBOV GP IgG ELISA at 21 days post-dose 2 by Ad26 neutralisation assay at baseline

B) EBOV GP neutralisation assay at 21 days post-dose 2 by Ad26 neutralisation assay at baseline

Fig D. CD4+ and CD8+ T Cell Responses in Adolescents (ICS)

Fig E. CD4+ and CD8+ T Cell Responses in Children (ICS)

Fig F: EBOV GP-specific IFN-γ Producing T Cell Responses (ELISpot)

1. Adolescents (12–17 years)
2. Children (4–11 years)

**Text A. Supplementary Methodology**

**Sample Positivity and Responder Definitions for Humoral Immunogenicity Assays**

*Filovirus Animal Non-clinical group (FANG) ELISA*

A FANG ELISA result (ELISA Units [EU]/mL) was considered positive if the value was above the assay lower limit of quantification (LLOQ; 36.11 EU/mL). Values below the LLOQ were imputed with LLOQ/2.

FANG ELISA responders were defined as negative at baseline and positive post-vaccination with a concentration 2.5-fold higher than the LLOQ, or positive at baseline with a greater than 2.5-fold increase post-vaccination.

*Pseudovirion Neutralisation Assay (psVNA)*

A psVNA result (IC_50_ titre) was considered positive if the specific IC_50_ titre was more than three times amphotropic murine leukemia virus (aMLV) and above the assay-specific LLOQ (120 IC_50_ titre). Values that were less than three times aMLV or below the LLOQ were imputed with LLOQ/2.

psVNA responders were defined as negative at baseline and positive post-vaccination with a titre two-fold higher than the LLOQ, or positive at baseline with a greater than two-fold increase post-vaccination.

**Cellular Immunogenicity Assays**

Two hundred eighty one samples were collected from a total of 71 individuals (between 16-20 individuals per cohort). The intention was to analyse all PBMC samples but, in practice, 176 samples (176/281 = 62.6%) were analysed for CD4+ response by ICS, 214 samples (214/281 = 76.2%) were analysed for CD8 response by ICS, and 171 samples (171/281 = 60.9) were analysed for IFNg response by ELISPOT. The reduction in number of analysed samples is due to a variety of reasons. Twenty five samples (25/281 = 8.9%) were lost in transit, eight samples (8/281 = 2.8%) had low recovery, 22 samples (22/281 = 7.8%) had low viability, and one (1/281 = 0.4%) sample had both low viability and low recovery, leaving 225 samples for analysis. Forty nine samples (11/281 = 14.4%) failed assay success criteria for CD4 ICS (the most common reason for failure was high CD4+ count). Eleven samples (11/281 = 3.9%) failed assay success criteria for CD8 ICS (the most common reason for failure was high CD8 count). Fifty four samples (54/281 = 19.2%) failed assay success criteria for ELISPOT (the most common reason for failure was high variation between replicates).

*Intracellular Cytokine Staining (ICS)*

CD4+ and CD8+ T cell responses were determined in a planned subset (29% adolescents and 22% children) of participants which included 18 (14 active and 4 placebo) of 131 and 20 (16 active and 4 placebo) of 131 adolescent recipients in 28- and 56-day interval groups, respectively; 15 (13 active and 2 placebo) of 132 and 14 (11 active and 3 placebo) of 132 children recipients in 28- and 56-day interval groups, respectively.

Sample interpretation was determined for each Ebola virus (EBOV) glycoprotein (GP) peptide pool (GP1 and GP2) separately. For each antigen, the total cytokine count of the considered antigen (IFN-γ+ or IL-2+ or TNF-α+) was compared with the total cytokine count in all negative control samples with Fisher’s exact test. The test was repeated for both peptide pools (GP1 and GP2) and for each antigen. If the observed p-value for at least one peptide pool (GP1 or GP2) was below 10^-5^/2 (division by 2 due to Bonferroni correction as this is done for each pool), the sample interpretation was considered positive for that antigen. Values below the assay-specific threshold were imputed with threshold /2 (0.02%). For the calculation of fold increases, values below the assay-specific threshold were imputed with the threshold.

A participant was a responder at a considered timepoint if sample interpretation was negative at baseline but positive post-baseline and the post-baseline value was greater than 2× the Sponsor-defined assay-specific threshold (0.04%), or sample interpretation was positive at both baseline and post-baseline and there was a greater than two-fold increase from baseline in background adjusted total cytokine response.

*Enzyme-linked Immunospot (ELISpot)*

IFN-γ producing T cell responses were determined at baseline, 21 days post-MVA-BN-Filo (day 50 or 78 for the 28- and 56-day interval groups, respectively), 6 months post-MVA-BN-Filo, and 1 year post-Ad26.ZEBOV in subsets of the participants. The planned subsets included 16 adolescents (14 vaccinees, 2 placebo) of 131 in the 28-day interval group and 19 adolescents (15 vaccinees, 4 placebo) of 131 in the 56-day interval group; and 12 children (11 vaccinees, 1 placebo) of 132 in the 28-day interval group and 12 children (10 vaccinees, 2 placebo) of 132 in the 56-day interval group.

Sample interpretation was determined for each EBOV GP peptide pool (GP1 and GP2) separately. If a sample was positive for at least one of the peptide pools, the sample was considered positive. Overall, a result was considered positive if the peptide pool-stimulated readout was greater than three-fold the unstimulated readout and the unstimulated-subtracted value was greater than the threshold (50 SFU/10^6^ PBMC). Values below the threshold were imputed with half of the threshold. For the calculation of fold increases, values below the threshold were imputed with the threshold.

A participant was a responder at a considered timepoint if the sample interpretation was negative at baseline and positive post-baseline and the post-baseline value was greater than 2x the threshold, or if the sample interpretation was positive both at baseline and post-baseline and there was a greater than two-fold increase from baseline.

**Text B. Supplementary Results**

*CD4+ and CD8+ T cell responses (ICS)*

CD4+ and CD8+ T cell responses were not observed in placebo recipients. At 21 days post-MVA-BN-Filo, CD4+ T cell responses were observed in 40% of adolescents in both the 28-day (2/5; median, 0.08% [IQR, <threshold–0.17%]) and 56-day interval groups (2/5; median: 0.08% [IQR, <threshold–0.11%]) (**S4A Fig**; **Table N in S1 Data**). CD8+ T cell responses were not quantifiable in the adolescents (**S4B Fig; Table O in S1 Data**). At 21 days post-MVA-BN-Filo, CD4+ T cell responses were observed in 6/10 (60%) and 3/9 (33%) children in the 28- and 56-day interval groups, respectively, with median values of 0.19% (IQR, 0.10%–0.30%) in the 28-day interval group and 0.07% (IQR, <threshold–0.23%) in the 56-day interval groups (**S5A Fig**; **Table N in S1 Data**). In 2/11 (18%) children in the 28-day interval group, CD8+ T cell responses were detected with a median below the LLOQ (IQR,<threshold–0.11%); CD8+ T cell responses were not detected in any children in the 56-day interval group (n=8) (**S5B Fig; Table O in S1 Data**). At 6 months post-MVA-BN-Filo and 1 year post-Ad26.ZEBOV, CD4+ T cell responses were low or not quantifiable in all groups. At 6 months post-MVA-BN-Filo and one-year post-Ad26.ZEBOV, CD8+ T cell responses were low or not quantifiable in the children (0–10% at 6 months, 0–22% at 1 year).

*IFN-γ Producing T Cell Responses (ELISpot)*

Unfortunately, due to PBMC shipment losses, assay failure and/or low sample viability, fewer numbers of participants were analysed by ELISpot than planned.

In the placebo groups, the median IFN-γ producing T cell frequency was below the threshold of 50 SFU/10^6^ PBMC at all timepoints. At 21 days post-MVA-BN-Filo, responses were observed in 1/8 (13%) and 2/7 (29%) adolescents in the 28-day and 56-day interval groups, respectively, with a median IFN-γ producing T cell response of 63 SFU/10^6^ PBMC [interquartile range (IQR), <50–152] in the 28-day interval group and 99 SFU/10^6^ PBMC (IQR, <50-122) in the 56-day interval group (**Table P in S1 Data; S6A Fig**). At the same timepoint (post-MVA-BN-Filo), responses were observed in 3/6 (50%) and 2/8 (25%) children in the 28-day and 56-day interval groups, respectively, with a median IFN-γ producing T cell response of 66 SFU/10^6^ PBMC (IQR, <50–130) in the 28-day interval group and 70 SFU/10^6^ PBMC (IQR, <50–117) in the 56-day interval group (**Table P in S1 Data; S6B Fig**). These responses had declined to below 50 SFU/10^6^ PBMC in both groups at 6 months and 1 year post-Ad26.ZEBOV.

**Text C. EBL2002 study group (in addition to authors)**

**EBOVAC 2 Executive Steering Committee**

Pr Yves LEVY, Inserm

Pr Peter PIOT, London School of Hygiene and Tropical Medicine

Pr Johan VAN HOOF, Janssen B.V.

**EBOVAC 2 Clinical Steering Committee**

Dr Macaya DOUOGUIH, Janssen B.V.

Dr Cynthia ROBINSON, Janssen B.V.

Pr Rodolphe THIEBAUT, Inserm

Dr Laura RICHERT, Inserm

Dr Deborah WATSON-JONES, London School of Hygiene and Tropical Medicine

Pr Brian GREENWOOD, London School of Hygiene and Tropical Medicine

Pr Andrew J POLLARD, University of Oxford

Dr Matthew D SNAPE, University of Oxford

Pr Nicolas MEDA, Centre Muraz

Dr Houreratou BARRY, Centre Muraz

Florance CHUNG, INSERM Transfert

Sinéad QUIGLEY, INSERM Transfert

**Janssen Vaccines and Prevention B.V.**

Kim OFFERGELD, Clinical Program Leader, Global Clinical Operations

Benoit CALLENDRET, Compound Development Team Leader

Stephanie DINCQ, Clinical Project Management Lead

Camille FERRAULT, Clinical Project Management Lead

Helga PISSENS, Global Trial Leader

Marleen Van Looveren, Regulatory Medical Writer

Sylvia Van Ballaert, Regulatory Medical Writer

Tinne DE CNODDER, Global Data Management Leader

Tracy HENDRICK, Clinical Programming Lead

**CLINICAL WORKING GROUP Members**

Sanne De Ridder, Clinical Trial Manager

Len Roza, ID&V Risk Management

Njinju FOGAP, Lead Programmer

Rachana Gundluru, Senior Specialist Central Monitoring Manager

Olanrewaju OLADIMEJI, Global Data Manager

Vanessa Errijgers, Supply Management Coordinator

Maartje VAN WELIJ, Senior Independent Drug Monitor Manager

Nicolette MULLER, Clinical Research and Functional Manager

**France EBL2002 Coordination Team**

Pr Yves LEVY, ESC Member

Anton OTTAVI, PhD, EBOVAC2 Project Coordinator

Eugénie DESTANDAU, Communication Officer

**Inserm CIC 1401, EUCLID/F-CRIN Clinical Trials Platform**

Christine SCHWIMMER, PhD, EUCLID Executive Director

Christine BETARD, Clinical trial project manager

Laetitia MOINOT, PharmD, Clinical Trial Project Manager

Cédrick WALLET, EUCLID Operations Manager

**INSERM U955, Vaccine Research Institute, Université Paris-Est Créteil**

Aurélie WIEDEMANN, PhD, Immunologist

Christine LACABARATZ, Immunologist

**Centre Médical de Suivi des Donneurs de Sang, Abidjan**

Dr Albert MINGA, Site Investigator

**Service de Santé au Travail SAPH, Toupah/Ousrou**

Pr Désiré BLEHOU, Site Investigator

**PAC-CI, CHU Treichville, Abidjan**

Dr Ida VIHO, MD, Study coordinator

Dr Patrick COFFIE, Expert

**CeDReS, Université Félix Houphouët-Boigny, Abidjan**

Dr André INWOLEY, Expert

**KAVI Institute of Clinical Research, University of Nairobi**

Nancy THAIRU, Finance Manager

Dr Borna NYAOKE, Investigator

Dr Lavinia BWISA, Investigator

Bashir FARAH, Laboratory Manager

Moses MUNDIA, Data Manager

Dorothy ESSENDI, Study Coordinator

**Makerere University Walter Reed Project, Kampala**

Dr Salim WAKABI, PI

Maureen MUKYALA, Study Coordinator

Amir WAMALA, PoR

Allan TINDIKAHWA, Regulatory and Compliance Manager

Dr Betty MWESIGWA, Sub Investigator

Ezra MUSINGYE, Data Manager

**MRC/UVRI and LSHTM Uganda Research Unit, Entebbe**

Prof. Pontiano KALEEBU, Co-PI

Dr Jennifer SERWANGA, Sub-Investigator/Immunologist

Dr Ggayi ABU-BAKER MUSTAPHER, Sub-Investigator/Project coordinator

Dr Jonathan KITONSA, Sub-Investigator/Study Clinician

Mr Paul TAIRE, Pharmacy Technician

Dr Laura Joan NSANGI, Sub-Investigator/Study Clinician

Mr Vincent BASAJJA, Community Liaisons Officer

Mr Tobias VUDRIKO, Safety Laboratory Technologist

Mr Ben GOMBE, Immunology Laboratory Technologist

Mr Hellen KALUNGI, Study Nurse

Mr Francis KASEKENDE, Study Pharmacist

Dr Mary NYANTARO, Sub-Investigator/Study Clinician

**Centre Muraz, Bobo-Dioulasso**

Dr Lionel Wilfried OUEDRAOGO, Laboratory Manager

Dr Armel PODA, Sub-Investigator

Dr Bachirou TINTO, Pharmacist

Dr Dramane KANIA, Laboratory Manager

Dr Naalona Sandrine HIEN, Sub-Investigator

Dr Guekoun LOUGUE, Pharmacist

Dr Innocent VALEA, Coordinator

Pr Halidou TINTO, Coordinator

Dr Ines Evelyne DA, Sub-investigator

**Groupe de Recherche Action en Santé (GRAS)/ Centre National de Recherche et de Formation sur le Paludisme, Ouagadougou**

Dr Alfred B. TIONO, Study Coordinator

Dr Alphonse OUEDRAOGO, Clinical Investigator

Dr Edith BOUGOUMA, Study Pharmacist

Dr Issa NEBIE, Senior Lab Manager

Dr Diarra AMIDOU, Lab Manager

Dr Daouda OUATTARA, Study Physician

**Independent Data Monitoring Committee**

Dr Bruce MCCLAIN

Dr Geert MOLENDBERGHS

Dr Tsiri AGBENYEGA

Dr Eric Wenceslas Joseph BALAYSSAC

| **Table A.** Solicited Local Adverse Events, Solicited Systemic Adverse Events, and Unsolicited Adverse Events; Full Analysis Set | | | | | | | | | | | | | | | | | | | | | | | |
| --- | --- | --- | --- | --- | --- | --- | --- | --- | --- | --- | --- | --- | --- | --- | --- | --- | --- | --- | --- | --- | --- | --- | --- |
|  | | | **Adolescents (12–17 years)** | | | | | | | | | | | | **Children (4–11 years)** | | | | | | | | |
|  | | | 28-day interval group | | | | 56-day interval group | | | | All groups | | | | 28-day interval group | | | 56-day interval group | | | | All groups | |
|  | | | **Vaccines** | | **Placebo** | | **Vaccines** | | **Placebo** | | **Vaccines** | | **Placebo** | | **Vaccines** | **Placebo** | | **Vaccines** | | **Placebo** | | **Vaccines** | **Placebo** |
| **Post-dose 1 (Ad26.ZEBOV/placebo)** | N | | 55 | | 11 | | 55 | | 10 | | 110 | | 21 | | 54 | | 12 | | 54 | | 12 | 108 | 24 |
| **Solicited local AEs, n (%)** | Any | | 26 (47) | | 3 (27) | | 30 (55) | | 5 (50) | | 56 (51) | | 8 (38) | | 26 (48) | | 6 (50) | | 29 (54) | | 5 (42) | 55 (51) | 11 (46) |
|  | Mild | | 20 (36) | | 2 (18) | | 26 (47) | | 4 (40) | | 46 (42) | | 6 (29) | | 21 (39) | | 6 (50) | | 26 (48) | | 5 (42) | 47 (44) | 11 (46) |
|  | Moderate | | 6 (11) | | 1 (9) | | 4 (7) | | 1 (10) | | 10 (9) | | 2 (10) | | 3 (6) | | 0 | | 2 (4) | | 0 | 5 (5) | 0 |
|  | Severe | | 0 | | 0 | | 0 | | 0 | | 0 | | 0 | | 2 (4) | | 0 | | 1 (2) | | 0 | 3 (3) | 0 |
| Erythema^a^ | Any | | 0 | | 0 | | 1 (2) | | 0 | | 1 (1) | | 0 | | 6 (11) | | 0 | | 2 (4) | | 2 (17) | 8 (7) | 2 (8) |
|  | Mild | | 0 | | 0 | | 1 (2) | | 0 | | 1 (1) | | 0 | | 6 (11) | | 0 | | 2 (4) | | 2 (17) | 8 (7) | 2 (8) |
|  | Moderate | | 0 | | 0 | | 1 (2) | | 0 | | 1 (1) | | 0 | | 5 (9) | | 0 | | 2 (4) | | 2 (17) | 7 (6) | 2 (8) |
|  | Severe | | 0 | | 0 | | 0 | | 0 | | 0 | | 0 | | 1 (2) | | 0 | | 0 | | 0 | 1 (1) | 0 |
| Pain | Any | | 24 (44) | | 1 (9) | | 26 (47) | | 2 (20) | | 50 (45) | | 3 (14) | | 21 (39) | | 3 (25) | | 24 (44) | | 4 (33) | 45 (42) | 7 (29) |
|  | Mild | | 19 (35) | | 1 (9) | | 22 (40) | | 1 (10) | | 41 (37) | | 2 (10) | | 18 (33) | | 3 (25) | | 21 (39) | | 4 (33) | 39 (36) | 7 (29) |
|  | Moderate | | 5 (9) | | 0 | | 4 (7) | | 1 (10) | | 9 (8) | | 1 (5) | | 2 (4) | | 0 | | 2 (4) | | 0 | 4 (4) | 0 |
|  | Severe | | 0 | | 0 | | 0 | | 0 | | 0 | | 0 | | 1 (2) | | 0 | | 1 (2) | | 0 | 2 (2) | 0 |
| Pruritis | Any | | 10 (18) | | 3 (27) | | 5 (9) | | 0 | | 15 (14) | | 3 (14) | | 7 (13) | | 3 (25) | | 7 (13) | | 2 (17) | 14 (13) | 5 (21) |
|  | Mild | | 10 (18) | | 3 (27) | | 5 (9) | | 0 | | 15 (14) | | 3 (14) | | 7 (13) | | 3 (25) | | 7 (13) | | 2 (17) | 14 (13) | 5 (21) |
|  | Moderate | | 9 (16) | | 2 (18) | | 5 (9) | | 0 | | 14 (13) | | 2 (10) | | 6 (11) | | 3 (25) | | 6 (11) | | 2 (17) | 12 (11) | 5 (21) |
|  | Severe | | 1 (2) | | 1 (9) | | 0 | | 0 | | 1 (1) | | 1 (5) | | 1 (2) | | 0 | | 1 (2) | | 0 | 2 (2) | 0 |
| Swelling | Any | | 11 (20) | | 2 (18) | | 9 (16) | | 3 (30) | | 20 (18) | | 5 (24) | | 12 (22) | | 1 (8) | | 7 (13) | | 2 (17) | 19 (18) | 3 (13) |
|  | Mild | | 9 (16) | | 2 (18) | | 9 (16) | | 3 (30) | | 18 (16) | | 5 (24) | | 10 (19) | | 1 (8) | | 7 (13) | | 2 (17) | 17 (16) | 3 (13) |
|  | Moderate | | 2 (4) | | 0 | | 0 | | 0 | | 2 (2) | | 0 | | 1 (2) | | 0 | | 0 | | 0 | 1 (1) | 0 |
|  | Severe | | 0 | | 0 | | 0 | | 0 | | 0 | | 0 | | 1 (2) | | 0 | | 0 | | 0 | 1 (1) | 0 |
|  |  | |  | |  | |  | |  | |  | |  | |  | |  | |  | |  |  |  |
| **Solicited systemic AEs, n (%)** | Any | | 29 (53) | | 4 (36) | | 30 (55) | | 5 (50) | | 59 (54) | | 9 (43) | | 24 (44) | | 2 (17) | | 23 (43) | | 2 (17) | 47 (44) | 4 (17) |
|  | Mild | | 19 (35) | | 4 (36) | | 21 (38) | | 3 (30) | | 40 (36) | | 7 (33) | | 14 (26) | | 2 (17) | | 18 (33) | | 2 (17) | 32 (30) | 4 (17) |
|  | Moderate | | 9 (16) | | 0 | | 8 (15) | | 2 (20) | | 17 (15) | | 2 (10) | | 9 (17) | | 0 | | 5 (9) | | 0 | 14 (13) | 0 |
|  | Severe | | 1 (2) | | 0 | | 1 (2) | | 0 | | 2 (2) | | 0 | | 1 (2) | | 0 | | 0 | | 0 | 1 (1) | 0 |
| Arthralgia | Any | | 11 (20) | | 2 (18) | | 9 (16) | | 1 (10) | | 20 (18) | | 3 (14) | | - | | - | | - | | - | - | - |
|  | Mild | | 6 (11) | | 2 (18) | | 7 (13) | | 1 (10) | | 13 (12) | | 3 (14) | | - | | - | | - | | - | - | - |
|  | Moderate | | 5 (9) | | 0 | | 2 (4) | | 0 | | 7 (6) | | 0 | | - | | - | | - | | - | - | - |
|  | Severe | | 0 | | 0 | | 0 | | 0 | | 0 | | 0 | | - | | - | | - | | - | - | - |
| Chills | Any | | 10 (18) | | 1 (9) | | 9 (16) | | 2 (20) | | 19 (17) | | 3 (14) | | - | | - | | - | | - | - | - |
|  | Mild | | 5 (9) | | 1 (9) | | 9 (16) | | 2 (20) | | 14 (13) | | 3 (14) | | - | | - | | - | | - | - | - |
|  | Moderate | | 4 (7) | | 0 | | 0 | | 0 | | 4 (4) | | 0 | | - | | - | | - | | - | - | - |
|  | Severe | | 1 (2) | | 0 | | 0 | | 0 | | 1 (1) | | 0 | | - | | - | | - | | - | - | - |
| Fatigue | Any | | 25 (45) | | 3 (27) | | 16 (29) | | 1 (10) | | 41 (37) | | 4 (19) | | - | | - | | - | | - | - | - |
|  | Mild | | 17 (31) | | 3 (27) | | 14 (25) | | 1 (10) | | 31 (28) | | 4 (19) | | - | | - | | - | | - | - | - |
|  | Moderate | | 8 (15) | | 0 | | 2 (4) | | 0 | | 10 (9) | | 0 | | - | | - | | - | | - | - | - |
|  | Severe | | 0 | | 0 | | 0 | | 0 | | 0 | | 0 | | - | | - | | - | | - | - | - |
| Headache | Any | | 24 (44) | | 2 (18) | | 23 (42) | | 5 (50) | | 47 (43) | | 7 (33) | | - | | - | | - | | - | - | - |
|  | Mild | | 17 (31) | | 2 (18) | | 15 (27) | | 3 (30) | | 32 (29) | | 5 (24) | | - | | - | | - | | - | - | - |
|  | Moderate | | 7 (13) | | 0 | | 8 (15) | | 2 (20) | | 15 (14) | | 2 (10) | | - | | - | | - | | - | - | - |
|  | Severe | | 0 | | 0 | | 0 | | 0 | | 0 | | 0 | | - | | - | | - | | - | - | - |
| Myalgia | Any | | 11 (20) | | 1 (9) | | 10 (18) | | 2 (20) | | 21 (19) | | 3 (14) | | - | | - | | - | | - | - | - |
|  | Mild | | 10 (18) | | 1 (9) | | 8 (15) | | 1 (10) | | 18 (16) | | 2 (10) | | - | | - | | - | | - | - | - |
|  | Moderate | | 1 (2) | | 0 | | 2 (4) | | 1 (10) | | 3 (3) | | 1 (5) | | - | | - | | - | | - | - | - |
|  | Severe | | 0 | | 0 | | 0 | | 0 | | 0 | | 0 | | - | | - | | - | | - | - | - |
| Nausea | Any | | 5 (9) | | 0 | | 2 (4) | | 0 | | 7 (6) | | 0 | | - | | - | | - | | - | - | - |
|  | Mild | | 5 (9) | | 0 | | 1 (2) | | 0 | | 6 (5) | | 0 | | - | | - | | - | | - | - | - |
|  | Moderate | | 0 | | 0 | | 1 (2) | | 0 | | 1 (1) | | 0 | | - | | - | | - | | - | - | - |
|  | Severe | | 0 | | 0 | | 0 | | 0 | | 0 | | 0 | | - | | - | | - | | - | - | - |
| Pyrexia^b^ | | Any | | 2 (4) | | 0 | | 4 (7) | | 1 (10) | | 6 (5) | | 1 (5) | 13 (24) | | 0 | | 11 (20) | | 0 | 24 (22) | 0 |
|  | | Mild | | 0 | | 0 | | 3 (5) | | 0 | | 3 (3) | | 0 | 4 (7) | | 0 | | 7 (13) | | 0 | 11 (10) | 0 |
|  | | Moderate | | 2 (4) | | 0 | | 0 | | 1 (10) | | 2 (2) | | 1 (5) | 9 (17) | | 0 | | 4 (7) | | 0 | 13 (12) | 0 |
|  | | Severe | | 0 | | 0 | | 1 (2) | | 0 | | 1 (1) | | 0 | 0 | | 0 | | 0 | | 0 | 0 | 0 |
| Decreased activity | | Any | | - | | - | | - | | - | | - | | - | 10 (19) | | 0 | | 10 (19) | | 1 (8) | 20 (19) | 1 (4) |
|  | | Mild | | - | | - | | - | | - | | - | | - | 7 (13) | | 0 | | 9 (17) | | 1 (8) | 16 (15) | 1 (4) |
|  | | Moderate | | - | | - | | - | | - | | - | | - | 2 (4) | | 0 | | 1 (2) | | 0 | 3 (3) | 0 |
|  | | Severe | | - | | - | | - | | - | | - | | - | 1 (2) | | 0 | | 0 | | 0 | 1 (1) | 0 |
| Decreased appetite | | Any | | - | | - | | - | | - | | - | | - | 9 (17) | | 1 (8) | | 7 (13) | | 0 | 16 (15) | 1 (4) |
|  | | Mild | | - | | - | | - | | - | | - | | - | 8 (15) | | 1 (8) | | 5 (9) | | 0 | 13 (12) | 1 (4) |
|  | | Moderate | | - | | - | | - | | - | | - | | - | 0 | | 0 | | 2 (4) | | 0 | 2 (2) | 0 |
|  | | Severe | | - | | - | | - | | - | | - | | - | 1 (2) | | 0 | | 0 | | 0 | 1 (1) | 0 |
| Irritability | | Any | | - | | - | | - | | - | | - | | - | 10 (19) | | 0 | | 9 (17) | | 1 (8) | 19 (18) | 1 (4) |
|  | | Mild | | - | | - | | - | | - | | - | | - | 8 (15) | | 0 | | 7 (13) | | 1 (8) | 15 (14) | 1 (4) |
|  | | Moderate | | - | | - | | - | | - | | - | | - | 1 (2) | | 0 | | 2 (4) | | 0 | 3 (3) | 0 |
|  | | Severe | | - | | - | | - | | - | | - | | - | 1 (2) | | 0 | | 0 | | 0 | 1 (1) | 0 |
| Vomiting | | Any | | - | | - | | - | | - | | - | | - | 2 (4) | | 1 (8) | | 7 (13) | | 0 | 9 (8) | 1 (4) |
|  | | Mild | | - | | - | | - | | - | | - | | - | 1 (2) | | 1 (8) | | 6 (11) | | 0 | 7 (6) | 1 (4) |
|  | | Moderate | | - | | - | | - | | - | | - | | - | 1 (2) | | 0 | | 1 (2) | | 0 | 2 (2) | 0 |
|  | | Severe | | - | | - | | - | | - | | - | | - | 0 | | 0 | | 0 | | 0 | 0 | 0 |
|  | |  | |  | |  | |  | |  | |  | |  |  | |  | |  | |  |  |  |
| **Unsolicited AEs, n (%)** | | Any | | 28 (51) | | 4 (36) | | 31 (56) | | 5 (50) | | 59 (54) | | 9 (43) | 23 (43) | | 4 (33) | | 19 (35) | | 4 (33) | 42 (39) | 8 (33) |
|  | | Mild | | 19 (35) | | 2 (18) | | 16 (29) | | 3 (30) | | 35 (32) | | 5 (24) | 16 (30) | | 2 (17) | | 14 (26) | | 3 (25) | 30 (28) | 5 (21) |
|  | | Moderate | | 5 (9) | | 2 (18) | | 8 (15) | | 0 | | 13 (12) | | 2 (10) | 7 (13) | | 2 (17) | | 4 (7) | | 1 (8) | 11 (10) | 3 (13) |
|  | | Severe | | 4 (7) | | 0 | | 7 (13) | | 2 (20) | | 11 (10) | | 2 (10) | 0 | | 0 | | 1 (2) | | 0 | 1 (1) | 0 |
|  | |  | |  | |  | |  | |  | |  | |  |  | |  | |  | |  |  |  |
| **Post-dose 2**  **(MVA-BN-Filo/placebo)** | | N | | 55 | | 10 | | 54 | | 10 | | 109 | | 20 | 54 | | 12 | | 54 | | 11 | 108 | 23 |
| **Solicited local AEs,** **n (%)** | | Any | | 29 (53) | | 3 (30) | | 20 (37) | | 3 (30) | | 49 (45) | | 6 (30) | 22 (41) | | 2 (17) | | 22 (41) | | 2 (18) | 44 (41) | 4 (17) |
|  | | Mild | | 20 (36) | | 2 (20) | | 16 (30) | | 2 (20) | | 36 (33) | | 4 (20) | 19 (35) | | 2 (17) | | 19 (35) | | 1 (9) | 38 (35) | 3 (13) |
|  | | Moderate | | 8 (15) | | 1 (10) | | 4 (7) | | 0 | | 12 (11) | | 1 (5) | 3 (6) | | 0 | | 3 (6) | | 1 (9) | 6 (6) | 1 (4) |
|  | | Severe | | 1 (2) | | 0 | | 0 | | 1 (10) | | 1 (1) | | 1 (5) | 0 | | 0 | | 0 | | 0 | 0 | 0 |
| Erythema | | Any | | 0 | | 0 | | 0 | | 0 | | 0 | | 0 | 4 (7) | | 0 | | 4 (7) | | 0 | 8 (7) | 0 |
|  | | Mild | | 0 | | 0 | | 0 | | 0 | | 0 | | 0 | 4 (7) | | 0 | | 4 (7) | | 0 | 8 (7) | 0 |
|  | | Moderate | | 0 | | 0 | | 0 | | 0 | | 0 | | 0 | 0 | | 0 | | 0 | | 0 | 0 | 0 |
|  | | Severe | | 0 | | 0 | | 0 | | 0 | | 0 | | 0 | 0 | | 0 | | 0 | | 0 | 0 | 0 |
| Pain | | Any | | 29 (53) | | 2 (20) | | 19 (35) | | 1 (10) | | 48 (44) | | 3 (15) | 19 (35) | | 1 (8) | | 17 (31) | | 2 (18) | 36 (33) | 3 (13) |
|  | | Mild | | 21 (38) | | 1 (10) | | 15 (28) | | 0 | | 36 (33) | | 1 (5) | 18 (33) | | 1 (8) | | 14 (26) | | 1 (9) | 32 (30) | 2 (9) |
|  | | Moderate | | 8 (15) | | 1 (10) | | 4 (7) | | 0 | | 12 (11) | | 1 (5) | 1 (2) | | 0 | | 3 (6) | | 1 (9) | 4 (4) | 1 (4) |
|  | | Severe | | 0 | | 0 | | 0 | | 1 (10) | | 0 | | 1 (5) | 0 | | 0 | | 0 | | 0 | 0 | 0 |
| Pruritis | | Any | | 9 (16) | | 1 (10) | | 6 (11) | | 2 (20) | | 15 (14) | | 3 (15) | 6 (11) | | 1 (8) | | 5 (9) | | 1 (9) | 11 (10) | 2 (9) |
|  | | Mild | | 7 (13) | | 1 (10) | | 6 (11) | | 1 (10) | | 13 (12) | | 2 (10) | 5 (9) | | 1 (8) | | 5 (9) | | 0 | 10 (9) | 1 (4) |
|  | | Moderate | | 2 (4) | | 0 | | 0 | | 0 | | 2 (2) | | 0 | 1 (2) | | 0 | | 0 | | 1 (9) | 1 (1) | 1 (4) |
|  | | Severe | | 0 | | 0 | | 0 | | 1 (10) | | 0 | | 1 (5) | 0 | | 0 | | 0 | | 0 | 0 | 0 |
| Swelling | | Any | | 7 (13) | | 2 (20) | | 7 (13) | | 3 (30) | | 14 (13) | | 5 (25) | 10 (19) | | 0 | | 10 (19) | | 1 (9) | 20 (19) | 1 (4) |
|  | | Mild | | 6 (11) | | 1 (10) | | 6 (11) | | 2 (20) | | 12 (11) | | 3 (15) | 9 (17) | | 0 | | 10 (19) | | 1 (9) | 19 (18) | 1 (4) |
|  | | Moderate | | 0 | | 1 (10) | | 1 (2) | | 0 | | 1 (1) | | 1 (5) | 1 (2) | | 0 | | 0 | | 0 | 1 (1) | 0 |
|  | | Severe | | 1 (2) | | 0 | | 0 | | 1 (10) | | 1 (1) | | 1 (5) | 0 | | 0 | | 0 | | 0 | 0 | 0 |
|  | | | |  | |  | |  | |  | |  | |  |  | |  | |  | |  |  |  |
| **Solicited systemic AEs,  n (%)** | | **Any** | | 27 (49) | | 3 (30) | | 25 (46) | | 6 (60) | | 52 (48) | | 9 (45) | 10 (19) | | 3 (25) | | 10 (19) | | 4 (36) | 20 (19) | 7 (30) |
|  | | Mild | | 23 (42) | | 2 (20) | | 20 (37) | | 4 (40) | | 43 (39) | | 6 (30) | 7 (13) | | 2 (17) | | 8 (15) | | 2 (18) | 15 (14) | 4 (17) |
|  | | Moderate | | 4 (7) | | 1 (10) | | 5 (9) | | 2 (20) | | 9 (8) | | 3 (15) | 3 (6) | | 1 (8) | | 2 (4) | | 2 (18) | 5 (5) | 3 (13) |
|  | | Severe | | 0 | | 0 | | 0 | | 0 | | 0 | | 0 | 0 | | 0 | | 0 | | 0 | 0 | 0 |
| Arthralgia | | Any | | 8 (15) | | 1 (10) | | 10 (19) | | 4 (40) | | 18 (17) | | 5 (25) | - | | - | | - | | - | - | - |
|  | | Mild | | 6 (11) | | 1 (10) | | 9 (17) | | 3 (30) | | 15 (14) | | 4 (20) | - | | - | | - | | - | - | - |
|  | | Moderate | | 2 (4) | | 0 | | 1 (2) | | 1 (10) | | 3 (3) | | 1 (5) | - | | - | | - | | - | - | - |
|  | | Severe | | 0 | | 0 | | 0 | | 0 | | 0 | | 0 | - | | - | | - | | - | - | - |
| Chills | | Any | | 10 (18) | | 1 (10) | | 9 (17) | | 1 (10) | | 19 (17) | | 2 (10) | - | | - | | - | | - | - | - |
|  | | Mild | | 9 (16) | | 1 (10) | | 7 (13) | | 0 | | 16 (15) | | 1 (5) | - | | - | | - | | - | - | - |
|  | | Moderate | | 1 (2) | | 0 | | 2 (4) | | 1 (10) | | 3 (3) | | 1 (5) | - | | - | | - | | - | - | - |
|  | | Severe | | 0 | | 0 | | 0 | | 0 | | 0 | | 0 | - | | - | | - | | - | - | - |
| Fatigue | | Any | | 15 (27) | | 2 (20) | | 13 (24) | | 4 (40) | | 28 (26) | | 6 (30) | - | | - | | - | | - | - | - |
|  | | Mild | | 12 (22) | | 1 (10) | | 11 (20) | | 3 (30) | | 23 (21) | | 4 (20) | - | | - | | - | | - | - | - |
|  | | Moderate | | 3 (5) | | 1 (10) | | 2 (4) | | 1 (10) | | 5 (5) | | 2 (10) | - | | - | | - | | - | - | - |
|  | | Severe | | 0 | | 0 | | 0 | | 0 | | 0 | | 0 | - | | - | | - | | - | - | - |
| Headache | | Any | | 21 (38) | | 2 (20) | | 18 (33) | | 6 (60) | | 39 (36) | | 8 (40) | - | | - | | - | | - | - | - |
|  | | Mild | | 18 (33) | | 1 (10) | | 15 (28) | | 4 (40) | | 33 (30) | | 5 (25) | - | | - | | - | | - | - | - |
|  | | Moderate | | 3 (5) | | 1 (10) | | 3 (6) | | 2 (20) | | 6 (6) | | 3 (15) | - | | - | | - | | - | - | - |
|  | | Severe | | 0 | | 0 | | 0 | | 0 | | 0 | | 0 | - | | - | | - | | - | - | - |
| Myalgia | | Any | | 10 (18) | | 1 (10) | | 15 (28) | | 2 (20) | | 25 (23) | | 3 (15) | - | | - | | - | | - | - | - |
|  | | Mild | | 7 (13) | | 1 (10) | | 13 (24) | | 1 (10) | | 20 (18) | | 2 (10) | - | | - | | - | | - | - | - |
|  | | Moderate | | 3 (5) | | 0 | | 2 (4) | | 1 (10) | | 5 (5) | | 1 (5) | - | | - | | - | | - | - | - |
|  | | Severe | | 0 | | 0 | | 0 | | 0 | | 0 | | 0 | - | | - | | - | | - | - | - |
| Nausea | | Any | | 4 (7) | | 1 (10) | | 2 (4) | | 1 (10) | | 6 (6) | | 2 (10) | - | | - | | - | | - | - | - |
|  | | Mild | | 3 (5) | | 0 | | 1 (2) | | 1 (10) | | 4 (4) | | 1 (5) | - | | - | | - | | - | - | - |
|  | | Moderate | | 1 (2) | | 1 (10) | | 1 (2) | | 0 | | 2 (2) | | 1 (5) | - | | - | | - | | - | - | - |
|  | | Severe | | 0 | | 0 | | 0 | | 0 | | 0 | | 0 | - | | - | | - | | - | - | - |
| Pyrexia^b^ | | Any | | 3 (6)^c^ | | 0 | | 2 (4) | | 1 (10) | | 5 (5)^d^ | | 1 (5) | 2 (4) | | 2 (17) | | 2 (4) | | 0 | 4 (4) | 2 (9) |
|  | | Mild | | 3 (5) | | 0 | | 2 (4) | | 0 | | 5 (5) | | 0 | 1 (2) | | 1 (8) | | 0 | | 0 | 1 (1) | 1 (4) |
|  | | Moderate | | 0 | | 0 | | 0 | | 1 (10) | | 0 | | 1 (5) | 1 (2) | | 1 (8) | | 2 (4) | | 0 | 3 (3) | 1 (4) |
|  | | Severe | | 0 | | 0 | | 0 | | 0 | | 0 | | 0 | 0 | | 0 | | 0 | | 0 | 0 | 0 |
| Decreased activity | | Any | | - | | - | | - | | - | | - | | - | 6 (11) | | 1 (8) | | 4 (7) | | 2 (18) | 10 (9) | 3 (13) |
|  | | Mild | | - | | - | | - | | - | | - | | - | 8 (15) | | 1 (8) | | 10 (19) | | 1 (9) | 18 (17) | 2 (9) |
|  | | Moderate | | - | | - | | - | | - | | - | | - | 3 (6) | | 0 | | 1 (2) | | 1 (9) | 4 (4) | 1 (4) |
|  | | Severe | | - | | - | | - | | - | | - | | - | 1 (2) | | 0 | | 0 | | 0 | 1 (1) | 0 |
| Decreased appetite | | Any | | - | | - | | - | | - | | - | | - | 4 (7) | | 1 (8) | | 5 (9) | | 2 (18) | 9 (8) | 3 (13) |
|  | | Mild | | - | | - | | - | | - | | - | | - | 9 (17) | | 1 (8) | | 6 (11) | | 1 (9) | 15 (14) | 2 (9) |
|  | | Moderate | | - | | - | | - | | - | | - | | - | 0 | | 0 | | 2 (4) | | 1 (9) | 2 (2) | 1 (4) |
|  | | Severe | | - | | - | | - | | - | | - | | - | 1 (2) | | 0 | | 0 | | 0 | 1 (1) | 0 |
| Irritability | | Any | | - | | - | | - | | - | | - | | - | 7 (13) | | 2 (17) | | 5 (9) | | 2 (18) | 12 (11) | 4 (17) |
|  | | Mild | | - | | - | | - | | - | | - | | - | 7 (13) | | 1 (8) | | 7 (13) | | 2 (18) | 14 (13) | 3 (13) |
|  | | Moderate | | - | | - | | - | | - | | - | | - | 2 (4) | | 1 (8) | | 2 (4) | | 1 (9) | 4 (4) | 2 (9) |
|  | | Severe | | - | | - | | - | | - | | - | | - | 1 (2) | | 0 | | 0 | | 0 | 1 (1) | 0 |
| Vomiting | | Any | | - | | - | | - | | - | | - | | - | 4 (7) | | 1 (8) | | 3 (6) | | 0 | 7 (6) | 1 (4) |
|  | | Mild | | - | | - | | - | | - | | - | | - | 5 (9) | | 1 (8) | | 8 (15) | | 0 | 13 (12) | 1 (4) |
|  | | Moderate | | - | | - | | - | | - | | - | | - | 1 (2) | | 0 | | 1 (2) | | 0 | 2 (2) | 0 |
|  | | Severe | | - | | - | | - | | - | | - | | - | 0 | | 0 | | 0 | | 0 | 0 | 0 |
|  | |  | |  | |  | |  | |  | |  | |  |  | |  | |  | |  |  |  |
| **Unsolicited AEs, n (%)** | | Any | | 18 (33) | | 4 (40) | | 26 (48) | | 4 (40) | | 44 (40) | | 8 (40) | 20 (37) | | 7 (58) | | 22 (41) | | 8 (73) | 42 (39) | 15 (65) |
|  | | Mild | | 9 (16) | | 2 (20) | | 9 (17) | | 4 (40) | | 18 (17) | | 6 (30) | 11 (20) | | 6 (50) | | 15 (28) | | 5 (45) | 26 (24) | 11 (48) |
|  | | Moderate | | 7 (13) | | 2 (20) | | 13 (24) | | 0 | | 20 (18) | | 2 (10) | 9 (17) | | 1 (8) | | 5 (9) | | 3 (27) | 14 (13) | 4 (17) |
|  | | Severe | | 2 (4) | | 0 | | 4 (7) | | 0 | | 6 (6) | | 0 | 0 | | 0 | | 2 (4) | | 0 | 2 (2) | 0 |
|  | |  | |  | |  | |  | |  | |  | |  |  | |  | |  | |  |  |  |

Abbreviations: AE, adverse event.
For solicited AEs, N= the number of participants with available reactogenicity data after the given dose; for unsolicited events, N= the number of participants who received the given dose.

Only unsolicited AEs reported between the dose 1 vaccination and 28 days post-dose 1, and between dose 2 vaccination and 28 days post-dose 2 are included in this table.

Different diaries were used in adolescents (12–17 years) and children (4–11 years) to collect solicited systemic AEs. Solicited systemic AEs collected in adolescents: arthralgia, chills, fatigue, headache, myalgia, nausea and pyrexia; in children: pyrexia, decreased activity, decreased appetite, irritability, and vomiting.

^a^ Per the Division of Microbiology and Infectious Diseases (DMID) Toxicity Tables used in this study, erythema was graded based on the diameter data only.

^b^ Pyrexia defined as: adolescents, Mild: 38.0–38.4°C, Moderate: 38.5-38.9°C, Severe: >38.9°C; children, Mild: 38.0-38.4°C, Moderate: 38.5-40.0°C, Severe: >40.0°C.

^c^ N=54.
^d^ N=108.

| **Table B.** Comparison (children [4-11 years] versus adolescents [12-17 years]) of solicited and unsolicited adverse events after each vaccination dose based on Fisher’s exact test; Full Analysis Set | | | | | | | | | |  |
| --- | --- | --- | --- | --- | --- | --- | --- | --- | --- | --- |
|  | | **Adolescents (12–17 years)** | | | **Children (4–11 years)** | | | **p-value^*^** | |  |
|  | |  | | |  | | |  | |  |
| **_** | | **Ad26.ZEBOV** | | | **Ad26.ZEBOV** | | |  | |  |
|  | |  | | |  | | |  | |  |
| **Post-dose 1 N** | | 110 | | | 108 | | | - | |  |
|  | |  | | |  | | |  | |  |
| Solicited local AE, n (%) | | 56 (50.9%) | | | 55 (50.9%) | | | 1.0 | |  |
|  | |  | | |  | | |  | |  |
| Solicited systemic AE, n (%) | | 59 (53.6%) | | | 47 (43.5%) | | | 0.14 | |  |
|  | |  | | |  | | |  | |  |
| Any fever, n (%) | | 6 (5.5%) | | | 24 (22.2%) | | | <0.001 | |  |
|  | |  | | |  | | |  | |  |
| Any unsolicited AE, n (%) | | 59 (53.6%) | | | 42 (38.9%) | | | 0.03 | |  |
|  | |  | | |  | | |  | |  |
| **_** | | **MVA-BN-Filo** | | | **MVA-BN-Filo** | | |  | |  |
|  | |  | | |  | | |  | |  |
| **Post-dose 2 N** | | 109 | | | 108 | | | - | |  |
|  | |  | | |  | | |  | |  |
| Solicited local AE, n (%) | | 49 (45.0%) | | | 44 (40.7%) | | | 0.58 | |  |
|  | |  | | |  | | |  | |  |
| Solicited systemic AE, n (%) | | 52 (47.7%) | | | 20 (18.5%) | | | <.001 | |  |
|  | |  | | |  | | |  | |  |
| Any fever, n (%) | | 5 (4.6%) | | | 4 (3.7%) | | | 1.0 | |  |
|  | |  | | |  | | |  | |  |
| Any unsolicited AE, n (%) | | 44 (40.4%) | | | 42 (38.9%) | | | 0.89 | |  |
|  | |  | | |  | | |  | |  |
| Entire study N | | 110 | | | 108 | | |  | |  |
|  | |  | | |  | | |  | |  |
| Any SAE, n (%) | | 1 (0.9%) | | | 1 (0.9%) | | | 1.0 | |  |
| *p-values based on Fisher’s exact test for comparing children (4-11 years) against adolescents (12-17 years).  Vaccines: Ad26.ZEBOV = Ad26.ZEBOV at a dose of 5x10 ^10^vp; MVA-BN-filo = MVA-BN-Filo at a dose of 1x10 ^8^ Inf.U.  AE, adverse event; N, number of participants with data at that timepoint; n (%): number (percentage) of participants with one or more events, where the denominator is the number of participants with available reactogenicity data after the given dose. | | | | | | | | | |  |
| **Table C.** Comparison (vaccinees versus placebo recipients) of solicited and unsolicited adverse events after each vaccination dose based on Fisher’s exact test; Full Analysis Set | | | | | | | | | | |
|  | Adolescents (12–17 years) | | | | | Children (4–11 years) | | | | |
|  |  | |  |  | |  |  | |  | |
|  |  | |  |  | |  |  | |  | |
| **_** | **Ad26.ZEBOV** | | **Placebo** | **p-value^*^** | | **Ad26.ZEBOV** | **Placebo** | | **p-value^*^** | |
|  |  | |  |  | |  |  | |  | |
| **Post-dose 1 N** | 110 | | 21 | - | | 108 | 24 | | - | |
|  |  | |  |  | |  |  | |  | |
| Solicited local AE, n (%) | 56 (50.9%) | | 8 (38.1%) | 0.34 | | 55 (50.9%) | 11 (45.8%) | | 0.82 | |
|  |  | |  |  | |  |  | |  | |
| Solicited systemic AE, n (%) | 59 (53.6%) | | 9 (42.9%) | 0.48 | | 47 (43.5%) | 4 (16.7%) | | 0.02 | |
|  |  | |  |  | |  |  | |  | |
| Any fever, n (%) | 6 (5.5%) | | 1 (4.8%) | 1.0 | | 24 (22.2%) | 0 | | 0.01 | |
|  |  | |  |  | |  |  | |  | |
| Any unsolicited AE, n (%) | 59 (53.6%) | | 9 (42.9%) | 0.48 | | 42 (38.9%) | 8 (33.3%) | | 0.65 | |
|  |  | |  |  | |  |  | |  | |
| **_** | **MVA-BN-Filo** | | **Placebo** | **-** | | **MVA-BN-Filo** | **Placebo** | | **p-value^*^** | |
|  |  | |  |  | |  |  | |  | |
| **Post-dose 2 N** | 109 | | 20 | - | | 108 | 23 | | - | |
|  |  | |  |  | |  |  | |  | |
| Solicited local AE, n (%) | 49 (45.0%) | | 6 (30%) | 0.23 | | 44 (40.7%) | 4 (17.4%) | | 0.05 | |
|  |  | |  |  | |  |  | |  | |
| Solicited systemic AE, n (%) | 52 (47.7%) | | 9 (45.0%) | 1.0 | | 20 (18.5%) | 7 (30.4%) | | 0.25 | |
|  |  | |  |  | |  |  | |  | |
| Any fever, n (%) | 5 (4.6%) | | 1 (5.0%) | 1.0 | | 4 (3.7%) | 2 (8.7%) | | 0.28 | |
|  |  | |  |  | |  |  | |  | |
| Any unsolicited AE, n (%) | 44 (40.4%) | | 8 (40.0%) | 1.0 | | 42 (38.9%) | 15 (65.2%) | | 0.04 | |
|  |  | |  |  | |  |  | |  | |
| **Entire study N** | 110 | | 21 | - | | 108 | 24 | | - | |
|  |  | |  |  | |  |  | |  | |
| Any SAE, n (%) | 1 (0.9%) | | 0 | 1.0 | | 1 (0.9%) | 1 (4.2%) | | 0.33 | |
| *p-values based on Fisher’s exact test for comparing vaccinees against placebo recipients.  Vaccines: Ad26 = Ad26.ZEBOV at a dose of 5x10 ^10^vp; MVA = MVA-BN-Filo at a dose of 1x10 ^8^ Inf.U.  AE, adverse event; N, number of participants with data at that timepoint; n (%): number (percentage) of participants with one or more events, where the denominator is the number of participants with available reactogenicity data after the given dose. | | | | | | | | | | |

| Table D. Comparison (Ad26.ZEBOV versus MVA-BN-Filo) of solicited and unsolicited adverse events in the Full Analysis Set based on Fisher’s exact test. (Study VAC52150EBL2002; Full Analysis Set). | | | | | | |
| --- | --- | --- | --- | --- | --- | --- |
|  | **Adolescents (12–17 years)** | | | **Children (4–11 years)** | | |
|  |  |  |  |  |  |  |
| **_** | **Ad26.ZEBOV** | **MVA-BN-Filo** | **p-value^*^** | **Ad26.ZEBOV** | **MVA-BN-Filo** | **p-value^*^** |
|  |  |  |  |  |  |  |
| **Post-dose N** | 110 | 109 | - | 108 | 108 | - |
|  |  |  |  |  |  |  |
| Solicited local AE, n (%) | 56 (50.9%) | 49 (45.0%) | 0.42 | 55 (50.9%) | 44 (40.7%) | 0.17 |
|  |  |  |  |  |  |  |
| Solicited systemic AE, n (%) | 59 (53.6%) | 52 (47.7%) | 0.42 | 47 (43.5%) | 20 (18.5%) | <0.001 |
|  |  |  |  |  |  |  |
| Any fever, n (%) | 6 (5.5%) | 5 (4.6%) | 1.0 | 24 (22.2%) | 4 (3.7%) | <0.001 |
|  |  |  |  |  |  |  |
| Any unsolicited AE, n (%) | 59 (53.6%) | 44 (40.4%) | 0.06 | 42 (38.9%) | 42 (38.9%) | 1.0 |
| *p-values based on Fisher’s exact test for comparing adverse events following Ad26.ZEBOV (first dose) and MVA-BN-Filo (second dose). Vaccines: Ad26.ZEBOV = Ad26.ZEBOV at a dose of 5x10 ^10^vp; MVA-BN-Filo = MVA-BN-Filo at a dose of 1x10 ^8^ Inf.U. AE, adverse event; N, number of participants with data at that timepoint; n (%): number (percentage) of participants with one or more events, where the denominator is the number of participants with available reactogenicity data after the given dose. | | | | | | |

| **Table E.** Duration of Solicited Local Adverse Events and Solicited Systemic Adverse Events; Full Analysis Set | | | | | | | |
| --- | --- | --- | --- | --- | --- | --- | --- |
|  | | **Adolescents (12–17 years)** | | | **Children (4–11 years)** | | |
|  | | **Ad26.ZEBOV** | **MVA-BN-Filo** | **Placebo** | **Ad26.ZEBOV** | **MVA-BN-Filo** | **Placebo** |
| Number of days,  Median (range) | #Doses | 110 | 109 | 41 | 108 | 108 | 47 |
| **Solicited local AEs** |  |  |  |  |  |  |  |
| Pain |  | 2.0 (1–8) | 3.0 (1–9) | 1.5 (1–6) | 1.0 (1–8) | 2.0 (1–14) | 1.0 (1–5) |
| Pruritis |  | 2.0 (1–6) | 3.0 (1–7) | 2.0 (1–6) | 1.0 (1–6) | 2.0 (1–7) | 1.0 (1–4) |
| Swelling |  | 1.5 (1–8) | 2.5 (1–6) | 1.0 (1–6) | 2.0 (1–7) | 1.5 (1–7) | 3.0 (1–6) |
|  |  |  |  |  |  |  |  |
| **Solicited systemic AEs** |  |  |  |  |  |  |  |
| Arthralgia |  | 2.5 (1–8) | 1.5 (1–11) | 1.5 (1–8) | - | - | - |
| Chills |  | 4.0 (1–8) | 3.0 (1–8) | 1.0 (1–8) | - | - | - |
| Fatigue |  | 2.0 (1–8) | 2.5 (1–9) | 1.0 (1–8) | - | - | - |
| Headache |  | 3.0 (1–10) | 3.0 (1–11) | 5.0 (1–8) | - | - | - |
| Myalgia |  | 2.0 (1–7) | 2.0 (1–8) | 2.0 (1–8) | - | - | - |
| Pyrexia |  | 1.0 (1–5) | 1.0 (1–5) | 4.5 (2–7) | 1.0 (1–5) | 3.5 (1–6) | 1.0 (1–1) |
| Decreases activity |  | - | - | - | 1.0 (1–7) | 2.0 (1–12) | 3.0 (1–7) |
| Decreased appetite |  | - | - | - | 1.5 (1–6) | 1.0 (1–7) | 2.5 (1–6) |
| Irritability |  | - | - | - | 2.0 (1–8) | 3.0 (1–8) | 1.0 (1–4) |
|  |  |  |  |  |  |  |  |

Abbreviations: AE, adverse event.

This table only includes solicited adverse events that were reported in at least 10% of doses in any group.

| **Table F.** Serious Adverse Events, Full Analysis Set | | | | | | | | |
| --- | --- | --- | --- | --- | --- | --- | --- | --- |
|  | **Adolescents (12–17 years)** | | | | **Children (4–11 years)** | | | |
|  | 28-day interval group | | 56-day interval group | | 28-day interval group | | 56-day interval group | |
| MedDRA System Organ Class Dictionary-derived Term | **Vaccines** | **Placebo** | **Vaccines** | **Placebo** | **Vaccines** | **Placebo** | **Vaccines** | **Placebo** |
| **Entire study** | 55 | 11 | 55 | 10 | 54 | 12 | 54 | 12 |
| Any event, n (%) | 1 (2) | 0 | 0 | 0 | 1 (2) | 0 | 0 | 1 (8) |
| Infections and infestations | 1 (2) | 0 | 0 | 0 | 1 (2) | 0 | 0 | 0 |
| Malaria | 1 (2)^a^ | 0 | 0 | 0 | 1 (2) | 0 | 0 | 0 |
| Typhoid fever | 1 (2)^a^ | 0 | 0 | 0 | 0 | 0 | 0 | 0 |
| Injury, poisoning and procedural complications | 0 | 0 | 0 | 0 | 0 | 0 | 0 | 1 (8) |
| Burns second degree | 0 | 0 | 0 | 0 | 0 | 0 | 0 | 1 (8) |
|  |  |  |  |  |  |  |  |  |
| **Post-dose 1** | 55 | 11 | 55 | 10 | 54 | 12 | 54 | 12 |
| Any event, n (%) | 0 | 0 | 0 | 0 | 0 | 0 | 0 | 0 |
|  |  |  |  |  |  |  |  |  |
| **Post-dose 1 follow-up** | 4 | 1 | 55 | 10 | 4 | 1 | 54 | 12 |
| Any event, n (%) | 0 | 0 | 0 | 0 | 0 | 0 | 0 | 1 (8) |
| Injury, poisoning and procedural complications | 0 | 0 | 0 | 0 | 0 | 0 | 0 | 1 (8) |
| Burns second degree | 0 | 0 | 0 | 0 | 0 | 0 | 0 | 1 (8) |
|  |  |  |  |  |  |  |  |  |
| **Post-dose 2** | 55 | 10 | 54 | 10 | 54 | 12 | 54 | 11 |
| Any event, n (%) | 0 | 0 | 0 | 0 | 0 | 0 | 0 | 0 |
|  |  |  |  |  |  |  |  |  |
| **Post-dose 2 follow-up** | 55 | 10 | 54 | 10 | 54 | 12 | 54 | 11 |
| Any event, n (%) | 1 (2) | 0 | 0 | 0 | 1 (2) | 0 | 0 | 0 |
| Infections and infestations | 1 (2) | 0 | 0 | 0 | 1 (2) | 0 | 0 | 0 |
| Malaria | 1 (2) | 0 | 0 | 0 | 1 (2) | 0 | 0 | 0 |
| Typhoid fever | 1 (2) | 0 | 0 | 0 | 0 | 0 | 0 | 0 |
|  |  |  |  |  |  |  |  |  |

Adverse events are coded using MedDRA version 21.1.
n (%): number (percentage) of participants with one or more events.

^a^Fatal case.

Ad26: Ad26.ZEBOV at a dose of 5x10^10^ vp; MVA: MVA-BN-Filo at a dose of 1x10^8^ Inf.U.

| **Table G.** EBOV GP-specific binding antibody responses (ELISA units/mL): geometric means concentrations and responder rates; Per Protocol Analysis Set | | | | | | | | | | |
| --- | --- | --- | --- | --- | --- | --- | --- | --- | --- | --- |
|  | | | **Adolescents (12–17 years)** | | | | **Children (4–11 years)** | | | |
|  | | | 28-day interval group | | 56-day interval group | | 28-day interval group | | 56-day interval group | |
| **Timing** | | | **Vaccines** | **Placebo** | **Vaccines** | **Placebo** | **Vaccines** | **Placebo** | **Vaccines** | **Placebo** |
| **Day 1** | | N | 54 | 10 | 53 | 10 | 53 | 12 | 52 | 11 |
| **Day 29** | | N | 54 | 10 | - |  | 53 | 12 | - | - |
| GMC  (95% CI) | | | 619  (490–782) | <LLOQ  (<LLOQ–63) | - | - | 713  (589–861) | <LLOQ  (<LLOQ–81) | - | - |
| Responders, n/N* (%) | | | 50/54 (93) | 1/10 (10) |  | - | 51/53 (96) | 1/12 (8) | - | - |
| **Day 50** | | N | 53 | 10 | - | - | 53 | 12 | - | - |
| GMC  (95% CI) | | | 6993  (5256–9303) | <LLOQ  (<LLOQ–74) | - | - | 8007  (6321–10 142) | <LLOQ  (<LLOQ–82) | - | - |
| Responders, n/N* (%) | | | 53/53 (100) | 1/10 (10) | - | - | 53/53 (100) | 1/12 (8) | - | - |
| **Day 57** | N | | - | - | 53 | 10 | - | - | 54 | 11 |
| GMC  (95% CI) | | | - | - | 562  (460–686) | 40  (<LLOQ–107) | - | - | 658  (556–780) | <LLOQ |
| Responders, n/N* (%) | | | - | - | 50/53 (94) | 1/10 (10) | - | - | 51/52 (98) | 0/11 (0) |
| **Day 78** | N | | - | - | 53 | 10 | - | - | 53 | 11 |
| GMC  (95% CI) | | | - | - | 13532  (10 732–17 061) | 37  (<LLOQ–89) | - | - | 17388  (12 973–23 306) | <LLOQ |
| Responders, n/N* (%) | | | - | - | 53/53 (100) | 1/10 (10) | - | - | 51/51 (100) | 0/11 (0) |
| **Day 209** | N | | 41 | 7 | - | - | 52 | 12 | - | - |
| GMC  (95% CI) | | | 565  (463–689) | 38  (<LLOQ–79) | - | - | 841  (721–980) | < LLOQ  (<LLOQ–69) | - | - |
| Responders, n/N* (%) | | | 38/41 (93) | 0/7 (0) | - | - | 51/52 (98) | 1/12 (8) | - | - |
| **Day 237** | N | | - | - | 41 | 8 | - | - | 53 | 11 |
| GMC  (95% CI) | | | - | - | 577  (454–734) | 48  (<LLOQ–152) | - | - | 715  (602–851) | <LLOQ |
| Responders, n/N* (%) | | | - | - | 38/41 (93) | 1/8 (13) | - | - | 51/52 (98) | 0/11 (0) |
| **Day 365** | N | | 50 | 9 | 52 | 10 | 53 | 12 | 54 | 11 |
| GMC  (95% CI) | | | 593  (477–738) | <LLOQ  (<LLOQ–70) | 541  (433–678) | <LLOQ  (<LLOQ–90) | 981  (814–1183) | <LLOQ  (<LLOQ–80) | 637  (529–767) | <LLOQ |
| Responders, n/N* (%) | | | 46/50 (92) | 0/9 (0) | 47/52 (90) | 1/10 (10) | 51/53 (96) | 1/12 (8) | 51/52 (98) | 1/11 (9) |
| Abbreviations: CI, confidence interval; EBOV, Ebola virus; ELISA, enzyme-linked immunosorbent assay; GP, glycoprotein; GMC, geometric means concentrations; LLOQ, lower limit of quantitation. N, number of participants with available data at that time point; N*, number of participants with available data at baseline and at that time point.  Vaccines: Ad26.ZEBOV at dose of 5x10^10^ vp; MVA-BN-Filo at a dose of 1x10^8^ Inf.U. | | | | | | | | | | |

| **Table H.** Comparison of EBOV-GP-specific binding antibodies in adolescents [12-17 years] and children [4-11 years] in the Ebola vaccine groups; Per Protocol Analysis Set | | | | | |
| --- | --- | --- | --- | --- | --- |
|  | Comparison | Timepoint | Difference (95% CI) | GMC Ratio (95% CI) | P-value |
| **Adolescents (12-17 years)** | Group 1  vs.  Group 2* | 21 days post-dose 2 | -0.29 (-0.44; -0.13) | 0.5 (0.4; 0.7) | <0.001 |
|  |  | 1-year post-dose 1 | 0.04 (-0.09; 0.17) | 1.1 (0.8; 1.5) | 0.56 |
| **Children (4-11 years)** | Group 1  vs.  Group 2* | 21 days post-dose 2 | -0.34 (-0.50; -0.18) | 0.5 (0.3; 0.7) | <0.001 |
|  |  | 1-year post-dose 1 | 0.19 (0.07; 0.30) | 1.5 (1.2; 2.0) | <0.001 |
| CI, confidence interval.  The computations are based on the log _10_-transformed data and assumed unequal variances in the comparison groups, The GMC ratio and its 95% CIs are obtained by back-transforming the estimated difference (on log _10_-transformed data) and its 95% CI.  Group 1: 28-day interval.  Group 2: 56-day interval.  * Group 2 is the reference comparison group | | | | | |

| **Table I.** Comparison of EBOV-GP-specific binding antibodies in children [4-11 years] versus adolescents [12-17 years] in the Ebola vaccine groups;Pper Protocol Analysis Set | | | | | |
| --- | --- | --- | --- | --- | --- |
|  | **Comparison** | **Timepoint** | **Difference (95% CI)** | **GMC Ratio (95% CI)** | **P-value** |
| **Group 1: 28-day interval** | Children (4-11 years)  vs.  Adolescents (12-17 years)* | 21 days post-dose 2 | 0.06 (-0.10; 0.22) | 1.1 (0.8; 1.7) | 0.47 |
|  |  | 1-year post-dose 1 | 0.22 (0.10; 0.34) | 1.7 (1.2; 2.2) | <0.001 |
| **Group 2: 56-day interval** | Children (4-11 years)  vs.  Adolescents (12-17 years)* | 21 days post-dose 2 | 0.11 (-0.05; 0.27) | 1.3 (0.9; 1.9) | 0.18 |
|  |  | 1-year post-dose 1 | 0.07 (-0.05; 0.20) | 1.2 (0.9; 1.6) | 0.27 |
| The computations are based on the log _10_-transformed data and assumed unequal variances in the comparison groups. The GMC ratio and its 95% CIs are obtained by back-transforming the estimated difference (on log _10_-transformed data) and its 95% CI.  CI, confidence interval.  * Adolescents (12-17 years) is the reference comparison cohort | | | | | |

| **Table J.** EBOV GP-specific Binding Antibody Responses (ELISA Units/mL): Geometric Means Concentrations and Responder Rates by Country; Per Protocol Analysis Set | | | | | | | | |
| --- | --- | --- | --- | --- | --- | --- | --- | --- |
|  | **Adolescents (12–17 years)** | | | | **Children (4–11 years)** | | | |
|  | 28-day interval group | | 56-day interval group | | 28-day interval group | | 56-day interval group | |
|  | **Vaccines** | **Placebo** | **Vaccines** | **Placebo** | **Vaccines** | **Placebo** | **Vaccines** | **Placebo** |
| **Country: Burkina Faso** |  |  |  |  |  |  |  |  |
| **Day 1** |  |  |  |  |  |  |  |  |
| N | 26 | 5 | 29 | 4 | 24 | 5 | 22 | 4 |
| GMC  (95% CI) | 54 (<LLOQ–99) | <LLOQ (<LLOQ–36) | <LLOQ (<LLOQ–41) | 83  (<LLOQ–975) | <LLOQ (<LLOQ–44) | <LLOQ (<LLOQ–<LLOQ) | <LLOQ (<LLOQ–54) | <LLOQ |
| **Day 29** |  |  |  |  |  |  |  |  |
| N | 26 | 5 |  |  | 24 | 5 |  |  |
| GMC  (95% CI) | 755  (532–1073) | <LLOQ (<LLOQ–89) | - | - | 785  (613–1005) | <LLOQ (<LLOQ–<LLOQ) | - | - |
| Responder, n (%) | 23/26 (88) | 1/5 (20) |  |  | 22/24 (92) | 0/5 (0) |  |  |
|  |  |  |  |  |  |  |  |  |
| **Day 50** |  |  |  |  |  |  |  |  |
| N | 26 | 5 |  |  | 24 | 5 |  |  |
| GMC  (95% CI) | 7801  (5113–11 902) | <LLOQ (<LLOQ–80) | - | - | 8795  (5919–13 069) | <LLOQ (<LLOQ–<LLOQ) | - | - |
| Responder, n/N* (%) | 26/26 (100) | 1/5 (20) |  |  | 24/24 (100) | 0/5 (0) |  |  |
|  |  |  |  |  |  |  |  |  |
| **Day 57** |  |  |  |  |  |  |  |  |
| N |  |  | 29 | 4 |  |  | 23 | 4 |
| GMC  (95% CI) | - | - | 517  (439–609) | 75  (<LLOQ–1437) | - | - | 752  (627–902) | <LLOQ |
| Responder, n/N* (%) |  |  | 27/29 (93) | 0/4 (0) |  |  | 21/22 (95) | 0/4 (0) |
|  |  |  |  |  |  |  |  |  |
| **Day 78** |  |  |  |  |  |  |  |  |
| N |  |  | 29 | 4 |  |  | 23 | 4 |
| GMC  (95% CI) | - | - | 13487  (9883–18 405) | 68  (<LLOQ–985) | - | - | 23 949  (16 739–34 266) | <LLOQ |
| Responder, n/N* (%) |  |  | 29/29 (100) | 0/4 (0) |  |  | 22/22 (100) | 0/4 (0) |
|  |  |  |  |  |  |  |  |  |
| **Day 209** |  |  |  |  |  |  |  |  |
| N | 25 | 5 |  |  | 24 | 5 |  |  |
| GMC  (95% CI) | 518  (394–681) | <LLOQ (<LLOQ–71) | - | - | 821  (627–1074) | <LLOQ | - | - |
| Responder, n/N* (%) | 22/25 (88) | 0/5 (0) |  |  | 23/24 (96) | 0/5 (0) |  |  |
|  |  |  |  |  |  |  |  |  |
| **Day 237** |  |  |  |  |  |  |  |  |
| N |  |  | 29 | 4 |  |  | 23 | 4 |
| GMC  (95% CI) | - | - | 497  (391–633) | 81  (<LLOQ–1194) | - | - | 745  (591–939) | <LLOQ |
| Responder, n/N^a^ (%) |  |  | 27/29 (93) | 0/4 (0) |  |  | 21/22 (95) | 0/4 (0) |
|  |  |  |  |  |  |  |  |  |
| **Day 365** |  |  |  |  |  |  |  |  |
| N | 25 | 5 | 28 | 4 | 24 | 5 | 23 | 4 |
| GMC  (95% CI) | 537  (406–710) | <LLOQ (<LLOQ–40) | 453  (359–571) | 60  (<LLOQ–1117) | 1062  (753–1500) | <LLOQ | 653  (526–810) | <LLOQ (<LLOQ–191) |
| Responder, n/N* (%) | 22/25 (88) | 0/5 (0) | 26/28 (93) | 0/4 (0) | 23/24 (96) | 0/5 (0) | 21/22 (95) | 1/4 (25) |
|  |  |  |  |  |  |  |  |  |
| **Country: Côte d’Ivoire** |  |  |  |  |  |  |  |  |
| **Day 1** |  |  |  |  |  |  |  |  |
| N | 0 | 0 | 0 | 0 | 12 | 3 | 11 | 2 |
| GMC  (95% CI) |  |  |  |  | <LLOQ (<LLOQ–<LLOQ) | 38  (<LLOQ–873) | <LLOQ | <LLOQ |
| **Day 29** |  |  |  |  |  |  |  |  |
| N | 0 | 0 | - | - | 12 | 3 |  |  |
| GMC  (95% CI) |  |  |  |  | 684  (426–1099) | 163  (<LLOQ–54 969) | - | - |
| Responder, n/N* (%) |  |  |  |  | 12/12 (100) | 1/3 (33) |  |  |
|  |  |  |  |  |  |  |  |  |
| **Day 50** |  |  |  |  |  |  |  |  |
| N | 0 | 0 | - | - | 12 | 3 | - | - |
| GMC  (95% CI) |  |  |  |  | 6073  (3671–10 045) | 166  (<LLOQ–63 087) |  |  |
| Responder, n/N* (%) |  |  |  |  | 12/12 (100) | 1/3 (33) |  |  |
|  |  |  |  |  |  |  |  |  |
| **Day 57** |  |  |  |  |  |  |  |  |
| N | - | - | 0 | 0 | - | - | 12 | 2 |
| GMC  (95% CI) |  |  |  |  |  |  | 395  (251–621) | <LLOQ |
| Responder, n/N* (%) |  |  |  |  |  |  | 11/11 (100) | 0/2 (0) |
|  |  |  |  |  |  |  |  |  |
| **Day 78** |  |  |  |  |  |  |  |  |
| N | - | - | 0 | 0 | - | - | 12 | 2 |
| GMC  (95% CI) |  |  |  |  |  |  | 16 682  (7045–39 504) | <LLOQ |
| Responder, n/N* (%) |  |  |  |  |  |  | 11/11 (100) | 0/2 (0) |
|  |  |  |  |  |  |  |  |  |
| **Day 209** |  |  |  |  |  |  |  |  |
| N | 0 | 0 | - | - | 12 | 3 | - | - |
| GMC  (95% CI) |  |  |  |  | 787  (576–1076) | 129  (<LLOQ–41 985) |  |  |
| Responder, n/N* (%) |  |  |  |  | 12/12 (100) | 1/3 (33) |  |  |
|  |  |  |  |  |  |  |  |  |
| **Day 237** |  |  |  |  |  |  |  |  |
| N | - | - | 0 | 0 | - | - | 11 | 2 |
| GMC  (95% CI) |  |  |  |  |  |  | 591  (340–1029) | <LLOQ |
| Responder, n/N* (%) |  |  |  |  |  |  | 11/11 (100) | 0/2 (0) |
|  |  |  |  |  |  |  |  |  |
| **Day 365** |  |  |  |  |  |  |  |  |
| N | 0 | 0 | 0 | 0 | 12 | 3 | 12 | 2 |
| GMC  (95% CI) |  |  |  |  | 837  (613–1143) | 143  (<LLOQ–161 195) | 433  (235–797) | <LLOQ |
| Responder, n/N* (%) |  |  |  |  | 12/12 (100) | 1/3 (33) | 11/11 (100) | 0/2 (0) |
|  |  |  |  |  |  |  |  |  |
| **Country: Kenya** |  |  |  |  |  |  |  |  |
| **Day 1** |  |  |  |  |  |  |  |  |
| N | 12 | 3 | 12 | 2 | 5 | 1 | 5 | 1 |
| GMC  (95% CI) | <LLOQ | 41  (<LLOQ–1330) | <LLOQ (<LLOQ–46) | <LLOQ | <LLOQ | <LLOQ | <LLOQ | <LLOQ |
|  |  |  |  |  |  |  |  |  |
| **Day 29** |  |  |  |  |  |  |  |  |
| N | 12 | 3 | - | - | 5 | 1 | - | - |
| GMC  (95% CI) | 521  (283–959) | <LLOQ (<LLOQ–473) |  |  | 739  (270–2023) | <LLOQ |  |  |
| Responder, n/N* (%) | 11/12 (92) | 0/3 (0) |  |  | 5/5 (100) | 0/1 (0) |  |  |
|  |  |  |  |  |  |  |  |  |
| **Day 50** |  |  |  |  |  |  |  |  |
| N | 11 | 3 | - | - | 5 | 1 | - | - |
| GMC  (95% CI) | 8170  (4591–145 40) | 36  (<LLOQ–737) |  |  | 11 230  (3592–35 112) | <LLOQ |  |  |
| Responder, n/N* (%) | 11/11 (100) | 0/3 (0) |  |  | 5/5 (100) | 0/1 (0) |  |  |
|  |  |  |  |  |  |  |  |  |
| **Day 57** |  |  |  |  |  |  |  |  |
| N | - | - | 12 | 2 | - | - | 5 | 1 |
| GMC  (95% CI) |  |  | 586  (377–912) | <LLOQ |  |  | 1076  (671–1726) | <LLOQ |
| Responder, n/N* (%) |  |  | 12/12 (100) | 0/2 (0) |  |  | 5/5 (100) | 0/1 (0) |
|  |  |  |  |  |  |  |  |  |
| **Day 78** |  |  |  |  |  |  |  |  |
| N | - | - | 12 | 2 | - | - | 5 | 1 |
| GMC  (95% CI) |  |  | 14 875  (9157–24163) | <LLOQ |  |  | 20 489  (10 806–38 851) | <LLOQ |
| Responder, n/N* (%) |  |  | 12/12 (100) | 0/2 (0) |  |  | 5/5 (100) | 0/1 (0) |
|  |  |  |  |  |  |  |  |  |
| **Day 209** |  |  |  |  |  |  |  |  |
| N | 0 | 0 | - | - | 5 | 1 | - | - |
| GMC  (95% CI) |  |  |  |  | 808  (400–1630) | <LLOQ |  |  |
| Responder, n/N* (%) |  |  |  |  | 5/5 (100) | 0/1 (0) |  |  |
|  |  |  |  |  |  |  |  |  |
| **Day 237** |  |  |  |  |  |  |  |  |
| N | - | - | 0 | 0 | - | - | 5 | 1 |
| GMC  (95% CI) |  |  |  |  |  |  | 1026  (654–1610) | <LLOQ |
| Responder, n/N* (%) |  |  |  |  |  |  | 5/5 (100) | 0/1 (0) |
|  |  |  |  |  |  |  |  |  |
| **Day 365** |  |  |  |  |  |  |  |  |
| N | 10 | 2 | 12 | 2 | 5 | 1 | 5 | 1 |
| GMC  (95% CI) | 722  (396–1315) | 55  (<LLOQ–>ULOQ) | 716  (371–1380) | <LLOQ | 1179  (560–2479) | <LLOQ | 1056  (681–1638) | <LLOQ |
| Responder, n/N* (%) | 10/10 (100) | 0/2 (0) | 11/12 (92) | 0/2 (0) | 5/5 (100) | 0/1 (0) | 5/5 (100) | 0/1 (0) |
|  |  |  |  |  |  |  |  |  |
| **Country: Uganda** |  |  |  |  |  |  |  |  |
| **Day 1** |  |  |  |  |  |  |  |  |
| N | 16 | 2 | 12 | 4 | 12 | 3 | 14 | 4 |
| GMC  (95% CI) | <LLOQ (<LLOQ–<LLOQ) | 52  (<LLOQ–>ULOQ) | <LLOQ  (<LLOQ–49) | <LLOQ | <LLOQ  (<LLOQ–44) | <LLOQ | <LLOQ (<LLOQ–<LLOQ) | <LLOQ |
|  |  |  |  |  |  |  |  |  |
| **Day 29** |  |  |  |  |  |  |  |  |
| N | 16 | 2 | - | - | 12 | 3 | - | - |
| GMC  (95% CI) | 509  (347–747) | 45  (<LLOQ–>ULOQ) |  |  | 602  (357–1015) | <LLOQ |  |  |
| Responder, n/N* (%) | 16/16 (100) | 0/2 (0) |  |  | 12/12 (100) | 0/3 (0) |  |  |
|  |  |  |  |  |  |  |  |  |
| **Day 50** |  |  |  |  |  |  |  |  |
| N | 16 | 2 | - | - | 12 | 3 | - | - |
| GMC  (95% CI) | 5260  (2879–9611) | 61  (<LLOQ–>ULOQ) |  |  | 7598  (4748–12 158) | <LLOQ |  |  |
| Responder, n/N* (%) | 16/16 (100) | 0/2 (0) |  |  | 12/12 (100) | 0/3 (0) |  |  |
|  |  |  |  |  |  |  |  |  |
| **Day 57** |  |  |  |  |  |  |  |  |
| N | - | - | 12 | 4 | - | - | 14 | 4 |
| GMC  (95% CI) |  |  | 658  (303–1428) | <LLOQ (<LLOQ–185) |  |  | 689  (472–1004) | <LLOQ |
| Responder, n/N* (%) |  |  | 11/12 (92) | 1/4 (25) |  |  | 14/14 (100) | 0/4 (0) |
|  |  |  |  |  |  |  |  |  |
| **Day 78** |  |  |  |  |  |  |  |  |
| N | - | - | 12 | 4 | - | - | 13 | 4 |
| GMC  (95% CI) |  |  | 12408  (6488–23 730) | <LLOQ (<LLOQ–119) |  |  | 9627  (4870–19 029) | <LLOQ (<LLOQ–47) |
| Responder, n/N* (%) |  |  | 12/12 (100) | 1/4 (25) |  |  | 13/13 (100) | 0/4 (0) |
|  |  |  |  |  |  |  |  |  |
| **Day 209** |  |  |  |  |  |  |  |  |
| N | 16 | 2 | - | - | 11 | 3 | - | - |
| GMC  (95% CI) | 648  (479–877) | 71  (<LLOQ–94 219) |  |  | 969  (722–1302) | <LLOQ |  |  |
| Responder, n/N* (%) | 16/16 (100) | 0/2 (0) |  |  | 11/11 (100) | 0/3 (0) |  |  |
|  |  |  |  |  |  |  |  |  |
| **Day 237** |  |  |  |  |  |  |  |  |
| N | - | - | 12 | 4 | - | - | 14 | 4 |
| GMC  (95% CI) |  |  | 829  (458–1500) | <LLOQ (<LLOQ–122) |  |  | 683  (464–1005) | <LLOQ |
| Responder, n/N* (%) |  |  | 11/12 (92) | 1/4 (25) |  |  | 14/14 (100) | 0/4 (0) |
|  |  |  |  |  |  |  |  |  |
| **Day 365** |  |  |  |  |  |  |  |  |
| N | 15 | 2 | 12 | 4 | 12 | 3 | 14 | 4 |
| GMC  (95% CI) | 616  (380–1000) | 71  (<LLOQ–60346) | 622  (343–1127) | <LLOQ (<LLOQ–138) | 908  (623–1325) | <LLOQ | 711  (494–1023) | <LLOQ |
| Responder, n/N* (%) | 14/15 (93) | 0/2 (0) | 10/12 (83) | 1/4 (25) | 11/12 (92) | 0/3 (0) | 14/14 (100) | 0/4 (0) |
|  |  |  |  |  |  |  |  |  |

Abbreviations: CI, confidence interval, EBOV, Ebola virus; ELISA, enzyme-linked immunosorbent assay; GP, glycoprotein; GMC, geometric mean concentration; LLOQ, lower limit of quantification.

N, number of participants with available data at that time point; N*, number of participants with available data at baseline and at that time point.

Vaccines: Ad26.ZEBOV at dose of 5 x 10^10^ vp-MVA-BN-Filo at a dose of 1 x 10^8^ Inf.U.

| **Table K.** EBOV GP-specific Neutralising Antibody Responses (psVNA; IC_50_ Titre); Per Protocol Analysis Set | | | | |
| --- | --- | --- | --- | --- |
|  | **Adolescents (12–17 years)** | | **Children (4–11 years)** | |
|  | 28-day interval group | 56-day interval group | 28-day interval group | 56-day interval group |
|  | **Vaccines** | **Vaccines** | **Vaccines** | **Vaccines** |
| **Day 1** |  |  |  |  |
| N | 54 | 53 | 53 | 54 |
| GMT  (95% CI) | <LLOQ | <LLOQ | <LLOQ | <LLOQ |
|  |  |  |  |  |
| **Day 50** |  |  |  |  |
| N | 53 | - | 53 | - |
| GMT  (95% CI) | 1879  (1424–2478) |  | 2506  (1903–3300) |  |
| Responder, n/N* (%) | 51/53 (96) |  | 53/53 (100) |  |
|  |  |  |  |  |
| **Day 78** |  |  |  |  |
| N | - | 53 | - | 53 |
| GMT  (95% CI) |  | 6403  (5289–7751) |  | 8352  (6025–11 577) |
| Responder, n/N* (%) |  | 53/53 (100) |  | 51/53 (96) |
|  |  |  |  |  |
| **Day 365** |  |  |  |  |
| N | 50 | 52 | 52 | 54 |
| GMT  (95% CI) | 251  (191–331) | 218  (174–273) | 447  (371–539) | 275  (224–338) |
| Responder, n/N* (%) | 27/50 (54) | 23/52 (44) | 44/52 (85) | 32/54 (60) |

Abbreviations: CI, confidence interval; EBOV, Ebola virus; GMT, geometric mean titre; GP, glycoprotein; IC_50_, 50% inhibitory concentration; LLOQ, lower limit of quantification; psVNA, pseudovirion neutralisation assay.

N, number of participants with available data at that time point; N*, number of participants with available data at baseline and at that time point.

All GMTs were <LLOQ in all placebo groups, and therefore GMTs are not shown.

Vaccines: Ad26.ZEBOV at a dose of 5 x 10^10^ vp; MVA-BN-Filo at a dose of 1 x 10^8^ Inf.U.

| **Table L.** EBOV GP-specific Neutralising Antibody Responses (psVNA; IC_50_ Titre) by Country; Per Protocol Analysis Set | | | | | | | | |
| --- | --- | --- | --- | --- | --- | --- | --- | --- |
|  | **Adolescents (12–17 years)** | | | | **Children (4–11 years)** | | | |
|  | 28-day interval group | | 56-day interval group | | 28-day interval group | | 56-day interval group | |
|  | **Vaccines** | **Placebo** | **Vaccines** | **Placebo** | **Vaccines** | **Placebo** | **Vaccines** | **Placebo** |
| **Country: Burkina Faso** |  |  |  |  |  |  |  |  |
| **Day 1** |  |  |  |  |  |  |  |  |
| N | 26 | 5 | 29 | 4 | 24 | 5 | 23 | 4 |
| GMT  (95% CI) | <LLOQ | <LLOQ | <LLOQ | <LLOQ | <LLOQ | <LLOQ | <LLOQ | <LLOQ |
|  |  |  |  |  |  |  |  |  |
| **Day 50** |  |  |  |  |  |  |  |  |
| N | 26 | 5 | - | - | 24 | 5 | - | - |
| GMT  (95% CI) | 2095  (1569–2797) | <LLOQ |  |  | 2939  (1851–4666) | <LLOQ |  |  |
| Responder, n/N* (%) | 26/26 (100) | 0/5 (0) |  |  | 24/24 (100) | 0/5 (0) |  |  |
|  |  |  |  |  |  |  |  |  |
| **Day 78** |  |  |  |  |  |  |  |  |
| N | - | - | 29 | 4 | - | - | 23 | 4 |
| GMT  (95% CI) |  |  | 7116  (5607–9030) | <LLOQ |  |  | 11025  (7497–16 214) | <LLOQ |
| Responder, n/N* (%) |  |  | 29/29 (100) | 0/4 (0) |  |  | 23/23 (100) | 0/4 (0) |
|  |  |  |  |  |  |  |  |  |
| **Day 365** |  |  |  |  |  |  |  |  |
| N | 25 | 5 | 28 | 4 | 24 | 5 | 23 | 4 |
| GMT  (95% CI) | 193  (142–262) | <LLOQ | 181  (135–244) | <LLOQ | 452  (324–632) | <LLOQ | 294  (229–377) | <LLOQ |
| Responder, n/N* (%) | 10/25 (40) | 0/5 (0) | 9/28 (32) | 0/4 (0) | 20/24 (83) | 0/5 (0) | 15/23 (65) | 0/4 (0) |
|  |  |  |  |  |  |  |  |  |
| **Country: Côte d’Ivoire** |  |  |  |  |  |  |  |  |
| **Day 1** |  |  |  |  |  |  |  |  |
| N | 0 | 0 | 0 | 0 | 12 | 3 | 12 | 2 |
| GMT  (95% CI) |  |  |  |  | <LLOQ | <LLOQ | <LLOQ | <LLOQ |
|  |  |  |  |  |  |  |  |  |
| **Day 50** |  |  |  |  |  |  |  |  |
| N | 0 | 0 | - | - | 12 | 3 | - | - |
| GMT  (95% CI) |  |  |  |  | 1838  (1000–3377) | <LLOQ (<LLOQ–1637) |  |  |
| Responder, n/N* (%) |  |  |  |  | 12/12 (100) | 1/3 (33) |  |  |
|  |  |  |  |  |  |  |  |  |
| **Day 78** |  |  |  |  |  |  |  |  |
| N | - | - | 0 | 0 | - | - | 12 | 2 |
| GMT  (95% CI) |  |  |  |  |  |  | 6274  (2190–17 974) | <LLOQ |
| Responder, n/N* (%) |  |  |  |  |  |  | 11/12 (91.7) | 0/2 (0) |
|  |  |  |  |  |  |  |  |  |
| **Day 365** |  |  |  |  |  |  |  |  |
| N | 0 | 0 | 0 | 0 | 12 | 3 | 12 | 2 |
| GMT  (95% CI) |  |  |  |  | 448  (317–635) | <LLOQ (<LLOQ–643) | 192  (<LLOQ–349) | <LLOQ |
| Responder, n/N* (%) |  |  |  |  | 11/12 (92) | 0/3 (0) | 5/12 (42) | 0/2 (0) |
|  |  |  |  |  |  |  |  |  |
| **Country: Kenya** |  |  |  |  |  |  |  |  |
| **Day 1** |  |  |  |  |  |  |  |  |
| N | 12 | 3 | 12 | 2 | 5 | 1 | 5 | 1 |
| GMT  (95% CI) | <LLOQ | <LLOQ | <LLOQ | <LLOQ | <LLOQ | <LLOQ | <LLOQ | <LLOQ |
|  |  |  |  |  |  |  |  |  |
| **Day 50** |  |  |  |  |  |  |  |  |
| N | 11 | 3 | - | - | 5 | 1 | - | - |
| GMT  (95% CI) | 2487  (1594–3878) | <LLOQ |  |  | 4642  (1254–17 186) | <LLOQ |  |  |
| Responder, n/N* (%) | 11/11 (100) | 0/3 (0) |  |  | 5/5 (100) | 0/1 (0) |  |  |
|  |  |  |  |  |  |  |  |  |
| **Day 78** |  |  |  |  |  |  |  |  |
| N | - | - | 12 | 2 | - | - | 5 | 1 |
| GMT  (95% CI) |  |  | 6656  (4307–10 287) | <LLOQ |  |  | 11783  (6074–22 861) | <LLOQ |
| Responder, n/N* (%) |  |  | 12/12 (100) | 0/2 (0) |  |  | 5/5 (100) | 0/1 (0) |
|  |  |  |  |  |  |  |  |  |
| **Day 365** |  |  |  |  |  |  |  |  |
| N | 10 | 2 | 12 | 2 | 5 | 1 | 5 | 1 |
| GMT  (95% CI) | 447  (244–819) | <LLOQ | 283  (153–521) | <LLOQ | 460  (216–978) | <LLOQ | 466  (293–741) | <LLOQ |
| Responder, n/N* (%) | 8/10 (80) | 0/2 (0) | 6/12 (50) | 0/2 (0) | 4/5 (80) | 0/1 (0) | 5/5 (100) | 0/1 (0) |
|  |  |  |  |  |  |  |  |  |
| **Country: Uganda** |  |  |  |  |  |  |  |  |
| **Day 1** |  |  |  |  |  |  |  |  |
| N | 16 | 2 | 12 | 4 | 12 | 3 | 14 | 4 |
| GMT  (95% CI) | <LLOQ | <LLOQ | <LLOQ | <LLOQ | <LLOQ | <LLOQ | <LLOQ | <LLOQ |
|  |  |  |  |  |  |  |  |  |
| **Day 50** |  |  |  |  |  |  |  |  |
| N | 16 | 2 | - | - | 12 | 3 | - | - |
| GMT  (95% CI) | 1297  (596–2823) | <LLOQ |  |  | 1922  (1240–2977) | <LLOQ |  |  |
| Responder, n/N* (%) | 14/16 (88) | 0/2 (0) |  |  | 12/12 (100) | 0/3 (0) |  |  |
|  |  |  |  |  |  |  |  |  |
| **Day 78** |  |  |  |  |  |  |  |  |
| N | - | - | 12 | 4 | - | - | 13 | 4 |
| GMT  (95% CI) |  |  | 4772  (2828–8051) | <LLOQ |  |  | 5829  (2717–12 505) | <LLOQ |
| Responder, n/N* (%) |  |  | 12/12 (100) | 0/4 (0) |  |  | 12/13 (92) | 0/4 (0) |
|  |  |  |  |  |  |  |  |  |
| **Day 365** |  |  |  |  |  |  |  |  |
| N | 15 | 2 | 12 | 4 | 11 | 2 | 14 | 4 |
| GMT  (95% CI) | 266  (134–528) | <LLOQ | 258  (166–401) | <LLOQ | 429  (286–646) | <LLOQ | 279  (173–451) | <LLOQ |
| Responder, n/N* (%) | 9/15 (60) | 0/2 (0) | 8/12 (67) | 0/4 (0) | 9/11 (82) | 0/2 (0) | 7/14 (50) | 0/4 (0) |
|  |  |  |  |  |  |  |  |  |

Abbreviations: CI, confidence interval; EBOV, Ebola virus; GMT, geometric mean titre; GP, glycoprotein; IC_50_, 50% inhibitory concentration; LLOQ, lower limit of quantification; psVNA, pseudovirion neutralisation assay.N, number of participants with available data at that time point; N*, number of participants with available data at baseline and at that time point.

Vaccines: Ad26.ZEBOV at dose of 5x10^10^ vp; MVA-BN-Filo at a dose of 1x10^8^ Inf.U.

| **Table M.** Ad26 Neutralising Antibodies (Ad26 VNA; IC_90_ Titre); Per Protocol Analysis Set | | | | | |
| --- | --- | --- | --- | --- | --- |
| **Adolescents (12–17 years)** | | | | | |
|  | 28-day interval group | | 56-day interval group | | All participants |
|  | **Vaccines** | **Placebo** | **Vaccines** | **Placebo** |  |
| **Day 1** |  |  |  |  |  |
| N | 54 | 10 | 53 | 10 | 127 |
| GMT  (95% CI) | 127  (85–190) | 62  (23–171) | 116  (76–176) | 82  (28–242) | 112  (86–145) |
| Positive Samples, n (%) | 48 (89) | 8 (80) | 47 (89) | 8 (80) | 111 (87) |
|  |  |  |  |  |  |
| **Children (4–11 years)** | | | | | |
|  | 28-day group | | 56-day group | | All participants |
|  | **Vaccines** | **Placebo** | **Vaccines** | **Placebo** |  |
| **Day 1** |  |  |  |  |  |
| N | 53 | 12 | 54 | 11 | 130 |
| GMT  (95% CI) | 67  (41–111) | 144  (40–523) | 92  (53–157) | 118  (24–577) | 86  (61–121) |
| Positive Samples, n (%) | 37 (70) | 9 (75) | 39 (72) | 7 (64) | 92 (71) |
|  |  |  |  |  |  |

Abbreviations: A26 VNA, Ad26-specific virus neutralisation assay; CI, confidence interval; GMT, geometric mean titre; IC_90_, 90% inhibitory concentration .

N: number of participants with available data at that time point.

Vaccines: Ad26.ZEBOV at a dose of 5x10^10^ vp; MVA-BN-Filo at a dose of 1x10^8^ Inf.U.

| **Table N.** EBOV GP-specific CD4+ T Cell Cytokine Responses (ICS, % of Subset); Per Protocol Analysis Set | | | | | | | | |
| --- | --- | --- | --- | --- | --- | --- | --- | --- |
|  | **Adolescents (12–17 Years)** | | | | **Children (4–11 Years)** | | | |
|  | 28-day interval group | | 56-day interval group | | 28-day interval group | | 56-day interval group | |
|  | **Vaccines** | **Placebo** | **Vaccines** | **Placebo** | **Vaccines** | **Placebo** | **Vaccines** | **Placebo** |
| **Day 1** |  |  |  |  |  |  |  |  |
| N | 5 | 1 | 5 | 3 | 12 | 2 | 10 | 2 |
| Median | <threshold | <threshold | <threshold | <threshold | <threshold | <threshold | <threshold | <threshold |
| (IQR) | (<threshold–<threshold) | (<threshold–<threshold) | (<threshold–<threshold) | (<threshold–<threshold) | (<threshold–0.07) | (<threshold–0.05) | (<threshold–<threshold) | (<threshold–0.04) |
|  |  |  |  |  |  |  |  |  |
| **Day 50** |  |  |  |  |  |  |  |  |
| N | 12 | 3 | - | - | 11 | 1 | - | - |
| Median | 0.08 | <threshold |  |  | 0.19 | < threshold |  |  |
| (IQR) | (<threshold–0.17) | (<threshold–<threshold) |  |  | (0.10–0.30) | (<threshold–<threshold) |  |  |
| Responder, n/N* (%) | 2/5 (40) | 0/1 (0) |  |  | 6/10 (60) | 0/1 (0) |  |  |
|  |  |  |  |  |  |  |  |  |
| **Day 78** |  |  |  |  |  |  |  |  |
| N | - | - | 12 | 2 | - | - | 10 | 2 |
| Median |  |  | 0.08 | <LLOQ |  |  | 0.07 | 0.06 |
| (IQR) |  |  | (<threshold–0.11) | (<threshold–<threshold) |  |  | (<threshold-0.23) | (0.04-0.08) |
| Responder, n/N* (%) |  |  | 2/5 (40) | 0/2 (0) |  |  | 3/9 (33) | 0/1 (0) |
|  |  |  |  |  |  |  |  |  |
| **Day 209** |  |  |  |  |  |  |  |  |
| N | 11 | 2 | - | - | 10 | 1 | - | - |
| Median | <threshold | <threshold |  |  | 0.08 | <threshold |  |  |
| (IQR) | (<threshold–<threshold) | (<threshold–<threshold) |  |  | (<threshold–0.12) | (<threshold–<threshold) |  |  |
| Responder, n/N* (%) | 0/5 (0) |  |  |  | 2/9 (22) | 0/1 (0) |  |  |
|  |  |  |  |  |  |  |  |  |
| **Day 237** |  |  |  |  |  |  |  |  |
| N | - | - | 10 | 2 | - | - | 10 | 2 |
| Median |  |  | <threshold | <threshold |  |  | 0.08 | 0.04 |
| (IQR) |  |  | (<threshold–<threshold) | (<threshold–<threshold) |  |  | (<threshold–0.17) | (<threshold–0.06) |
| Responder, n/N* (%) |  |  | 0/4 (0) | 0/2 (0) |  |  | 2/10 (20) | 0/1 (0) |
|  |  |  |  |  |  |  |  |  |
| **Day 365** |  |  |  |  |  |  |  |  |
| N | 5 | 0 | 7 | 2 | 9 | 1 | 9 | 2 |
| Median | <threshold |  | 0.05 | <threshold | <threshold | <threshold | <threshold | <threshold |
| (IQR) | (<threshold–<threshold) |  | (<threshold–0.13) | (<threshold–<threshold) | (<threshold–<threshold) | (<threshold–<threshold) | (<threshold–0.07) | (<threshold–<threshold) |
| Responder, n/N* (%) | 0/4 (0) |  | 0/5 (0) | 0/2 (0) | 0/9 (0) | 0/1 (0) | 1/9 (11) | 0/2 (0) |
|  |  |  |  |  |  |  |  |  |

Abbreviations: EBOV, Ebola virus; GP, glycoprotein; ICS, intracellular cytokine staining; IQR, interquartile range.Total background-adjusted percentage (interferon [IFN]γ+ or interleukin [IL]2+ or tumour necrosis factor [TNF]α+) data are shown.

N, number of subjects with data at that time point; N*, number of subjects with data at baseline and at that time point.

Threshold, Sponsor-defined threshold (0.04%).

Vaccines: Ad26.ZEBOV at a dose of 5x10^10^ vp; MVA-BN-Filo at a dose of 1x10^8^ Inf.U.

| **Table O.** EBOV GP-specific CD8+ T Cell Cytokine Responses (ICS, % of Subset); Per Protocol Analysis Set | | | | | | | | |
| --- | --- | --- | --- | --- | --- | --- | --- | --- |
|  | **Adolescents (12–17 Years)** | | | | **Children (4–11 Years)** | | | |
|  | 28-day interval group | | 56-day interval group | | 28-day interval group | | 56-day interval group | |
|  | **Vaccines** | **Placebo** | **Vaccines** | **Placebo** | **Vaccines** | **Placebo** | **Vaccines** | **Placebo** |
| **Day 1** |  |  |  |  |  |  |  |  |
| N | 9 | 2 | 9 | 4 | 12 | 2 | 9 | 2 |
| Median | <threshold | <threshold | <threshold | <threshold | <threshold | <threshold | <threshold | <threshold |
| (IQR) | (<threshold–<threshold) | (<threshold–<threshold) | (<threshold–<threshold) | (<threshold–<threshold) | (<threshold–<threshold) | (<threshold–<threshold) | (<threshold–<threshold) | (<threshold–<threshold) |
|  |  |  |  |  |  |  |  |  |
| **Day 50** |  |  |  |  |  |  |  |  |
| N | 14 | 3 | - | - | 12 | 1 | - | - |
| Median | <threshold | <threshold |  |  | <threshold | <threshold |  |  |
| (IQR) | (<threshold–0.05) | (<threshold–<threshold) |  |  | (<threshold–0.11) | (<threshold–<threshold) |  |  |
| Responder, n/N* (%) | 0/9 (0) | 0/2 (0) |  |  | 2/11 (18) | 0/1 (0) |  |  |
|  |  |  |  |  |  |  |  |  |
| **Day 78** |  |  |  |  |  |  |  |  |
| N | - | - | 15 | 3 | - | - | 10 | 3 |
| Median |  |  | <threshold | <threshold |  |  | <threshold | <threshold |
| (IQR) |  |  | (<threshold–<threshold) | (<threshold–<threshold) |  |  | (<threshold–<threshold) | (<threshold–<threshold) |
| Responder, n/N* (%) |  |  | 0/9 (0) | 0/3 (0) |  |  | 0/8 (0) | 0/2 (0) |
|  |  |  |  |  |  |  |  |  |
| **Day 209** |  |  |  |  |  |  |  |  |
| N | 13 | 2 | - | - | 11 | 1 | - | - |
| Median | <threshold | <threshold |  |  | <threshold | <threshold |  |  |
| (IQR) | (<threshold–<threshold) | (<threshold–<threshold) |  |  | (<threshold–0.04) | (<threshold–<threshold) |  |  |
| Responder, n/N* (%) | 0/9 (0) | 0/1 (0) |  |  | 1/10 (10) | 0/1 (0) |  |  |
|  |  |  |  |  |  |  |  |  |
| **Day 237** |  |  |  |  |  |  |  |  |
| N | - | - | 15 | 4 | - | - | 10 | 3 |
| Median |  |  | <threshold | <threshold |  |  | <threshold | <threshold |
| (IQR) |  |  | (<threshold–<threshold) | (<threshold–<threshold) |  |  | (<threshold–<threshold) | (<threshold–<threshold) |
| Responder, n/N* (%) |  |  | 0/9 (0) | 0/4 (0) |  |  | 0/9 (0) | 0/2 (0) |
|  |  |  |  |  |  |  |  |  |
| **Day 365** |  |  |  |  |  |  |  |  |
| N | 9 | 1 | 11 | 3 | 9 | 1 | 9 | 2 |
| Median | <threshold | <threshold | <threshold | <threshold | <threshold | <threshold | <threshold | <threshold |
| (IQR) | (<threshold–<threshold) | (<threshold–<threshold) | (<threshold–<threshold) | (<threshold–<threshold) | (<threshold–<threshold) | (<threshold–<threshold) | (<threshold–<threshold) | (<threshold–<threshold) |
| Responder, n/N* (%) | 0/7 (0) | 0/1 (0) | 0/8 (0) | 0/3 (0) | 2/9 (22) | 0/1 (0) | 0/9 (0) | 0/2 (0) |
|  |  |  |  |  |  |  |  |  |

Abbreviations: EBOV, Ebola virus; GP, glycoprotein; ICS, intracellular cytokine staining; IQR, interquartile range.Total background-adjusted percentage (interferon [IFN]γ+ or interleukin [IL]2+ or tumour necrosis factor [TNF]α+) data are shown.

N, number of subjects with data at that time point; N*, number of subjects with data at baseline and at that time point.

Threshold, Sponsor-defined threshold (0.04%).

Vaccines: Ad26.ZEBOV at a dose of 5x10^10^ vp; MVA-BN-Filo at a dose of 1x10^8^ Inf.U.

| **Table P.** EBOV GP-specific IFN-γ Producing T Cell Responses (IFN-γ ELISpot, SFU/10^6^ PBMC); Per Protocol Analysis Set | | | | | | | | |
| --- | --- | --- | --- | --- | --- | --- | --- | --- |
|  | **Adolescents (12–17 Years)** | | | | **Children (4–11 Years)** | | | |
|  | 28-day interval group | | 56-day interval group | | 28-day interval group | | 56-day interval group | |
| **ELISpot – Pool 1 + 2** | **Vaccines** | **Placebo** | **Vaccines** | **Placebo** | **Vaccines** | **Placebo** | **Vaccines** | **Placebo** |
| **Day 1** |  |  |  |  |  |  |  |  |
| N | 8 | 1 | 8 | 4 | 6 | 0 | 9 | 0 |
| Median | <50 | <50 | <50 | <50 | <50 |  | <50 |  |
| (IQR) | (<50–<50) | (<50–<50) | (<50–<50) | (<50–<50) | (<50–<50) |  | (<50–<50) |  |
|  |  |  |  |  |  |  |  |  |
| **Day 50** |  |  |  |  |  |  |  |  |
| N | 14 | 1 | - | - | 10 | 0 | - | - |
| Median | 63 | <50 |  |  | 66 |  |  |  |
| (IQR) | (<50–152) | (<50–<50) |  |  | (<50–130) |  |  |  |
|  |  |  |  |  |  |  |  |  |
| Responder, n/N* (%) | 1/8 (13) | 0/1 (0) |  |  | 3/6 (50) |  |  |  |
|  |  |  |  |  |  |  |  |  |
| **Day 78** |  |  |  |  |  |  |  |  |
| N | - | - | 14 | 3 | - | - | 9 | 1 |
| Median |  |  | 99 | <50 |  |  | 70 | <50 |
| (IQR) |  |  | (<50–122) | (<50–57) |  |  | (<50–117) | (<50–<50) |
|  |  |  |  |  |  |  |  |  |
| Responder, n/N* (%) |  |  | 2/7 (29) | 0/3 (0) |  |  | 2/8 (25) |  |
|  |  |  |  |  |  |  |  |  |
| **Day 209** |  |  |  |  |  |  |  |  |
| N | 11 | 1 | - | - | 8 | 1 | - | - |
| Median | <50 | <50 |  |  | <50 | <50 |  |  |
| (IQR) | (<50–68) | (<50–<50) |  |  | (<50–<50) | (<50–<50) |  |  |
|  |  |  |  |  |  |  |  |  |
| Responder, n/N* (%) | 0/5 (0) | 0/1 (0) |  |  | 0/5 (0) |  |  |  |
|  |  |  |  |  |  |  |  |  |
| **Day 237** |  |  |  |  |  |  |  |  |
| N | - | - | 14 | 3 | - | - | 9 | 2 |
| Median |  |  | <50 | <50 |  |  | <50 | <50 |
| (IQR) |  |  | (<50-67) | (<50-<50) |  |  | (<50–<50) | (<50–<50) |
|  |  |  |  |  |  |  |  |  |
| Responder, n/N* (%) |  |  | 1/8 (12) | 0/3 (0) |  |  | 0/9 (0) |  |
|  |  |  |  |  |  |  |  |  |
| **Day 365** |  |  |  |  |  |  |  |  |
| N | 8 | 1 | 7 | 2 | 7 | 0 | 8 | 1 |
| Median | <50 | <50 | <50 | <50 | <50 |  | <50 | <50 |
| (IQR) | (<50–57) | (<50–<50) | (<50–<50) | (<50–<50) | (<50–<50) |  | (<50–<50) | (<50–<50) |
|  |  |  |  |  |  |  |  |  |
| Responder, n/N* (%) | 0/6 (0) | 0/1 (0) | 0/5 (0) | 0/2 (0) | 0/4 (0) |  | 0/8 (0) |  |
|  |  |  |  |  |  |  |  |  |

Abbreviations: CI, confidence interval; EBOV, Ebola virus; ELISpot, enzyme-linked immunospot; GP, glycoprotein; IFN-γ, interferon-γ; IQR, interquartile range; SFU/10^6^ PBMC, spot-forming units per million peripheral blood mononuclear cells.

N, number of participants with available data at that time point; N*, number of participants with available data at baseline and at that time point.

Vaccines: Ad26.ZEBOV at a dose of 5x10^10^ vp; MVA-BN-Filo at a dose of 1x10^8^ Inf.U.

**Fig A**. EBOV GP-specific Neutralising Antibody Responses – Regimen Plot (psVNA; IC_50_ Titre); Per Protocol Analysis Set


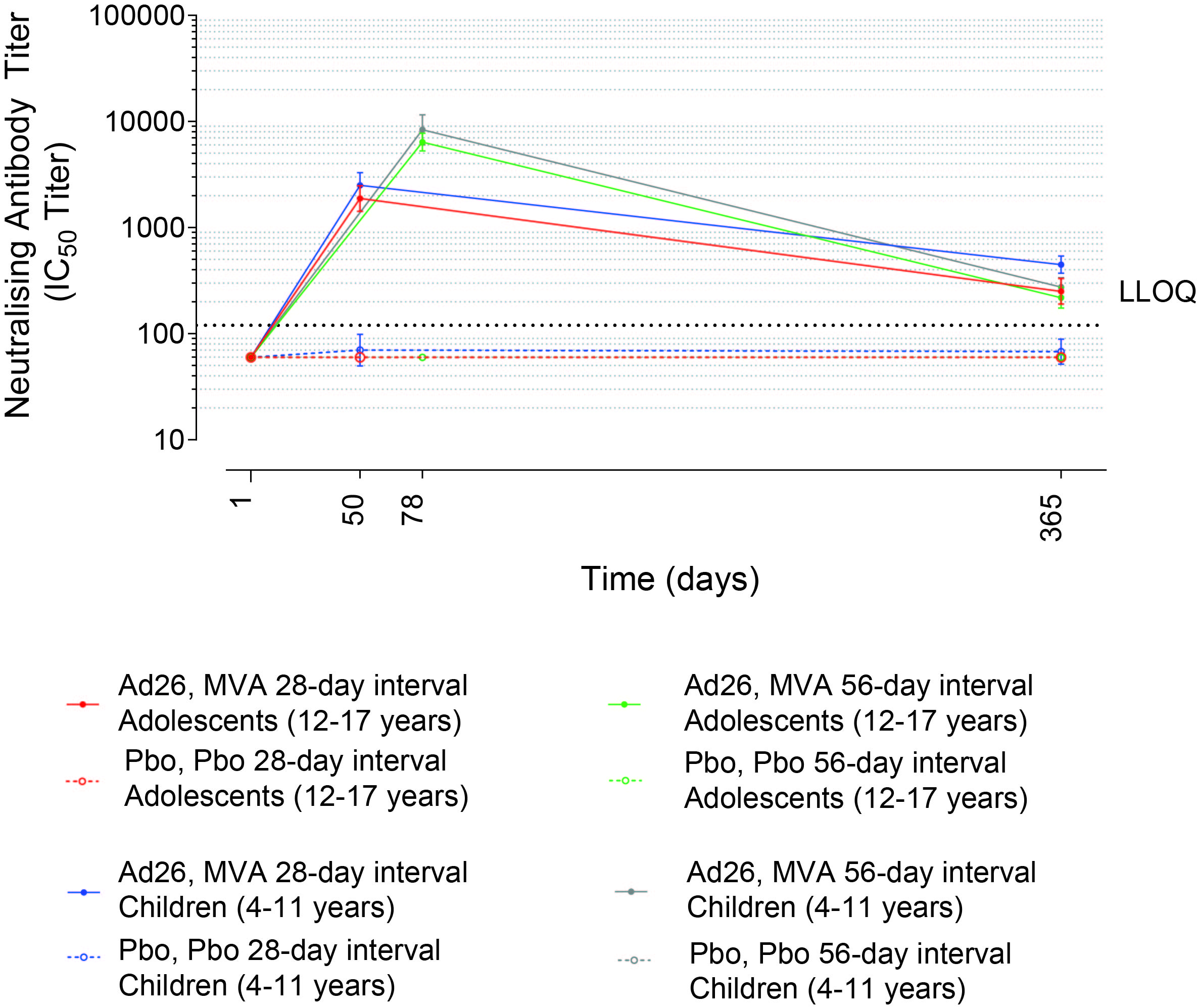


Participants administered with Ad26.ZEBOV or placebo on Day 1 and MVA-BN-Filo or placebo 28 or 56 days later as indicated. Responses are expressed as geometric mean titres (IC_50_ titre, 95% CI). Responses in placebo groups are shown as open symbols.

Grey dotted line represents the LLOQ. The points (symbols) denote GMTs and error bars denote 95% CIs.

Ad26: Ad26.ZEBOV at a dose of 5x10^10^ vp; MVA: MVA-BN-Filo at a dose of 1x10^8^ Inf.U.

EBOV, Ebola virus; GP, glycoprotein; IC_50_, 50% inhibitory concentration; LLOQ, lower limit of quantitation; Pbo, placebo; psVNA, pseudovirion neutralisation assay.

**Fig B**. Spearman Correlation Between EBOV GP-specific Binding and Neutralising Antibody Responses 21 Days Post-MVA-BN-Filo; Per Protocol Analysis Set

1. 21 days post-dose 2
2. 364 days post-dose 1


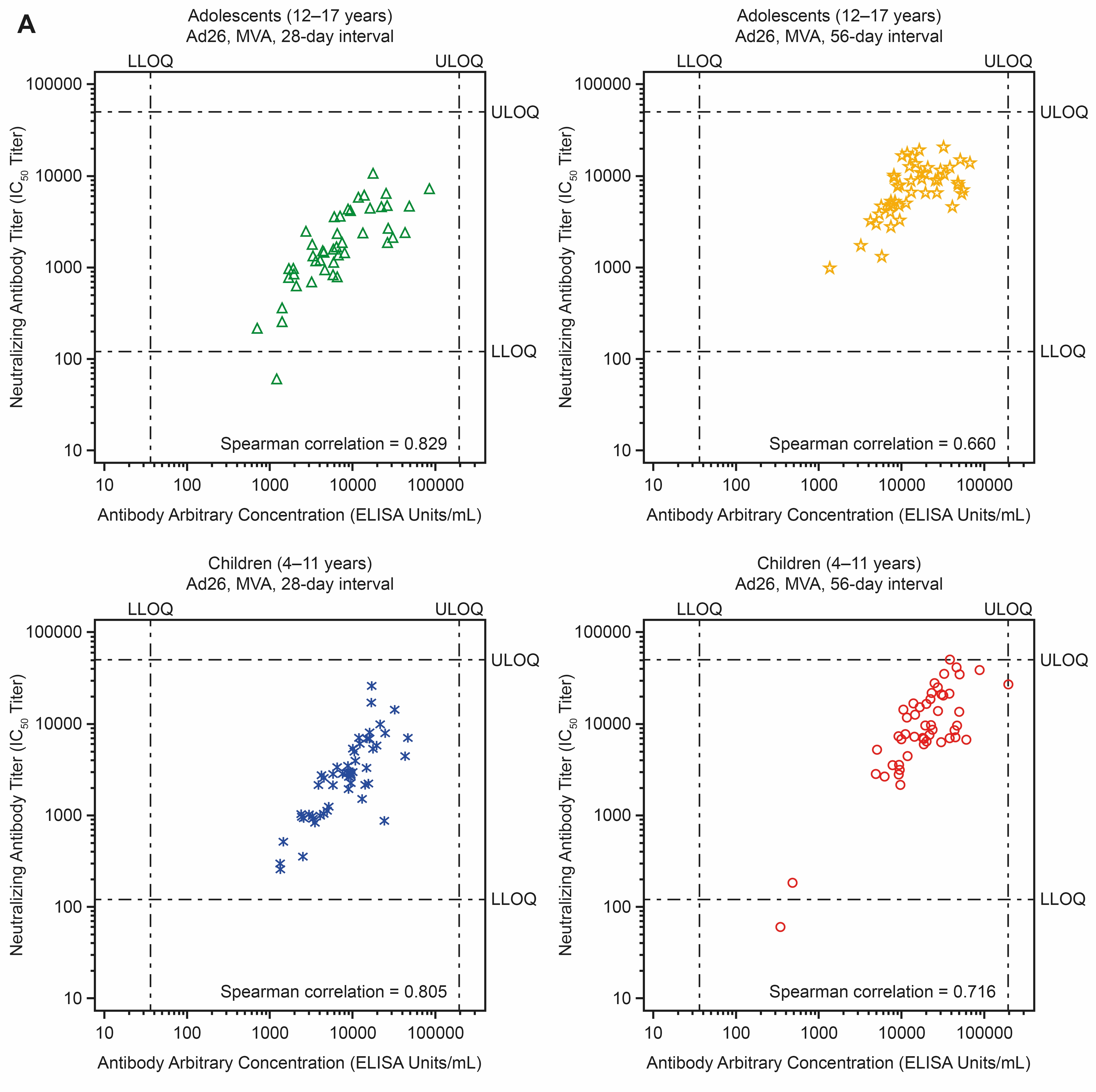


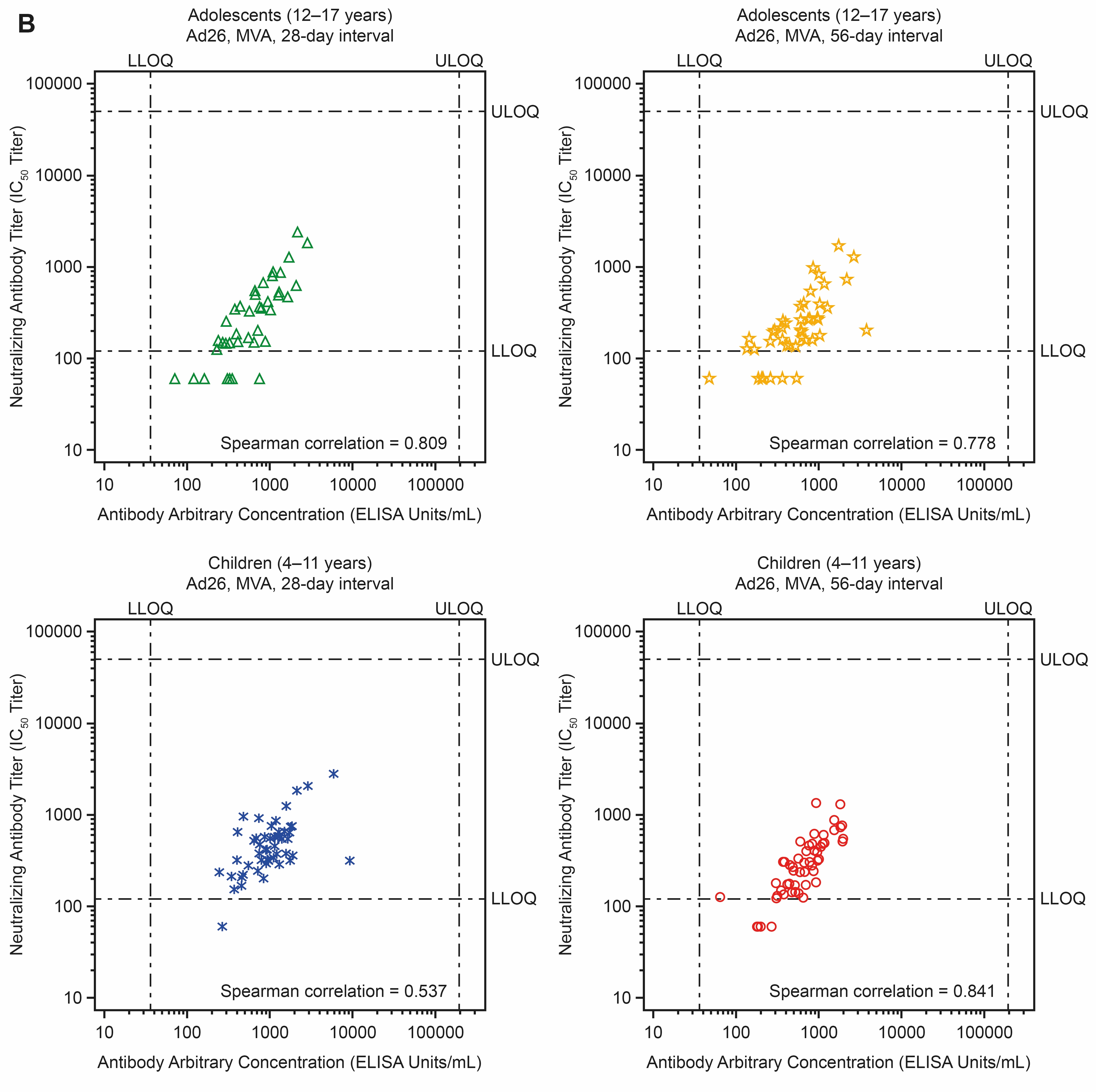


Placebo subjects are excluded from this display.

Ad26: Ad26.ZEBOV at a dose of 5x10^10^ vp; MVA: MVA-BN-Filo at a dose of 1x10^8^ Inf.U.

EBOV, Ebola virus; ELISA, enzyme-linked immunosorbent assay; GP, glycoprotein; LLOQ, lower limit of quantitation; ULOQ, upper limit of quantification.

**Fig C**. Correlations between Ad26-specific neutralising antibody titres at baseline and EBOV GP-specific binding and neutralising antibodies 21 days post-dose 2

A) Anti EBOV GP IgG ELISA at 21 days post-dose 2 by Ad26 neutralisation assay at baseline

B) EBOV GP neutralisation assay at 21 days post-dose 2 by Ad26 neutralisation assay at baseline


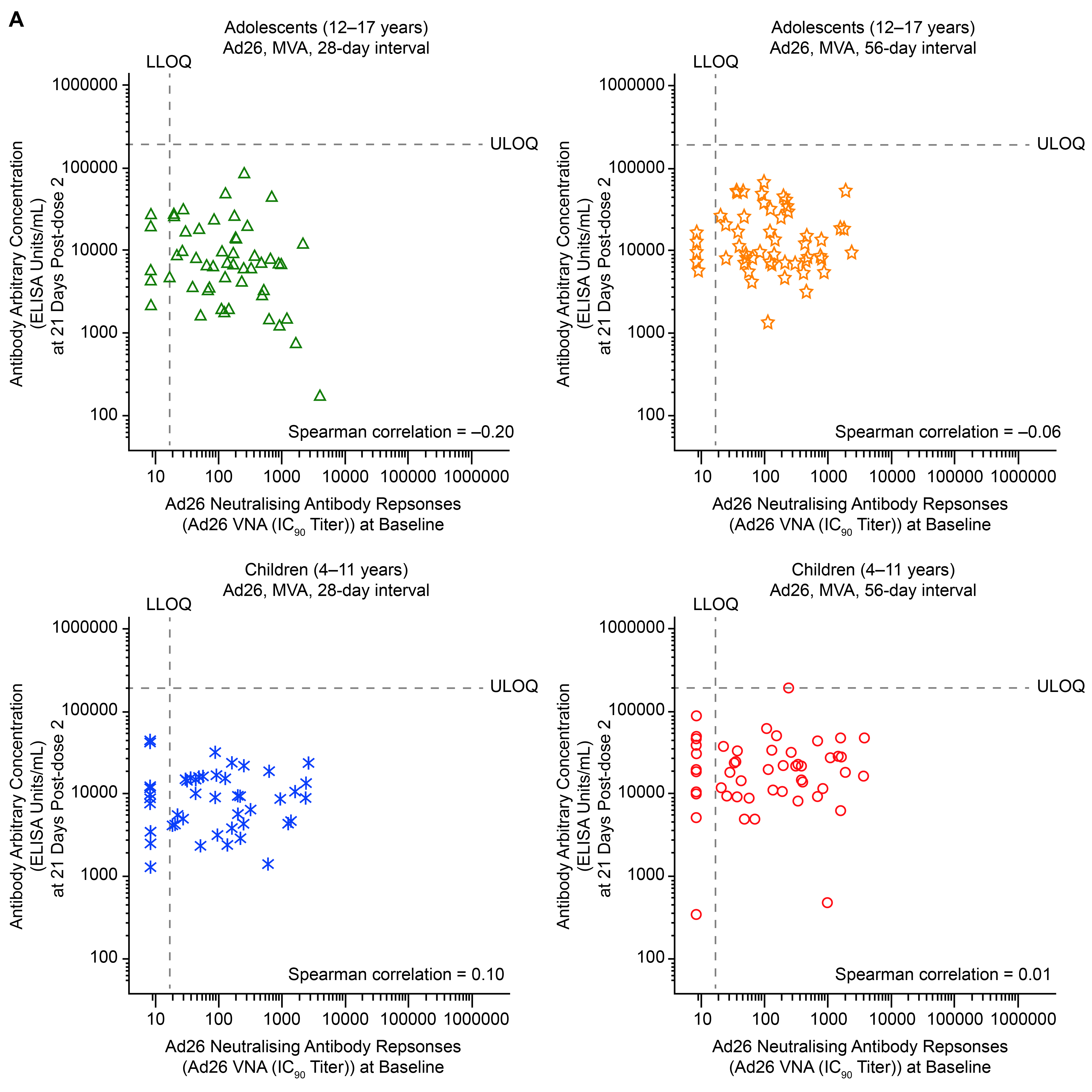


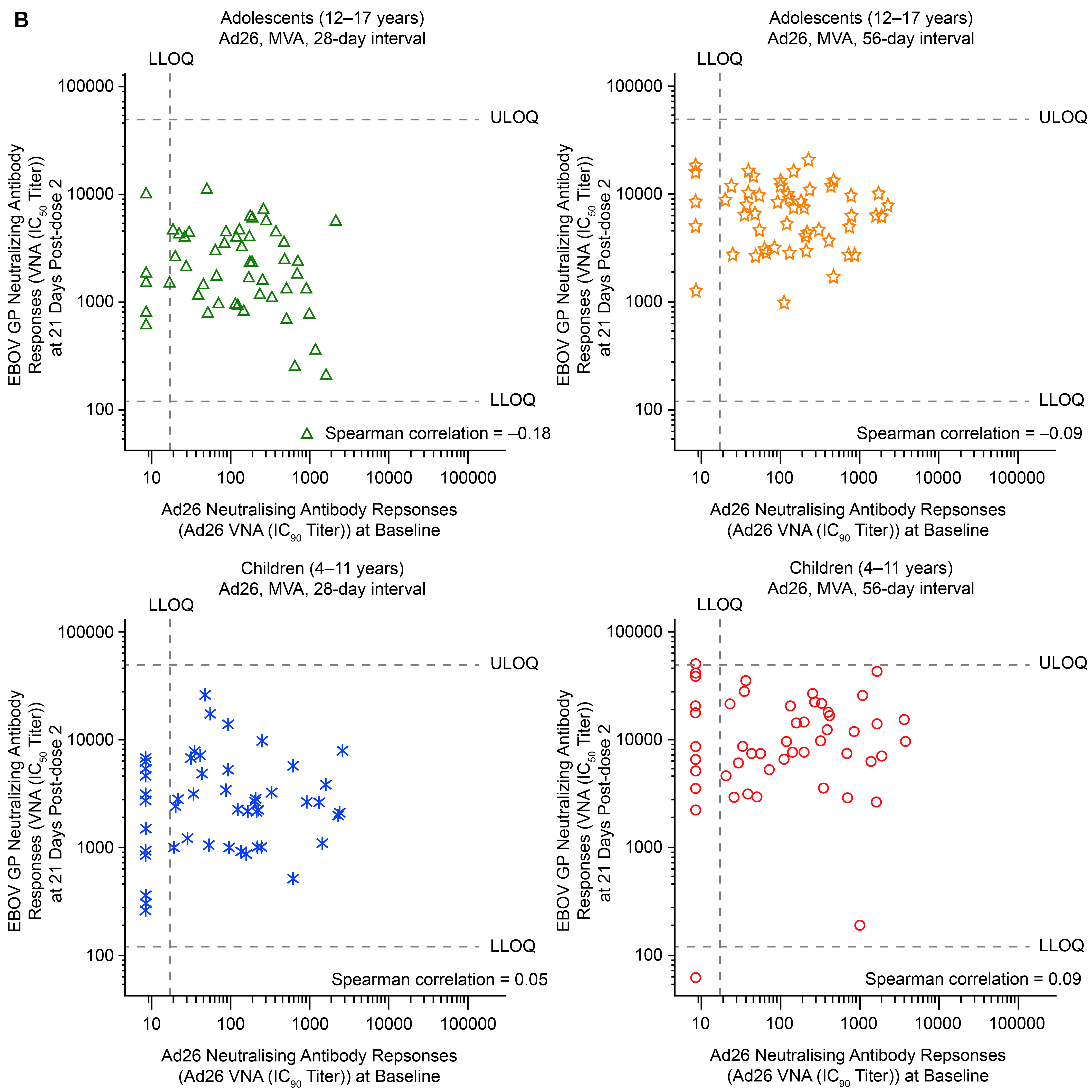


Placebo recipients are excluded from this display.

Ad26: Ad26.ZEBOV at a dose of 5x10^10^ vp; MVA: MVA-BN-Filo at a dose of 1x10^8^ Inf.U.

EBOV, Ebola virus; ELISA, enzyme-linked immunosorbent assay; GP, glycoprotein; IC90, 90% inhibitory concentration; LLOQ, lower limit of quantitation; ULOQ, upper limit of quantification; VNA, virus neutralisation assay.

**Fig D**. CD4+ and CD8+ T Cell Responses in Adolescents (ICS)

CD4+ (**A**) and CD8+ (**B**) T cell responses in the subsets of adolescents (12–17 years) from the 28- and 56-day interval groups and placebo.


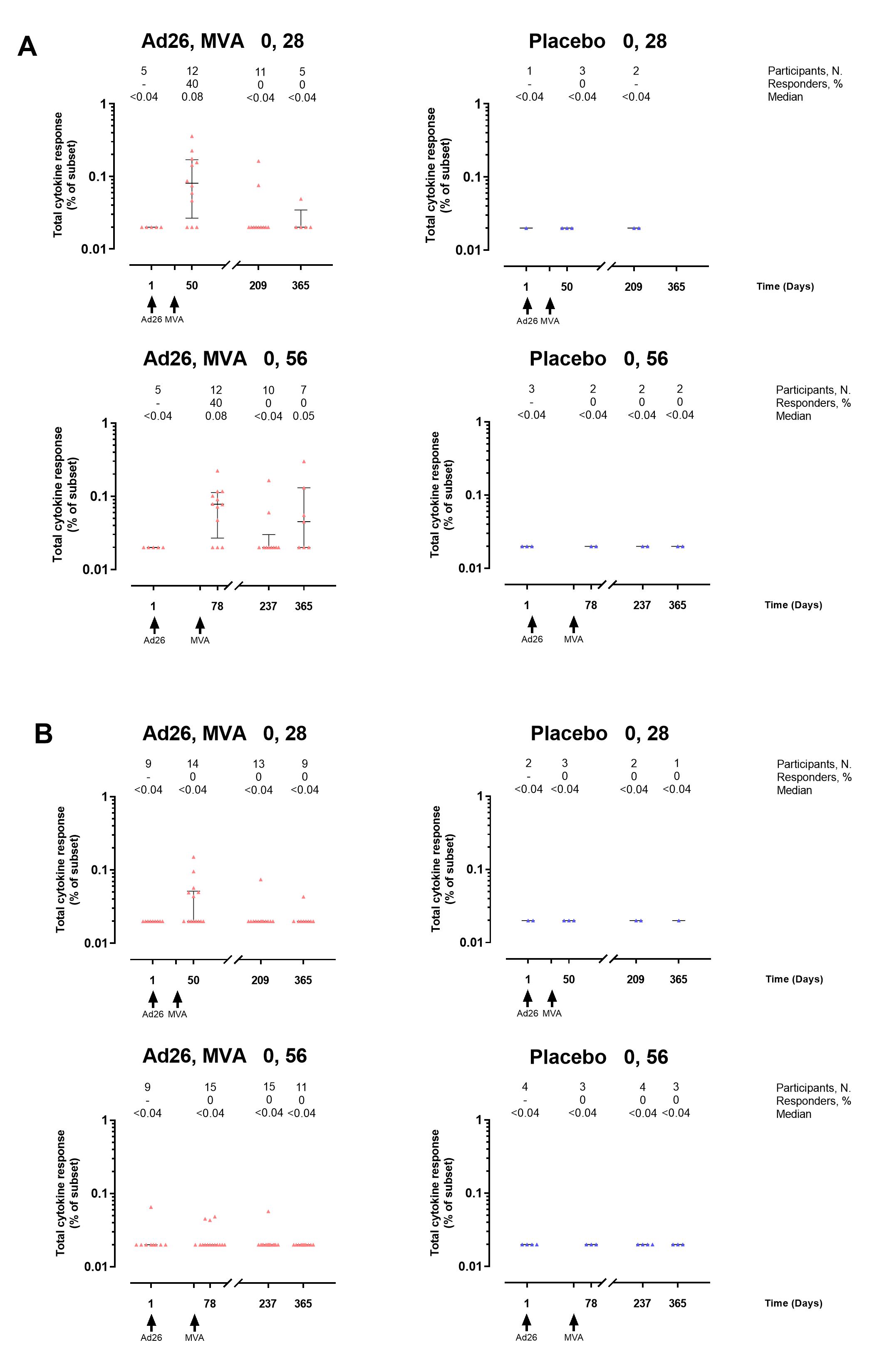


The vertical lines represent the IQR (Q1–Q3), where the horizontal lines depict the lower quartile (Q1), and upper quartile (Q3). The middle horizontal line depicts the median (Q2).

Ad26: Ad26.ZEBOV at a dose of 5x10^10^ vp; MVA: MVA-BN-Filo at a dose of 1x10^8^ Inf.U.

N, number of participants with data at that timepoint.

**Fig E**. CD4+ and CD8+ T Cell Responses in Children (ICS)

CD4+ (panel **A**) and CD8+ (panel **B**) T cell responses in the subsets of children (4–11 years) from the 28- and 56-day interval groups and placebo.


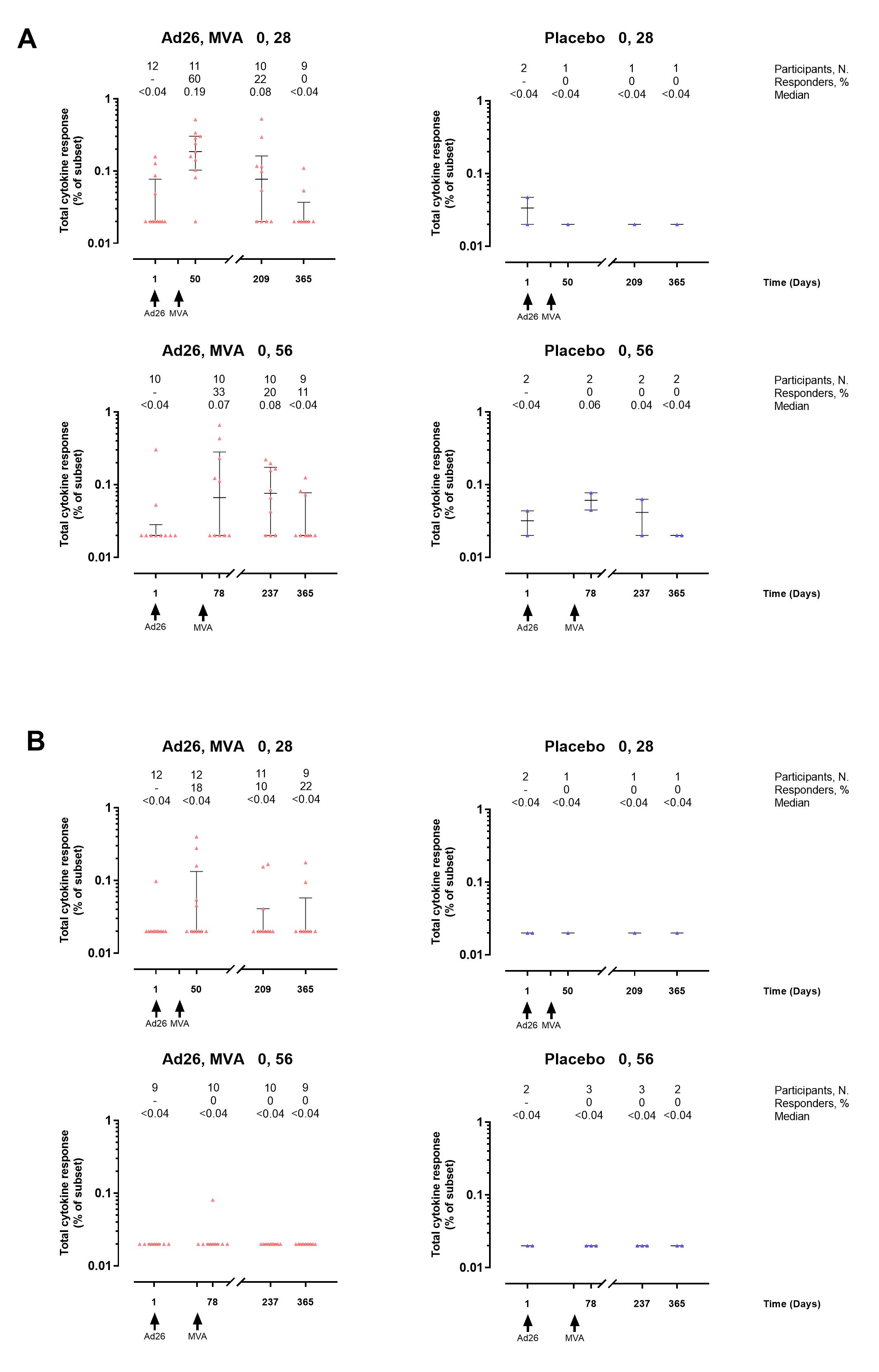


The vertical lines represent the IQR (Q1–Q3), where the horizontal lines depict the lower quartile (Q1), and upper quartile (Q3). The middle horizontal line depicts the median (Q2).

Ad26: Ad26.ZEBOV at a dose of 5x10^10^ vp; MVA: MVA-BN-Filo at a dose of 1x10^8^ Inf.U.

N, number of participants with data at that timepoint.

**Fig F.** EBOV GP-specific IFN-γ Producing T Cell Responses (ELISpot)

1. Adolescents (12–17 years)
2. Children (4–11 years)


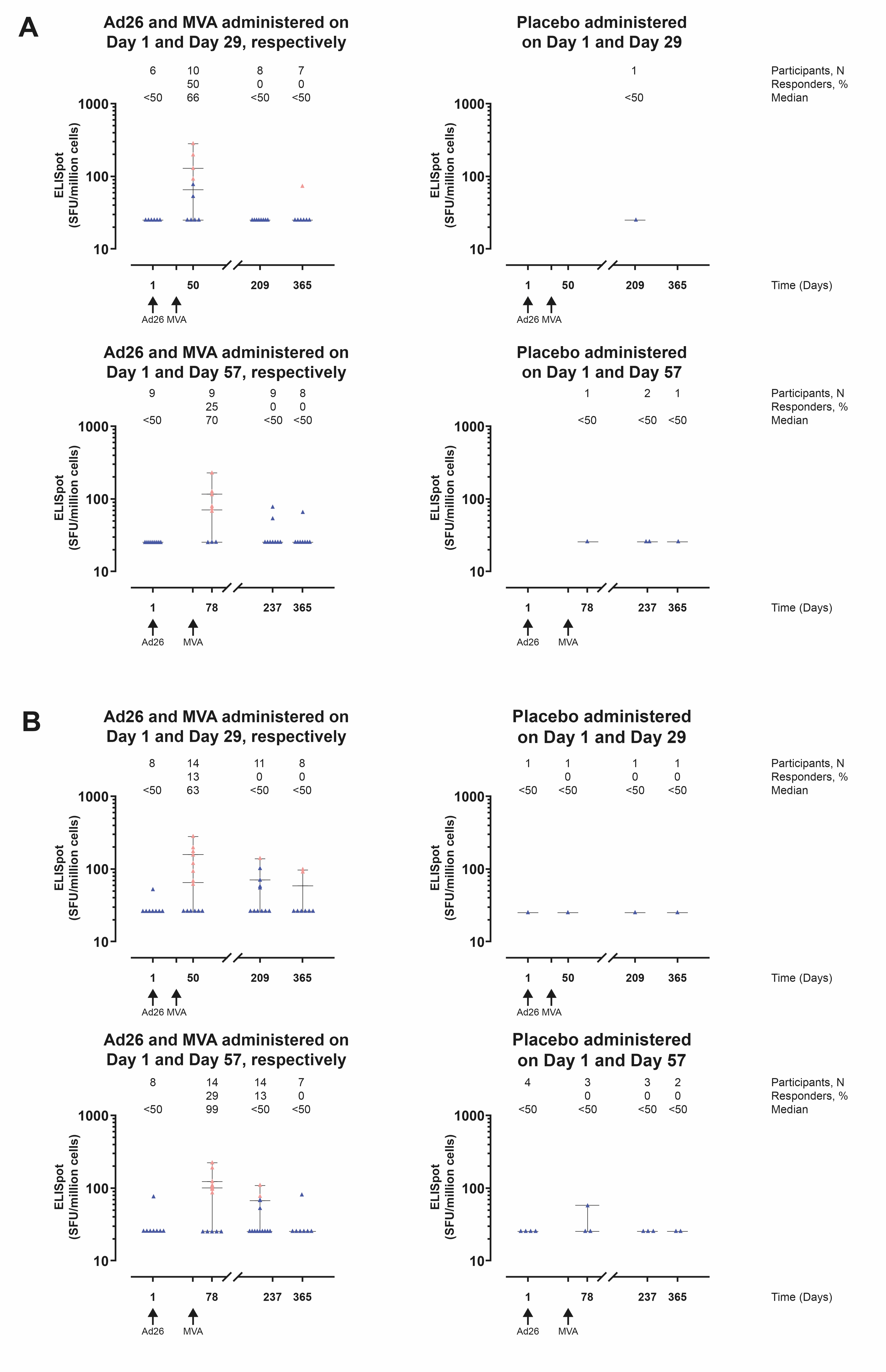


The vertical lines represent the IQR (Q1–Q3), where the horizontal lines depict the lower quartile (Q1), and upper quartile (Q3). The middle horizontal line depicts the median (Q2).

Ad26: Ad26.ZEBOV at a dose of 5x10^10^ vp; MVA: MVA-BN-Filo at a dose of 1x10^8^ Inf.U.

EBOV, Ebola virus; ELISpot, enzyme-linked immunospot; GP, glycoprotein; IFN-γ, interferon- γ; SFU, spot-forming units.

N, number of participants with available data at that time point.
